# Mycobacterium tuberculosis PknK Substrate Profiling Reveals Essential Transcription Terminator Protein Rho and Two-Component Response Regulators PrrA and MtrA as Novel Targets for Phosphorylation

**DOI:** 10.1128/spectrum.01354-21

**Published:** 2022-04-11

**Authors:** Vandana Malhotra, Blessing P. Okon, Akash T. Satsangi, Sumana Das, Uchenna Watson Waturuocha, Atul Vashist, Josephine E. Clark-Curtiss, Deepak Kumar Saini

**Affiliations:** a Department of Biochemistry, Sri Venkateswara College, University of Delhi, New Delhi, India; b Center for Infectious Diseases and Vaccinology, Biodesign Institute, Arizona State University, Tempe, Arizona, USA; c Department of Molecular Reproduction, Development and Genetics, Indian Institute of Science, Bangalore, India; d Department of Biotechnology, All India Institute of Medical Sciences, New Delhi, India; Indian Institute of Science Bangalore

**Keywords:** *M. tuberculosis*, serine/threonine protein kinase, two-component system (TCS) response regulators, transcription terminator, metabolic adaptation, phosphorylation, *Mycobacterium tuberculosis*

## Abstract

The Mycobacterium tuberculosis protein kinase K regulates growth adaptation by facilitating mycobacterial survival in response to a variety of *in vitro* and *in vivo* stress conditions. Here, we further add that *pknK* transcription is responsive to carbon and nitrogen starvation signals. The increased survival of an M. tuberculosis Δ*pknK* mutant strain under carbon- and nitrogen-limiting growth conditions compared to the wild-type (WT) H37Rv suggests an integral role of PknK in regulating growth during metabolic stress. To identify the downstream targets of PknK-mediated signaling, we compared phosphoproteomic and transcription profiles of mycobacterial strains overexpressing WT and phosphorylation-defective PknK. Results implicate PknK as a signaling protein that can regulate several enzymes involved in central metabolism, transcription regulation, and signal transduction. A key finding of this study was the identification of two essential two-component response regulator (RR) proteins, PrrA and MtrA, and Rho transcription terminator, as unique targets for PknK. We confirm that PknK interacts with and phosphorylates PrrA, MtrA, and Rho *in vivo*. PknK-mediated phosphorylation of MtrA appears to increase binding of the RR to the cognate probe DNA. However, dual phosphorylation of MtrA and PrrA response regulators by PknK and their respective cognate sensor kinases *in vitro* showed nominal additive effect on the mobility of the protein-DNA complex, suggesting the presence of a potential fine-tuning of the signal transduction pathway which might respond to multiple cues.

**IMPORTANCE** Networks of gene regulation and signaling cascades are fundamental to the pathogenesis of Mycobacterium tuberculosis in adapting to the continuously changing intracellular environment in the host. M. tuberculosis protein kinase K is a transcription regulator that responds to diverse environmental signals and facilitates stress-induced growth adaptation in culture and during infection. This study identifies multiple signaling interactions of PknK and provides evidence that PknK can change the transcriptional landscape during growth transitions by connecting distinctly different signal transduction and regulatory pathways essential for mycobacterial survival.

## INTRODUCTION

Despite all efforts, tuberculosis (TB) continues to be a global health concern. A hallmark of the disease is the ability of Mycobacterium tuberculosis to persist in the host for years, causing an asymptomatic infection called latent tuberculosis infection (LTBI). By mechanisms not entirely understood, M. tuberculosis is able to transition from active growth to dormancy (1) and can persist for extensive periods of time, with the potential of causing reactivation disease in the elderly or immunocompromised individuals. Preventing the progression of LTBI to active TB disease is an important public health goal that would substantially reduce TB transmission. The differences in drug susceptibilities of replicating and nondividing bacterial cells (2, 3) and the inability to diagnose and target LTBI are probably the greatest limitations of current therapy. Thus, it is imperative to understand the mechanisms that drive and regulate mycobacterial growth and persistence.

To survive within the human host, M. tuberculosis must process vast amounts of environmental information to generate sophisticated responses that enable metabolic shifts during initial infection and during establishment of latency (reviewed in reference 4). At the core of responsive signal transduction events is the recognition of external signals and their conversion into specific transcriptional activation or repression processes. Annotation of the M. tuberculosis genome revealed the presence of more than 200 genes involved in cellular communication and information processing with two-component systems (TCSs) and “eukaryotic-like” serine/threonine protein kinases (STPKs) as major contributors (5, 6). These signaling proteins regulate diverse cellular functions ranging from mycobacterial cell growth, division, metabolism, and nutrient uptake to virulence and persistence.

In comparison to the classic TCSs, eukaryotic-like STPKs offer an exquisite level of control by rapidly phosphorylating and dephosphorylating substrate proteins. M. tuberculosis possesses 11 STPKs, of which nine are transmembrane and are classified in three groups (PknA/B/L, PknF/I/J, and PknD/E/H), whereas the remaining two, PknG and PknK, are soluble/membrane-associated kinases (5–7). The kinase activities of all the M. tuberculosis STPKs have been characterized, and they have been shown to function as key regulators of cell shape, cell division, sugar uptake, nitrogen metabolism, and transcription (reviewed in references 8 and 9). Although several STPKs including PknK regulate mycobacterial growth (7, 10–15), our knowledge about the activating signal(s), interacting proteins, and underlying mechanisms of how these signal transduction networks coordinate metabolic and physiological adaptations in mycobacteria is still limited.

Our research efforts have focused on the functional characterization of PknK, a transcriptional regulatory STPK of M. tuberculosis. Thus far, we have elucidated several structural and functional aspects of PknK-mediated growth regulation *in vitro* and *in vivo.* Results from our studies have established that PknK participates in regulatory pathways that slow growth of M. tuberculosis in a variety of *in vitro* stress environments and during persistent infection in mice (7). PknK mediates transcriptional reprogramming of M. tuberculosis by regulating tRNA expression during logarithmic and stationary growth phases in broth cultures as a means to facilitate adaptation to changing energy requirements during growth (14). Furthermore, overexpression of wild-type (WT) *pknK_Mtb_* but not *pknK_Mtb_* K55M (phosphorylation-defective kinase wherein lysine at position 55 is substituted with methionine) in Mycobacterium smegmatis severely inhibits the rate of growth and colony development (14). Mechanistically, PknK directly inhibits *in vitro* transcription and translation through a phosphorylation-dependent mechanism (14); however, the details are largely unknown.

It is apparent that the molecular events that define the entry of M. tuberculosis into a state of persistence require an intricate network of signaling proteins that coordinate transition into a slow-growing or dormant phase. Several TCS response regulator (RR) genes have been shown to be either constitutively (*pdtaR* and *mtrA*) or differentially (*prrA*, *mprA*, *devR*, *regX3*, *phoP*, *kdpE*, *trcR*, and *trcX*) expressed during growth in human macrophages, highlighting their involvement in mechanisms that support mycobacterial adaptation and survival within the host (16). We hypothesize that PknK-mediated phosphorylation is an integral component of these growth regulatory networks. However, the repertoire of M. tuberculosis target proteins phosphorylated by PknK is limited to VirS transcription regulator (17), FabD (18), and PtkA kinase (19) with roles in cell wall synthesis, lipid metabolism, and pathogenesis. To identify novel substrates of PknK and to gain insights into its role as a regulator of mycobacterial growth, we compared the transcription and phosphoprotein profiles of recombinant M. smegmatis strains overexpressing WT and phosphorylation-defective PknK_Mtb_ protein. We report several crucial signaling interactions of PknK that not only reveal its ability to connect atypical signal transduction pathways but also highlight its role in regulation of metabolic homeostasis.

## RESULTS

### Transcription of M. tuberculosis *pknK* is responsive to carbon and nitrogen starvation.

**FIG 1:**
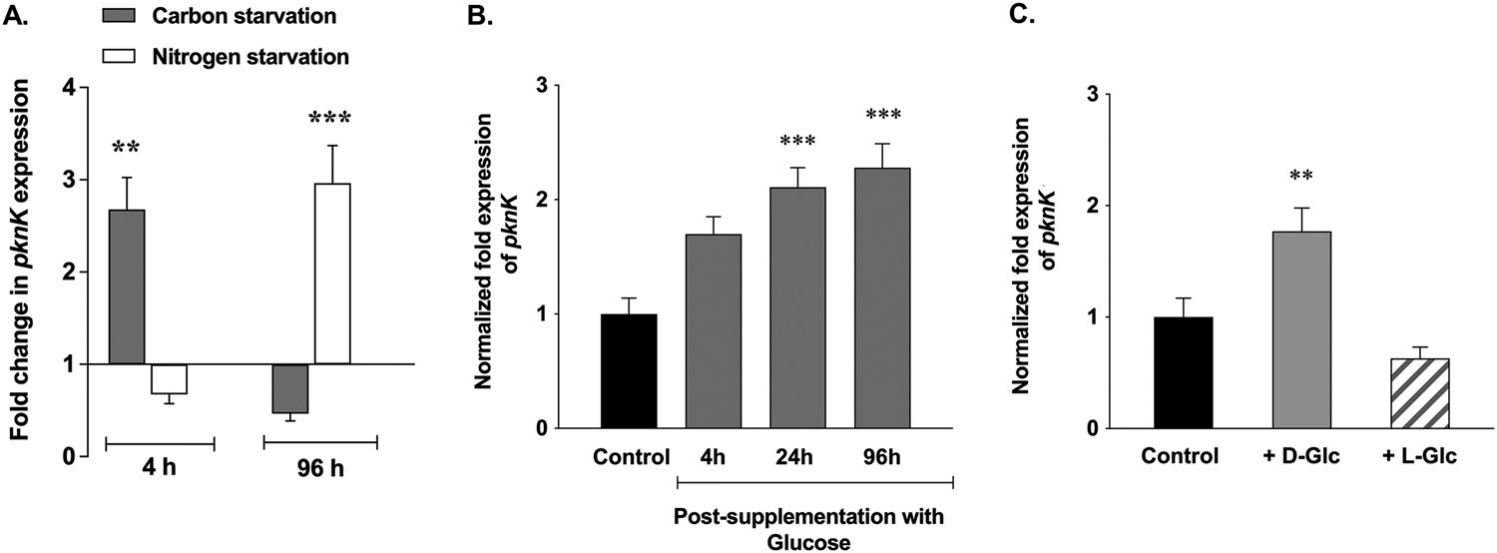
Link between PknK and regulation of carbon and nitrogen metabolism. (A) qRT-PCR analysis of *pknK* expression in M. tuberculosis H37Rv exposed to carbon starvation or nitrogen-limiting environments. The fold change in *pknK* expression in H37Rv grown under metabolic stress conditions with respect to H37Rv grown in stress-free medium (untreated control) (baseline expression set to 1.0) is presented as the mean ± SD from three independent experiments. ** and *** represent *P* < 0.01 and *P* < 0.001, respectively, for the differences in expression between H37Rv grown under stress condition and untreated control. (B and C) qRT-PCR for *pknK* expression in RNA isolated from carbon-starved cultures supplemented with 0.2% d-glucose and grown for 4 h, 24 h, and 96 h or supplemented with 0.2% l-glucose (nonmetabolized form of glucose). The unspiked cultures served as control in these experiments. Normalization of expression was done using 16S rRNA as an internal control, and data are presented as mean ± SD from two independent experiments. ** and *** represent *P* < 0.01 and *P* < 0.001, respectively, for the differences in expression between carbon-starved H37Rv supplemented with d-glucose and the unspiked control.

Previously, we reported that *pknK* transcription is induced by *in vitro* environments such as stationary phase (7). To elaborate on the role of PknK in nutrient stress adaptation, we investigated if M. tuberculosis *pknK* expression was regulated by signals of carbon or nitrogen stress. Using quantitative reverse transcription-PCR (qRT-PCR), *pknK* transcripts under stress conditions were quantified relative to the expression in the stress-free control medium. Interestingly, with carbon and nitrogen stress, a biphasic profile of *pknK* expression was observed (Fig. 1A). During carbon starvation, *pknK*-specific transcripts were upregulated at 4 h (∼2.7-fold) but decreased by 96 h (Fig. 1A). In contrast, under conditions of nitrogen stress, *pknK* transcripts were repressed at 4 h but significantly induced at 96 h (∼3.0-fold, Fig. 1A). To gain insights into PknK function under metabolic stress conditions, we compared the growth phenotypes of M. tuberculosis H37Rv, Δ*pknK* mutant, and complemented mutant strains under carbon- and nitrogen-limiting conditions for 5 days. Viable count estimations revealed increase in the CFU of mutant cells compared to H37Rv or complemented mutant in carbon- and nitrogen-limiting medium (see Fig. S1 in the supplemental material), implicating PknK in nutrient stress-induced growth adaptation.

### PknK function is linked to carbon utilization pathways.

Given that carbon metabolic pathways are integrated with all cellular pathways and that *pknK* transcription is responsive to carbon starvation signals (Fig. 1A), we questioned if PknK was involved in regulation of carbon utilization/uptake pathways. Since the carbon stress medium used in these experiments is devoid of dextrose, we analyzed *pknK* expression in carbon-starved cultures that were supplemented with d-glucose. M. tuberculosis was cultured in carbon-limiting medium for 2 days as described in Materials and Methods before being supplemented with 0.2% d-glucose and further grown aerobically. We rationalized that supplementation of a carbon source (here, glucose) in starved cells would lead to induction of carbon utilization pathways and that if PknK is involved in carbon metabolism, it will also result in induction of *pknK* expression. As expected, *pknK* transcripts were induced progressively at 4 h (∼1.7-fold), 24 h (∼2.1-fold), and 96 h (∼2.3-fold) after addition of d-glucose (Fig. 1B). To eliminate the possibility that the mere presence of carbon could also yield similar results, we repeated the experiment with l-glucose, a nonmetabolized carbon source. As shown in Fig. 1C, there was no induction of *pknK* expression after spiking carbon-starved cells with l-glucose. These results support our hypothesis that it is not the presence of carbon in the medium but its utilization or uptake that is linked to PknK function. While these observations may seem relevant to carbohydrate metabolism specifically, we cannot exclude the possibility of a broader role of PknK in carbon metabolism.

### Substrate profiling for PknK using 2D-DIGE.

To identify target proteins for PknK, we exploited the dramatic growth retardation phenotype observed in earlier studies with recombinant M. smegmatis strains overexpressing WT *pknK_Mtb_* (14). Whole-cell lysates of vector control (VC) and overexpression strains, WT_OE_ (LIX79) and Mut_OE_ (LIX80) strains, were prepared from respective cell pellets at 96 h after induction with acetamide and subjected to two-dimensional differential gel electrophoresis (2D-DIGE) analysis.

**FIG 2:**
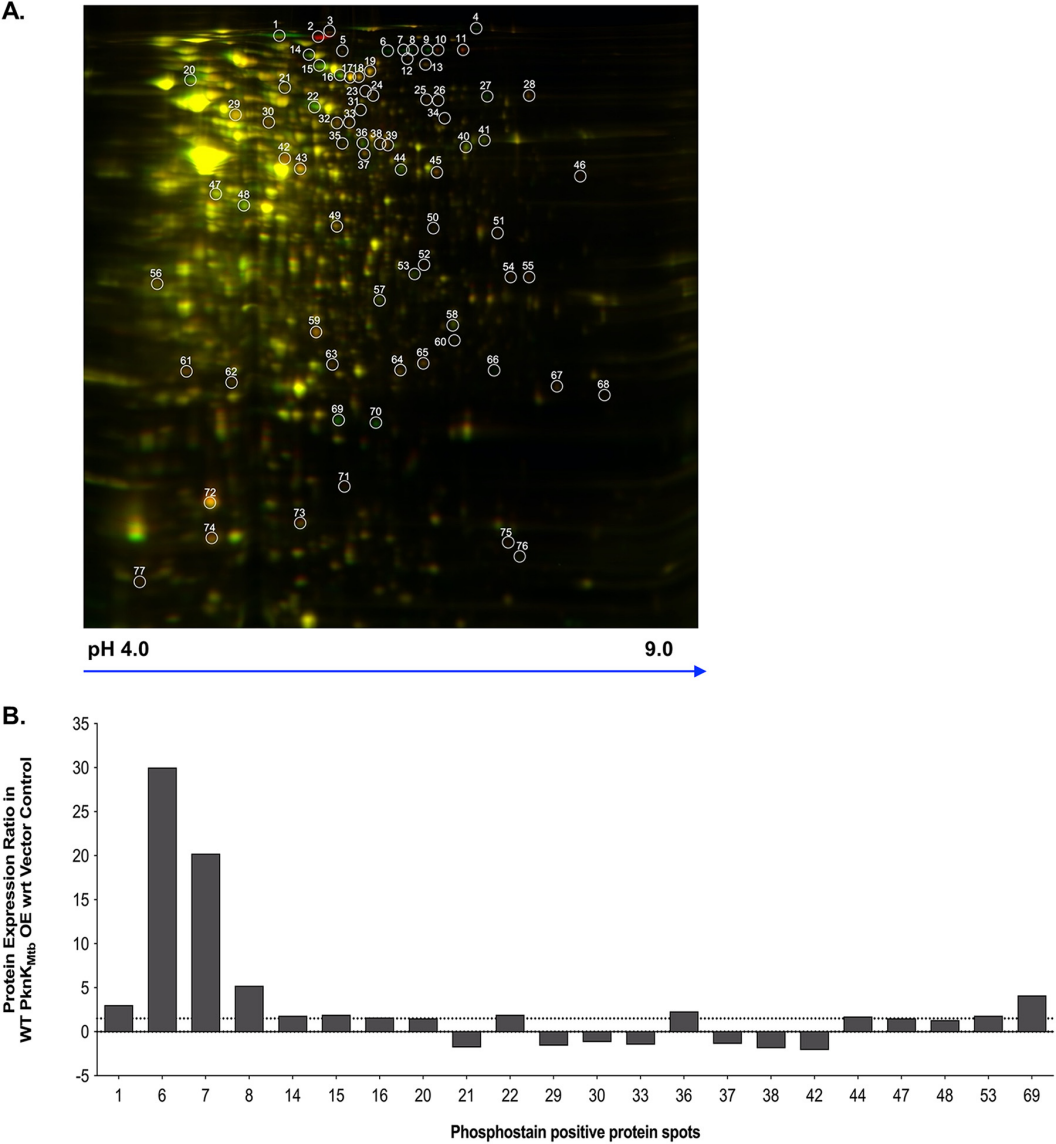
2D-DIGE of M. smegmatis strains overexpressing WT and phosphorylation-defective PknK. (A) Overlay of scanned images depicting differential expression in WT_OE_ (in green)/Mut_OE_ (in red). The spots that are present in both strains appear yellow. Data from two technical replicates were analyzed by 2D-DIGE. One representative gel is shown with well-resolved spots marked in white. (B) Graphical representation of the expression ratios of proteins that were positive for phosphostaining with the ProQ Diamond stain in the WT_OE_ strain. The positive and negative values indicate up- and downregulated proteins, respectively (cutoff ≥1.5-fold).

Proteomic comparisons of image overlays revealed spots that were expressed because of elevated levels of WT PknK_Mtb_ (in red) relative to the control (in green) (see Fig. S2, left panel). Similarly, Mut_OE_ image overlay revealed protein spots that were expressed as a result of K55M mutant kinase overexpression (in red) relative to the control (in green) (Fig. S2, center panel). Image overlay between Mut_OE_ and WT_OE_ overexpression strains highlighted differentially expressed proteins between mutant (in red) and wild-type (in green) cells (Fig. S2, right panel). Note that yellow spots indicate proteins that are expressed common in both strains, but green spots point toward proteins that are uniquely expressed as a result of WT *pknK_Mtb_* overexpression only. As shown in Fig. 2A, 77 well-resolved spots were analyzed for up- or downregulation in the comparison pairs WT_OE_/vector control and Mut_OE_/vector control using the DeCyder software. The complete list of expression ratios for 77 spots is included as Table S2 in the supplemental material. More than 50% of spots were reduced in the WT_OE_ strain. Among the spots that showed enrichment, only spots 1, 4, 6 to 9, 27, 36, 66, 69, and 70 were high-intensity spots while others were only marginally higher (Table S2). Spots with differential intensities of ≥1.5-fold were included for analysis.

Next, we analyzed these spots for their phosphostaining status by staining the gel with ProQ Diamond phosphoprotein stain. Phosphostaining analysis revealed 22 spots that were positive for phosphostaining (Fig. 2B and see Fig. S3) and were also differentially expressed in the WT_OE_ and Mut_OE_ strains (Table S2). We observed that the protein amount does not necessarily correlate with the intensity of phosphostaining signal, suggesting that a less abundant protein may be extensively phosphorylated and vice versa.

### Target identification by MS methods.

Based on the signal intensities, their phosphostain status, and resolution, 18 spots were selected and subjected to mass spectrometry analysis. The significant upregulation of spot 2 in the Mut_OE_ strain but not in the WT_OE_ strain is intriguing, and thus, it was also included for mass spectrometric (MS) analysis (Tables S2 and S3). Table 1 shows the protein ratios (Mut_OE_/WT_OE_), corresponding phosphostain status, and identity of the spots as determined by matrix-assisted laser desorption ionization–time of flight (MALDI-TOF) MS and TOF/TOF tandem MS/MS analysis. Detailed MS analysis is included as Table S3. Both spot 1 and spot 2 were identified as the M. tuberculosis PknK protein; however, spot 2 is observed only in Mut_OE_ cells. Since phosphorylation of a protein would shift its mobility toward acidic pH, we reasoned that spot 1 was phosphorylated PknK_Mtb_ and spot 2 was the phosphorylation-defective K55M PknK_Mtb_ protein. These observations were validated by phosphostaining wherein no signal was detected with regard to (wrt) spot 2 in mutant cells. Interestingly, spots 6, 7, and 9 were identified as the same protein, Rho transcription terminator (MSMEG_4954). While spots 6, 7, and 8 were positive for phosphostaining, spot 9 was not. We infer that spots 6 and 7 are potentially differentially modified forms of the same protein having different mobilities with spot 9 as the unmodified form. Although we were not able to identify (ID) spot 8 due to technical limitations, there is a high probability that it is also an isoform of the Rho terminator protein. Similarly, spots 69 and 70 of different mobilities were identified as a hypothetical protein (MSMEG_5010), which was reduced in the mutant cells. We noted that spot 69 was positive for the phosphostain (Fig. 2B and Table S2) while spot 70 was negative, suggesting that it may be the unmodified form.

**TABLE 1:** Identification of candidate PknK target proteins by 2D-DIGE and mass spectrometry^*e*^

Spot no.	Mut_OE_/WT_OE_ ratio^*a*^	Positive for phosphostaining (Y/N)^*b*^	Protein ID	Description	Protein score C.I.%^*c*^	Total ion C.I.%^*d*^
4	−2.4	N	MSMEG_4936	ATP synthase beta subunit	0	99
6	−27.6	Y	MSMEG_4954	Transcription terminator Rho protein	100	100
7	−22.2	Y	MSMEG_4954	Transcription terminator Rho protein	100	100
9	−14.5	N	MSMEG_4954	Transcription terminator Rho protein	100	100
11	5.3	N	MSMEG_2776	1-Deoxy-d-xylulose-5-phosphate synthase	0	96
14	−2.2	Y	MSMEG_6091	ClpC/MecA, ClpC ATPase family	100	100
15	−2.2	Y	MSMEG_1654	Isocitrate dehydrogenase	100	100
16	−2.6	Y	MSMEG_2299	Ribonucleotide-diphosphate reductase α subunit	100	100
20	−1.7	Y	MSMEG_0234	Metallopeptidase	100	100
22	−2.3	Y	MSMEG_5119	1-Pyrroline-5-carboxylate dehydrogenase	100	100
27	−3.9	N	MSMEG_5042	ATP-dependent RNA helicase	100	100
36	−2.3	Y	MSMEG_1183	Histidine ammonia-lyase	100	100
44	−2.0	Y	MSMEG_0373	Acetyl-CoA acetyltransferase	100	100
53	−1.7	Y	MSMEG_1878	S30AE family protein	100	100
69	−5.7	Y	MSMEG_5010	Hypothetical protein	100	100
70	−7.0	N	MSMEG_5010	Hypothetical protein	100	100

a Ratio of spot intensity signal obtained in Mut_OE_/WT_OE_ cells. Negative sign indicates downregulation in mutant cells versus wild-type cells and vice versa.

b Phosphostain status of the spot as determined by ProQ Diamond phosphoprotein staining. Y, yes; N, no.

c Confidence (%) of the protein ID calculated from MS data. Scores of >95% are significant.

d Confidence (%) of the protein ID calculated from MS/MS data. Scores of >95% are significant.

e Shaded rows represent spots that have the same identity in MS and MS/MS but have different pIs and migrations possibly due to phosphorylation.

Among other spots that were lower in the Mut_OE_ strain but not in the WT_OE_ strain and were also positive for the phosphostain, we identified ClpC/MceA protease (MSMEG_6091), isocitrate dehydrogenase (ICDH) (MSMEG_1654), ribonucleotide diphosphate reductase alpha-subunit (MSMEG_2299), metallopeptidase (MSMEG_0234), 1-pyrroline 5-carboxylate dehydrogenase (MSMEG_5119), histidine ammonia lyase (MSMEG_1183), acetyl coenzyme A (CoA) acetyltransferase (MSMEG_0373), and S30AE family protein (MSMEG_1878) as candidate PknK targets (Table 1 and Table S3). These data further implicate a potential role for PknK in the regulation of central metabolic pathways.

### Cellular abundance of PknK_Mtb_ directs temporal changes in transcription profiles that are consistent with viability.

**FIG 3:**
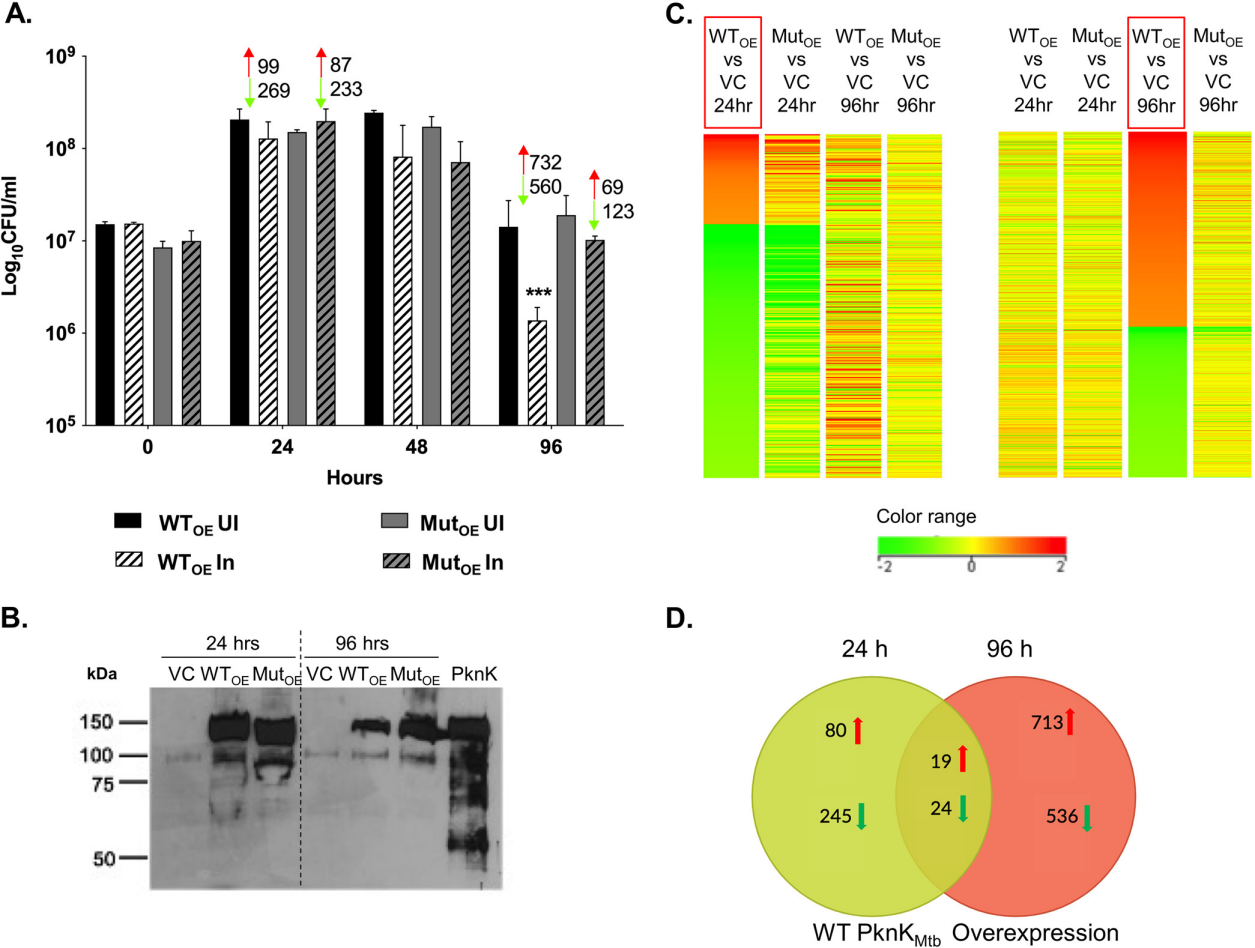
Characterization of PknK overexpression strains. (A) Growth profile of PknK_Mtb_ WT_OE_ and Mut_OE_ strains and its correlation with the number of differentially expressed genes from microarray analysis (red indicates upregulation and green indicates downregulation). Viable counts (mean ± SD) from 3 independent experiments are plotted. *** represents *P* < 0.001 for the decrease in CFU of WT_OE_ at 96 h compared to the empty-vector control. UI, uninduced; In, induced. (B) Immunoblot analysis of VC, WT_OE_, and Mut_OE_ whole-cell lysates at 24 h and 96 h postinduction for PknK levels using rabbit anti-PknK antibody (1:3,000). Purified PknK protein served as a positive control. (C) Heat map of DEGs in WT_OE_ at 24 h and 96 h postinduction (boxed in red). Color bar indicates a range of log_2_ fold changes of repression (green) and induction (red) from −2 and +2, respectively. (D) Venn diagram highlighting the number of genes in WT_OE_ strains that are unique and common to the 24-h (green circle) and 96-h (red circle) time points. The arrows denote up- or downregulation.

To better understand the transcription regulatory role of PknK as a function of growth, we characterized the WT_OE_ or Mut_OE_ strain relative to the VC strain at 24 h and 96 h after induction of *pknK* expression at 3 levels: CFU differences, PknK levels, and the differential gene expression profiles by microarrays. Viable counts were determined for recombinant strains, both uninduced and induced for *pknK* expression over a period of 96 h. RNA and protein were isolated from the same batch of cultures at the marked time points (24 h and 96 h postinduction). In agreement with previous results, no significant effect on growth was observed at the 24-h postinduction time point (14) (Fig. 3A). However, there were significant changes in the transcriptional profiles, with many genes being repressed in both WT_OE_ and Mut_OE_ strains. It is possible that at 24 h postinduction, the cells undergo adaptive changes that are reflected in the expression profiles; however, these changes do not affect viability.

Interestingly, at 96 h postinduction, WT PknK_Mtb_ significantly inhibited growth whereas no effect was observed due to the phosphorylation-defective protein (Fig. 3A). These data corroborate earlier observations that growth inhibition due to PknK is a function of its kinase activity (14). This growth defect at 96 h when correlated with the transcriptional changes mediated by WT protein revealed variations in the expression of many genes (upregulated, 732; downregulated, 560). Interestingly, 192 genes were seen to be differentially regulated in the Mut_OE_ strain, pointing toward a transcription modulation ability of PknK that is independent of its kinase function.

Western blot analysis of whole-cell lysates of M. smegmatis overexpressing WT and mutant kinase was done using rabbit anti-PknK antibody as described previously (7). At 24 h, amounts of WT PknK_Mtb_ and K55M mutant proteins were similar in WT_OE_ and Mut_OE_ cell lysates, respectively (Fig. 3B, lanes 2 and 3).

### Novel candidate targets of PknK include signaling proteins, sigma factor, transcription terminator, and genes involved in central metabolism.

Overexpression of WT *pknK_Mtb_* and K55M *pknK_Mtb_* resulted in a significant shift in the transcriptional landscape as a function of growth. This is evident from the fact that the transcriptome changed from a repressive one at 24 h (*n* = 502 downregulated) to a primarily inducive one at 96 h (*n* = 801 upregulated) time point (Fig. 3A and C and Table S4). The complete data sets of differentially expressed genes (DEGs) in wild-type and mutant overexpression strains are included in Tables S5 to S8. To ascertain if there was a set of core genes that were regulated by PknK during growth transitions, we compared profiles obtained with the WT_OE_ strain at both 24-h and 96-h time points. As evident from the heat maps and Venn diagram (Fig. 3C and D), the data sets of DEGs at 24 h and 96 h during growth of the WT_OE_ strain showed very little overlap (19 upregulated and 24 downregulated) between time points, indicating that PknK mediates changes that are distinctly different and suggestive of large-scale shifts in metabolic pathways during growth transitioning events.

**FIG 4:**
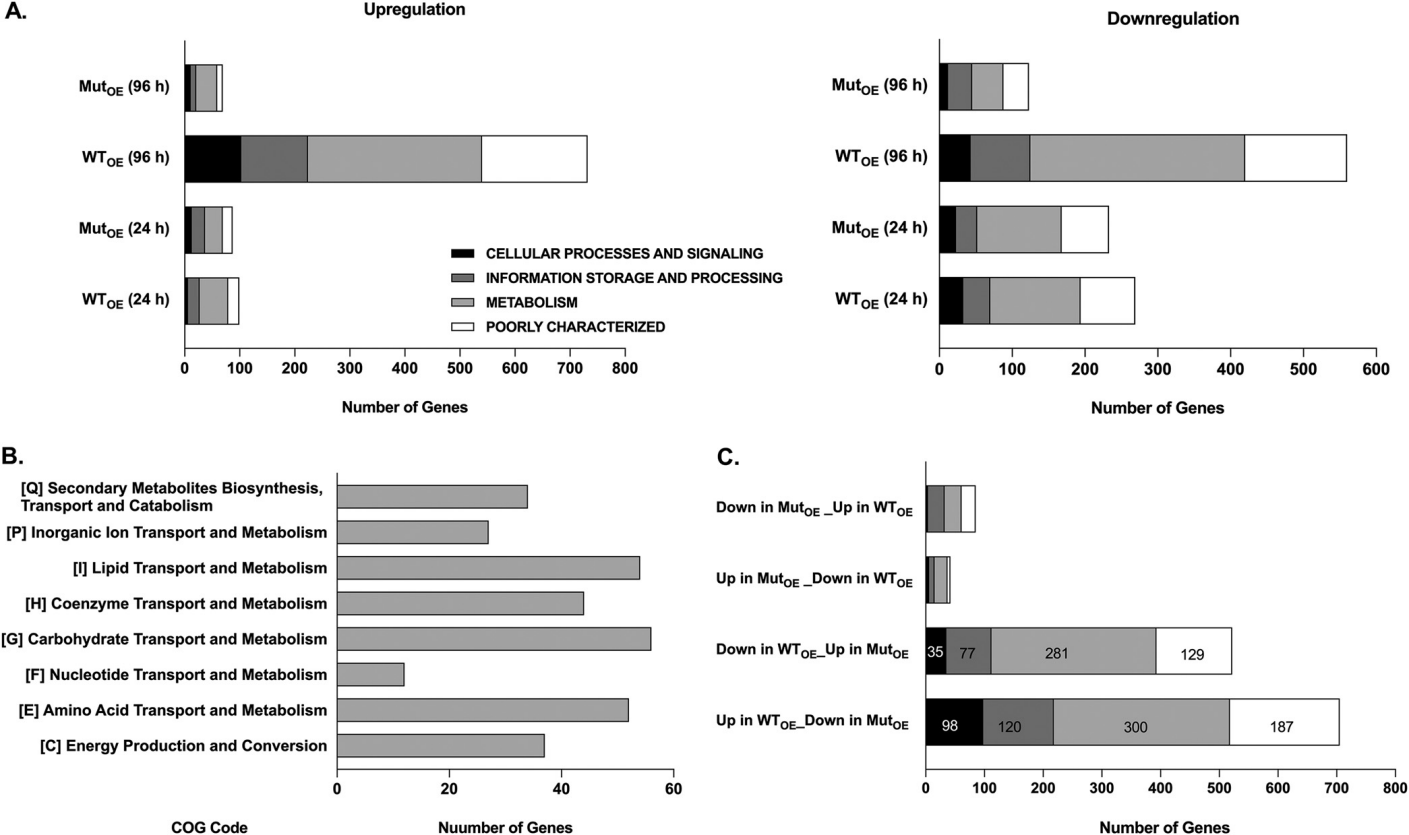
Functional categorization of DEGs. The microarray data were divided into 4 categories, namely, cellular processes and signaling, information storage and processing, metabolism, and poorly characterized. (A) Graphical representation for the number of genes that were upregulated (left panel) and downregulated (right panel) along with their category-wise distribution at 24 h and 96 h postinduction during growth in the various data sets as indicated. (B) Distribution of genes upregulated in the WT_OE_ strain at 96 h under the metabolism category per the COG codes. (C) Graphical representation of the number of genes that are upregulated or downregulated in the WT_OE_ strain but not in the Mut_OE_ strain at the 96-h time point.

The DEGs were analyzed and classified based on the Clusters of Orthologous Groups (COG) functional category classification as described in Materials and Methods. The up- or downregulated genes were categorized into 4 main groups: cellular processes and signaling, information storage and processing, metabolism, and genes that are poorly characterized. As shown in Fig. 4A, left panel, a significant proportion of the 732 genes that were upregulated at 96 h in the WT_OE_ strain are either involved in cellular metabolism (*n* = 316) or poorly characterized (*n *= 192) followed by information storage & processing (*n* = 121) and cellular processes and signaling (*n* = 103). A similar trend was observed with downregulated genes (Fig. 4A, right panel). Further inspection of the 316 genes belonging to the cellular metabolism category revealed a maximum number of genes involved in lipid, carbohydrate, and amino acid metabolism (Fig. 4B), alluding to a role for PknK in carbon and nitrogen metabolic pathways.

Using the rationale that genes that were up- or downregulated in the WT_OE_ strain at 96 h but not in the Mut_OE_ strain would help identify downstream effects of PknK-mediated phosphorylation, we screened for genes whose upregulation seemed phosphorylation dependent (Fig. 4C). A complete list of these genes is provided in Table S9. In this study, we focused on genes involved in signaling pathways and transcription regulation. We rationalized that phosphorylation of multiple transcription-regulatory proteins could be the reason for such global changes in downstream gene expression observed in microarrays. Among the most notable putative targets were the genes encoding two-component response regulators MSMEG_0244 (*prrA*; Rv0902c) and MSMEG_1874 (*mtrA*; Rv3746c), transcription terminator protein MSMEG_4954 (*rho*; Rv1297), phosphotyrosine protein phosphatase MSMEG_0100 (*ptpB*; Rv0153), MSMEG_6091 (ClpC; Rv3596c), and alternate sigma factor MSMEG_6931 (*sigM*; Rv3911). Notably, the Rho transcription terminator and the ClpC protease were also identified as candidate target proteins in the 2D-DIGE screen (Table 1), further validating our experimental approach.

### PknK forms homodimers *in vivo* and interacts with MtrA and PrrA RRs.

Based on the transcriptomics data, we investigated the corresponding M. tuberculosis H37Rv orthologs as targets of PknK. We characterized protein-protein interactions of PknK with M. tuberculosis PrrA and MtrA response regulators using the mycobacterial protein fragment complementation (M-PFC) assay. Full-length coding sequences of M. tuberculosis *pknK* (Rv3080c), *prrA* (Rv0903c), and *mtrA* (Rv3246c) were cloned into pUAB300 and pUAB400 to generate C-terminal fusions with the complementary fragments of mouse dihydrofolate reductase (mDHFR).

**FIG 5:**
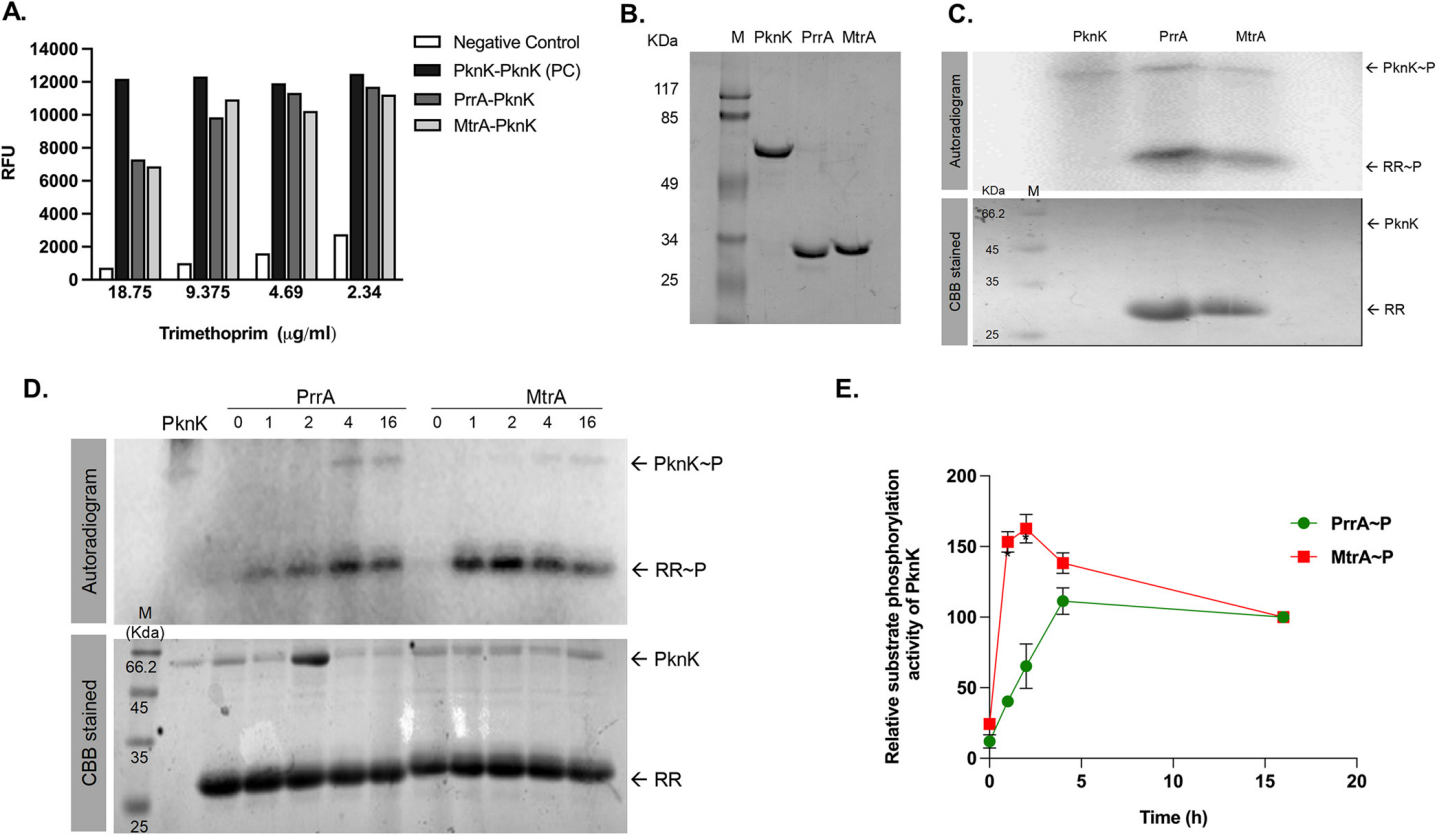
*In vivo* and *in vitro* protein-protein interactions of PknK, MtrA, and PrrA. (A) M-PFC liquid assay using alamarBlue to test interactions of PknK with PknK/PrrA/MtrA. PknK-DHFR F[1,2] and PknK-DHFR F[3] interaction was taken as positive control. Empty vector pUAB300-pUAB400::PknK served as negative control. Relative fluorescence units (RFU) obtained was plotted against concentrations of trimethoprim (TRIM) used in the assay. An average from two independent estimations is plotted. (B) SDS-PAGE analysis of recombinant purified proteins, PknK (lane 2), PrrA (lane 3), and MtrA (lane 4) along with protein marker M (lane 1). (C) Kinase activity analysis of purified PknK and RR substrate phosphorylation. (Top panel) Autoradiogram of PknK and target proteins, PrrA and MtrA. (Bottom panel) Corresponding gel image stained by Coomassie brilliant blue (CBB). (D) Time course of PknK-dependent phosphorylation of PrrA and MtrA RR proteins. (Top panel) Autoradiogram of PknK and substrate phosphorylation of PrrA and MtrA at 25°C for 0, 1, 2, 4, and 16 h. (Bottom panel) Corresponding gel image stained by CBB. A representative set of images is shown. (E) Densitometric analysis of the kinetics of PrrA and MtrA phosphorylation using the Image J software. Data from three independent replicates are plotted (mean ± standard error [SE]).

The plasmid pairs pUAB400::*pknK* and pUAB300::*pknK* or *prrA* or *mtrA* were cotransformed into M. smegmatis to generate strains carrying the following expression pairs: PknK/PknK, PknK/PrrA, and PknK/MtrA, respectively. Resistance to trimethoprim (TRIM) indicates protein-protein interaction resulting from the functional reconstitution of mDHFR. A liquid M-PFC assay was done with TRIM concentrations ranging from 2.3 to 18.75 μg/mL for identifying possible interactions (if any) of PknK with PrrA or MtrA as described in Materials and Methods. Robust interaction between PknK and PknK indicates formation of PknK homodimers *in vivo* (Fig. 5A). This observation is in agreement with our previous findings wherein we have shown that PknK undergoes oligomerization *in vitro* (14), and thus, PknK/PknK interaction was treated as a positive control (PC). As shown in Fig. 5A, both PrrA and MtrA showed strong interactions with PknK. Similar results were obtained using solid agar plates (data not shown).

### M. tuberculosis PrrA and MtrA response regulators are bona fide targets of PknK-mediated phosphorylation.

Given our finding that two essential TCS response regulators interact with an STPK, which generates an interesting signaling cascade in M. tuberculosis, we next characterized the biochemical basis for these interactions. Toward this, the kinase domain of M. tuberculosis PknK and full-length MtrA and PrrA proteins were purified to homogeneity (Fig. 5B) and used in an *in vitro* kinase assay. As shown in Fig. 5C, the PknK kinase domain (∼59 kDa) was autophosphorylated (upper panel). Substrate phosphorylation was observed for both PrrA (∼28.5 kDa) and MtrA (∼28.4 kDa) (Fig. 5C, upper panel), evident by the presence of labeled phosphate on RRs in the presence of PknK. The RR proteins do not undergo autophosphorylation (data not shown).

Time kinetics analysis was performed to determine the phosphorylation rates for PrrA and MtrA through activated PknK. The RRs were incubated with autophosphorylated PknK at 25°C for 0, 1, 2, 4, and 16 h, and relative phosphorylation levels were calculated. Results show that phosphorylation was initiated on the RRs as early as 1 h and was stable for over 16 h and 4 h for PrrA and MtrA, respectively (Fig. 5D). As the signal saturated on RRs at the 4- and 16-h time points, we were able to see faint signal on PknK as well, implying a prolonged and sustained autophosphorylated state. Densitometric quantitation revealed similar trends (Fig. 5E). Collectively, these results confirm that M. tuberculosis PknK binds to and phosphorylates M. tuberculosis response regulator proteins PrrA and MtrA.

### PknK modulates DNA binding abilities of MtrA and PrrA RRs.

**FIG 6:**
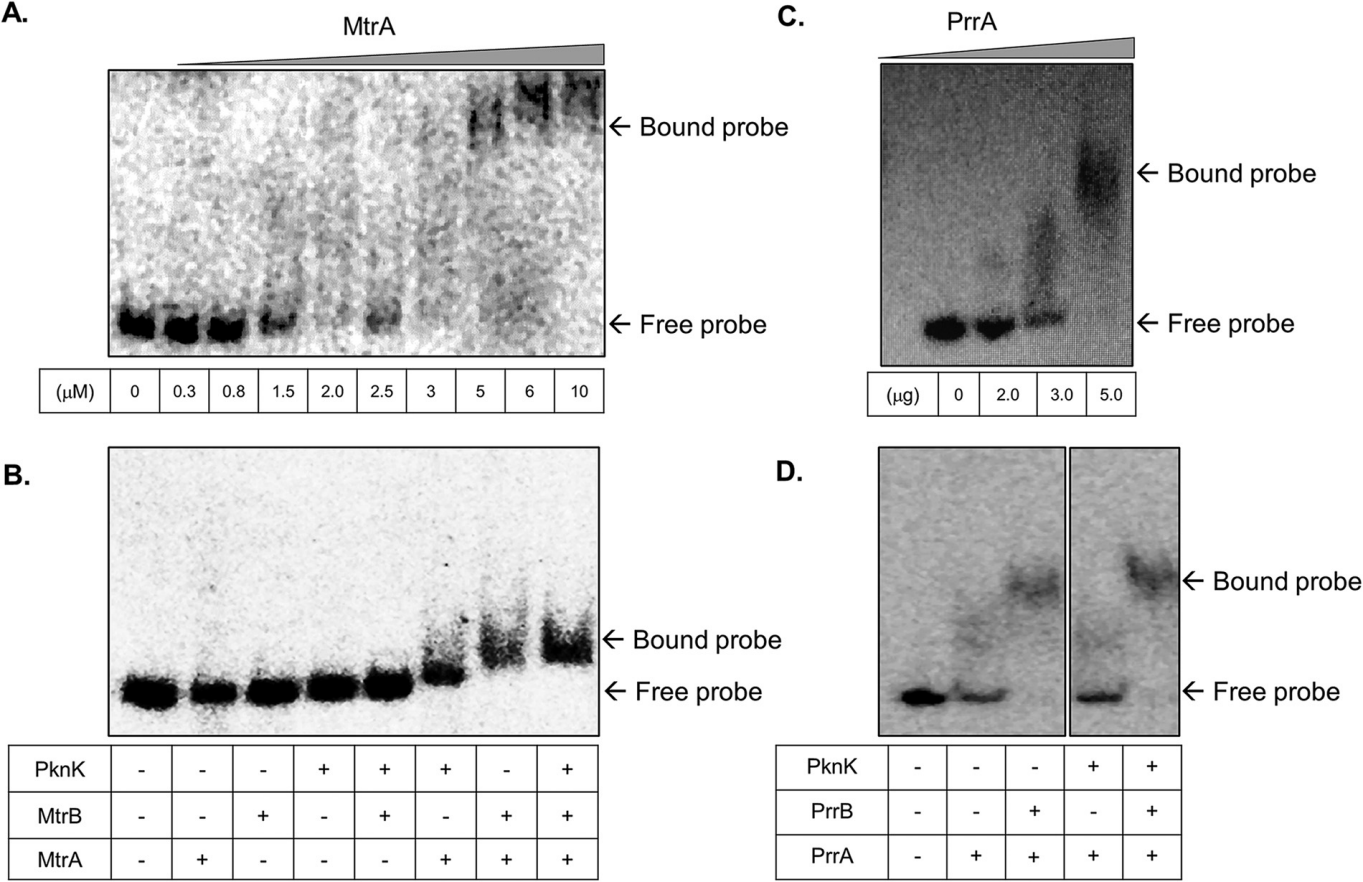
EMSAs of MtrA and PrrA binding in the presence of phosphorylation through cognate sensor kinase and PknK. (A and C) Analysis of the binding affinity of various amounts of RRs MtrA and PrrA with *oriC* DNA and *prrA* promoter region, respectively. (B and D) Analysis of the binding affinity of MtrA and PrrA RRs for their respective target DNAs in the presence of cognate SK, MtrB or PrrB, alone and in combination with STPK PknK. The SK or PknK wherever indicated is used in a phosphorylated state.

M. tuberculosis MtrA and PrrA RRs are known to be essential for mycobacterial viability (20, 21), and thus it was imperative to study the impact of PknK-mediated phosphorylation on the activities of these RRs. We investigated the effect of phosphorylation through PknK on the ability of MtrA and PrrA proteins to bind their respective target DNA through electrophoretic mobility shift assay (EMSA). Since MtrA is known to regulate cell division (22), the DNA region corresponding to the *oriC*, where it is known to bind, was used and subjected to EMSA. Initially, unphosphorylated MtrA protein was used at different concentrations to determine the minimal amount at which the protein binds to the target DNA and results in mobility shift. As seen in Fig. 6A, 1.5 μM MtrA was found to be sufficient for retardation and was used for further analysis.

It is important to mention here that while phosphorylation from the cognate sensor kinase MtrB occurs on a conserved aspartate residue, the phosphorylation from PknK can occur on one or more Ser or Thr residues, making RRs amenable to multisite phosphorylation. We recorded a distinct increase in MtrA binding to *oriC* DNA when MtrA was phosphorylated by MtrB SK (Fig. 6B, lane 7). Phosphorylation through PknK also revealed a shift; however, the retardation of the DNA-protein complex in this case was less than that observed with MtrB SK (Fig. 6B, lane 6). Interestingly, phosphorylation of MtrA in the presence of both PknK and MtrB resulted in a slightly enhanced but consistent retardation of the protein-DNA complex (Fig. 6B, lane 8), suggesting a potential additive effect of dual phosphorylation.

Next, we examined the effect of STPK-mediated phosphorylation on the binding efficiency of PrrA to its own promoter DNA. A 317-bp promoter region of *prrA* has been reported as a PrrA binding site (23). As shown in Fig. 6C, 3.0 μg (∼2.5 μM) of PrrA protein binds the target DNA and retards its mobility. An EMSA showed that in comparison to cognate phosphorylation through PrrB sensor kinase, PknK alone did not have any effect on binding of PrrA to its promoter region (Fig. 6D, lane 3). However, dual phosphorylation of Asp and Ser/Thr residues of PrrA by PrrB and PknK, respectively, revealed a nominal shift of the DNA-protein complex (Fig. 6D, lane 5). Collectively, these studies revealed, first, that MtrA∼P generated through PknK is able to bind its target DNA, albeit with less affinity than for the canonical pathway through MtrB sensor kinase (SK); second, that PknK phosphorylation does not have any impact on PrrA∼P binding to the *prrA* promoter; and lastly, that dual phosphorylation of both regulators, through their cognate SKs and PknK, resulted in slightly enhanced DNA binding abilities.

## DISCUSSION

Mycobacterium tuberculosis protein kinase K is a multidomain, transcriptional regulatory STPK that is classified as a member of the unique STAND (signal transduction ATPase with numerous domain) family of proteins involved in the integration of multiple signaling events (24, 25). Our previous studies established PknK as a key regulator of mycobacterial growth (7, 14). The fact that metabolic states during logarithmic versus stationary phase of growth have distinct requirements defined by a unique transcription and translation signature makes it noteworthy that PknK regulates growth under diverse metabolic conditions. Despite these observations, our knowledge of the identity of PknK target proteins is very limited. The focus of this study is to elucidate the PknK-regulated pathways and their components through a combination of proteomic and transcriptomic approaches. We identified targets involved in the regulation of cellular metabolism, Clp protease, sigma factor, and several proteins known to be essential for M. tuberculosis including the Rho transcription terminator and two-component system response regulator proteins, highlighting the transcription networks driven by PknK-mediated phosphorylation.

We show that in addition to stationary-phase stress (7), *pknK_Mtb_* is differentially expressed under carbon and nitrogen starvation, implicating PknK in the regulation of metabolic homeostasis. The time-dependent up-/downregulation of *pknK* transcripts in carbon and nitrogen starvation suggests its involvement in sensing C/N metabolic fluxes, aiding in the growth adaptation of mycobacteria during metabolic stress. The enhanced survival of the Δ*pknK* mutant strain under carbon- and nitrogen-limiting conditions supports this hypothesis. Furthermore, these observations draw a parallel with the microarray data wherein a significant ∼43% of the genes upregulated in the presence of WT PknK_Mtb_ were involved in central metabolism with major contributions from lipid, carbohydrate, and amino acid metabolic pathways. The addition of nutrient stress to the list of stress conditions such as stationary phase, hypoxia, and oxidative stress wherein PknK directs growth adaptation by slowing down growth (7) highlights a far-reaching role for PknK-mediated signaling in stress-induced mycobacterial growth and survival.

2D-DIGE and mass spectrometry of M. smegmatis strains overexpressing WT and mutant PknK_Mtb_ revealed a profile of PknK candidate target proteins that included several enzymes involved in central metabolism. We focused on differentially expressed candidate proteins that also exhibited positive phosphostaining in the WT_OE_ strain. Understandably, these may include both direct and indirect targets of PknK. Nevertheless, the data provide valuable insights into the elements of PknK-dependent regulatory pathways. One such example is the identification of isocitrate dehydrogenase (ICDH) as a possible PknK candidate target protein (Table 1). It is well known that transition into persistence or a state of dormancy necessitates changes in several mycobacterial metabolic pathways, of which the requirement of an active glyoxylate shunt is notable (26). Isocitrate dehydrogenase, a Krebs cycle enzyme, converts isocitrate into alpha-ketoglutarate. Interestingly, the movement of the isocitrate molecule into the glyoxylate shunt or through the tricarboxylic acid (TCA) cycle is regulated by the phosphorylation of the Escherichia coli ICDH enzyme (27). Although the existence of any such regulatory mechanism for the mycobacterial ICDH is unknown, our results indicate a distinct possibility and, moreover, highlight the function of PknK as a possible regulator switch coordinating different energy-producing pathways while facilitating carbon/nitrogen homeostasis.

In addition, we identified multiple isoforms of the phosphorylated Rho protein *in vivo*, suggesting a role of PknK in the regulation of transcription termination. Botella et al. have established that the M. tuberculosis Rho protein is required for mycobacterial growth in culture and survival during infection in mice (28). It is possible that PknK-Rho interactions may contribute toward shaping the mycobacterial transcriptome conducive for survival in the macrophage host cell environment. It is interesting that in addition to transcription termination, the Rho protein is involved in silencing gene expression in mycobacteria, particularly of genes involved in virulence (28). As new regulatory roles for the Rho protein are discovered, the possibility of Rho phosphorylation altering its function, impacting physiological processes such as regulation of gene expression, genome stability, and pervasive transcription, cannot be undermined.

Comparative transcriptomics of strains overexpressing WT and K55M mutant *pknK_Mtb_* highlighted PknK as a dual transcription regulator exhibiting repression at the 24-h time point followed by an inducive role at the 96-h time point during growth. The differential expression observed due to overexpression of the phosphorylation-defective PknK protein was surprising particularly because very little overlap was observed with the DEG patterns seen with the WT protein. It is possible that the PknK K55M mutant protein could itself be subjected to phosphorylation through other kinases and cause downstream transcriptional changes directed through protein-protein interactions.

It is known that M. tuberculosis PknK enables successful establishment of infection in host cells (7, 29). Coincidentally, *pknK* transcripts were found to be significantly upregulated at 18 h after infection of human macrophages (7). Additionally, *esxA*, encoding the ESAT-6 early antigen secreted by virulent mycobacteria during infection and known for its immunomodulatory function, and the gene encoding alternate sigma factor M, *sigM*, were found to be upregulated in cells overexpressing the WT PknK kinase (see Tables S5 and S9 in the supplemental material). Raman et al. have shown that SigM regulates the Esx secreted protein and nonribosomal peptide synthetase genes (30). It is noteworthy that nonribosomal peptides are considered drivers of mycobacterial virulence during early stages of infection (31). The possibility of SigM as a putative target of PknK is of significant interest as SigM regulates cell surface and secreted molecules that are likely to function in host-pathogen interactions.

The expansive differential regulation of ∼15% of the M. smegmatis genome observed because of WT PknK_Mtb_ implicates PknK as a nodal point connecting diverse regulatory pathways. In support of this argument, we elucidate interactions of PknK with signaling proteins belonging to the traditional two-component signaling systems. Compared to the histidine/aspartate phosphorylation by the TCS proteins, serine and threonine phosphorylations are more stable, thus having the potential to cause widespread changes in the transcriptome. While there is evidence that membrane-bound STPKs, namely, PknB and PknH, phosphorylate TCS RR proteins (31–34), there is little information on interactions of cytoplasmic kinases with signaling proteins. Recently, Mishra et al. reported phosphorylation of PrrA RR by PknG, -K, and -J kinases (35). Our data is in agreement, and we further show that M. tuberculosis MtrA and PrrA response regulators of the MtrAB and PrrAB TCSs are bona fide targets of PknK-mediated phosphorylation *in vitro* and *in vivo*. Of all the RRs, MtrA and PrrA are the only two response regulators that are essential for mycobacterial growth and viability (20, 21). Several crucial physiological processes such as DNA replication, cell division, and cell wall integrity are regulated by the MtrAB TCS (22). Acetylation of MtrA, now known to regulate its DNA binding activity and transcription in mycobacterial strains based on nutrient availability, highlights its importance in the regulation of the mycobacterial metabolome (36, 37). Contrary to the EMSA results obtained with MtrA phosphorylated by PknK, PknA/B-mediated MtrA phosphorylation has been shown to decrease its DNA binding ability (38), suggesting possible dual effects of MtrA on downstream regulation of gene expression. This is a distinct possibility as MtrA is known to exhibit both activator and repressor activities (37, 39). As observed with PknK, M. tuberculosis PrrAB TCS is required during early events of human macrophage infection (40) and, coincidentally, is also upregulated under nitrogen-limiting conditions (20). More recently, studies have established a role for M. smegmatis PrrAB in the regulation of energy and metabolic homeostasis and its dormancy response (41). Undoubtedly, the RRs of these key TCSs being targets of PknK introduces an added tier of regulation of signaling and response to such cues.

**FIG 7:**
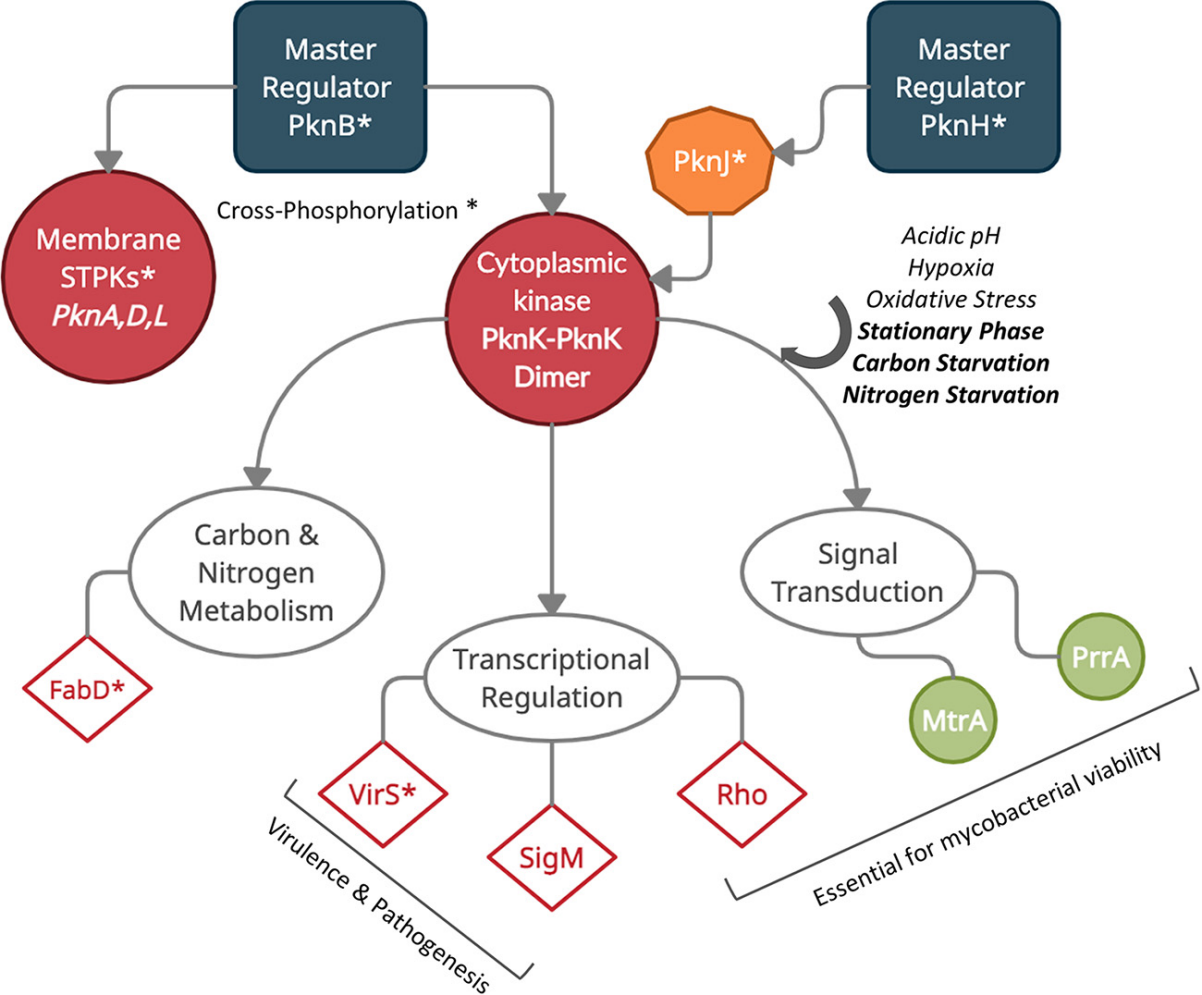
A schematic model for PknK function in signal integration and transduction. Data from other laboratories (marked with an asterisk) and our previous studies are compiled to propose a model for PknK function as a signaling node. Master regulator PknB phosphorylates membrane kinases (PknA, -D, and -L) and cytoplasmic/membrane-associated kinase (PknK) (42). PknK is also phosphorylated by PknJ, which in turn is phosphorylated by PknH, another master regulator (42). Evidence suggests that PknK enables mycobacterial growth adaptation in response to multiple stress environments such as acidic pH, hypoxia, oxidative stress, stationary phase (7), and carbon and nitrogen starvation (this study) with the latter three signals also causing an increase in *pknK* transcripts. Autoactivated PknK dimer phosphorylates downstream substrate proteins involved in major pathways of cellular metabolism, transcription regulation, and signal transduction. Among notable targets are the Rho transcription terminator and response regulator proteins, PrrA and MtrA, essential for mycobacterial growth. Although direct phosphorylation of SigM was not confirmed in this study, we have included it to highlight a potentially important link between PknK function and virulence that warrants further investigation. Other known PknK targets, VirS (17) and FabD (18), are marked with an asterisk.

Noncanonical phosphorylation of response regulators and, moreover, cross-phosphorylation events between Ser/Thr protein kinases and two-component signaling systems seem to echo the theory that signal integration in mycobacteria often occurs through multiple pathways. Based on the data presented here, we propose a model wherein a cytoplasmic kinase such as PknK could potentially act as a nodal point capable of integrating multiple signals either directly or indirectly through cross-phosphorylation via other kinases, with a plethora of regulatory proteins as downstream targets (Fig. 7). Signaling interactions of PknK with membrane receptor kinases such as PknB and PknH, also called the master regulators (42), lend support to this hypothesis. Are these interactions part of a larger scaffold assembly? What is the identity of other regulatory partners and the dynamics of these interactions? While these questions justify deeper investigations, it is clear that PknK is a key player in the complex layers of networks that govern mycobacterial growth, metabolism, and pathogenesis.

With signaling proteins being promising druggable targets (43), the notion that a central regulatory protein such as PknK interacting with essential TCSs could be a novel target for antitubercular therapy is attractive, and while this may be a speculation, there is enough evidence to continue our efforts in this direction.

## MATERIALS AND METHODS

### Strains, plasmids, and growth conditions.

The strains and plasmids used in this study are listed in Table S1 in the supplemental material. E. coli strains XL1-Blue (Stratagene) and Artic (Agilent) were used for cloning and expression of proteins, respectively. E. coli cells were grown in Luria broth and supplemented with 100 μg/mL ampicillin, 150 μg/mL hygromycin, 50 μg/mL kanamycin, and 25 μg/mL gentamicin as required and incubated at 37°C with constant shaking overnight at 150 rpm. The M. smegmatis strains LIX79 overexpressing WT *pknK_Mtb_* (henceforth referred to as WT_OE_) and LIX80 overexpressing K55M mutant *pknK_Mtb_* (henceforth referred to as Mut_OE_) along with LIX70 vector control (VC) were grown as described previously (14). Briefly, these strains were grown in LB broth supplemented with 0.05% Tween 80 with aeration or on LB agar plates at 37°C. Acetamide was added at a final concentration of 0.2% during exponential growth in liquid medium to induce *pknK_Mtb_* expression. For CFU assays, broth-grown M. tuberculosis H37Rv, Δ*pknK* deletion mutant, and complemented mutant strains were used as described previously (7). All chemicals were obtained from Sigma-Aldrich, USA, unless stated otherwise.

### Exposure to carbon and nitrogen stress.

Stress-induced expression studies were performed as described previously (20, 44). Logarithmic-phase cultures of M. tuberculosis H37Rv were centrifuged and washed with 7H9 basal medium (no supplements) before resuspension in stress-appropriate or stress-free control medium. At the indicated time points, samples were removed for RNA extraction. For carbon stress, cultures were resuspended in Middlebrook 7H9-Tween 80-albumin-NaCl medium (without dextrose). Two-day carbon-starved M. tuberculosis cultures were supplemented with 0.2% glucose (d- or l-) and further grown at 37°C. For nitrogen-limiting conditions, cells were incubated in Middlebrook 7H9-Tween 80-albumin-dextrose-saline (ADS) medium supplemented with 200 μM l-methionine *S*-sulfoximine (MSX) as described previously (21, 45). For comparison of the *in vitro* growth rates, WT H37Rv, Δ*pknK*, and complemented mutant strains were cultured aerobically at 37°C. Growth was monitored in carbon/nitrogen-limiting medium described above for 5 days. Serial dilutions of the cultures at various time points were plated on agar plates and incubated at 37°C for 3 weeks before quantification of viable counts.

### Two-dimensional differential gel electrophoresis (2D-DIGE) and mass spectrometry.

Logarithmic-phase cultures of overexpression strains, WT_OE_ and phosphorylation-defective Mut_OE_, were induced with 0.2% acetamide and further grown for 96 h and centrifuged to obtain cell pellets that were processed for 2D-DIGE. Data from two technical replicates are reported. Detailed methods for DIGE are provided in the supplemental material. Briefly, the spots of interest were picked up by an Ettan Spot Picker (Amersham BioSciences) based on the in-gel analysis and spot picking design by DeCyder software. The spots were subjected to MALDI-TOF MS and TOF/TOF tandem MS/MS on an AB Sciex TOF/TOF 5800 system (AB Sciex, Framingham, MA). Both the resulting peptide mass and the associated fragmentation spectra were submitted to a GPS Explorer workstation equipped with a Mascot search engine (Matrix Science) to search the National Center for Biotechnology Information nonredundant (NCBInr) database. Candidates with either protein score confidence interval (C.I.)% or ion C.I.% greater than 95 were considered significant.

### RNA isolation and qRT-PCR.

Aliquots for RNA isolation were taken from M. tuberculosis H37Rv cultures at 4 h and 96 h after exposure to carbon or nitrogen stress in three independent experiments. Untreated cells at the same time points served as controls. For microarrays, RNA was isolated from two independent replicates of VC, WT_OE_, and Mut_OE_ strains at 24 h and 96 h after induction with 0.2% acetamide. RNA isolation and quantitative RT-PCR (qRT-PCR) were performed as described previously (46). Total RNA integrity was assessed using a 2100 Bioanalyzer (Agilent, Palo Alto, CA) following the manufacturer’s protocol.

### Microarrays.

Detailed methods for microarrays are provided in the supplemental material. Briefly, the samples were labeled using an Agilent Quick Amp kit (part number 5190-0444). Total RNA was reverse transcribed using random hexamer primer tagged to T7 promoter sequence. cDNA thus obtained was converted to double-stranded cDNA in the same reaction. Further, the cDNA was converted to cRNA in the *in vitro* transcription step using T7 RNA polymerase enzyme, and Cy3/Cy5 dye was added into the reaction mix. The labeled cRNA samples were hybridized onto an Agilent Custom M. smegmatis 8x15k gene expression array designed by Genotypic Technology Pvt. Ltd. (AMADID: 043029). Hybridization and feature extraction were done as described previously (47). Genes upregulated >1.0 (logbase_2_) and downregulated <−1.0 (logbase_2_) in the test samples with respect to control sample were identified. A dye-swap experiment for two replicates was performed to nullify dye biasness. Statistical Student *t* test *P* value among the replicates was calculated based on a volcano plot-based algorithm using Agilent GeneSpring GX software. The clusters of orthologous groups (COG) annotation classified all the genes into major functional and subfunctional categories (http://www.ncbi.nlm.nih.gov/COG). The Clusters of Orthologous Groups of proteins (COGs) database has been designed as an attempt to classify proteins from completely sequenced genomes based on the orthology concept. Heat maps were generated for the COG-based functionally classified differentially regulated genes using Agilent GeneSpring GX software.

### *In vivo* M-PFC assay.

*In vivo* interactions with PknK were investigated through mycobacterial protein fragment complementation assay (M-PFC assay) as described previously (48). The full-length M. tuberculosis *pknK* gene was cloned in pUAB300 and pUAB400 vectors to express recombinant PknK proteins tagged with F[1,2] and F[3] DHFR domains at its C terminus. The confirmed plasmids were sequence verified and electroporated in M. smegmatis. Similarly, putative target genes *Rv3246c* and *Rv0903c* were cloned in pUAB300 vectors.

A liquid format of the M-PFC assay described earlier (48) was standardized for the optimal concentration of trimethoprim. Primary cultures of M. smegmatis strains harboring different pairs of recombinant plasmids were subcultured twice in 7H9-ADS medium at 220 rpm at 37°C in the presence of kanamycin (25 μg/mL) and hygromycin (50 μg/mL). Two-fold serial dilution of trimethoprim was achieved using 7H9-ADS medium containing kanamycin and hygromycin and subsequently adding bacterial cultures of optical density value at 595 nm (OD_595_) of 0.05 in a 96-well clear-bottom black plate in triplicate. Plates were incubated for 24 h at 37°C before adding 30 μL of filter-sterilized 0.02% (wt/vol) Alamar Blue solution and 12.5 μL of 20% (vol/vol) Tween 80. Fluorescence intensity readings were taken at an excitation and emission wavelength of 530 and 590 nm, respectively, until the highest reading in any of the samples approached saturating levels of approximately 10,000 units.

### Cloning, expression, and purification of recombinant proteins.

The purified proteins for PrrA and MtrA RRs were obtained as previously described (49). The kinase domain (KD) region of the *pknK* gene encoding 1 to 300 amino acids was amplified using M. tuberculosis H37Rv genomic DNA as the template and cloned in plasmid pGEX 4T2 to yield recombinant plasmid pYA1554 expressing a glutathione *S*-transferase (GST)-tagged PknK KD. Recombinant proteins were purified using standard methods described in the supplemental material.

### *In vitro* kinase assay.

Purified PknK protein (150 picomoles) was incubated in PIPES [piperazine-*N*,*N*′-bis(2-ethanesulfonic acid)] buffer (100 mM PIPES, pH 7.0, 80 mM NaCl, and 20 mM MgCl_2_) containing 1 to 2 μCi of [γ-^32^P]ATP for 30 min at 25°C. Autophosphorylated PknK was mixed with PrrA and MtrA for *in vitro* substrate phosphorylation in the same buffer. The reaction mixture was incubated at 25°C for 45 min followed by addition of 5× SDS sample loading buffer and separation of proteins by SDS-PAGE. After electrophoresis, the gel was exposed to a phosphorimaging plate for 2 to 12 h and scanned with a Typhoon 9210 imager. The gel was then stained with Coomassie R_250_ to visualize the protein bands. Time kinetics assays were performed to check the intensity of phosphorylation of PrrA and MtrA through activated PknK. PrrA and MtrA were incubated with active PknK at 25°C for 0, 1, 2, 4, and 16 h. An i*n vitro* kinase assay was performed as described above, and relative phosphorylation activity as a function of time was calculated using Image J software.

### Electrophoretic mobility shift assay.

The ability of phosphorylated MtrA or PrrA (RR∼P) to bind *oriC* region or *prrA* promoter region DNA, respectively, was analyzed using the electrophoretic mobility shift assay (EMSA). The amplified DNA region was labeled by T4 polynucleotide kinase (Thermo Scientific) using 1 to 2 μCi of [γ-^32^P]ATP and purified using a GeneJET gel extraction kit. The RRs used for the EMSAs were phosphorylated (wherever indicated) by PknK and/or cognate SK with nonradioactive ATP for 1 h and further incubated for 1 h at 16°C with the radiolabeled probe, in binding buffer (25 mM Tris-HCl [pH 8.0], 6 mM MgCl_2_, 20 mM KCl, 0.10 mg/mL bovine serum albumin (BSA), 0.5% glycerol, 1 mM dithiothreitol (DTT), 0.5 mM EDTA, 2 mM ATP, and 1 μg poly(dI-dC)). The reaction mixtures were loaded onto a 5% native polyacrylamide gel (prerun for 2 to 3 h at 100 V), and samples were resolved at constant voltage, in 0.5× Tris-borate-EDTA buffer at 4°C. The gel was exposed to a phosphorimaging plate, and bands were visualized.

### Statistical analysis.

Statistical analyses were performed using Student’s *t* test or one-way analysis of variance (ANOVA) using GraphPad Prism 7.0 software for CFU determinations and qRT-PCR experiments. A *P* value of <0.05 was considered statistically significant. *, **, and *** indicate *P* values of <0.05, <0.01, and <0.001, respectively, wherever indicated.

### Data availability.

The microarray data presented in this study have been deposited in the NCBI Gene Expression Omnibus (GEO) under the GEO series accession number GSE180348.
