# Supplementary material for: Mycobacterium tuberculosis PknK Substrate Profiling Reveals Essential Transcription Terminator Protein Rho and Two-Component Response Regulators PrrA and MtrA as Novel Targets for Phosphorylation
Source: Microbiol Spectr. 2022 Apr 11;10(2):e01354-21. doi: 10.1128/spectrum.01354-21 (PMC9045387; doi:10.1128/spectrum.01354-21)
Supplement: SUPPLEMENTAL FILE 1 — Supplemental material. Download spectrum.01354-21-s0001.pdf, PDF file, 3.1 MB [file spectrum.01354-21-s0001.pdf]

Supplementary Methods.

2-Dimensional Differential Gel Electrophoresis (2D-DIGE).

2-D DIGE was performed by Applied Biomics, Inc (Hayward, CA). Briefly, the cell pellets were washed with Washing Buffer (10 mM Tris-HCl, 5 mM magnesium acetate, pH8.0) three times. 200 ul of 2-D cell lysis buffer (30 mM Tris-HCl, pH 8.8, containing 7 M urea, 2 M thiourea and 4 % CHAPS) was added. The mixture was sonicated at 4°C followed by shaking for 30 minutes at room temperature. The samples were then centrifuged for 30 minutes at 14,000 rpm and the supernatant was collected. Protein concentration was measured using Bio-Rad protein assay method. For CyDye labeling, 30 μg of protein of each sample was mixed with 1.0 μl of diluted CyDye and kept in dark on ice for 30 min. Samples were labeled with Cy2, Cy3 and Cy5 respectively. The labeling reaction was stopped by adding 1.0 μl of 10 mM Lysine to each sample and incubated in dark on ice for additional 15 min before mixing the samples together. IEF and SDS-PAGE was done on (pH 3-10 Linear) as per the manufacturer's instructions (Amersham BioSciences). Upon finishing the IEF, the IPG strips were incubated in the freshly made equilibration buffer-1 (50 mM Tris-HCl, pH 8.8, containing 6 M urea, 30% glycerol, 2% SDS, trace amount of bromophenol blue and 10 mg/ml DTT) for 15 minutes with gentle shaking. Then the strips were rinsed in the freshly made equilibration buffer-2 (50 mM Tris-HCl, pH 8.8, containing 6 M urea, 30% glycerol, 2% SDS, trace amount of bromophenol blue and 45 mg/ml DTT) for 10 minutes with gentle shaking. Next the IPG strips were rinsed in the SDS-gel running buffer before transferring into 12% SDS-gels. The SDS-gels were run at 15 ^{o}C until the dye front running out of the gels. Gel images were scanned immediately following the SDS-PAGE using Typhoon TRIO (GE Healthcare). The scanned images were then analyzed by Image Quant software (version 6.0, GE Healthcare), followed by in-gel analysis using DeCyder software (version 6.5, GE Healthcare). For Phospho-staining, the gel was then stained using Pro-Q ® Diamond Phosphoprotein Gel Stain following manufacturer's

protocol (Invitrogen), followed by scanning using Typhoon TRIO (GE Healthcare) and analysis using DeCyder software version 6.5 (GE Healthcare).

Protein identification by Mass Spectrometry.

The spots of interest were picked up by Ettan Spot Picker (Amersham BioSciences) based on the in-gel analysis and spot picking design by DeCyder software. The gel spots were washed a few times then digested in-gel with modified porcine trypsin protease (Trypsin Gold, Promega). The digested tryptic peptides were desalted by Zip-tip C18 (Millipore). Peptides were eluted from the Zip-tip with 0.5 μl of matrix solution (α-cyano-4-hydroxycinnamic acid (5 mg/ml in 50% acetonitrile, 0.1% trifluoroacetic acid, 25 mM ammonium bicarbonate) and spotted on the AB SCIEX MALDI plate (Opti-TOF™ 384 Well Insert). MALDI-TOF MS and TOF/TOF tandem MS/MS were performed on an AB SCIEX TOF/TOF™ 5800 System (AB SCIEX, Framingham, MA). MALDI-TOF mass spectra were acquired in reflectron positive ion mode, averaging 4000 laser shots per spectrum. TOF/TOF tandem MS fragmentation spectra were acquired for each sample, averaging 4000 laser shots per fragmentation spectrum on each of the 10 most abundant ions present in each sample (excluding trypsin autolytic peptides and other known background ions). Both of the resulting peptide mass and the associated fragmentation spectra were submitted to GPS Explorer workstation equipped with MASCOT search engine (Matrix science) to search the database of National Center for Biotechnology Information non-redundant (NCBInr). Searches were performed without constraining protein molecular weight or isoelectric point, with variable carbamidomethylation of cysteine and oxidation of methionine residues, and with one missed cleavage also allowed in the search parameters. Candidates with either protein score C.I.% or Ion C.I.% greater than 95 were considered significant.

Microarray Sample Labeling and Hybridization.

Microarrays were performed by Genotypic Technology Pvt Ltd, (Bangalore, India). The samples were labeled using Agilent Quick Amp Kit (Part number: 5190-0444). 500ng of total RNA was reverse transcribed using Random hexamer primer tagged to T7 promoter sequence. cDNA thus obtained was converted to double stranded cDNA in the same reaction. Further the cDNA was converted to cRNA in the in-vitro transcription step using T7 RNA polymerase enzyme and Cy3/Cy5 dye was added into the reaction mix. During cRNA synthesis Cy3/Cy5 dye was incorporated into the newly synthesized strands. cRNA obtained was cleaned up using Qiagen RNeasy columns (Qiagen, Cat No: 74106). Concentration and amount of dye incorporated was determined using Nanodrop. Labeled cRNA was cleaned up and quality assessed for yields and specific activity using Nanodrop.

The labeled cRNA samples were hybridized on to an Agilent Custom *M. smegmatis* 8x15k Gene expression designed by Genotypic Technology Pvt. Ltd. (AMADID: 043029). 1000 ng of Cy3 labeled samples and 1000 ng of Cy5 labeled samples were pooled. Fragmentation of labeled cRNA and hybridization were done using the Gene Expression Hybridization kit of Agilent (Part Number 5190-0404). Hybridization was carried out in Agilent's Surehyb Chambers at 65º C for 16 hours. The hybridized slides were washed using Agilent Gene Expression wash buffers (Part No: 5188-5327) and scanned using Agilent Scanner (Agilent Technologies, Part Number G2505C).

Feature Extraction and Microarray Data Analysis.

Data extraction from Images was done using Feature Extraction software Version 10.7 of Agilent. Feature extracted raw data was analyzed using Agilent GeneSpring GX Version 11.0 software. Normalization of the data was performed in GeneSpring GX using Lowess Normalization. In two-color experiments, where two fluorescent dyes (red and green) have

been used, intensity dependent variation in dye bias may introduce spurious variations in the collected data. Lowess normalization merges two-color data, applying a smoothing adjustment that removes such variation. Significant genes up regulated fold > 1.0 ($logbase_2$) and down regulated < -1.0 ($logbase_2$) in the test samples with respect to control sample were identified. A Dye-swap experiment was performed to nullify dye biasness. Statistical student T-test p-value among the replicates was calculated based on volcano plot algorithm. The clusters of orthologous groups (COG) annotation classified all the genes into major functional and sub-functional categories (http://www.ncbi.nlm.nih.gov/COG). The Clusters of Orthologous Groups of proteins (COGs) database has been designed as an attempt to classify proteins from completely sequenced genomes based on the orthology concept. Heat maps were generated for the COG based functionally classified differentially regulated genes using Agilent GeneSpring GX Software.

Protein Purification.

Primary culture inoculum for the recombinant plasmids was inoculated in 400 ml of 2XYT broth and grown at 37°C, 200 rpm till O.D 600 reached ~0.5-0.6. Then the culture was kept in 4°C for 30 min, followed by IPTG induction at final concentration of 0.1mM - 1mM and incubated for 16 h at 16°C on constant shaking. Cells were harvested by centrifugation at 3500 rpm for 10mins at 4°C. The pellet of PrrA and MtrA were resuspended in 10 ml of native lysis buffer (50 mM Tris-HCl, pH 7.4, 500 mM NaCl, 10 mM imidazole, 10% glycerol) containing 1 mM PMSF and 1 mM Benzamidine, sonicated on ice for 10 min at 50% amplitude (pulse on 2s, pulse off 1s). Then the slurry was centrifuged at 12000 rpm for 30 min at 4°C. The supernatant containing the protein was loaded into the Ni^{+2}-NTA column pre-equilibrated with native lysis buffer at 4°C. After 45 min of incubation with intermittent shaking, the unbound proteins were discarded as flow through followed by washing the column with wash buffer (50

mM Tris-HCl, pH 7.4, 500 mM NaCl, 20-40 mM imidazole, 10% glycerol). The protein was eluted by using the elution buffer (50mM Tris-HCl pH 7.4, 500 mM NaCl, 250 mM imidazole, 10% glycerol).

The cell pellet of PknK was resuspended in 10ml of lysis buffer (50 mM Tris-HCl, pH 8, 100 mM NaCl), containing 1mM PMSF and 1 mM Benzamidine, sonicated on ice for 10 min (pulse on 2s, pulse off 1s) at 50% amplitude. Then the sonicated solution was centrifuged at 12000 rpm for 30 min at 4°C. The supernatant containing the target protein was loaded into the Glutathione sepharose-4B column pre-equilibrated with lysis buffer at 4°C and incubated for overnight. The unbound proteins were discarded as flow through followed by washing the column with wash buffer (50 mM Tris-HCl, pH 8, 100 mM NaCl). Then the proteins were eluted by using the elution buffer (50 mM Tris-HCl, pH 8, 200 mM NaCl, 5 mM EDTA, 5 mM DTT, 10 mM reduced glutathione and 10% glycerol).

The eluted fractions of proteins were pooled together and dialyzed against the dialysis buffer I (50 mM Tris-HCl, pH 8.0, 50 mM NaCl, 0.1 mM DTT, 10% glycerol) for 6 h and the with dialysis buffer II or storage buffer (50 mM Tris-HCl, pH 8.0, 50 mM NaCl, 0.1 mM DTT, 50% glycerol) for 12 h. The protein concentration after dialysis was calculated by Bradford assay using BSA as standard and purity of the proteins were checked on SDS-PAGE. The purified proteins were stored at -20°C.

Figure S1

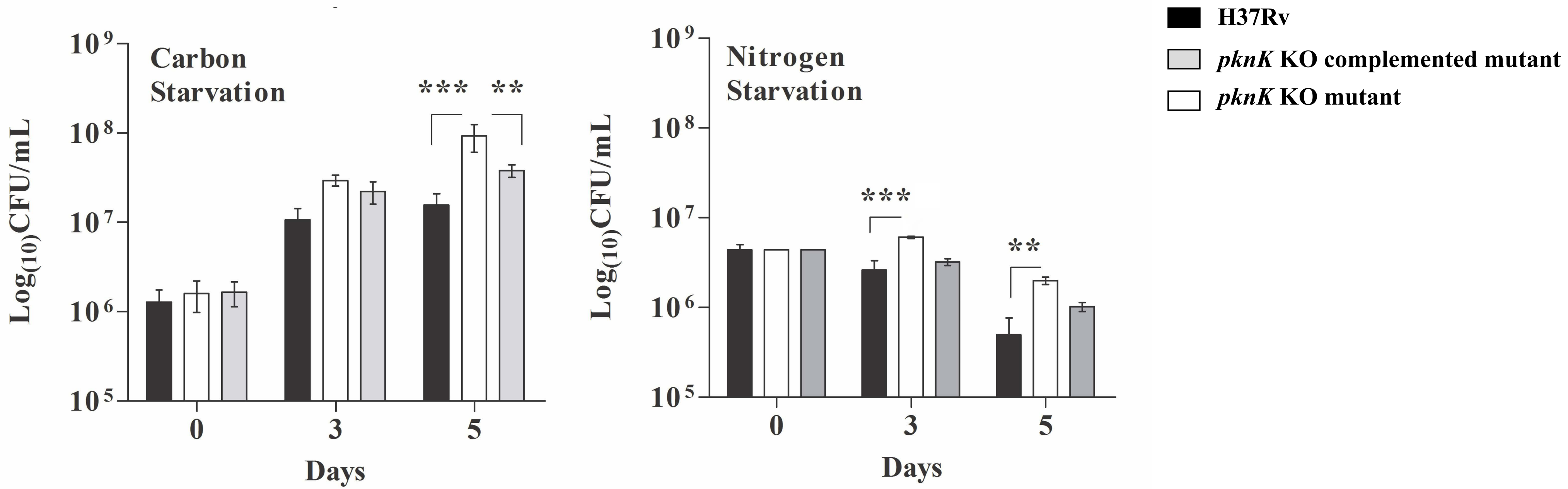

Figure S1. PknK facilitates survival under carbon and nitrogen starvation growth conditions.
The viable counts of *M. tuberculosis* H37Rv (solid black), PknK mutant (solid white) and PknK complemented mutant (solid gray) were determined at 0, 3 and 5 days post exposure to carbon or nitrogen stress. The average mean ± SD counts obtained from three independent experiments are shown. **, *** represent P<0.01 and P<0.001, respectively, for the differences in the growth of mutant compared to H37Rv or complemented mutant in carbon and nitrogen-limiting media at the indicated time points.

Figure S2

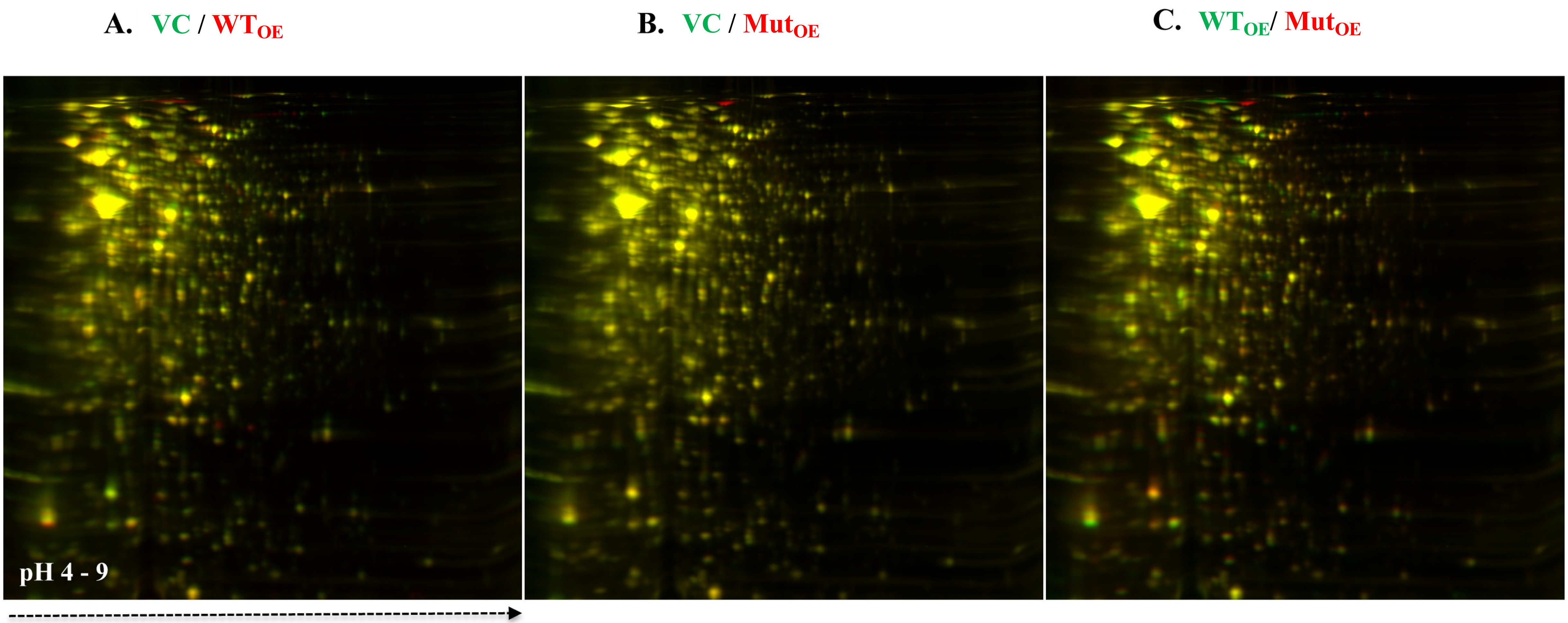

Figure S2. Differential protein profiles as a result of overexpression of wild type and Phosphorylation-defective PknK by 2D-DIGE
The cells were grown, induced with acetamide for 4 days and pelleted for 2D- DIGE analysis. The images were captured and overlayed to enhance the identification of differentially expressed proteins in the strains WT$_{OE}$ and Mut$_{OE}$.

VC = *M. smegmatis* with empty vector (pJFR19)

WT$_{OE}$= *M. smegmatis* with pJFR19-PknK$_{Mtb}$ (wild type PknK)

Mut$_{OE}$= *M. smegmatis* with pJFR19- K55M PknK$_{Mtb}$ (phosphorylation-defective PknK)

Figure S3

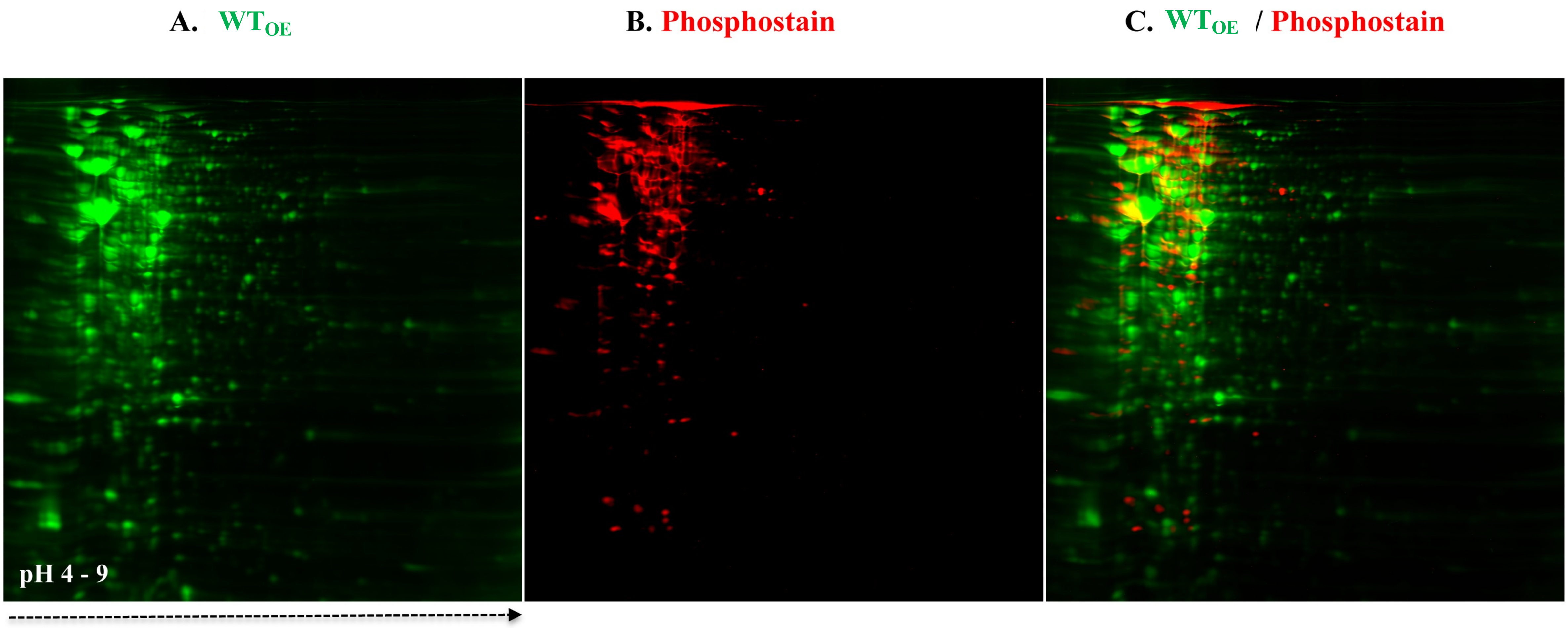

Figure S3. Phosphoprotein staining of 2D-DIGE profile of WT PknK overexpression strain

The 2D image of WT_{OE} sample was overlayed with a phosphostained gel image to mark the proteins that are phosphorylated by WT PknK. ProQ phosphoprotein stain was used to identify the phosphorylated proteins as per instructions of the manufacturer.

Table S1. Strains and Plasmids used in the study

**Strain/Plasmid**	**Description**	**Source/Reference**
M. tuberculosis H37Rv	Virulent laboratory strain	ATCC 25618
M. smegmatis mc^{2}155	*ept-1*	Dr. William R. Jacobs, Jr, Albert Einstein College of Medicine, NY, USA
E. coli JM109	e14- (McrA-940) *recA1 endA1 gyrA96 thi*-1 941 *hsdR17 supE44 relA1 (lac-proAB)*	Stratagene
E. coli XL1-Blue	*recA1 endA1 gyrA96 thi*-1 *hsdR17 supE44 relA1 lac* [F' *proAB lacIq ZΔM15* Tn*10* (*Tet*^{r})]	Stratagene
E. coli Arctic Express	B F$^-$ *ompT hsdS* (rB$^-$ mB$^-$) *dcm*$^+$ *Tet*^{r} *gal endA* Hte [*cpn10 cpn60 Gent*^{r}]	Agilent
pknK KO (LIX11)	H37Rv Δ*pknK*	(1)
pknK KO complement (LIX16)	H37Rv Δ*pknK::*pMV306-*pknK*	(1)
VC (LIX70)	mc^{2}155::pJFR19	(2)
WT_{OE} (LIX79)	mc^{2}155::pYA1662	(2)
Mut_{OE} (LIX80)	mc^{2}155::pYA1671	(2)
**Plasmids**		
pUAB100	Hyg^r, *oriM oriE hsp60*-GCN4-Gly_{10} -mDHFR [F1,2]	(3)
pUAB200	Kan^r, *attP int hsp60*-GCN4-Gly_{10} -mDHFR [F3]	(3)
pUAB300	Hyg^r, *oriM oriE hsp60*-mDHFR[F1,2]-Gly_{10} -MCS	(3)
pUAB400	Kan^r, *attP int hsp60*-mDHFR [F3]-Gly_{10}-MCS	(3)
pPROEx-PrrA	*prrA* cloned in pProEx-HTa	(4)
pPROEx-MtrA	*mtrA* cloned in pProEx-HTb	(4)
pYA1554	*pknK* $_{(1\text{-}900\ bp)}$ in *Bam*HI/*Not*I of pGEX-4T2	This study
pUAB300-PknK	pUAB300::*pknK* in *Hind*III site	This study
pUAB400-PknK	pUAB400::*pknK* in *Hind*III site	This study
pUAB300-PrrA	pUAB300::*prrA* in *Bam*HI and *Hind*III sites	This study
pUAB300-MtrA	pUAB300::*mtrA* in *Bam*HI and *Hind*III sites	This study

References.

1. Malhotra, V., Arteaga-Cortes, L. T., Clay, G., and Clark-Curtiss, J. E. (2010) Mycobacterium tuberculosis protein kinase K confers survival advantage during early infection in mice and regulates growth in culture and during persistent infection: implications for immune modulation. *Microbiology (Reading)* **156**, 2829-2841
2. Malhotra, V., Okon, B. P., and Clark-Curtiss, J. E. (2012) Mycobacterium tuberculosis protein kinase K enables growth adaptation through translation control. *J Bacteriol* **194**, 4184-4196
3. Singh, A., Mai, D., Kumar, A., and Steyn, A. J. (2006) Dissecting virulence pathways of Mycobacterium tuberculosis through protein-protein association. *Proc Natl Acad Sci U S A* **103**, 11346-11351
4. Agrawal, R., Pandey, A., Rajankar, M. P., Dixit, N. M., and Saini, D. K. (2015) The two-component signalling networks of Mycobacterium tuberculosis display extensive cross-talk in vitro. *Biochem J* **469**, 121-134

Table S2: Protein Expression Ratios and Phosphostaining status of DIGE spots

Protein Ratio
Cut off : 1.5 fold
VC= Vector Control **WT_{OE}= WT PknK OE** **Mut_{OE}=K55M PknK OE**

		Protein Expression Ratio by 2D-DIGE		
**Spot No.**	**Original No.**	**WT_{OE} / VC**	**Mut_{OE} / VC**	**Positive for Phosphostaining**
1	111	3.0	1.0	x
2	121	1.5	27.2	
3	99	1.2	1.6	
4	80	2.4	-1.0	
5	199	-1.6	-1.1	
6	196	30.0	1.1	x
7	195	20.2	-1.1	x
8	186	5.2	-1.2	x
9	188	12.8	-1.1	
10	189	-3.1	-1.0	
11	191	-4.5	1.2	
12	240	-1.7	-1.0	
13	285	-1.6	-1.0	
14	221	1.8	-1.3	x
15	292	1.9	-1.1	x
16	342	1.6	-1.7	x
17	348	-1.6	-1.1	
18	349	-1.5	1.1	
19	309	-1.4	1.2	
20	359	1.5	-1.1	x
21	398	-1.8	1.0	x
22	508	1.9	-1.2	x
23	418	-1.2	1.5	
24	430	-1.4	1.1	
25	461	-1.4	1.1	
26	462	-1.5	1.1	
27	440	3.4	-1.2	
28	434	-1.6	1.1	
29	542	-1.6	-1.1	x
30	567	-1.2	1.5	x
31	518	1.2	1.7	
32	574	-1.4	1.1	
33	576	-1.5	1.2	x
34	555	-1.5	-1.0	
35	678	-2.2	1.0	
36	671	2.3	1.0	x
37	724	-1.4	1.1	x
38	670	-1.9	1.0	x
39	679	-1.7	1.1	
40	697	1.7	1.1	
41	662	1.5	-1.1	
42	749	-2.1	-1.2	x
43	812	-1.6	-1.1	
44	803	1.7	-1.2	x
45	824	-1.8	1.0	
46	853	-2.2	1.1	
47	926	1.5	-1.0	x
48	980	1.3	-1.3	x
49	1075	-1.5	-1.0	
50	1085	-1.5	1.0	
51	1103	-1.2	1.3	
52	1214	-2.3	-1.1	
53	1253	1.8	1.0	x
54	1261	-2.0	-1.1	
55	1264	-2.3	-1.0	
56	1286	-2.0	-1.4	
57	1359	1.6	-1.0	
58	1460	1.9	1.4	
59	1489	-1.6	1.1	
60	1517	1.5	1.1	
61	1636	-2.4	-1.2	
62	1667	-1.4	1.1	
63	1612	-1.3	1.2	
64	1638	-1.4	1.1	
65	1605	-1.8	-1.1	
66	1635	6.6	-1.1	
67	1695	-1.6	1.1	
68	1721	-1.7	1.1	
69	1785	4.1	-1.4	x
70	1789	6.9	-1.0	
71	1972	-1.6	1.0	
72	1996	-1.7	1.2	
73	2053	-1.4	1.1	
74	2103	-1.5	1.2	
75	2115	-2.3	1.0	
76	2173	1.6	-1.0	
77	2243	-1.6	1.3	

Table S3. Mass SpectrometryAnalysis

Spot number	MALDI well number	Match Quality	Top Ranked Protein Name (Species)	Accession No.	Protein MW	Protein PI	Pep.Count	Protein Score	Protein Score C. I. %	Total Ion Score	Total Ion C. I. %
1	B5		serine/threonine-protein kinase transcriptional regulator [Mycobacterium bovis AF2122/97]	gi|31794259	119,373	5.5	52	1,050	100	850	100
2	B6		serine/threonine-protein kinase transcriptional regulator [Mycobacterium bovis AF2122/97]	gi|31794259	119,373	5.5	48	753	100	612	100
6	B7		transcription termination factor Rho [Mycobacterium smegmatis str. MC2 155]	gi|118471197	71,705	6.7	24	158	100		
7	B8		transcription termination factor Rho [Mycobacterium smegmatis str. MC2 155]	gi|118471197	71,705	6.7	26	327	100	238	100
36	B9		histidine ammonia-lyase [Mycobacterium smegmatis str. MC2 155]	gi|118471752	53,355	5.9	21	778	100	746	100
69	B10		hypothetical protein MSMEG_5010 [Mycobacterium smegmatis str. MC2 155]	gi|118469659	19,503	6.0	16	514	100	425	100

Control

1 fmol	B11		Beta-galactosidase OS=Escherichia coli O127:H6 (strain E2348/69 / EPEC) GN=lacZ PE=3 SV=1	BGAL_ECO27	116,343	5.2	22	418	100	315	100
2 fmol	B12		Beta-galactosidase OS=Escherichia coli O127:H6 (strain E2348/69 / EPEC) GN=lacZ PE=3 SV=1	BGAL_ECO27	116,343	5.2	22	451	100	349	100
5 fmol	B13		Beta-galactosidase OS=Escherichia coli (strain K12) GN=lacZ PE=1 SV=2	BGAL_ECOLI	116,409	5.3	25	510	100	379	100
10 fmol	B14		Beta-galactosidase OS=Escherichia coli (strain K12) GN=lacZ PE=1 SV=2	BGAL_ECOLI	116,409	5.3	23	466	100	355	100

high confidence
low confidence
no confidence

2012_11

MARA121105

Analysis Information

Report Type	Protein-Peptide Summary by Spot	Analysis Type	Combined (MS+MS/MS)
Sample Set Name	MARA121105	Database	NCBInr
Analysis Name	Mycobaterium smegmatis_NCBI_1P	Creation Date	
Reported By		Last Modified	
MS Acq. : Proc. Methods	(Unspecified) : (Unspecified)		
Interpretation Method	(Unspecified)		

Gel Idx/Pos	32/B5	Instr./Gel Origin	BA2060/121107A	Process Status	Analysis Succeeded
Plate [#] Name	[1] 31598	Instrument Sample Name		Spectra	8

Rank	Protein Name	Accession No.	Protein MW	Protein PI	Pep. Count	Protein Score	Protein Score C. I. %	Total Ion Score	Total Ion C. I. %
1	serine/threonine-protein kinase transcriptional regulator [Mycobacterium bovis AF2122/97]	gi|31794259	119372.9	5.52	52	1,050	100	850	100

Peptide Information

Calc. Mass	Obsrv. Mass	± da	± ppm	Start Seq.	End Seq.	Sequence	Ion Score	C. I. %	Modification	Rank	Result Type
846.5195	846.5076	-0.0119	-14	232	238	VIAQFLR					Mascot
854.4518	854.4397	-0.0121	-14	35	42	GGFGVVYR					Mascot
880.4424	880.4305	-0.0119	-14	118	124	HGPLDWR					Mascot
932.4948	932.4824	-0.0124	-13	375	382	STLAAQWR					Mascot
945.5363	945.522	-0.0143	-15	109	116	NSLETLIR					Mascot
977.505	977.4896	-0.0154	-16	792	800	AVAEVFADR					Mascot
987.5217	987.506	-0.0157	-16	564	573	GGGDATQLLR					Mascot
1008.5584	1008.5435	-0.0149	-15	133	142	LAGALEAAHR					Mascot
1036.5436	1036.5271	-0.0165	-16	117	124	RHGPLDWR					Mascot
1085.5044	1085.49	-0.0144	-13	627	635	AMLEEAEHR					Mascot
1139.642	1139.624	-0.018	-16	239	248	ITSQPIPDLR					Mascot
1179.5066	1179.4906	-0.016	-14	642	650	TEDDPNWFR					Mascot
1179.5066	1179.4906	-0.016	-14	642	650	TEDDPNWFR	50	8.026			Mascot
1223.6532	1223.6353	-0.0179	-15	456	465	IAVVIDDWHR					Mascot
1252.7412	1252.7192	-0.022	-18	363	374	LILIHAPSGFGK					Mascot
1259.7106	1259.6931	-0.0175	-14	877	887	LDIVAALQNFR					Mascot
	1259.6931										Mascot
1263.6943	1263.6771	-0.0172	-14	600	610	EFLLVASVTER					Mascot
1263.6943	1263.6771	-0.0172	-14	600	610	EFLLVASVTER	61	93.548			Mascot
1267.7368	1267.7136	-0.0232	-18	239	249	ITSQPIPDLRK					Mascot
1293.5925	1293.658	0.0655	51	721	730	MTTLLAIVQK			Oxidation (M)[1], Phospho (ST)[2,3]		Mascot
1310.7063	1310.6835	-0.0228	-17	250	262	QGLPADVAAAIER					Mascot
1313.6696	1313.6511	-0.0185	-14	773	784	AELPEATQADLR					Mascot
1361.7172	1361.6919	-0.0253	-19	792	803	AVAEVFADRVER					Mascot
1438.8013	1438.7759	-0.0254	-18	249	262	KQGLPADVAAAIER					Mascot
1448.6892	1448.6661	-0.0231	-16	651	661	FHQMFADFLHR					Mascot
1448.6892	1448.6661	-0.0231	-16	651	661	FHQMFADFLHR	81	99.94			Mascot
1464.6841	1464.658	-0.0261	-18	651	661	FHQMFADFLHR			Oxidation (M)[4]		Mascot
1472.759	1472.7329	-0.0261	-18	505	518	IGDELAEIDSAALR					Mascot
1484.8068	1484.781	-0.0258	-17	967	981	LSTGGDTAVQLGLPR					Mascot
1512.757	1512.7281	-0.0289	-19	336	348	YRPSVPTGSLVTR			Phospho (Y)[1]		Mascot
1518.7693	1518.7423	-0.027	-18	611	626	TCGGLASALAGITNGR			Carbamidomethyl (C)[2]		Mascot
1529.8546	1529.8271	-0.0275	-18	875	887	NRLDIVAALQNFR					Mascot
1542.8057	1542.7792	-0.0265	-17	822	837	VPGTAGNTAALAAICR			Carbamidomethyl (C)[15]		Mascot
1545.7867	1545.764	-0.0227	-15	56	68	VLSTDLDRDNLER					Mascot
1622.772	1622.9424	0.1704	105	822	837	VPGTAGNTAALAAICR			Carbamidomethyl (C)[15], Phospho (ST)[4]		Mascot
1631.9843	1631.9554	-0.0289	-18	993	1009	LGIALPAAVAADLLAPR					Mascot
1631.9843	1631.9554	-0.0289	-18	993	1009	LGIALPAAVAADLLAPR	157	100			Mascot
1681.8405	1681.8092	-0.0313	-19	888	904	TAFEVGTAVGAHSHAAR					Mascot
1748.845	1748.8118	-0.0332	-19	519	535	FDTDEAAALLNDAGGLR					Mascot
1762.8137	1762.7773	-0.0364	-21	1032	1047	LLSAGDSADRDQACQR			Carbamidomethyl (C)[14]		Mascot
1804.8937	1804.8574	-0.0363	-20	267	283	HPADRPATAADVGEELR					Mascot
1828.8113	1828.7734	-0.0379	-21	519	535	FDTDEAAALLNDAGGLR			Phospho (ST)[3]		Mascot
1846.9293	1846.8922	-0.0371	-20	539	556	ADVQALTTSTDGWAAAL	157	100			Mascot
						R					
1846.9293	1846.8922	-0.0371	-20	539	556	ADVQALTTSTDGWAAAL					Mascot
						R					
1907.8651	1907.8225	-0.0426	-22	1014	1031	DNGIATMTAELDEDSAVR					Mascot
1914.9291	1914.8871	-0.042	-22	704	720	AVDLVEQDETNLPEQSK					Mascot
1931.8698	1931.8961	0.0263	14	1086	1102	NELAPVATKCAELGLSR			Phospho (ST)[16], Phospho (ST)[8]		Mascot
1939.9794	1939.9386	-0.0408	-21	289	306	NGVSVDEMPLPVELGVE					Mascot
						R					
1955.9742	1955.9326	-0.0416	-21	289	306	NGVSVDEMPLPVELGVE			Oxidation (M)[8]		Mascot
						R					
1956.7905	1956.9248	0.1343	69	318	335	HTGGGTPTVPTPPTPATK			Phospho (ST)[2,6,8]		Mascot
1995.9663	1995.9735	0.0072	4	125	142	ETLSIGVKLAGALEAAHR			Phospho (ST)[4], Phospho (ST)[2]		Mascot
2012.0117	2011.967	-0.0447	-22	804	821	VDDLLAEAMSRPDTLPP			Oxidation (M)[9]		Mascot
						R					
2090.1128	2090.0718	-0.041	-20	905	925	LAGSLLAELLYETGDLAG					Mascot
						AGR					
2230.0696	2230.0222	-0.0474	-21	926	946	LMDESYLLGSEGGAVDY					Mascot
						LAAR					
2230.0696	2230.0222	-0.0474	-21	926	946	LMDESYLLGSEGGAVDY	161	100			Mascot
						LAAR					
2243.2983	2243.2505	-0.0478	-21	1060	1080	RPLAALQAQILHIETLAAT					Mascot
						GR					
2246.0645	2246.0164	-0.0481	-21	926	946	LMDESYLLGSEGGAVDY			Oxidation (M)[2]		Mascot
						LAAR					
2303.1487	2303.1006	-0.0481	-21	267	287	HPADRPATAADVGEELR					Mascot
						DVQR					
2358.1797	2358.1289	-0.0508	-22	418	439	VRPTLAESLGHVLEEHG					Mascot
						DDAGR					
2510.2773	2510.2209	-0.0564	-22	12	34	DLVPNIPAELLEAGFDNV					Mascot
						EEIGR					
2632.3616	2632.3037	-0.0579	-22	149	172	DVKPGNILLTDYGEPQLT	184	100			Mascot
						DFGIAR					
2632.3616	2632.3037	-0.0579	-22	149	172	DVKPGNILLTDYGEPQLT					Mascot
						DFGIAR					
2739.291	2739.2271	-0.0639	-23	678	703	ASAWFAENGYLHEAVDH					Mascot
						ALAAGDPAR					
2840.4312	2840.3694	-0.0618	-22	574	599	GLSGASDVIHEFLSENVL					Mascot
						DTLEPELR					

Rank	Protein Name	Accession No.	Protein MW	Protein PI	Pep. Count	Protein Score	Protein Score C. I. %	Total Ion Score	Total Ion C. I. %
2	conserved hypothetical protein [Mycobacterium tuberculosis 94_M4241A]	gi|298526552	119372.9	5.52	52	1,050	100	850	100

Peptide Information

Calc. Mass	Obsrv. Mass	± da	± ppm	Start Seq.	End Seq.	Sequence	Ion Score	C. I. %	Modification	Rank	Result Type
846.5195	846.5076	-0.0119	-14	232	238	VIAQFLR					Mascot
854.4518	854.4397	-0.0121	-14	35	42	GGFGVVYR					Mascot
880.4424	880.4305	-0.0119	-14	118	124	HGPLDWR					Mascot
932.4948	932.4824	-0.0124	-13	375	382	STLAAQWR					Mascot
945.5363	945.522	-0.0143	-15	109	116	NSLETLIR					Mascot
977.505	977.4896	-0.0154	-16	792	800	AVAEVFADR					Mascot
987.5217	987.506	-0.0157	-16	564	573	GGGDATQLLR					Mascot
1008.5584	1008.5435	-0.0149	-15	133	142	LAGALEAAHR					Mascot
1036.5436	1036.5271	-0.0165	-16	117	124	RHGPLDWR					Mascot
1085.5061	1085.49	-0.0161	-15	731	739	LPTSMVVSR			Oxidation (M)[5], Phospho (ST)[3]		Mascot

	1139.642	1139.624	-0.018	-16	239	248	ITSQPIPDLR									Mascot
	1179.5066	1179.4906	-0.016	-14	642	650	TEDDPNWFR									Mascot
	1179.5066	1179.4906	-0.016	-14	642	650	TEDDPNWFR			50	8.026					Mascot
	1223.6532	1223.6353	-0.0179	-15	456	465	IAVVIDDWHR									Mascot
	1252.7412	1252.7192	-0.022	-18	363	374	LILIHAPSGFGK									Mascot
	1259.7106	1259.6931	-0.0175	-14	877	887	LDIVAALQNFR									Mascot
		1259.6931														Mascot
	1263.6943	1263.6771	-0.0172	-14	600	610	EFLLVASVTER									Mascot
	1263.6943	1263.6771	-0.0172	-14	600	610	EFLLVASVTER			61	93.548					Mascot
	1267.7368	1267.7136	-0.0232	-18	239	249	ITSQPIPDLRK									Mascot
	1293.5925	1293.658	0.0655	51	721	730	MTTLLAIVQK					Oxidation (M)[1], Phospho (ST)[2,3]				Mascot
	1310.7063	1310.6835	-0.0228	-17	250	262	QGLPADVAAAIER									Mascot
	1313.6696	1313.6511	-0.0185	-14	773	784	AELPEATQADLR									Mascot
	1361.7172	1361.6919	-0.0253	-19	792	803	AVAEVFADRVER									Mascot
	1438.8013	1438.7759	-0.0254	-18	249	262	KQGLPADVAAAIER									Mascot
	1448.6892	1448.6661	-0.0231	-16	651	661	FHQMFADFLHR									Mascot
	1448.6892	1448.6661	-0.0231	-16	651	661	FHQMFADFLHR			81	99.94					Mascot
	1464.6841	1464.658	-0.0261	-18	651	661	FHQMFADFLHR					Oxidation (M)[4]				Mascot
	1472.759	1472.7329	-0.0261	-18	505	518	IGDELAEIDSAALR									Mascot
	1484.8068	1484.781	-0.0258	-17	967	981	LSTGGDTAVQLGLPR									Mascot
	1512.757	1512.7281	-0.0289	-19	336	348	YRPSVPTGSLVTR					Phospho (Y)[1]				Mascot
	1518.7693	1518.7423	-0.027	-18	611	626	TCGGLASALAGITNGR					Carbamidomethyl (C)[2]				Mascot
	1529.8546	1529.8271	-0.0275	-18	875	887	NRLDIVAALQNFR									Mascot
	1542.8057	1542.7792	-0.0265	-17	822	837	VPGTAGNTAALAAICR					Carbamidomethyl (C)[15]				Mascot
	1545.7867	1545.764	-0.0227	-15	56	68	VLSTDLDRDNLER									Mascot
	1622.772	1622.9424	0.1704	105	822	837	VPGTAGNTAALAAICR					Carbamidomethyl (C)[15], Phospho (ST)[4]				Mascot
	1631.9843	1631.9554	-0.0289	-18	993	1009	LGIALPAAVAADLLAPR									Mascot
	1631.9843	1631.9554	-0.0289	-18	993	1009	LGIALPAAVAADLLAPR			157	100					Mascot
	1681.8405	1681.8092	-0.0313	-19	888	904	TAFEVGTAVGAHSHAAR									Mascot
	1748.845	1748.8118	-0.0332	-19	519	535	FDTDEAAALLNDAGGLR									Mascot
	1762.8137	1762.7773	-0.0364	-21	1032	1047	LLSAGDSADRDQACQR					Carbamidomethyl (C)[14]				Mascot
	1804.8937	1804.8574	-0.0363	-20	267	283	HPADRPATAADVGEELR									Mascot
	1828.8113	1828.7734	-0.0379	-21	519	535	FDTDEAAALLNDAGGLR					Phospho (ST)[3]				Mascot
	1846.9293	1846.8922	-0.0371	-20	539	556	ADVQALTTSTDGWAAAL			157	100					Mascot
							R									
	1846.9293	1846.8922	-0.0371	-20	539	556	ADVQALTTSTDGWAAAL									Mascot
							R									
	1907.8651	1907.8225	-0.0426	-22	1014	1031	DNGIATMTAELDEDSAVR									Mascot
	1914.9291	1914.8871	-0.042	-22	704	720	AVDLVEQDETNLPEQSK									Mascot
	1931.8698	1931.8961	0.0263	14	1086	1102	NELAPVATKCAELGLSR					Phospho (ST)[16], Phospho (ST)[8]				Mascot
	1939.9794	1939.9386	-0.0408	-21	289	306	NGVSVDEMPLPVELGVE									Mascot
							R									
	1955.9742	1955.9326	-0.0416	-21	289	306	NGVSVDEMPLPVELGVE					Oxidation (M)[8]				Mascot
							R									
	1956.7905	1956.9248	0.1343	69	318	335	HTGGGTPTVPTPPTPATK					Phospho (ST)[2,6,8]				Mascot
	1995.9663	1995.9735	0.0072	4	125	142	ETLSIGVKLAGALEAAHR					Phospho (ST)[4], Phospho (ST)[2]				Mascot
	2012.0117	2011.967	-0.0447	-22	804	821	VDDLLAEAMSRPDTLPP					Oxidation (M)[9]				Mascot
							R									
	2090.1128	2090.0718	-0.041	-20	905	925	LAGSLLAELLYETGDLAG									Mascot
							AGR									
	2230.0696	2230.0222	-0.0474	-21	926	946	LMDESYLLGSEGGAVDY									Mascot
							LAAR									
	2230.0696	2230.0222	-0.0474	-21	926	946	LMDESYLLGSEGGAVDY			161	100					Mascot
							LAAR									
	2243.2983	2243.2505	-0.0478	-21	1060	1080	RPLAALQAQILHIETLAAT									Mascot
							GR									
	2246.0645	2246.0164	-0.0481	-21	926	946	LMDESYLLGSEGGAVDY					Oxidation (M)[2]				Mascot
							LAAR									
	2303.1487	2303.1006	-0.0481	-21	267	287	HPADRPATAADVGEELR									Mascot
							DVQR									
	2358.1797	2358.1289	-0.0508	-22	418	439	VRPTLAESLGHVLEEHG									Mascot
							DDAGR									
	2510.2773	2510.2209	-0.0564	-22	12	34	DLVPNIPAELLEAGFDNV									Mascot
							EEIGR									
	2632.3616	2632.3037	-0.0579	-22	149	172	DVKPGNILLTDYGEPQLT			184	100					Mascot
							DFGIAR									
	2632.3616	2632.3037	-0.0579	-22	149	172	DVKPGNILLTDYGEPQLT									Mascot
							DFGIAR									
	2739.291	2739.2271	-0.0639	-23	678	703	ASAWFAENGYLHEAVDH									Mascot
							ALAAGDPAR									
	2840.4312	2840.3694	-0.0618	-22	574	599	GLSGASDVIHEFLSENVL									Mascot
							DTLEPELR									
3	serine/threonine protein kinase K [Mycobacterium bovis					gi|154795739		119415.9	5.52	49	1,020	100	850	100		
	BCG str. Pasteur 1173P2]															
	**Peptide Information**															
	**Calc. Mass**	**Obsrv. Mass**	**± da**	**± ppm**	**Start**	**End**	**Sequence**			**Ion**	**C. I. %**	**Modification**			**Rank**	**Result Type**
					**Seq.**	**Seq.**				**Score**						
	846.5195	846.5076	-0.0119	-14	232	238	VIAQFLR									Mascot
	854.4518	854.4397	-0.0121	-14	35	42	GGFGVVYR									Mascot
	880.4424	880.4305	-0.0119	-14	118	124	HGPLDWR									Mascot
	932.4948	932.4824	-0.0124	-13	375	382	STLAAQWR									Mascot
	945.5363	945.522	-0.0143	-15	109	116	NSLETLIR									Mascot
	977.505	977.4896	-0.0154	-16	792	800	AVAEVFADR									Mascot
	987.5217	987.506	-0.0157	-16	564	573	GGGDATQLLR									Mascot
	1008.5584	1008.5435	-0.0149	-15	133	142	LAGALEAAHR									Mascot
	1036.5436	1036.5271	-0.0165	-16	117	124	RHGPLDWR									Mascot
	1085.5044	1085.49	-0.0144	-13	627	635	AMLEEAEHR									Mascot
	1139.642	1139.624	-0.018	-16	239	248	ITSQPIPDLR									Mascot
	1179.5066	1179.4906	-0.016	-14	642	650	TEDDPNWFR									Mascot
	1179.5066	1179.4906	-0.016	-14	642	650	TEDDPNWFR			50	8.026					Mascot
	1223.6532	1223.6353	-0.0179	-15	456	465	IAVVIDDWHR									Mascot
	1252.7412	1252.7192	-0.022	-18	363	374	LILIHAPSGFGK									Mascot
	1259.7106	1259.6931	-0.0175	-14	877	887	LDIVAALQNFR									Mascot
		1259.6931														Mascot
	1263.6943	1263.6771	-0.0172	-14	600	610	EFLLVASVTER									Mascot
	1263.6943	1263.6771	-0.0172	-14	600	610	EFLLVASVTER			61	93.548					Mascot
	1267.7368	1267.7136	-0.0232	-18	239	249	ITSQPIPDLRK									Mascot
	1293.5925	1293.658	0.0655	51	721	730	MTTLLAIVQK					Oxidation (M)[1], Phospho (ST)[2,3]				Mascot
	1313.6696	1313.6511	-0.0185	-14	773	784	AELPEATQADLR									Mascot
	1361.7172	1361.6919	-0.0253	-19	792	803	AVAEVFADRVER									Mascot
	1448.6892	1448.6661	-0.0231	-16	651	661	FHQMFADFLHR									Mascot
	1448.6892	1448.6661	-0.0231	-16	651	661	FHQMFADFLHR			81	99.94					Mascot
	1464.6841	1464.658	-0.0261	-18	651	661	FHQMFADFLHR					Oxidation (M)[4]				Mascot
	1472.759	1472.7329	-0.0261	-18	505	518	IGDELAEIDSAALR									Mascot
	1484.8068	1484.781	-0.0258	-17	967	981	LSTGGDTAVQLGLPR									Mascot
	1518.7693	1518.7423	-0.027	-18	611	626	TCGGLASALAGITNGR					Carbamidomethyl (C)[2]				Mascot
	1529.8546	1529.8271	-0.0275	-18	875	887	NRLDIVAALQNFR									Mascot
	1542.8057	1542.7792	-0.0265	-17	822	837	VPGTAGNTAALAAICR					Carbamidomethyl (C)[15]				Mascot
	1545.7867	1545.764	-0.0227	-15	56	68	VLSTDLDRDNLER									Mascot
	1622.772	1622.9424	0.1704	105	822	837	VPGTAGNTAALAAICR					Carbamidomethyl (C)[15], Phospho (ST)[4]				Mascot
	1631.9843	1631.9554	-0.0289	-18	993	1009	LGIALPAAVAADLLAPR									Mascot
	1631.9843	1631.9554	-0.0289	-18	993	1009	LGIALPAAVAADLLAPR			157	100					Mascot
	1681.8405	1681.8092	-0.0313	-19	888	904	TAFEVGTAVGAHSHAAR									Mascot
	1748.845	1748.8118	-0.0332	-19	519	535	FDTDEAAALLNDAGGLR									Mascot
	1762.8137	1762.7773	-0.0364	-21	1032	1047	LLSAGDSADRDQACQR					Carbamidomethyl (C)[14]				Mascot
	1804.8937	1804.8574	-0.0363	-20	267	283	HPADRPATAADVGEELR									Mascot
	1828.8113	1828.7734	-0.0379	-21	519	535	FDTDEAAALLNDAGGLR					Phospho (ST)[3]				Mascot
	1846.9293	1846.8922	-0.0371	-20	539	556	ADVQALTTSTDGWAAAL			157	100					Mascot
							R									
	1846.9293	1846.8922	-0.0371	-20	539	556	ADVQALTTSTDGWAAAL									Mascot

							R									
	1907.8651	1907.8225	-0.0426	-22	1014	1031	DNGIATMTAELDEDSAVR									Mascot
	1914.9291	1914.8871	-0.042	-22	704	720	AVDLVEQDETNLPEQSK									Mascot
	1931.8698	1931.8961	0.0263	14	1086	1102	NELAPVATKCAELGLSR					Phospho (ST)[16], Phospho (ST)[8]				Mascot
	1939.9794	1939.9386	-0.0408	-21	289	306	NGVSVDEMPLPVELGVE									Mascot
							R									
	1955.9742	1955.9326	-0.0416	-21	289	306	NGVSVDEMPLPVELGVE					Oxidation (M)[8]				Mascot
							R									
	1956.7905	1956.9248	0.1343	69	318	335	HTGGGTPTVPTPPTPATK					Phospho (ST)[2,6,8]				Mascot
	1995.9663	1995.9735	0.0072	4	125	142	ETLSIGVKLAGALEAAHR					Phospho (ST)[4], Phospho (ST)[2]				Mascot
	2012.0117	2011.967	-0.0447	-22	804	821	VDDLLAEAMSRPDTLPP					Oxidation (M)[9]				Mascot
							R									
	2090.1128	2090.0718	-0.041	-20	905	925	LAGSLLAELLYETGDLAG									Mascot
							AGR									
	2230.0696	2230.0222	-0.0474	-21	926	946	LMDESYLLGSEGGAVDY									Mascot
		2230.0222														Mascot
							LAAR									
	2230.0696	2230.0222	-0.0474	-21	926	946	LMDESYLLGSEGGAVDY			161	100					Mascot
							LAAR									
	2243.2983	2243.2505	-0.0478	-21	1060	1080	RPLAALQAQILHIETLAAT									Mascot
							GR									
	2246.0645	2246.0164	-0.0481	-21	926	946	LMDESYLLGSEGGAVDY					Oxidation (M)[2]				Mascot
							LAAR									
	2303.1487	2303.1006	-0.0481	-21	267	287	HPADRPATAADVGEELR									Mascot
							DVQR									
	2358.1797	2358.1289	-0.0508	-22	418	439	VRPTLAESLGHVLEEHG									Mascot
							DDAGR									
	2510.2773	2510.2209	-0.0564	-22	12	34	DLVPNIPAELLEAGFDNV									Mascot
							EEIGR									
	2632.3616	2632.3037	-0.0579	-22	149	172	DVKPGNILLTDYGEPQLT			184	100					Mascot
							DFGIAR									
	2632.3616	2632.3037	-0.0579	-22	149	172	DVKPGNILLTDYGEPQLT									Mascot
							DFGIAR									
	2739.291	2739.2271	-0.0639	-23	678	703	ASAWFAENGYLHEAVDH									Mascot
							ALAAGDPAR									
	2840.4312	2840.3694	-0.0618	-22	574	599	GLSGASDVIHEFLSENVL									Mascot
							DTLEPELR									
**Gel Idx/Pos**		33/B6					**Instr./Gel Origin**			BA2060/121107A				**Process Status**	Analysis Succeeded	
**Plate [#] Name**		[1] 31598					**Instrument Sample Name**							**Spectra**	8	
**Rank**	**Protein Name**					**Accession No.**		**Protein MW**	**Protein PI**	**Pep. Count**	**Protein Score**	**Protein Score C. I. %**	**Total Ion Score**	**Total Ion C. I. %**		
1	serine/threonine-protein kinase transcriptional regulator [Mycobacterium bovis AF2122/97]					gi|31794259		119372.9	5.52	48	753	100	612	100		
	**Peptide Information**															
	**Calc. Mass**	**Obsrv. Mass**	**± da**	**± ppm**	**Start Seq.**	**End Seq.**	**Sequence**			**Ion Score**	**C. I. %**	**Modification**			**Rank**	**Result Type**
	846.5195	846.5083	-0.0112	-13	232	238	VIAQFLR									Mascot
	854.4518	854.4405	-0.0113	-13	35	42	GGFGVVYR									Mascot
	880.4424	880.4323	-0.0101	-11	118	124	HGPLDWR									Mascot
	932.4948	932.4852	-0.0096	-10	375	382	STLAAQWR									Mascot
	945.5363	945.5241	-0.0122	-13	109	116	NSLETLIR									Mascot
	977.505	977.492	-0.013	-13	792	800	AVAEVFADR									Mascot
	987.5217	987.5089	-0.0128	-13	564	573	GGGDATQLLR									Mascot
	1008.5584	1008.5454	-0.013	-13	133	142	LAGALEAAHR									Mascot
	1036.5436	1036.5291	-0.0145	-14	117	124	RHGPLDWR									Mascot
	1139.642	1139.6284	-0.0136	-12	239	248	ITSQPIPDLR									Mascot
	1179.5066	1179.4916	-0.015	-13	642	650	TEDDPNWFR									Mascot
	1179.5066	1179.4916	-0.015	-13	642	650	TEDDPNWFR			38	0					Mascot
	1223.6532	1223.6361	-0.0171	-14	456	465	IAVVIDDWHR									Mascot
	1252.7412	1252.7229	-0.0183	-15	363	374	LILIHAPSGFGK									Mascot
	1259.7106	1259.6946	-0.016	-13	877	887	LDIVAALQNFR									Mascot
		1259.6946														Mascot
	1263.6943	1263.6783	-0.016	-13	600	610	EFLLVASVTER									Mascot
	1263.6943	1263.6783	-0.016	-13	600	610	EFLLVASVTER			38	0					Mascot
	1267.7368	1267.7156	-0.0212	-17	239	249	ITSQPIPDLRK									Mascot
	1310.7063	1310.6873	-0.019	-14	250	262	QGLPADVAAAIER									Mascot
	1313.6696	1313.6522	-0.0174	-13	773	784	AELPEATQADLR									Mascot
	1361.7172	1361.6934	-0.0238	-17	792	803	AVAEVFADRVER									Mascot
	1432.7906	1432.7662	-0.0244	-17	336	348	YRPSVPTGSLVTR									Mascot
	1448.6892	1448.6676	-0.0216	-15	651	661	FHQMFADFLHR			55	77.837					Mascot
	1448.6892	1448.6676	-0.0216	-15	651	661	FHQMFADFLHR									Mascot
	1464.6841	1464.6588	-0.0253	-17	651	661	FHQMFADFLHR					Oxidation (M)[4]				Mascot
	1472.759	1472.735	-0.024	-16	505	518	IGDELAEIDSAALR									Mascot
	1484.8068	1484.7839	-0.0229	-15	967	981	LSTGGDTAVQLGLPR									Mascot
	1518.7693	1518.7469	-0.0224	-15	611	626	TCGGLASALAGITNGR					Carbamidomethyl (C)[2]				Mascot
	1529.8546	1529.8276	-0.027	-18	875	887	NRLDIVAALQNFR									Mascot
	1542.8057	1542.7795	-0.0262	-17	822	837	VPGTAGNTAALAAICR					Carbamidomethyl (C)[15]				Mascot
	1545.7867	1545.7667	-0.02	-13	56	68	VLSTDLDRDNLER									Mascot
	1622.772	1622.913	0.141	87	822	837	VPGTAGNTAALAAICR					Carbamidomethyl (C)[15], Phospho (ST)[4]				Mascot
	1631.9843	1631.957	-0.0273	-17	993	1009	LGIALPAAVAADLLAPR			144	100					Mascot
	1631.9843	1631.957	-0.0273	-17	993	1009	LGIALPAAVAADLLAPR									Mascot
	1681.8405	1681.8108	-0.0297	-18	888	904	TAFEVGTAVGAHSHAAR									Mascot
	1748.845	1748.8136	-0.0314	-18	519	535	FDTDEAAALLNDAGGLR									Mascot
	1748.845	1748.8136	-0.0314	-18	519	535	FDTDEAAALLNDAGGLR			136	100					Mascot
	1762.8137	1762.7791	-0.0346	-20	1032	1047	LLSAGDSADRDQACQR					Carbamidomethyl (C)[14]				Mascot
	1804.8937	1804.8573	-0.0364	-20	267	283	HPADRPATAADVGEELR									Mascot
	1846.9293	1846.8928	-0.0365	-20	539	556	ADVQALTTSTDGWAAAL			157	100					Mascot
							R									
	1846.9293	1846.8928	-0.0365	-20	539	556	ADVQALTTSTDGWAAAL									Mascot
							R									
	1907.8651	1907.8235	-0.0416	-22	1014	1031	DNGIATMTAELDEDSAVR									Mascot
	1914.9291	1914.8868	-0.0423	-22	704	720	AVDLVEQDETNLPEQSK									Mascot
	1931.8698	1931.8981	0.0283	15	1086	1102	NELAPVATKCAELGLSR					Phospho (ST)[16], Phospho (ST)[8]				Mascot
	1939.9794	1939.9385	-0.0409	-21	289	306	NGVSVDEMPLPVELGVE									Mascot
							R									
	1995.9663	1995.9725	0.0062	3	125	142	ETLSIGVKLAGALEAAHR					Phospho (ST)[4], Phospho (ST)[2]				Mascot
	2012.0117	2011.9703	-0.0414	-21	804	821	VDDLLAEAMSRPDTLPP					Oxidation (M)[9]				Mascot
							R									
	2090.1128	2090.0718	-0.041	-20	905	925	LAGSLLAELLYETGDLAG									Mascot
							AGR									
	2230.0696	2230.0239	-0.0457	-20	926	946	LMDESYLLGSEGGAVDY									Mascot
							LAAR									
	2243.2983	2243.2512	-0.0471	-21	1060	1080	RPLAALQAQILHIETLAAT			43	0					Mascot
		2243.2512														Mascot
							GR									
	2243.2983	2243.2512	-0.0471	-21	1060	1080	RPLAALQAQILHIETLAAT									Mascot
							GR									
	2246.0645	2246.0183	-0.0462	-21	926	946	LMDESYLLGSEGGAVDY					Oxidation (M)[2]				Mascot
							LAAR									
	2303.1487	2303.1021	-0.0466	-20	267	287	HPADRPATAADVGEELR									Mascot
							DVQR									
	2358.1797	2358.1292	-0.0505	-21	418	439	VRPTLAESLGHVLEEHG									Mascot
							DDAGR									
	2510.2773	2510.2205	-0.0568	-23	12	34	DLVPNIPAELLEAGFDNV									Mascot
							EEIGR									
	2632.3616	2632.3037	-0.0579	-22	149	172	DVKPGNILLTDYGEPQLT									Mascot

							DFGIAR								
	2739.291	2739.2261	-0.0649	-24	678	703	ASAWFAENGYLHEAVDH								Mascot
							ALAAGDPAR								
	2840.4312	2840.3696	-0.0616	-22	574	599	GLSGASDVIHEFLSENVL								Mascot
							DTLEPELR								
2	serine/threonine protein kinase K [Mycobacterium bovis					gi|154795739	119415.9	5.52	46	735	100	612	100		
	BCG str. Pasteur 1173P2]														
	**Peptide Information**														
	**Calc. Mass**	**Obsrv. Mass**	**± da**	**± ppm**	**Start**	**End**	**Sequence**		**Ion**	**C. I. %**	**Modification**			**Rank**	**Result Type**
					**Seq.**	**Seq.**			**Score**						
	846.5195	846.5083	-0.0112	-13	232	238	VIAQFLR								Mascot
	854.4518	854.4405	-0.0113	-13	35	42	GGFGVVYR								Mascot
	880.4424	880.4323	-0.0101	-11	118	124	HGPLDWR								Mascot
	932.4948	932.4852	-0.0096	-10	375	382	STLAAQWR								Mascot
	945.5363	945.5241	-0.0122	-13	109	116	NSLETLIR								Mascot
	977.505	977.492	-0.013	-13	792	800	AVAEVFADR								Mascot
	987.5217	987.5089	-0.0128	-13	564	573	GGGDATQLLR								Mascot
	1008.5584	1008.5454	-0.013	-13	133	142	LAGALEAAHR								Mascot
	1036.5436	1036.5291	-0.0145	-14	117	124	RHGPLDWR								Mascot
	1139.642	1139.6284	-0.0136	-12	239	248	ITSQPIPDLR								Mascot
	1179.5066	1179.4916	-0.015	-13	642	650	TEDDPNWFR								Mascot
	1179.5066	1179.4916	-0.015	-13	642	650	TEDDPNWFR		38	0					Mascot
	1223.6532	1223.6361	-0.0171	-14	456	465	IAVVIDDWHR								Mascot
	1252.7412	1252.7229	-0.0183	-15	363	374	LILIHAPSGFGK								Mascot
	1259.7106	1259.6946	-0.016	-13	877	887	LDIVAALQNFR								Mascot
	1263.6943	1263.6783	-0.016	-13	600	610	EFLLVASVTER								Mascot
	1263.6943	1263.6783	-0.016	-13	600	610	EFLLVASVTER		38	0					Mascot
	1267.7368	1267.7156	-0.0212	-17	239	249	ITSQPIPDLRK								Mascot
	1313.6696	1313.6522	-0.0174	-13	773	784	AELPEATQADLR								Mascot
	1361.7172	1361.6934	-0.0238	-17	792	803	AVAEVFADRVER								Mascot
	1448.6892	1448.6676	-0.0216	-15	651	661	FHQMFADFLHR		55	77.837					Mascot
	1448.6892	1448.6676	-0.0216	-15	651	661	FHQMFADFLHR								Mascot
	1464.6841	1464.6588	-0.0253	-17	651	661	FHQMFADFLHR				Oxidation (M)[4]				Mascot
	1472.759	1472.735	-0.024	-16	505	518	IGDELAEIDSAALR								Mascot
	1484.8068	1484.7839	-0.0229	-15	967	981	LSTGGDTAVQLGLPR								Mascot
	1518.7693	1518.7469	-0.0224	-15	611	626	TCGGLASALAGITNGR				Carbamidomethyl (C)[2]				Mascot
	1529.8546	1529.8276	-0.027	-18	875	887	NRLDIVAALQNFR								Mascot
	1542.8057	1542.7795	-0.0262	-17	822	837	VPGTAGNTAALAAICR				Carbamidomethyl (C)[15]				Mascot
	1545.7867	1545.7667	-0.02	-13	56	68	VLSTDLDRDNLER								Mascot
	1622.772	1622.913	0.141	87	822	837	VPGTAGNTAALAAICR				Carbamidomethyl (C)[15], Phospho (ST)[4]				Mascot
	1631.9843	1631.957	-0.0273	-17	993	1009	LGIALPAAVAADLLAPR		144	100					Mascot
	1631.9843	1631.957	-0.0273	-17	993	1009	LGIALPAAVAADLLAPR								Mascot
	1681.8405	1681.8108	-0.0297	-18	888	904	TAFEVGTAVGAHSHAAR								Mascot
	1748.845	1748.8136	-0.0314	-18	519	535	FDTDEAAALLNDAGGLR								Mascot
	1748.845	1748.8136	-0.0314	-18	519	535	FDTDEAAALLNDAGGLR		136	100					Mascot
	1762.8137	1762.7791	-0.0346	-20	1032	1047	LLSAGDSADRDQACQR				Carbamidomethyl (C)[14]				Mascot
	1804.8937	1804.8573	-0.0364	-20	267	283	HPADRPATAADVGEELR								Mascot
	1846.9293	1846.8928	-0.0365	-20	539	556	ADVQALTTSTDGWAAAL		157	100					Mascot
							R								
	1846.9293	1846.8928	-0.0365	-20	539	556	ADVQALTTSTDGWAAAL								Mascot
							R								
	1907.8651	1907.8235	-0.0416	-22	1014	1031	DNGIATMTAELDEDSAVR								Mascot
	1914.9291	1914.8868	-0.0423	-22	704	720	AVDLVEQDETNLPEQSK								Mascot
	1931.8698	1931.8981	0.0283	15	1086	1102	NELAPVATKCAELGLSR				Phospho (ST)[16], Phospho (ST)[8]				Mascot
	1939.9794	1939.9385	-0.0409	-21	289	306	NGVSVDEMPLPVELGVE								Mascot
							R								
	1995.9663	1995.9725	0.0062	3	125	142	ETLSIGVKLAGALEAAHR				Phospho (ST)[4], Phospho (ST)[2]				Mascot
	2012.0117	2011.9703	-0.0414	-21	804	821	VDDLLAEAMSRPDTLPP				Oxidation (M)[9]				Mascot
							R								
	2090.1128	2090.0718	-0.041	-20	905	925	LAGSLLAELLYETGDLAG								Mascot
							AGR								
	2230.0696	2230.0239	-0.0457	-20	926	946	LMDESYLLGSEGGAVDY								Mascot
							LAAR								
	2243.2983	2243.2512	-0.0471	-21	1060	1080	RPLAALQAQILHIETLAAT		43	0					Mascot
							GR								
	2243.2983	2243.2512	-0.0471	-21	1060	1080	RPLAALQAQILHIETLAAT								Mascot
							GR								
	2246.0645	2246.0183	-0.0462	-21	926	946	LMDESYLLGSEGGAVDY				Oxidation (M)[2]				Mascot
							LAAR								
	2303.1487	2303.1021	-0.0466	-20	267	287	HPADRPATAADVGEELR								Mascot
							DVQR								
	2358.1797	2358.1292	-0.0505	-21	418	439	VRPTLAESLGHVLEEHG								Mascot
							DDAGR								
	2510.2773	2510.2205	-0.0568	-23	12	34	DLVPNIPAELLEAGFDNV								Mascot
							EEIGR								
	2632.3616	2632.3037	-0.0579	-22	149	172	DVKPGNILLTDYGEPQLT								Mascot
							DFGIAR								
	2739.291	2739.2261	-0.0649	-24	678	703	ASAWFAENGYLHEAVDH								Mascot
		2739.2261													Mascot
							ALAAGDPAR								
	2840.4312	2840.3696	-0.0616	-22	574	599	GLSGASDVIHEFLSENVL								Mascot
							DTLEPELR								
**Gel Idx/Pos**		34/B7					**Instr./Gel Origin**		BA2060/121107A				**Process Status**	Analysis Succeeded	
**Plate [#] Name**		[1] 31598					**Instrument Sample Name**						**Spectra**	8	
**Rank**	**Protein Name**					**Accession No.**	**Protein MW**	**Protein**	**Pep.**	**Protein**	**Protein**	**Total Ion**	**Total Ion**		
								**PI**	**Count**	**Score**	**Score**	**Score**	**C. I. %**		
											**C. I. %**				
1	transcription termination factor Rho [Mycobacterium					gi|118471197	71704.7	6.71	24	158	100				
	smegmatis str. MC2 155]														
	**Peptide Information**														
	**Calc. Mass**	**Obsrv. Mass**	**± da**	**± ppm**	**Start**	**End**	**Sequence**		**Ion**	**C. I. %**	**Modification**			**Rank**	**Result Type**
					**Seq.**	**Seq.**			**Score**						
	861.494	861.4823	-0.0117	-14	369	375	KRPEFGK								Mascot
	870.4679	870.5305	0.0626	72	112	120	QAGAPVAEK								Mascot
	870.4679	870.5305	0.0626	72	112	120	QAGAPVAEK								Mascot
	1001.5486	1001.5614	0.0128	13	325	334	RGDAVTGAVR								Mascot
	1087.5742	1087.5573	-0.0169	-16	49	59	EIGVEGASGLR								Mascot
	1100.6058	1100.5905	-0.0153	-14	385	393	LRLETSPER								Mascot
	1102.5891	1102.5742	-0.0149	-14	376	384	LTPLYPNER								Mascot
	1102.5891	1102.5742	-0.0149	-14	376	384	LTPLYPNER		18	0					Mascot
	1107.5177	1107.5084	-0.0093	-8	510	520	AYNNASPASGR								Mascot
	1165.7052	1165.7242	0.019	16	408	418	GQRALIVSPPK								Mascot
	1165.7052	1165.7242	0.019	16	408	418	GQRALIVSPPK								Mascot
	1327.6892	1327.6654	-0.0238	-18	645	655	TNYEFLVQVSK								Mascot
	1358.7063	1358.6805	-0.0258	-19	591	603	VFPAVDVNPSGTR								Mascot
	1403.7562	1403.7301	-0.0261	-19	32	44	SASLSTMVLPELR								Mascot
	1419.7512	1419.7244	-0.0268	-19	32	44	SASLSTMVLPELR				Oxidation (M)[7]				Mascot
	1429.8009	1429.7726	-0.0283	-20	422	434	TTILQDIANAITR								Mascot
	1514.8074	1514.7772	-0.0302	-20	590	603	RVFPAVDVNPSGTR								Mascot
	1656.746	1656.7128	-0.0332	-20	172	187	AAEAPEAGESQESQPR								Mascot
	1673.9221	1673.8881	-0.034	-20	521	536	ILSGGVDSTALYPPKR								Mascot
	1702.7488	1702.7097	-0.0391	-23	219	233	SERDQDQQGGGQQNR								Mascot
	1854.0007	1853.958	-0.0427	-23	604	619	KDELLLSPDEFAIVHK								Mascot
	1884.0801	1884.0375	-0.0426	-23	490	506	LVEQGKDVVVLLDSITR								Mascot
	2073.9731	2073.9258	-0.0473	-23	301	319	TSGYLAGPNDVYVSMNM								Mascot

							VR									
	2078.1604	2078.1108	-0.0496	-24	623	641	VLSGLDSHQAIDLLISQLR									Mascot
	2206.2554	2206.2046	-0.0508	-23	623	642	VLSGLDSHQAIDLLISQLR									Mascot
							K									
	2398.1116	2398.0515	-0.0601	-25	78	102	TAEASSPATADAQTGEQ									Mascot
							AAEAPAPR									
	2710.343	2710.271	-0.072	-27	462	486	GEVIASTFDRPPSDHTQA									Mascot
							AELAIER									
	2910.3542	2910.282	-0.0722	-25	435	458	NNPECHLMVVLVDERPE					Carbamidomethyl (C)[5]				Mascot
							EVTDMQR									
**Gel Idx/Pos**		35/B8					**Instr./Gel Origin**			BA2060/121107A				**Process Status**	Analysis Succeeded	
**Plate [#] Name**		[1] 31598					**Instrument Sample Name**							**Spectra**	8	
**Rank**	**Protein Name**					**Accession No.**		**Protein MW**	**Protein PI**	**Pep. Count**	**Protein Score**	**Protein Score C. I. %**	**Total Ion Score**	**Total Ion C. I. %**		
1	transcription termination factor Rho [Mycobacterium smegmatis str. MC2 155]					gi|118471197		71704.7	6.71	26	327	100	238	100		
	**Peptide Information**															
	**Calc. Mass**	**Obsrv. Mass**	**± da**	**± ppm**	**Start Seq.**	**End Seq.**	**Sequence**			**Ion Score**	**C. I. %**	**Modification**			**Rank**	**Result Type**
	861.494	861.4862	-0.0078	-9	369	375	KRPEFGK									Mascot
	870.4679	870.5341	0.0662	76	112	120	QAGAPVAEK									Mascot
	870.4679	870.5341	0.0662	76	112	120	QAGAPVAEK			5	0					Mascot
	1001.5486	1001.5644	0.0158	16	325	334	RGDAVTGAVR									Mascot
	1087.5742	1087.5667	-0.0075	-7	49	59	EIGVEGASGLR									Mascot
	1100.6058	1100.5977	-0.0081	-7	385	393	LRLETSPER									Mascot
	1102.5891	1102.5808	-0.0083	-8	376	384	LTPLYPNER									Mascot
	1102.5891	1102.5808	-0.0083	-8	376	384	LTPLYPNER			26	0					Mascot
	1107.5177	1107.5112	-0.0065	-6	510	520	AYNNASPASGR									Mascot
	1165.7052	1165.7329	0.0277	24	408	418	GQRALIVSPPK									Mascot
	1229.71	1229.6971	-0.0129	-10	496	506	DVVVLLDSITR									Mascot
	1327.6892	1327.6702	-0.019	-14	645	655	TNYEFLVQVSK									Mascot
	1358.7063	1358.6932	-0.0131	-10	591	603	VFPAVDVNPSGTR									Mascot
	1403.7562	1403.7416	-0.0146	-10	32	44	SASLSTMVLPELR									Mascot
	1419.7512	1419.7369	-0.0143	-10	32	44	SASLSTMVLPELR					Oxidation (M)[7]				Mascot
	1429.8009	1429.7863	-0.0146	-10	422	434	TTILQDIANAITR									Mascot
	1514.8074	1514.7861	-0.0213	-14	590	603	RVFPAVDVNPSGTR									Mascot
	1656.746	1656.7239	-0.0221	-13	172	187	AAEAPEAGESQESQPR									Mascot
	1673.9221	1673.8937	-0.0284	-17	521	536	ILSGGVDSTALYPPKR									Mascot
	1702.7488	1702.7273	-0.0215	-13	219	233	SERDQDQQGGGQQNR									Mascot
	1854.0007	1853.9689	-0.0318	-17	604	619	KDELLLSPDEFAIVHK									Mascot
	1884.0801	1884.0531	-0.027	-14	490	506	LVEQGKDVVVLLDSITR									Mascot
	1993.7874	1993.9427	0.1553	78	521	536	ILSGGVDSTALYPPKR					Phospho (ST)[3,8], Phospho (ST)[9],				Mascot
												Phospho (Y)[12]				
	2073.9731	2073.9404	-0.0327	-16	301	319	TSGYLAGPNDVYVSMNM									Mascot
							VR									
	2078.1604	2078.1296	-0.0308	-15	623	641	VLSGLDSHQAIDLLISQLR									Mascot
	2078.1604	2078.1296	-0.0308	-15	623	641	VLSGLDSHQAIDLLISQLR			73	99.577					Mascot
	2089.968	2089.9331	-0.0349	-17	301	319	TSGYLAGPNDVYVSMNM					Oxidation (M)[15]				Mascot
							VR									
	2105.9629	2105.9214	-0.0415	-20	301	319	TSGYLAGPNDVYVSMNM					Oxidation (M)[15,17]				Mascot
							VR									
	2206.2554	2206.2178	-0.0376	-17	623	642	VLSGLDSHQAIDLLISQLR									Mascot
							K									
	2297.0044	2297.1589	0.1545	67	645	664	TNYEFLVQVSKTAPGSM					Oxidation (M)[17], Phospho (Y)[3]				Mascot
							DVD									
	2398.1116	2398.0706	-0.041	-17	78	102	TAEASSPATADAQTGEQ									Mascot
							AAEAPAPR									
	2398.1116	2398.0706	-0.041	-17	78	102	TAEASSPATADAQTGEQ			165	100					Mascot
							AAEAPAPR									
	2710.343	2710.2959	-0.0471	-17	462	486	GEVIASTFDRPPSDHTQA									Mascot
							AELAIER									
	2910.3542	2910.3025	-0.0517	-18	435	458	NNPECHLMVVLVDERPE					Carbamidomethyl (C)[5]				Mascot
							EVTDMQR									
	2949.2939	2949.29	-0.0039	-1	435	458	NNPECHLMVVLVDERPE					Oxidation (M)[8], Phospho (ST)[20]				Mascot
							EVTDMQR									
**Gel Idx/Pos**		36/B9					**Instr./Gel Origin**			BA2060/121107A				**Process Status**	Analysis Succeeded	
**Plate [#] Name**		[1] 31598					**Instrument Sample Name**							**Spectra**	8	
**Rank**	**Protein Name**					**Accession No.**		**Protein MW**	**Protein PI**	**Pep. Count**	**Protein Score**	**Protein Score C. I. %**	**Total Ion Score**	**Total Ion C. I. %**		
1	histidine ammonia-lyase [Mycobacterium smegmatis str. MC2 155]					gi|118471752		53354.7	5.86	21	778	100	746	100		
	**Peptide Information**															
	**Calc. Mass**	**Obsrv. Mass**	**± da**	**± ppm**	**Start Seq.**	**End Seq.**	**Sequence**			**Ion Score**	**C. I. %**	**Modification**			**Rank**	**Result Type**
	821.4628	821.4551	-0.0077	-9	69	75	HVAPELR									Mascot
	904.4958	904.4536	-0.0422	-47	255	262	TSASNLRR									Mascot
	1020.4857	1020.47	-0.0157	-15	475	484	DHVAGPGPDR									Mascot
	1210.5811	1210.5658	-0.0153	-13	85	96	SHAAGTGPEVER									Mascot
	1283.7682	1283.75	-0.0182	-14	441	452	NVLAIEILTAAR			65	97.388					Mascot
	1283.7682	1283.75	-0.0182	-14	441	452	NVLAIEILTAAR									Mascot
	1342.6783	1342.6614	-0.0169	-13	263	274	LLADSAIMESHR									Mascot
	1360.708	1360.6805	-0.0275	-20	472	484	ALRDHVAGPGPDR									Mascot
	1580.7373	1580.7095	-0.0278	-18	275	288	GPDCTVVQDAYSLR					Carbamidomethyl (C)[4]				Mascot
	1586.9012	1586.8735	-0.0277	-17	8	23	VGVGPLAPSDVLHVAR									Mascot
	1586.9012	1586.8735	-0.0277	-17	8	23	VGVGPLAPSDVLHVAR			92	99.994					Mascot
	1594.7407	1594.7233	-0.0174	-11	262	274	RLLADSAIMESHR					Oxidation (M)[9], Phospho (ST)[6]				Mascot
	1675.8762	1675.8447	-0.0315	-19	311	326	ELAAAVDNPVVLHDGR									Mascot
	1675.8762	1675.8447	-0.0315	-19	311	326	ELAAAVDNPVVLHDGR			88	99.984					Mascot
	1804.9109	1804.8784	-0.0325	-18	221	238	TADIAAAMSVEALLGTDR									Mascot
	1819.9562	1819.9235	-0.0327	-18	239	254	VFADDLQALRPHPGQR			53	57.044					Mascot
	1819.9562	1819.9235	-0.0327	-18	239	254	VFADDLQALRPHPGQR									Mascot
	1915.8828	1915.8469	-0.0359	-19	289	305	CSPQVHGAAHDTLEHTR					Carbamidomethyl (C)[1]				Mascot
	2550.3521	2550.3022	-0.0499	-20	167	192	DASGTLRPAADALAAAGL									Mascot
							QPVQLSEK									
	2550.3521	2550.3022	-0.0499	-20	167	192	DASGTLRPAADALAAAGL			158	100					Mascot
							QPVQLSEK									
	2589.3306	2589.2791	-0.0515	-20	43	68	DAVEQLAQAPVPAYGVS			224	100					Mascot
							TGFGALATR									
	2589.3306	2589.2791	-0.0515	-20	43	68	DAVEQLAQAPVPAYGVS									Mascot
							TGFGALATR									
	2832.4639	2832.4084	-0.0555	-20	41	68	SRDAVEQLAQAPVPAYG			67	98.059					Mascot
							VSTGFGALATR									
	2832.4639	2832.4084	-0.0555	-20	41	68	SRDAVEQLAQAPVPAYG									Mascot
							VSTGFGALATR									
	2904.3979	2904.3511	-0.0468	-16	263	288	LLADSAIMESHRGPDCTV					Carbamidomethyl (C)[16]				Mascot
							VQDAYSLR									
	2917.3455	2917.2859	-0.0596	-20	403	430	LATPASVDSIPSSAMQED									Mascot
							HVSMGWSSAR									
	2933.3403	2933.2827	-0.0576	-20	403	430	LATPASVDSIPSSAMQED					Oxidation (M)[15]				Mascot
							HVSMGWSSAR									
	2949.3352	2949.2842	-0.051	-17	403	430	LATPASVDSIPSSAMQED					Oxidation (M)[15,22]				Mascot
							HVSMGWSSAR									
	2979.2488	2979.4312	0.1824	61	139	166	EYGSLGCSGDLAPLAHC					Phospho (ST)[4], Phospho (Y)[2]				Mascot

							ALALMGEGEVR									
	3412.7205	3412.6516	-0.0689	-20	371	402	GLPPFLADDPGVDSGHM									Mascot
							IAQYTQASIVSELKR									
2	histidine ammonia-lyase [Streptomyces clavuligerus					gi|254389884	52552	5.9	5	88	98.469	88	99.984			
	ATCC 27064]															
	**Peptide Information**															
	**Calc. Mass**	**Obsrv. Mass**	**± da**	**± ppm**	**Start Seq.**	**End Seq.**	**Sequence**		**Ion Score**	**C. I. %**	**Modification**				**Rank**	**Result Type**
	882.408	882.5545	0.1465	166	447	454	TAVDALGR				Phospho (ST)[1]					Mascot
	1675.8762	1675.8447	-0.0315	-19	324	339	ELAAAVDNPVVLHDGR									Mascot
	1675.8762	1675.8447	-0.0315	-19	324	339	ELAAAVDNPVVLHDGR		88	99.984						Mascot
	2799.4487	2799.2385	-0.2102	-75	252	278	VLAPELHAVRPHPGQAA				Phospho (ST)[23]					Mascot
							AAANMSAVLK									
	2933.3186	2933.2827	-0.0359	-12	10	36	TLLNMDMHTVVVGTSGT				Phospho (ST)[1,9]					Mascot
							TADDVVAVAR									
	2949.3135	2949.2842	-0.0293	-10	10	36	TLLNMDMHTVVVGTSGT				Oxidation (M)[5], Phospho (ST)[1,9]					Mascot
							TADDVVAVAR									
	2979.1667	2979.4312	0.2645	89	56	81	SIVDTLAATPEPVYGVST				Phospho (ST)[1,17], Phospho (ST)[5,9],					Mascot
							GFGALASR				Phospho (Y)[14]					
**Gel Idx/Pos**		37/B10					**Instr./Gel Origin**		BA2060/121107A				**Process Status**	Analysis Succeeded		
**Plate [#] Name**		[1] 31598					**Instrument Sample Name**						**Spectra**	8		
**Rank**	**Protein Name**					**Accession No.**	**Protein MW**	**Protein PI**	**Pep. Count**	**Protein Score**	**Protein Score C. I. %**	**Total Ion Score**	**Total Ion C. I. %**			
1	hypothetical protein MSMEG_5010 [Mycobacterium					gi|118469659	19502.9	6	16	514	100	425	100			
	smegmatis str. MC2 155]															
	**Peptide Information**															
	**Calc. Mass**	**Obsrv. Mass**	**± da**	**± ppm**	**Start Seq.**	**End Seq.**	**Sequence**		**Ion Score**	**C. I. %**	**Modification**				**Rank**	**Result Type**
	837.4213	837.4078	-0.0135	-16	63	68	FREETR									Mascot
	870.4553	870.5247	0.0694	80	3	8	HQHHRR									Mascot
	917.4323	917.4154	-0.0169	-18	74	81	IADEAQDR									Mascot
	931.4995	931.485	-0.0145	-16	86	93	VSWGVEVR									Mascot
	1001.4958	1001.4702	-0.0256	-26	1	7	MRHQHHR									Mascot
	1004.5159	1004.4965	-0.0194	-19	135	143	SDALAWSVR		45	0						Mascot
	1004.5159	1004.4965	-0.0194	-19	135	143	SDALAWSVR									Mascot
	1017.4907	1017.4682	-0.0225	-22	1	7	MRHQHHR				Oxidation (M)[1]					Mascot
	1059.5945	1059.5759	-0.0186	-18	85	93	KVSWGVEVR									Mascot
	1059.5945	1059.5759	-0.0186	-18	85	93	KVSWGVEVR		62	95.257						Mascot
	1247.6492	1247.6222	-0.027	-22	133	143	SRSDALAWSVR									Mascot
	1247.6492	1247.6222	-0.027	-22	133	143	SRSDALAWSVR		60	92.904						Mascot
	1277.6233	1277.593	-0.0303	-24	74	84	IADEAQDRFGR									Mascot
	1309.5807	1309.5553	-0.0254	-19	9	20	DASEAAEWFAGR									Mascot
	1309.5807	1309.5553	-0.0254	-19	9	20	DASEAAEWFAGR		113	100						Mascot
	1356.7482	1356.7175	-0.0307	-23	120	132	QVLDTLVDAGVAR									Mascot
	1366.7114	1366.6792	-0.0322	-24	144	155	LVGQHADEWLAK									Mascot
	1366.7114	1366.6792	-0.0322	-24	144	155	LVGQHADEWLAK		61	94.752						Mascot
	1465.6819	1465.6459	-0.036	-25	8	20	RDASEAAEWFAGR									Mascot
	1515.8175	1515.7826	-0.0349	-23	101	113	IMFTHIAVPVMTR									Mascot
	1515.8175	1515.7826	-0.0349	-23	101	113	IMFTHIAVPVMTR		83	99.965						Mascot
	1531.8124	1531.7721	-0.0403	-26	101	113	IMFTHIAVPVMTR				Oxidation (M)[2]					Mascot
	1531.8124	1531.7721	-0.0403	-26	101	113	IMFTHIAVPVMTR		31	0	Oxidation (M)[2]					Mascot
	1547.8073	1547.7632	-0.0441	-28	101	113	IMFTHIAVPVMTR				Oxidation (M)[2,11]					Mascot
	1787.8962	1787.8499	-0.0463	-26	21	36	LPDGWFTGDPTVIVDR									Mascot
	2044.0081	2043.9503	-0.0578	-28	37	55	EEITVIGTLPDVEGESEAR									Mascot
	2283.907	2284.113	0.206	90	37	55	EEITVIGTLPDVEGESEAR				Phospho (ST)[16], Phospho (ST)[4,8]					Mascot
2	hypothetical protein Mmcs_3943 [Mycobacterium sp.					gi|108800907	22579.4	5.89	6	123	100	105	100			
	MCS]															
	**Peptide Information**															
	**Calc. Mass**	**Obsrv. Mass**	**± da**	**± ppm**	**Start Seq.**	**End Seq.**	**Sequence**		**Ion Score**	**C. I. %**	**Modification**				**Rank**	**Result Type**
	917.4331	917.4154	-0.0177	-19	12	18	CNHVIMK				Carbamidomethyl (C)[1], Oxidation (M)[6]					Mascot
	996.4761	996.5776	0.1015	102	66	73	EEITVIGR				Phospho (ST)[4]					Mascot
	1004.5159	1004.4965	-0.0194	-19	165	173	SDALAWSVR		45	0						Mascot
	1004.5159	1004.4965	-0.0194	-19	165	173	SDALAWSVR									Mascot
	1247.6492	1247.6222	-0.027	-22	163	173	SRSDALAWSVR									Mascot
	1247.6492	1247.6222	-0.027	-22	163	173	SRSDALAWSVR		60	92.904						Mascot
	1356.7482	1356.7175	-0.0307	-23	150	162	QVLDTLVDAGVAR									Mascot
	1577.8274	1577.7028	-0.1246	-79	131	143	ILFTHIAVPVMTR				Phospho (ST)[4]					Mascot
	1593.8223	1593.6909	-0.1314	-82	131	143	ILFTHIAVPVMTR				Oxidation (M)[11], Phospho (ST)[4]					Mascot

Table S3: Mass Spectrometry Analysis

Spot number	MALDI well number	Match Quality	Top Ranked Protein Name [Species]	NCBI Accession No.	Protein MW	Protein PI	Pep.Count	Protein Score	Protein Score C.I.%	Total Ion Score	Total Ion C.I.%
**4**	**G21**		ATP synthase subunit beta OS=Mycobacterium smegmatis (strain ATCC 700084 / mc(2)155) GN=atpD PE=1 S	ATPB_MYCS2	51,584	4.8	5	26	0	26	99
**9**	**G22**		transcription termination factor Rho [Mycobacterium smegmatis str. MC2 155]	gi|118471197	71,705	6.7	25	262	100	195	100
**11**	**G23**		1-deoxy-D-xylulose-5-phosphate synthase OS=Mycobacterium smegmatis (strain ATCC 700084 /mc(2)155)	DXS_MYCS2	67,993	5.7	5	19	0	19	96
**14**	**G24**		negative regulator of genetic competence ClpC/mecB [Mycobacterium smegmatis str. MC2 155]	gi|118472509	93,496	5.5	41	688	100	550	100
**15**	**H1**		isocitrate dehydrogenase [Mycobacterium smegmatis str. MC2 155]	gi|118467432	82,552	5.6	33	271	100	193	100
**16**	**H2**		ribonucleotide-diphosphate reductase subunit alpha [Mycobacterium smegmatis str. MC2 155]	gi|118468034	80,496	5.7	26	344	100	276	100
**20**	**H3**		metallopeptidase [Mycobacterium smegmatis str. MC2 155]	gi|118467687	74,084	4.8	34	718	100	615	100
**22**	**H4**		1-pyrroline-5-carboxylate dehydrogenase [Mycobacterium smegmatis str. MC2 155]	gi|118469637	58,397	5.5	24	700	100	615	100
**27**	**H5**		ATP-dependent rna helicase, dead/deah box family protein [Mycobacterium smegmatis str. MC2 155]	gi|118470234	63,685	7.3	20	252	100	217	100
**44**	**H6**		acetyl-CoA acetyltransferase [Mycobacterium smegmatis str. MC2 155]	gi|118468168	45,205	6.13	12	225	100	221	100
**53**	**H7**		S30AE family protein [Mycobacterium smegmatis str. MC2 155]	gi|118473146	26,382	6.27	15	253	100	217	100
**70**	**H8**		hypothetical protein MSMEG_5010 [Mycobacterium smegmatis str. MC2 155]	gi|118469659	19,503	6.0	18	426	100	293	100

Control

**1 fmol**	**H9**		Beta-galactosidase OS=Escherichia coli O127:H6 (strain E2348/69 / EPEC) GN=lacZ PE=3 SV=1	BGAL_ECO27	116,343	5.2	22	312	100	209	100
**2 fmol**	**H10**		Beta-galactosidase OS=Escherichia coli O127:H6 (strain E2348/69 / EPEC) GN=lacZ PE=3 SV=1	BGAL_ECO27	116,343	5.2	23	450	100	338	100
**5 fmol**	**H11**		Beta-galactosidase OS=Escherichia coli (strain K12) GN=lacZ PE=1 SV=2	BGAL_ECOLI	116,409	5.3	24	492	100	371	100
**10 fmol**	**H12**		Beta-galactosidase OS=Escherichia coli (strain K12) GN=lacZ PE=1 SV=2	BGAL_ECOLI	116,409	5.3	25	520	100	391	100

high confidence
low confidence
no confidence

2012_11

MAVA121113

Analysis Information

**Report Type**	Protein-Peptide Summary by Spot	**Analysis Type**	Combined (MS+MS/MS)
**Sample Set Name**	MAVA121113	**Database**	NCBInr
**Analysis Name**	M.Smegmatis_P	**Creation Date**	
**Reported By**		**Last Modified**	
**MS Acq. : Proc. Methods**	(Unspecified) : (Unspecified)		
**Interpretation Method**	(Unspecified)		

**Gel Idx/Pos**	170/G21	**Instr./Gel Origin**	BA2060/121115A	**Process Status**	Analysis Succeeded
**Plate [#] Name**	[1] 31598	**Instrument Sample Name**		**Spectra**	11

Rank	Protein Name	Accession No.	Protein MW	Protein PI	Pep. Count	Protein Score	Protein Score C. I. %	Total Ion Score	Total Ion C. I. %
1	ATP synthase subunit beta OS=Mycobacterium smegmatis (strain ATCC 700084 / mc(2)155) GN=atpD PE=1 S	ATPB_MYCS2	51584.4	4.84	5	26	0	26	98.682

Peptide Information

Calc. Mass	Obsrv. Mass	± da	± ppm	Start Seq.	End Seq.	Sequence	Ion Score	C. I. %	Modification	Rank	Result Type
856.5111	856.483	-0.0281	-33	400	406	QLVNRAR					Mascot
870.4193	870.4999	0.0806	93	294	300	ITSTRGR	15	81.297	Phospho (ST)[3]		Mascot
870.4193	870.4999	0.0806	93	294	300	ITSTRGR			Phospho (ST)[2]		Mascot
1165.465	1165.6904	0.2254	193	2	11	TATAEKTAGR	12	62.51	Phospho (ST)[3,7]		Mascot
1165.465	1165.6904	0.2254	193	2	11	TATAEKTAGR			Phospho (ST)[1,3]		Mascot
1334.6157	1334.8275	0.2118	159	143	152	VVDLLTPYVR			Phospho (ST)[6], Phospho (Y)[8]		Mascot
2283.1929	2283.0503	-0.1426	-62	156	176	IALFGGAGVGKTVLIQEMI			Oxidation (M)[18], Phospho (ST)[12]		Mascot
						NR					
2283.1929	2283.0503	-0.1426	-62	156	176	IALFGGAGVGKTVLIQEMI			Oxidation (M)[18], Phospho (ST)[12]		Mascot
						NR					

**Gel Idx/Pos**	171/G22	**Instr./Gel Origin**	BA2060/121115A	**Process Status**	Analysis Succeeded
**Plate [#] Name**	[1] 31598	**Instrument Sample Name**		**Spectra**	8

Rank	Protein Name	Accession No.	Protein MW	Protein PI	Pep. Count	Protein Score	Protein Score C. I. %	Total Ion Score	Total Ion C. I. %
1	transcription termination factor Rho [Mycobacterium smegmatis str. MC2 155]	gi|118471197	71704.7	6.71	25	262	100	195	100

Peptide Information

Calc. Mass	Obsrv. Mass	± da	± ppm	Start Seq.	End Seq.	Sequence	Ion Score	C. I. %	Modification	Rank	Result Type
861.494	861.4538	-0.0402	-47	369	375	KRPEFGK					Mascot
870.4679	870.5002	0.0323	37	112	120	QAGAPVAEK					Mascot
870.4679	870.5002	0.0323	37	112	120	QAGAPVAEK					Mascot
1000.6149	1000.5636	-0.0513	-51	60	68	KSELIAAIR					Mascot
1001.5486	1001.5173	-0.0313	-31	325	334	RGDAVTGAVR					Mascot
1087.5742	1087.5203	-0.0539	-50	49	59	EIGVEGASGLR					Mascot
1102.5891	1102.5358	-0.0533	-48	376	384	LTPLYPNER	17	0			Mascot
1102.5891	1102.5358	-0.0533	-48	376	384	LTPLYPNER					Mascot
1107.5177	1107.4714	-0.0463	-42	510	520	AYNNASPASGR					Mascot
	1107.4714										Mascot
1165.7052	1165.6866	-0.0186	-16	408	418	GQRALIVSPPK					Mascot
1229.71	1229.6475	-0.0625	-51	496	506	DVVVLLDSITR					Mascot
1327.6892	1327.6201	-0.0691	-52	645	655	TNYEFLVQVSK					Mascot
1358.7063	1358.6399	-0.0664	-49	591	603	VFPAVDVNPSGTR					Mascot
1403.7562	1403.6868	-0.0694	-49	32	44	SASLSTMVLPELR					Mascot
1419.7512	1419.6788	-0.0724	-51	32	44	SASLSTMVLPELR			Oxidation (M)[7]		Mascot
1429.8009	1429.7295	-0.0714	-50	422	434	TTILQDIANAITR					Mascot
1429.8009	1429.7295	-0.0714	-50	422	434	TTILQDIANAITR	44	0			Mascot
1514.8074	1514.7292	-0.0782	-52	590	603	RVFPAVDVNPSGTR					Mascot
1656.746	1656.6627	-0.0833	-50	172	187	AAEAPEAGESQESQPR					Mascot
1702.7488	1702.6627	-0.0861	-51	219	233	SERDQDQQGGGQQNR					Mascot
1854.0007	1853.9037	-0.097	-52	604	619	KDELLLSPDEFAIVHK					Mascot
1884.0801	1883.986	-0.0941	-50	490	506	LVEQGKDVVVLLDSITR					Mascot
2073.9731	2073.8679	-0.1052	-51	301	319	TSGYLAGPNDVYVSMNM					Mascot
						VR					
2078.1604	2078.0542	-0.1062	-51	623	641	VLSGLDSHQAIDLLISQLR					Mascot
2297.0044	2297.0825	0.0781	34	645	664	TNYEFLVQVSKTAPGSM			Oxidation (M)[17], Phospho (Y)[3]		Mascot
						DVD					
2298.0293	2298.0725	0.0432	19	301	320	TSGYLAGPNDVYVSMNM			Oxidation (M)[15], Phospho (Y)[4]		Mascot
						VRK					
2398.1116	2397.99	-0.1216	-51	78	102	TAEASSPATADAQTGEQ					Mascot
						AAEAPAPR					
2398.1116	2397.99	-0.1216	-51	78	102	TAEASSPATADAQTGEQ	128	100			Mascot
						AAEAPAPR					
2710.343	2710.2104	-0.1326	-49	462	486	GEVIASTFDRPPSDHTQA	23	0			Mascot
						AELAIER					
2710.343	2710.2104	-0.1326	-49	462	486	GEVIASTFDRPPSDHTQA					Mascot
						AELAIER					
2910.3542	2910.2083	-0.1459	-50	435	458	NNPECHLMVVLVDERPE			Carbamidomethyl (C)[5]		Mascot
						EVTDMQR					

Rank	Protein Name	Accession No.	Protein MW	Protein PI	Pep. Count	Protein Score	Protein Score C. I. %	Total Ion Score	Total Ion C. I. %
2	transcription termination factor Rho [Mycobacterium leprae Br4923]	gi|221230040	66063.6	5.31	15	58	0	44	0

Peptide Information

Calc. Mass	Obsrv. Mass	± da	± ppm	Start Seq.	End Seq.	Sequence	Ion Score	C. I. %	Modification	Rank	Result Type
861.494	861.4538	-0.0402	-47	315	321	KRPEFGK					Mascot
	861.4538										Mascot
1001.5486	1001.5173	-0.0313	-31	273	282	RGDAVTGAVR					Mascot
1107.5177	1107.4714	-0.0463	-42	456	466	AYNNASPASGR					Mascot
1165.7052	1165.6866	-0.0186	-16	354	364	GQRALIVSPPK					Mascot
1229.71	1229.6475	-0.0625	-51	442	452	DVVVLLDSITR					Mascot
1327.6892	1327.6201	-0.0691	-52	591	601	TNYEFLVQVSK					Mascot
1358.7063	1358.6399	-0.0664	-49	537	549	VFPAVDVNPSGTR					Mascot
1429.8009	1429.7295	-0.0714	-50	368	380	TTILQDIANAITR					Mascot
1429.8009	1429.7295	-0.0714	-50	368	380	TTILQDIANAITR	44	0			Mascot
1514.8074	1514.7292	-0.0782	-52	536	549	RVFPAVDVNPSGTR					Mascot
1884.0801	1883.986	-0.0941	-50	436	452	LVEQGKDVVVLLDSITR					Mascot
2230.063	2230.0942	0.0312	14	249	268	TSGYLAGPHDVYVSMSM			Oxidation (M)[15,17]		Mascot
						VRK					
2247.0122	2246.989	-0.0232	-10	591	610	TNYEFLVQVSKTTPGSM					Mascot
						DDD					
2384.1445	2383.8267	-0.3178	-133	569	588	VLSGLDSHQAIDLLMSQL			Phospho (ST)[3,7]		Mascot
						RK					
2565.7712	2566.1416	0.3704	144	249	267	TSGYLAGPHDVYVSMSM			Oxidation (M)[15], Phospho (ST)[2,14,16],		Mascot
						VR			Phospho (ST)[1], Phospho (Y)[4,12]		
2709.8611	2710.2104	0.3493	129	249	268	TSGYLAGPHDVYVSMSM			Oxidation (M)[15,17], Phospho (ST)[2,14,16],		Mascot
						VRK			Phospho (ST)[1], Phospho (Y)[4,12]		
2709.8611	2710.2104	0.3493	129	249	268	TSGYLAGPHDVYVSMSM			Oxidation (M)[15,17], Phospho (ST)[2,14,16],		Mascot
						VRK			Phospho (ST)[1], Phospho (Y)[4,12]		
3337.3098	3337.5999	0.2901	87	92	120	DHGGSATAISTEALAAQE			Phospho (ST)[5,10], Phospho (ST)[7],		Mascot
						EQNYAIVEVSR			Phospho (Y)[22]		

**Gel Idx/Pos**	172/G23	**Instr./Gel Origin**	BA2060/121115A	**Process Status**	Analysis Succeeded
**Plate [#] Name**	[1] 31598	**Instrument Sample Name**		**Spectra**	8

Rank	Protein Name	Accession No.	Protein MW	Protein PI	Pep. Count	Protein Score	Protein Score C. I. %	Total Ion Score	Total Ion C. I. %

											C. I. %				
1	1-deoxy-D-xylulose-5-phosphate synthase				DXS_MYCS2		67992.9	5.7	5	19	0	19	95.991		
	OS=Mycobacterium smegmatis (strain ATCC 700084 /														
	mc(2)155)														
	Peptide Information														
	Calc. Mass	Obsrv. Mass	± da	± ppm	Start	End	Sequence		Ion	C. I. %	Modification			Rank	Result Type
					Seq.	Seq.			Score						
	1102.6289	1102.5399	-0.089	-81	514	523	MALAVAELLR				Oxidation (M)[1]				Mascot
	1307.6355	1307.6167	-0.0188	-14	524	535	SQGIGVTVVDPR				Phospho (ST)[1]				Mascot
	2225.0784	2225.0132	-0.0652	-29	333	353	DVVAITAAMPGPTGLSAF				Phospho (ST)[6]				Mascot
							RDR								
	2225.0784	2225.0132	-0.0652	-29	333	353	DVVAITAAMPGPTGLSAF		19	95.991	Phospho (ST)[6]				Mascot
							RDR								
	2269.9841	2269.9272	-0.0569	-25	332	351	RDVVAITAAMPGPTGLSA				Phospho (ST)[17], Phospho (ST)[7,14]				Mascot
							FR								
	2720.9126	2720.1482	-0.7644	-281	311	331	ASGTSWTSVFSDELISQA				Phospho (ST)[2,5,8,11], Phospho (ST)[4,7]				Mascot
							AKR								
Gel Idx/Pos	173/G24						Instr./Gel Origin	BA2060/121115A					Process Status	Analysis Succeeded	
Plate [#] Name	[1] 31598						Instrument Sample Name						Spectra	8	
Rank	Protein Name				Accession No.		Protein MW	Protein	Pep.	Protein	Protein	otal Ion	Total Ion		
								PI	Count	Score	Score	Score	C. I. %		
											C. I. %				
1	negative regulator of genetic competence ClpC/mecB				gi|118472509		93496	5.45	41	688	100	550	100		
	[Mycobacterium smegmatis str. MC2 155]														
	Peptide Information														
	Calc. Mass	Obsrv. Mass	± da	± ppm	Start	End	Sequence		Ion	C. I. %	Modification			Rank	Result Type
					Seq.	Seq.			Score						
	812.3798	812.3414	-0.0384	-47	366	371	YEAHHR								Mascot
	829.5141	829.4765	-0.0376	-45	87	93	VLELSLR								Mascot
	832.4709	832.437	-0.0339	-41	200	206	VMQVLSR								Mascot
	840.4249	840.388	-0.0369	-44	818	825	FTFSGGPK								Mascot
	900.4608	900.4171	-0.0437	-49	415	422	MTAPPDLR								Mascot
	931.4189	931.4565	0.0376	40	510	516	MEEELHK				Oxidation (M)[1]				Mascot
	996.5472	996.5475	0.0003	0	695	702	QKVHDELK								Mascot
	1071.4854	1071.4399	-0.0455	-42	261	268	YRGDFEER								Mascot
	1083.5078	1083.4543	-0.0535	-49	364	371	DRYEAHHR								Mascot
	1087.52	1087.5461	0.0261	24	510	517	MEEELHKR				Oxidation (M)[1]				Mascot
	1113.6262	1113.5708	-0.0554	-50	12	21	VVVLAQEEAR								Mascot
	1143.6005	1143.5475	-0.053	-46	399	409	AIDLIDEAGAR								Mascot
	1215.6382	1215.5828	-0.0554	-46	704	712	HFRPEFLNR		20	0					Mascot
	1215.6382	1215.5828	-0.0554	-46	704	712	HFRPEFLNR								Mascot
	1269.7273	1269.6663	-0.061	-48	11	21	RVVVLAQEEAR								Mascot
	1293.7161	1293.6525	-0.0636	-49	210	222	NNPVLIGEPGVGK								Mascot
	1359.7413	1359.6835	-0.0578	-43	196	206	EIERVMQVLSR								Mascot
	1359.7478	1359.6835	-0.0643	-47	48	60	SLESLGISLEGVR		67	98.246					Mascot
	1461.806	1461.7352	-0.0708	-48	136	148	QQVIQLLSGYQGK								Mascot
	1550.8464	1550.7694	-0.077	-50	617	629	KPFSVVLFDEIEK								Mascot
	1563.8278	1563.7537	-0.0741	-47	544	559	RPSGSFIFAGPSGVGK		69	98.86					Mascot
	1563.8278	1563.7537	-0.0741	-47	544	559	RPSGSFIFAGPSGVGK								Mascot
	1592.8643	1592.7865	-0.0778	-49	246	260	QLYTLDLGSLVAGSR								Mascot
	1666.8282	1666.7502	-0.078	-47	315	329	GELQTIGATTLDEYR								Mascot
	1691.8633	1691.7784	-0.0849	-50	372	388	VSITDSAMVAAATLADR								Mascot
	1706.9475	1706.8636	-0.0839	-49	616	629	RKPFSVVLFDEIEK								Mascot
	1784.8197	1784.7339	-0.0858	-48	676	692	AVGLGFSQGGSENNYER								Mascot
	1784.8197	1784.7339	-0.0858	-48	676	692	AVGLGFSQGGSENNYER		71	99.28					Mascot
	1794.9232	1794.8361	-0.0871	-49	315	330	GELQTIGATTLDEYRK								Mascot
	1809.9592	1809.8735	-0.0857	-47	659	675	NTVLIFTSNLGTSDISK								Mascot
	1820.8773	1820.7891	-0.0882	-48	159	176	GGESGNPSTSLVLDQFG		83	99.961					Mascot
							R								
	1820.8773	1820.7891	-0.0882	-48	159	176	GGESGNPSTSLVLDQFG								Mascot
							R								
	1985.0087	1984.9116	-0.0971	-49	630	646	AHQEIYNSLLQVLEDGR		124	100					Mascot
	1985.0087	1984.9116	-0.0971	-49	630	646	AHQEIYNSLLQVLEDGR								Mascot
	2083.989	2083.8875	-0.1015	-49	826	848	RAETAEPDLAGAGAAGA								Mascot
							PTAGTE								
	2112.0283	2111.929	-0.0993	-47	594	613	LFGAPPGYVGYEEGGQL								Mascot
							TEK								
	2161.1863	2161.0808	-0.1055	-49	223	243	TAVVEGLAQAIVHGEVPE								Mascot
							TLK								
	2164.1648	2164.0605	-0.1043	-48	342	360	FQPVQVGEPTVEHTIEILK								Mascot
	2283.8542	2284.0649	0.2107	92	676	694	AVGLGFSQGGSENNYER				Phospho (ST)[7,11], Phospho (Y)[15]				Mascot
							MK								
	2320.2659	2320.1504	-0.1155	-50	341	360	RFQPVQVGEPTVEHTIEI								Mascot
							LK								
	2360.3083	2360.1919	-0.1164	-49	94	114	EALQLGHNYIGTEHILLGL								Mascot
							IR								
	2404.3081	2404.189	-0.1191	-50	223	245	TAVVEGLAQAIVHGEVPE								Mascot
							TLKDK								
	2476.2578	2476.1362	-0.1216	-49	61	83	SQVEEIIGQGQQAPSGHI								Mascot
							PFTPR								
	2476.2578	2476.1362	-0.1216	-49	61	83	SQVEEIIGQGQQAPSGHI		140	100					Mascot
							PFTPR								
	2723.2769	2723.1482	-0.1287	-47	565	588	ALANFLFGDDDALIQIDM								Mascot
							GEFHDR								
	2739.272	2739.1421	-0.1299	-47	565	588	ALANFLFGDDDALIQIDM				Oxidation (M)[18]				Mascot
							GEFHDR								
	2829.4714	2829.3354	-0.136	-48	22	47	MLNHNYIGTEHILLGLIHE								Mascot
							GEGVAAK								
	3087.4792	3087.343	-0.1362	-44	789	817	ILFEEIGPGQLVTVDVEG								Mascot
							WDGEGQGEDAK								
2	ATP-dependent Clp protease, ATP-binding subunit				gi|15843209		93467.2	5.64	34	490	100	396	100		
	ClpC [Mycobacterium tuberculosis CDC1551]														
	Peptide Information														
	Calc. Mass	Obsrv. Mass	± da	± ppm	Start	End	Sequence		Ion	C. I. %	Modification			Rank	Result Type
					Seq.	Seq.			Score						
	812.3798	812.3414	-0.0384	-47	366	371	YEAHHR								Mascot
	829.5141	829.4765	-0.0376	-45	87	93	VLELSLR								Mascot
	832.4709	832.437	-0.0339	-41	200	206	VMQVLSR								Mascot
	900.4608	900.4171	-0.0437	-49	415	422	MTAPPDLR								Mascot
	931.4189	931.4565	0.0376	40	510	516	MEEELHK				Oxidation (M)[1]				Mascot
	996.4852	996.5475	0.0623	63	730	737	MVDLMISR				Oxidation (M)[1,5]				Mascot
	1071.4854	1071.4399	-0.0455	-42	261	268	YRGDFEER								Mascot
	1083.5078	1083.4543	-0.0535	-49	364	371	DRYEAHHR								Mascot
	1087.52	1087.5461	0.0261	24	510	517	MEEELHKR				Oxidation (M)[1]				Mascot
	1113.6262	1113.5708	-0.0554	-50	12	21	VVVLAQEEAR								Mascot
	1143.6005	1143.5475	-0.053	-46	399	409	AIDLIDEAGAR								Mascot
	1215.6382	1215.5828	-0.0554	-46	704	712	HFRPEFLNR		20	0					Mascot
	1215.6382	1215.5828	-0.0554	-46	704	712	HFRPEFLNR								Mascot
	1293.7161	1293.6525	-0.0636	-49	210	222	NNPVLIGEPGVGK								Mascot
	1359.7413	1359.6835	-0.0578	-43	196	206	EIERVMQVLSR								Mascot
	1359.7478	1359.6835	-0.0643	-47	48	60	SLESLGISLEGVR		67	98.246					Mascot
	1461.806	1461.7352	-0.0708	-48	136	148	QQVIQLLSGYQGK								Mascot
	1550.8464	1550.7694	-0.077	-50	617	629	KPFSVVLFDEIEK								Mascot
		1550.7694													Mascot
	1563.8278	1563.7537	-0.0741	-47	544	559	RPSGSFIFAGPSGVGK		69	98.86					Mascot
	1563.8278	1563.7537	-0.0741	-47	544	559	RPSGSFIFAGPSGVGK								Mascot
	1592.8499	1592.7865	-0.0634	-40	730	743	MVDLMISRVAGQLK				Oxidation (M)[1,5]				Mascot

	1666.8282	1666.7502	-0.078	-47	315	329	GELQTIGATTLDEYR									Mascot
	1691.8633	1691.7784	-0.0849	-50	372	388	VSITDAAMVAAATLADR					Oxidation (M)[8]				Mascot
	1706.9475	1706.8636	-0.0839	-49	616	629	RKPFSVVLFDEIEK									Mascot
	1794.9232	1794.8361	-0.0871	-49	315	330	GELQTIGATTLDEYRK									Mascot
	1985.0087	1984.9116	-0.0971	-49	630	646	AHQEIYNSLLQVLEDGR			124	100					Mascot
	1985.0087	1984.9116	-0.0971	-49	630	646	AHQEIYNSLLQVLEDGR									Mascot
	2112.0283	2111.929	-0.0993	-47	594	613	LFGAPPGYVGYEEGGQL									Mascot
							TEK									
	2161.1863	2161.0808	-0.1055	-49	223	243	TAVVEGLAQAIVHGEVPE									Mascot
							TLK									
	2164.1648	2164.0605	-0.1043	-48	342	360	FQPVQVGEPTVEHTIEILK									Mascot
	2320.2659	2320.1504	-0.1155	-50	341	360	RFQPVQVGEPTVEHTIEI									Mascot
							LK									
	2360.3083	2360.1919	-0.1164	-49	94	114	EALQLGHNYIGTEHILLGL									Mascot
							IR									
	2404.3081	2404.189	-0.1191	-50	223	245	TAVVEGLAQAIVHGEVPE									Mascot
							TLKDK									
	2476.2578	2476.1362	-0.1216	-49	61	83	SQVEEIIGQGQQAPSGHI									Mascot
							PFTPR									
	2476.2578	2476.1362	-0.1216	-49	61	83	SQVEEIIGQGQQAPSGHI			140	100					Mascot
							PFTPR									
	2723.2769	2723.1482	-0.1287	-47	565	588	ALANFLFGDDDALIQIDM									Mascot
							GEFHDR									
	2739.272	2739.1421	-0.1299	-47	565	588	ALANFLFGDDDALIQIDM					Oxidation (M)[18]				Mascot
							GEFHDR									
	2780.3518	2779.8145	-0.5373	-193	159	186	GGESGSPSTSLVLDQFG									Mascot
							RNLTAAAMEGK									
	2780.3518	2780.3076	-0.0442	-16	159	186	GGESGSPSTSLVLDQFG									Mascot
							RNLTAAAMEGK									
	2829.4714	2829.3354	-0.136	-48	22	47	MLNHNYIGTEHILLGLIHE									Mascot
							GEGVAAK									
3	ATP-dependent protease ATP-binding subunit ClpC1					gi|183985068		93677.1	5.59	33	483	100	396	100		
	[Mycobacterium marinum M]															
	**Peptide Information**															
	**Calc. Mass**	**Obsrv. Mass**	**± da**	**± ppm**	**Start Seq.**	**End Seq.**	**Sequence**			**Ion Score**	**C. I. %**	**Modification**			**Rank**	**Result Type**
	812.3798	812.3414	-0.0384	-47	366	371	YEAHHR									Mascot
	829.5141	829.4765	-0.0376	-45	87	93	VLELSLR									Mascot
	832.4709	832.437	-0.0339	-41	200	206	VMQVLSR									Mascot
	900.4608	900.4171	-0.0437	-49	415	422	MTAPPDLR									Mascot
	996.4219	996.5475	0.1256	126	415	422	MTAPPDLR					Oxidation (M)[1], Phospho (ST)[2]				Mascot
	1051.4976	1051.6637	0.1661	158	746	754	DMALELTDK					Oxidation (M)[2]				Mascot
	1071.4854	1071.4399	-0.0455	-42	261	268	YRGDFEER									Mascot
	1083.5078	1083.4543	-0.0535	-49	364	371	DRYEAHHR									Mascot
	1113.6262	1113.5708	-0.0554	-50	12	21	VVVLAQEEAR									Mascot
	1143.6005	1143.5475	-0.053	-46	399	409	AIDLIDEAGAR									Mascot
	1215.6382	1215.5828	-0.0554	-46	704	712	HFRPEFLNR			20	0					Mascot
	1215.6382	1215.5828	-0.0554	-46	704	712	HFRPEFLNR									Mascot
	1269.7273	1269.6663	-0.061	-48	11	21	RVVVLAQEEAR									Mascot
	1293.7161	1293.6525	-0.0636	-49	210	222	NNPVLIGEPGVGK									Mascot
	1359.7413	1359.6835	-0.0578	-43	196	206	EIERVMQVLSR									Mascot
	1359.7478	1359.6835	-0.0643	-47	48	60	SLESLGISLEGVR			67	98.246					Mascot
	1461.806	1461.7352	-0.0708	-48	136	148	QQVIQLLSGYQGK									Mascot
	1550.8464	1550.7694	-0.077	-50	617	629	KPFSVVLFDEIEK									Mascot
	1563.8278	1563.7537	-0.0741	-47	544	559	RPSGSFIFAGPSGVGK			69	98.86					Mascot
	1563.8278	1563.7537	-0.0741	-47	544	559	RPSGSFIFAGPSGVGK									Mascot
	1592.8135	1592.7865	-0.027	-17	725	737	DEIIRMVDLMIGR					Oxidation (M)[6,10]				Mascot
	1666.8282	1666.7502	-0.078	-47	315	329	GELQTIGATTLDEYR									Mascot
	1691.8633	1691.7784	-0.0849	-50	372	388	VSITDSAMVAAATLADR									Mascot
	1706.9475	1706.8636	-0.0839	-49	616	629	RKPFSVVLFDEIEK									Mascot
	1794.9232	1794.8361	-0.0871	-49	315	330	GELQTIGATTLDEYRK									Mascot
	1985.0087	1984.9116	-0.0971	-49	630	646	AHQEIYNSLLQVLEDGR			124	100					Mascot
	1985.0087	1984.9116	-0.0971	-49	630	646	AHQEIYNSLLQVLEDGR									Mascot
	2112.0283	2111.929	-0.0993	-47	594	613	LFGAPPGYVGYEEGGQL									Mascot
							TEK									
	2161.1863	2161.0808	-0.1055	-49	223	243	TAVVEGLAQAIVHGEVPE									Mascot
							TLK									
	2164.1648	2164.0605	-0.1043	-48	342	360	FQPVQVGEPTVEHTIEILK									Mascot
	2320.2659	2320.1504	-0.1155	-50	341	360	RFQPVQVGEPTVEHTIEI									Mascot
							LK									
	2360.3083	2360.1919	-0.1164	-49	94	114	EALQLGHNYIGTEHILLGL									Mascot
							IR									
	2404.3081	2404.189	-0.1191	-50	223	245	TAVVEGLAQAIVHGEVPE									Mascot
							TLKDK									
	2476.2578	2476.1362	-0.1216	-49	61	83	SQVEEIIGQGQQAPSGHI									Mascot
							PFTPR									
	2476.2578	2476.1362	-0.1216	-49	61	83	SQVEEIIGQGQQAPSGHI			140	100					Mascot
							PFTPR									
	2723.2769	2723.1482	-0.1287	-47	565	588	ALANFLFGDDDALIQIDM									Mascot
							GEFHDR									
	2739.272	2739.1421	-0.1299	-47	565	588	ALANFLFGDDDALIQIDM					Oxidation (M)[18]				Mascot
							GEFHDR									
	2780.3518	2779.8145	-0.5373	-193	159	186	GGESGSPSTSLVLDQFG									Mascot
							RNLTAAAMEGK									
	2780.3518	2780.3076	-0.0442	-16	159	186	GGESGSPSTSLVLDQFG									Mascot
							RNLTAAAMEGK									
	2829.4714	2829.3354	-0.136	-48	22	47	MLNHNYIGTEHILLGLIHE									Mascot
							GEGVAAK									
4	ClpC [Mycobacterium avium subsp. paratuberculosis					gi|41406559		93222.9	5.59	30	470	100	396	100		
	K-10]															
	**Peptide Information**															
	**Calc. Mass**	**Obsrv. Mass**	**± da**	**± ppm**	**Start Seq.**	**End Seq.**	**Sequence**			**Ion Score**	**C. I. %**	**Modification**			**Rank**	**Result Type**
	812.3798	812.3414	-0.0384	-47	366	371	YEAHHR									Mascot
	829.5141	829.4765	-0.0376	-45	87	93	VLELSLR									Mascot
	832.4709	832.437	-0.0339	-41	200	206	VMQVLSR									Mascot
	900.4608	900.4171	-0.0437	-49	415	422	MTAPPDLR									Mascot
	996.4219	996.5475	0.1256	126	415	422	MTAPPDLR					Oxidation (M)[1], Phospho (ST)[2]				Mascot
	1071.4854	1071.4399	-0.0455	-42	261	268	YRGDFEER									Mascot
	1083.5078	1083.4543	-0.0535	-49	364	371	DRYEAHHR									Mascot
	1113.6262	1113.5708	-0.0554	-50	12	21	VVVLAQEEAR									Mascot
	1143.6005	1143.5475	-0.053	-46	399	409	AIDLIDEAGAR									Mascot
	1215.6382	1215.5828	-0.0554	-46	704	712	HFRPEFLNR			20	0					Mascot
	1215.6382	1215.5828	-0.0554	-46	704	712	HFRPEFLNR									Mascot
	1269.7273	1269.6663	-0.061	-48	11	21	RVVVLAQEEAR									Mascot
	1293.7161	1293.6525	-0.0636	-49	210	222	NNPVLIGEPGVGK									Mascot
	1359.7413	1359.6835	-0.0578	-43	196	206	EIERVMQVLSR									Mascot
	1359.7478	1359.6835	-0.0643	-47	48	60	SLESLGISLEGVR			67	98.246					Mascot
	1461.806	1461.7352	-0.0708	-48	136	148	QQVIQLLSGYQGK									Mascot
	1550.8464	1550.7694	-0.077	-50	617	629	KPFSVVLFDEIEK									Mascot
	1563.8278	1563.7537	-0.0741	-47	544	559	RPSGSFIFAGPSGVGK			69	98.86					Mascot
	1563.8278	1563.7537	-0.0741	-47	544	559	RPSGSFIFAGPSGVGK									Mascot
		1563.7537														Mascot
	1592.8643	1592.7865	-0.0778	-49	246	260	QLYTLDLGSLVAGSR									Mascot
	1666.8282	1666.7502	-0.078	-47	315	329	GELQTIGATTLDEYR									Mascot
	1691.8633	1691.7784	-0.0849	-50	372	388	VSITDSAMVAAATLADR									Mascot
	1706.9475	1706.8636	-0.0839	-49	616	629	RKPFSVVLFDEIEK									Mascot
	1794.9232	1794.8361	-0.0871	-49	315	330	GELQTIGATTLDEYRK									Mascot
	1985.0087	1984.9116	-0.0971	-49	630	646	AHQEIYNSLLQVLEDGR			124	100					Mascot
	1985.0087	1984.9116	-0.0971	-49	630	646	AHQEIYNSLLQVLEDGR									Mascot

	Calc. Mass	Obsrv. Mass	± da	± ppm	Start Seq.	End Seq.	Sequence	Ion Score	C. I. %	Modification	Rank	Result Type
	2112.0283	2111.929	-0.0993	-47	594	613	LFGAPPGYVGYEEGGQL					Mascot
							TEK					
	2164.1648	2164.0605	-0.1043	-48	342	360	FQPVQVGEPTVEHTIEILK					Mascot
	2320.1348	2320.1504	0.0156	7	223	243	TAVVEGLAQAIVHGQVPE			Phospho (ST)[1,19]		Mascot
							TLK					
	2360.3083	2360.1919	-0.1164	-49	94	114	EALQLGHNYIGTEHILLGL					Mascot
							IR					
	2476.2578	2476.1362	-0.1216	-49	61	83	SQVEEIIGQGQQAPSGHI					Mascot
							PFTPR					
	2476.2578	2476.1362	-0.1216	-49	61	83	SQVEEIIGQGQQAPSGHI	140	100			Mascot
							PFTPR					
	2723.2769	2723.1482	-0.1287	-47	565	588	ALANFLFGDDDALIQIDM					Mascot
							GEFHDR					
	2739.272	2739.1421	-0.1299	-47	565	588	ALANFLFGDDDALIQIDM			Oxidation (M)[18]		Mascot
							GEFHDR					
	2780.3518	2779.8145	-0.5373	-193	159	186	GGESGSPSTSLVLDQFG					Mascot
							RNLTAAAMEGK					
	2780.3518	2780.3076	-0.0442	-16	159	186	GGESGSPSTSLVLDQFG					Mascot
							RNLTAAAMEGK					
	2829.4714	2829.3354	-0.136	-48	22	47	MLNHNYIGTEHILLGLIHE					Mascot
							GEGVAAK					

Rank	Protein Name	Accession No.	Protein MW	Protein PI	Pep. Count	Protein Score	Protein Score C. I. %	Total Ion Score	Total Ion C. I. %
5	negative regulator of genetic competence ClpC/mecB [Mycobacterium avium 104]	gi|118466434	90715.7	5.58	28	460	100	396	100

Peptide Information

Calc. Mass	Obsrv. Mass	± da	± ppm	Start Seq.	End Seq.	Sequence	Ion Score	C. I. %	Modification	Rank	Result Type
812.3798	812.3414	-0.0384	-47	345	350	YEAHHR					Mascot
829.5141	829.4765	-0.0376	-45	66	72	VLELSLR					Mascot
832.4709	832.437	-0.0339	-41	179	185	VMQVLSR					Mascot
900.4608	900.4171	-0.0437	-49	394	401	MTAPPDLR					Mascot
996.4219	996.5475	0.1256	126	394	401	MTAPPDLR			Oxidation (M)[1], Phospho (ST)[2]		Mascot
1071.4854	1071.4399	-0.0455	-42	240	247	YRGDFEER					Mascot
1083.5078	1083.4543	-0.0535	-49	343	350	DRYEAHHR					Mascot
1143.6005	1143.5475	-0.053	-46	378	388	AIDLIDEAGAR					Mascot
1215.6382	1215.5828	-0.0554	-46	683	691	HFRPEFLNR	20	0			Mascot
1215.6382	1215.5828	-0.0554	-46	683	691	HFRPEFLNR					Mascot
1293.7161	1293.6525	-0.0636	-49	189	201	NNPVLIGEPGVGK					Mascot
	1293.6525										Mascot
1359.7413	1359.6835	-0.0578	-43	175	185	EIERVMQVLSR					Mascot
1359.7478	1359.6835	-0.0643	-47	27	39	SLESLGISLEGVR	67	98.48			Mascot
1461.806	1461.7352	-0.0708	-48	115	127	QQVIQLLSGYQGK					Mascot
1550.8464	1550.7694	-0.077	-50	596	608	KPFSVVLFDEIEK					Mascot
1563.8278	1563.7537	-0.0741	-47	523	538	RPSGSFIFAGPSVGK	69	99.012			Mascot
1563.8278	1563.7537	-0.0741	-47	523	538	RPSGSFIFAGPSVGK					Mascot
1592.8643	1592.7865	-0.0778	-49	225	239	QLYTLDLGSLVAGSR					Mascot
1666.8282	1666.7502	-0.078	-47	294	308	GELQTIGATTLDEYR					Mascot
1691.8633	1691.7784	-0.0849	-50	351	367	VSITDSAMVAAATLADR					Mascot
1706.9475	1706.8636	-0.0839	-49	595	608	RKPFSVVLFDEIEK					Mascot
1794.9232	1794.8361	-0.0871	-49	294	309	GELQTIGATTLDEYRK					Mascot
1985.0087	1984.9116	-0.0971	-49	609	625	AHQEIYNSLLQVLEDGR	124	100			Mascot
1985.0087	1984.9116	-0.0971	-49	609	625	AHQEIYNSLLQVLEDGR					Mascot
2112.0283	2111.929	-0.0993	-47	573	592	LFGAPPGYVGYEEGGQL					Mascot
						TEK					
2164.1648	2164.0605	-0.1043	-48	321	339	FQPVQVGEPTVEHTIEILK					Mascot
2320.1348	2320.1504	0.0156	7	202	222	TAVVEGLAQAIVHGQVPE			Phospho (ST)[1,19]		Mascot
						TLK					
2360.3083	2360.1919	-0.1164	-49	73	93	EALQLGHNYIGTEHILLGL					Mascot
						IR					
2476.2578	2476.1362	-0.1216	-49	40	62	SQVEEIIGQGQQAPSGHI					Mascot
						PFTPR					
2476.2578	2476.1362	-0.1216	-49	40	62	SQVEEIIGQGQQAPSGHI	140	100			Mascot
						PFTPR					
2723.2769	2723.1482	-0.1287	-47	544	567	ALANFLFGDDDALIQIDM					Mascot
						GEFHDR					
2739.272	2739.1421	-0.1299	-47	544	567	ALANFLFGDDDALIQIDM			Oxidation (M)[18]		Mascot
						GEFHDR					
2780.3518	2779.8145	-0.5373	-193	138	165	GGESGSPSTSLVLDQFG					Mascot
						RNLTAAAMEGK					
2780.3518	2780.3076	-0.0442	-16	138	165	GGESGSPSTSLVLDQFG					Mascot
						RNLTAAAMEGK					
2829.4714	2829.3354	-0.136	-48	1	26	MLNHNYIGTEHILLGLIHE					Mascot
						GEGVAAK					

**Gel Idx/Pos**	174/H1	**Instr./Gel Origin**	BA2060/121115A	**Process Status**	Analysis Succeeded
**Plate [#] Name**	[1] 31598	**Instrument Sample Name**		**Spectra**	8

Rank	Protein Name	Accession No.	Protein MW	Protein PI	Pep. Count	Protein Score	Protein Score C. I. %	Total Ion Score	Total Ion C. I. %
1	isocitrate dehydrogenase [Mycobacterium smegmatis str. MC2 155]	gi|118467432	82552.1	5.58	33	271	100	193	100

Peptide Information

Calc. Mass	Obsrv. Mass	± da	± ppm	Start Seq.	End Seq.	Sequence	Ion Score	C. I. %	Modification	Rank	Result Type
856.3812	856.4507	0.0695	81	635	642	ALDTATGK			Phospho (ST)[4]		Mascot
1053.4636	1053.3704	-0.0932	-88	180	188	TGDFYHGEK					Mascot
1099.5895	1099.4961	-0.0934	-85	466	474	DAPIRDWVK					Mascot
1107.5428	1107.4495	-0.0933	-84	377	386	AVNPESTFSR					Mascot
1107.5428	1107.4495	-0.0933	-84	377	386	AVNPESTFSR					Mascot
1118.6027	1118.4713	-0.1314	-117	248	257	TGVMFSLHVK					Mascot
1138.6942	1138.5974	-0.0968	-85	131	141	ILGSAVNPVLR	35	0			Mascot
1138.6942	1138.5974	-0.0968	-85	131	141	ILGSAVNPVLR					Mascot
1290.7317	1290.621	-0.1107	-86	263	274	VSHPIVFGHAVK					Mascot
1329.7485	1329.6324	-0.1161	-87	538	550	GKDTIAATGNILR					Mascot
1334.6376	1334.5201	-0.1175	-88	106	117	GYNVPDYPANPK					Mascot
1372.7228	1372.6071	-0.1157	-84	519	529	IQIMPQVWAMR					Mascot
1372.7228	1372.6071	-0.1157	-84	519	529	IQIMPQVWAMR	16	0			Mascot
1388.7178	1388.5994	-0.1184	-85	519	529	IQIMPQVWAMR			Oxidation (M)[4]		Mascot
1475.5404	1475.6351	0.0947	64	387	396	MYQEMINFCK			Carbamidomethyl (C)[9], Oxidation (M)[1,5], Phospho (Y)[2]		Mascot
1510.7874	1510.6597	-0.1277	-85	592	603	HVHQLVEENHLR					Mascot
1510.7874	1510.6597	-0.1277	-85	592	603	HVHQLVEENHLR	73	99.644			Mascot
1616.7479	1616.6095	-0.1384	-86	712	725	TVDIGGYYYPDPEK					Mascot
1634.984	1634.8411	-0.1429	-87	85	100	LPNISASVPQLLAAIK					Mascot
1637.7601	1637.6205	-0.1396	-85	159	172	KHPHSMGEWSQASR					Mascot
1653.755	1653.6143	-0.1407	-85	159	172	KHPHSMGEWSQASR			Oxidation (M)[6]		Mascot
1657.818	1657.6716	-0.1464	-88	50	63	ILAEFGDHLTEEQR					Mascot
1657.818	1657.6716	-0.1464	-88	50	63	ILAEFGDHLTEEQR	85	99.976			Mascot
1779.8265	1779.6713	-0.1552	-87	324	338	CHEHRPELAMVDSAK			Carbamidomethyl (C)[1]		Mascot
1796.9613	1796.8582	-0.1031	-57	131	147	ILGSAVNPVLREGNSDR					Mascot
1802.7714	1802.7313	-0.0401	-22	324	338	CHEHRPELAMVDSAK			Phospho (ST)[13]		Mascot
1896.0001	1895.8351	-0.165	-87	551	567	DYLTDLFPILELGTSAK					Mascot
1930.9796	1930.7876	-0.192	-99	232	247	KALIAFYEEQIEDAYK					Mascot
2015.0291	2014.8545	-0.1746	-87	693	711	ALAEQEEAIVSELNSVQG					Mascot
						K					
2165.1448	2164.9546	-0.1902	-88	64	84	VPDNLGELGALTQDPSA					Mascot
						NIIK					
2210.9893	2210.9138	-0.0755	-34	519	534	IQIMPQVWAMRYTLER			Oxidation (M)[4], Phospho (ST)[13], Phospho (Y)[12]		Mascot
2232.9883	2232.896	-0.0923	-41	604	623	WDSLGEFLALGASLEDM			Phospho (ST)[3]		Mascot
						GNK					

	2282.9053	2282.9839		0.0786	34	726	743	TSEVMRPSKTFNTTLESV					Oxidation (M)[5], Phospho (ST)[2,8],				Mascot	
													Phospho (ST)[1]					
	2284.1213	2283.981		-0.1403	-61	511	529	DHDTEGLKIQIMPQVWA					Oxidation (M)[12]				Mascot	
								MR										
	2344.1423	2343.9404		-0.2019	-86	339	360	GISNFHSPSDVIVDASMP									Mascot	
		2343.9404															Mascot	
								AMIR										
	2360.1372	2359.9363		-0.2009	-85	339	360	GISNFHSPSDVIVDASMP					Oxidation (M)[17]				Mascot	
								AMIR										
	2510.2861	2510.0698		-0.2163	-86	482	502	LSGMPVVFWLDTERPHE									Mascot	
								VELR										
	2872.1589	2872.1433		-0.0156	-5	712	734	TVDIGGYYYPDPEKTSEV					Phospho (Y)[7,8,9]				Mascot	
								MRPSK										
	2973.4265	2973.1682		-0.2583	-87	661	686	GSQFYLSLFWAQALAEQ									Mascot	
								TEDAELAER										
	3090.5742	3090.3069		-0.2673	-86	287	314	LFDELGVNVNNGLSDLY									Mascot	
								DKIEALPASQR										
	3091.6714	3091.3442		-0.3272	-106	2	29	SAQQPTIIYTLTDEAPLLA									Mascot	
								TYAFLPVVR										
	3687.791	3687.4727		-0.3183	-86	428	460	TFEIPEDGVADIVDIDTGE									Mascot	
								VLLTQNVEEGDIWR										
2	isocitrate dehydrogenase [Mycobacterium vanbaalenii					gi|120404190			82312.6	5.26	17	82	94.567	73	99.644			
	PYR-1]																	
	**Peptide Information**																	
	**Calc. Mass**	**Obsrv. Mass**		**± da**	**± ppm**	**Start**	**End**	**Sequence**			**Ion**	**C. I. %**	**Modification**			**Rank**	**Result Type**	
						**Seq.**	**Seq.**				**Score**							
	1045.4384	1045.4758		0.0374	36	189	196	SMTLDKDR					Phospho (ST)[1]				Mascot	
	1099.6293	1099.4961		-0.1332	-121	462	471	MPVVKDAAIR									Mascot	
	1107.5428	1107.4495		-0.0933	-84	378	387	AVNPESTFSR									Mascot	
	1107.5428	1107.4495		-0.0933	-84	378	387	AVNPESTFSR									Mascot	
	1118.6027	1118.4713		-0.1314	-117	249	258	TGVMFSLHVK									Mascot	
	1277.5388	1277.6024		0.0636	50	222	232	GDVIDSMYMSK					Oxidation (M)[7,9]				Mascot	
	1290.5857	1290.621		0.0353	27	388	397	MYQEVINFCK					Oxidation (M)[1]				Mascot	
	1510.7874	1510.6597		-0.1277	-85	593	604	HVHQLVEENHLR									Mascot	
	1510.7874	1510.6597		-0.1277	-85	593	604	HVHQLVEENHLR			73	99.644					Mascot	
	1634.984	1634.8411		-0.1429	-87	85	100	LPNISASVPQLLAAIK									Mascot	
	1637.7601	1637.6205		-0.1396	-85	159	172	KHPHSMGEWSQASR									Mascot	
	1653.755	1653.6143		-0.1407	-85	159	172	KHPHSMGEWSQASR					Oxidation (M)[6]				Mascot	
	1657.6437	1657.6716		0.0279	17	406	419	TMGTVPNVGLMAMK					Oxidation (M)[2,11,13], Phospho (ST)[1,4]				Mascot	
	1657.6437	1657.6716		0.0279	17	406	419	TMGTVPNVGLMAMK					Oxidation (M)[2,11,13], Phospho (ST)[1,4]				Mascot	
	1779.8265	1779.6713		-0.1552	-87	325	339	CHEHRPELAMVDSAK					Carbamidomethyl (C)[1]				Mascot	
	1802.7714	1802.7313		-0.0401	-22	325	339	CHEHRPELAMVDSAK					Phospho (ST)[13]				Mascot	
	1896.0001	1895.8351		-0.165	-87	552	568	DYLTDLFPILELGTSAK									Mascot	
	2165.1382	2164.9546		-0.1836	-85	85	103	LPNISASVPQLLAAIKELK					Phospho (ST)[5,7]				Mascot	
	2344.1423	2343.9404		-0.2019	-86	340	361	GISNFHSPSDVIVDASMP									Mascot	
								AMIR										
	2360.1372	2359.9363		-0.2009	-85	340	361	GISNFHSPSDVIVDASMP					Oxidation (M)[17]				Mascot	
								AMIR										
	2510.1179	2510.0698		-0.0481	-19	662	682	GSQFYLAMYWAQELAEQ					Oxidation (M)[8]				Mascot	
								TDDK										
	3090.6106	3090.3069	#	-0.3037	-98	288	315	LFDELGVNVNNGLSDLYS									Mascot	Mas
								KIEVLPASQR										
	3686.8071	3687.4727		0.6656	181	429	461	TFEIPEDGVANIVDIDTGE									Mascot	
								VLLTQNVEEGDIWR										
3	isocitrate dehydrogenase, NADP-dependent					gi|118463374			83086.2	5.82	11	73	58.784	73	99.644			
	[Mycobacterium avium 104]																	
	**Peptide Information**																	
	**Calc. Mass**	**Obsrv. Mass**		**± da**	**± ppm**	**Start**	**End**	**Sequence**			**Ion**	**C. I. %**	**Modification**			**Rank**	**Result Type**	
						**Seq.**	**Seq.**				**Score**							
	1045.4496	1045.4758		0.0262	25	189	196	SMTLDRAR					Oxidation (M)[2], Phospho (ST)[1]				Mascot	
	1107.5428	1107.4495		-0.0933	-84	377	386	AVNPESTFSR									Mascot	
	1107.5714	1107.4495		-0.1219	-110	197	205	NVKMELETK					Oxidation (M)[4]				Mascot	
	1109.421	1109.4098		-0.0112	-10	189	196	SMTLDRAR					Phospho (ST)[1], Phospho (ST)[3]				Mascot	
	1118.4799	1118.4713		-0.0086	-8	200	208	MELETKSGK					Oxidation (M)[1], Phospho (ST)[5]				Mascot	
	1290.7317	1290.621		-0.1107	-86	263	274	VSHPIVFGHAVK									Mascot	
	1510.7874	1510.6597		-0.1277	-85	592	603	HVHQLVEENHLR									Mascot	
	1510.7874	1510.6597		-0.1277	-85	592	603	HVHQLVEENHLR			73	99.644					Mascot	
	1616.5062	1616.6095		0.1033	64	220	231	TGDIIDSMFMSK					Oxidation (M)[8,10], Phospho (ST)[7,11],				Mascot	
													Phospho (ST)[1]					
	1707.7689	1707.6257		-0.1432	-84	128	141	YAKCLGSAVNPVLR					Carbamidomethyl (C)[4], Phospho (ST)[7],				Mascot	
													Phospho (Y)[1]					
	1896.0001	1895.8351		-0.165	-87	551	567	DYLTDLFPILELGTSAK									Mascot	
	2238.9783	2238.9409		-0.0374	-17	320	338	DLHACHEHRPELAMVDS					Phospho (ST)[17]				Mascot	
								AK										
	3091.4902	3091.3442		-0.146	-47	232	257	KALVEFYEQQMQDAYET									Mascot	
								GVMFSLHVK										
4	isocitrate dehydrogenase, NADP-dependent					gi|118465799			83549.2	5.7	10	73	58.784	73	99.644			
	[Mycobacterium avium 104]																	
	**Peptide Information**																	
	**Calc. Mass**	**Obsrv. Mass**		**± da**	**± ppm**	**Start**	**End**	**Sequence**			**Ion**	**C. I. %**	**Modification**			**Rank**	**Result Type**	
						**Seq.**	**Seq.**				**Score**							
	842.3655	842.4393		0.0738	88	635	642	SLDAATGK					Phospho (ST)[1]				Mascot	
	1045.4496	1045.4758		0.0262	25	189	196	SMTLDRAR					Oxidation (M)[2], Phospho (ST)[1]				Mascot	
	1109.421	1109.4098		-0.0112	-10	189	196	SMTLDRAR					Phospho (ST)[1], Phospho (ST)[3]				Mascot	
	1290.7317	1290.621		-0.1107	-86	263	274	VSHPIVFGHAVK									Mascot	
	1320.431	1320.4691		0.0381	29	377	386	AVSPESTFSR					Phospho (ST)[3,6], Phospho (ST)[7]				Mascot	
	1329.7122	1329.6324		-0.0798	-60	538	550	GQDTIAATGNILR									Mascot	
	1510.7874	1510.6597		-0.1277	-85	592	603	HVHQLVEENHLR									Mascot	
	1510.7874	1510.6597		-0.1277	-85	592	603	HVHQLVEENHLR			73	99.644					Mascot	
	1895.8279	1895.8351		0.0072	4	216	231	VSLRDGDVIDSMFMSK					Oxidation (M)[12], Phospho (ST)[2]				Mascot	
	2238.9783	2238.9409		-0.0374	-17	320	338	DLHACHEHRPELAMVDS					Phospho (ST)[17]				Mascot	
								AK										
	2283.1079	2282.9839		-0.124	-54	726	744	LTAVMRPSKTLNDALVTH					Phospho (ST)[8], Phospho (ST)[2]				Mascot	
								R										
	2297.0374	2296.9937		-0.0437	-19	160	179	HPHSMGEWSMASRTHV					Oxidation (M)[5]				Mascot	
								ATMK										
**Gel Idx/Pos**		175/H2						**Instr./Gel Origin**			BA2060/121115A			**Process Status**	Analysis Succeeded			
**Plate [#] Name**		[1] 31598						**Instrument Sample Name**						**Spectra**	8			
**Rank**	**Protein Name**					**Accession No.**			**Protein MW**	**Protein**	**Pep.**	**Protein**	**Protein**	**Total Ion**	**Total Ion**			
										**PI**	**Count**	**Score**	**Score**	**Score**	**C. I. %**			
													**C. I. %**					
1	ribonucleotide-diphosphate reductase subunit alpha					gi|118468034			80495.6	5.72	26	344	100	276	100			
	[Mycobacterium smegmatis str. MC2 155]																	
	**Peptide Information**																	
	**Calc. Mass**	**Obsrv. Mass**		**± da**	**± ppm**	**Start**	**End**	**Sequence**			**Ion**	**C. I. %**	**Modification**			**Rank**	**Result Type**	
						**Seq.**	**Seq.**				**Score**							
	828.4614	828.3935		-0.0679	-82	682	687	TLYYIR									Mascot	
	907.4784	907.4011		-0.0773	-85	671	677	AQIYAWR			26	0					Mascot	
	907.4784	907.4011		-0.0773	-85	671	677	AQIYAWR									Mascot	
	1009.4948	1009.4092		-0.0856	-85	67	74	EVLDQYSR									Mascot	
	1027.5612	1027.4716		-0.0896	-87	91	99	FPTFLGAFK									Mascot	
	1029.5575	1029.4779		-0.0796	-77	140	148	LVDEIIDGR									Mascot	
	1036.5786	1036.4456		-0.133	-128	79	87	SLLDRAYAK									Mascot	
	1107.5275	1107.4509		-0.0766	-69	91	99	FPTFLGAFK					Phospho (ST)[3]				Mascot	
	1161.4882	1161.3923		-0.0959	-83	323	331	YYEMVDDAR									Mascot	
	1165.5525	1165.4802		-0.0723	-62	529	537	YTDQVWEPK									Mascot	
	1165.5525	1165.6443		0.0918	79	529	537	YTDQVWEPK									Mascot	

	1169.7001	1169.6041	-0.096	-82	196	207	GGGVALLLSNIR			4	0					Mascot
	1169.7001	1169.6041	-0.096	-82	196	207	GGGVALLLSNIR									Mascot
	1177.483	1177.5038	0.0208	18	323	331	YYEMVDDAR					Oxidation (M)[4]				Mascot
	1179.531	1179.5062	-0.0248	-21	175	184	IEDNMESIGR					Oxidation (M)[5]				Mascot
		1179.5062														Mascot
	1407.7267	1407.597	-0.1297	-92	149	161	FQPATPTFLNSGK			31	0					Mascot
	1407.7267	1407.597	-0.1297	-92	149	161	FQPATPTFLNSGK									Mascot
	1461.7631	1461.6401	-0.123	-84	163	174	QRGEPVSCFLLR					Carbamidomethyl (C)[8]				Mascot
	1510.6396	1510.6488	0.0092	6	323	333	YYEMVDDARIR					Phospho (Y)[1]				Mascot
	1619.7588	1619.6206	-0.1382	-85	55	66	LDYLIENEYYER									Mascot
	1619.7588	1619.6206	-0.1382	-85	55	66	LDYLIENEYYER			42	0					Mascot
	1683.8336	1683.6906	-0.143	-85	229	243	LLEDSFSYANQLGAR			97	99.999					Mascot
	1683.8336	1683.6906	-0.143	-85	229	243	LLEDSFSYANQLGAR									Mascot
	1763.7999	1763.709	-0.0909	-52	229	243	LLEDSFSYANQLGAR					Phospho (Y)[8]				Mascot
	1763.8633	1763.709	-0.1543	-87	417	432	AMDSPDFAQTIEVAIR			63	95.618					Mascot
	1779.6914	1779.7051	0.0137	8	55	66	LDYLIENEYYER					Phospho (Y)[3,9]				Mascot
	1815.7632	1815.8851	0.1219	67	690	706	QMALEGTEVEGCVSCML					Oxidation (M)[2]				Mascot
	1884.9127	1884.7505	-0.1622	-86	544	559	LFADADIHIPTQDDWK									Mascot
	1904.9514	1904.7892	-0.1622	-85	244	260	QGAGAVYLHAHHPDIYR			47	0					Mascot
	1904.9514	1904.7892	-0.1622	-85	244	260	QGAGAVYLHAHHPDIYR									Mascot
	1905.8688	1905.762	-0.1068	-56	294	308	KNEDMYLFSPYDVER									Mascot
	1921.8636	1921.6995	-0.1641	-85	294	308	KNEDMYLFSPYDVER					Oxidation (M)[5]				Mascot
	2041.0138	2040.8396	-0.1742	-85	543	559	RLFADADIHIPTQDDWK									Mascot
	2210.0076	2209.8198	-0.1878	-85	520	537	YASGEFFDKYTDQVWEP									Mascot
							K									
	2366.9229	2367.0586	0.1357	57	91	107	FPTFLGAFKYYTSYTLK					Phospho (ST)[3], Phospho (Y)[10,11,14]				Mascot
	2509.1895	2508.9763	-0.2132	-85	35	54	QYFLQHVNQNTVFFHSQ									Mascot
							DEK									
	2552.3396	2552.1208	-0.2188	-86	638	660	IIDTYAAATQHVDQGLSLT									Mascot
							LFFK									
	3517.5129	3517.4856	-0.0273	-8	638	666	IIDTYAAATQHVDQGLSLT					Phospho (ST)[17], Phospho (ST)[4,9],				Mascot
							LFFKDTATTR					Phospho (Y)[5]				
2	ribonucleotide-diphosphate reductase subunit alpha					gi|54026278		124681.1	6.01	25	286	100	233	100		
	[Nocardia farcinica IFM 10152]															
	**Peptide Information**															
	**Calc. Mass**	**Obsrv. Mass**	**± da**	**± ppm**	**Start**	**End**	**Sequence**			**Ion**	**C. I. %**	**Modification**			**Rank**	**Result Type**
					**Seq.**	**Seq.**				**Score**						
	828.4614	828.3935	-0.0679	-82	1078	1083	TLYYIR									Mascot
	842.5457	842.4412	-0.1045	-124	467	474	LGIVSVVR									Mascot
	870.4832	870.4692	-0.014	-16	59	65	YSRAFVK									Mascot
	907.4784	907.4011	-0.0773	-85	1067	1073	AQIYAWR			26	0					Mascot
	907.4784	907.4011	-0.0773	-85	1067	1073	AQIYAWR									Mascot
	1027.5612	1027.4716	-0.0896	-87	78	86	FPTFLGAFK									Mascot
	1029.5575	1029.4779	-0.0796	-77	127	135	LVDEIIDGR									Mascot
	1045.4463	1045.4753	0.029	28	506	513	NLQNYTGR					Phospho (Y)[5]				Mascot
	1107.5275	1107.4509	-0.0766	-69	78	86	FPTFLGAFK					Phospho (ST)[3]				Mascot
	1169.7001	1169.6041	-0.096	-82	592	603	GGGVALLLSNIR			4	0					Mascot
	1169.7001	1169.6041	-0.096	-82	592	603	GGGVALLLSNIR									Mascot
	1177.6129	1177.5038	-0.1091	-93	1078	1085	TLYYIRLR					Phospho (Y)[3]				Mascot
	1179.5082	1179.5062	-0.002	-2	66	74	SLFQQAYDK					Phospho (Y)[7]				Mascot
	1222.3774	1222.5355	0.1581	129	719	726	YYEMVDDK					Phospho (Y)[1,2]				Mascot
	1234.5409	1234.5753	0.0344	28	719	727	YYEMVDDKR					Oxidation (M)[4]				Mascot
	1407.7267	1407.597	-0.1297	-92	136	148	FQPATPTFLNSGK			31	0					Mascot
	1407.7267	1407.597	-0.1297	-92	136	148	FQPATPTFLNSGK									Mascot
	1683.8336	1683.6906	-0.143	-85	625	639	LLEDSFSYANQLGAR			97	99.999					Mascot
	1683.8336	1683.6906	-0.143	-85	625	639	LLEDSFSYANQLGAR									Mascot
	1763.7999	1763.709	-0.0909	-52	625	639	LLEDSFSYANQLGAR					Phospho (Y)[8]				Mascot
	1763.8633	1763.709	-0.1543	-87	813	828	AMDSPDFAQTIEVAIR			63	95.618					Mascot
	1779.8582	1779.7051	-0.1531	-86	813	828	AMDSPDFAQTIEVAIR					Oxidation (M)[2]				Mascot
	1815.7632	1815.8851	0.1219	67	1086	1102	QMALEGTEVEGCVSCML					Oxidation (M)[2]				Mascot
	1884.8488	1884.7505	-0.0983	-52	178	194	AGDRVLSHDGSYATVEK					Phospho (Y)[12]				Mascot
	1904.9514	1904.7892	-0.1622	-85	640	656	QGAGAVYLHAHHPDIYR			47	0					Mascot
	1904.9514	1904.7892	-0.1622	-85	640	656	QGAGAVYLHAHHPDIYR									Mascot
	1905.8688	1905.762	-0.1068	-56	690	704	KNEDMYLFSPYDVER									Mascot
	1921.8636	1921.6995	-0.1641	-85	690	704	KNEDMYLFSPYDVER					Oxidation (M)[5]				Mascot
	2067.9648	2067.9622	-0.0026	-1	939	955	QLFADAGVRIPTQDDWR					Phospho (ST)[12]				Mascot
	2238.9841	2238.9404	-0.0437	-20	42	58	LDYLVEENYYEPEVLDR					Phospho (Y)[3]				Mascot
	2366.9229	2367.0586	0.1357	57	78	94	FPTFLGAFKYYTSYTLK					Phospho (ST)[3], Phospho (Y)[10,11,14]				Mascot
	2509.2441	2508.9763	-0.2678	-107	392	414	MVCNSKIVASLLLSLAGT					Carbamidomethyl (C)[3], Oxidation (M)[1],				Mascot
							GYSTK					Phospho (Y)[20]				
	2552.3396	2552.1208	-0.2188	-86	1034	1056	IIDTYAAATQHVDQGLSLT									Mascot
							LFFK									
	3517.5078	3517.4856	-0.0222	-6	544	570	QVYNLHVEGTHTYTIRGA					Carbamidomethyl (C)[23], Phospho				Mascot
							VVHNCFLLR					(ST)[10,12], Phospho (Y)[3,13]				
3	ribonucleotide-diphosphate reductase subunit alpha					gi|108798785		79307.2	6.12	19	277	100	233	100		
	[Mycobacterium sp. MCS]															
	**Peptide Information**															
	**Calc. Mass**	**Obsrv. Mass**	**± da**	**± ppm**	**Start**	**End**	**Sequence**			**Ion**	**C. I. %**	**Modification**			**Rank**	**Result Type**
					**Seq.**	**Seq.**				**Score**						
	828.4614	828.3935	-0.0679	-82	669	674	TLYYIR									Mascot
	907.4784	907.4011	-0.0773	-85	658	664	AQIYAWR			26	0					Mascot
	907.4784	907.4011	-0.0773	-85	658	664	AQIYAWR									Mascot
	1009.4948	1009.4092	-0.0856	-85	54	61	EVLDQYSR									Mascot
		1009.4092														Mascot
	1027.5612	1027.4716	-0.0896	-87	78	86	FPTFLGAFK									Mascot
	1029.5575	1029.4779	-0.0796	-77	127	135	LVDEIIDGR									Mascot
	1107.5275	1107.4509	-0.0766	-69	78	86	FPTFLGAFK					Phospho (ST)[3]				Mascot
	1169.7001	1169.6041	-0.096	-82	183	194	GGGVALLLSNIR			4	0					Mascot
	1169.7001	1169.6041	-0.096	-82	183	194	GGGVALLLSNIR									Mascot
	1177.483	1177.5038	0.0208	18	310	318	YYEMVDDSR									Mascot
	1179.531	1179.5062	-0.0248	-21	162	171	IEDNMESIGR					Oxidation (M)[5]				Mascot
	1407.7267	1407.597	-0.1297	-92	136	148	FQPATPTFLNSGK			31	0					Mascot
	1407.7267	1407.597	-0.1297	-92	136	148	FQPATPTFLNSGK									Mascot
	1461.7631	1461.6401	-0.123	-84	150	161	QRGEPVSCFLLR					Carbamidomethyl (C)[8]				Mascot
	1683.8336	1683.6906	-0.143	-85	216	230	LLEDSFSYANQLGAR			97	99.999					Mascot
	1683.8336	1683.6906	-0.143	-85	216	230	LLEDSFSYANQLGAR									Mascot
	1763.7999	1763.709	-0.0909	-52	216	230	LLEDSFSYANQLGAR					Phospho (Y)[8]				Mascot
	1763.8633	1763.709	-0.1543	-87	404	419	AMDSPDFAQTIEVAIR			63	95.618					Mascot
	1779.8582	1779.7051	-0.1531	-86	404	419	AMDSPDFAQTIEVAIR					Oxidation (M)[2]				Mascot
	1815.7632	1815.8851	0.1219	67	677	693	QMALEGTEVEGCVSCML					Oxidation (M)[2]				Mascot
	1904.9514	1904.7892	-0.1622	-85	231	247	QGAGAVYLHAHHPDIYR			47	0					Mascot
	1904.9514	1904.7892	-0.1622	-85	231	247	QGAGAVYLHAHHPDIYR									Mascot
	1905.8688	1905.762	-0.1068	-56	281	295	KNEDMYLFSPYDVER									Mascot
	1921.8636	1921.6995	-0.1641	-85	281	295	KNEDMYLFSPYDVER					Oxidation (M)[5]				Mascot
	2366.9229	2367.0586	0.1357	57	78	94	FPTFLGAFKYYTSYTLK					Phospho (ST)[3], Phospho (Y)[10,11,14]				Mascot
	2509.2734	2508.9763	-0.2971	-118	231	252	QGAGAVYLHAHHPDIYR									Mascot
							FLDTK									
	2552.3396	2552.1208	-0.2188	-86	625	647	IIDTYAAATQHVDQGLSLT									Mascot
							LFFK									
	3517.5129	3517.4856	-0.0273	-8	625	653	IIDTYAAATQHVDQGLSLT					Phospho (ST)[17], Phospho (ST)[4,9],				Mascot
							LFFKDTATTR					Phospho (Y)[5]				
4	ribonucleotide-diphosphate reductase subunit alpha					gi|120403036		82227.5	5.87	20	268	100	227	100		
	[Mycobacterium vanbaalenii PYR-1]															
	**Peptide Information**															
	**Calc. Mass**	**Obsrv. Mass**	**± da**	**± ppm**	**Start**	**End**	**Sequence**			**Ion**	**C. I. %**	**Modification**			**Rank**	**Result Type**
					**Seq.**	**Seq.**				**Score**						
	828.4614	828.3935	-0.0679	-82	698	703	TLYYIR									Mascot
	907.4784	907.4011	-0.0773	-85	687	693	AQIYAWR			26	0					Mascot
	907.4784	907.4011	-0.0773	-85	687	693	AQIYAWR									Mascot
	1009.4948	1009.4092	-0.0856	-85	83	90	EVLDQYSR									Mascot
	1029.58	1029.4779	-0.1021	-99	516	524	ASNRIAIER									Mascot
	1161.4882	1161.3923	-0.0959	-83	339	347	YYEMVDDAR									Mascot

	1169.4275	1169.6041	0.1766	151	83	90	EVLDQYSR				Phospho (ST)[7], Phospho (Y)[6]					Mascot
	1169.4275	1169.6041	0.1766	151	83	90	EVLDQYSR				Phospho (ST)[7], Phospho (Y)[6]					Mascot
	1177.483	1177.5038	0.0208	18	339	347	YYEMVDDAR				Oxidation (M)[4]					Mascot
	1179.531	1179.5062	-0.0248	-21	191	200	IEDNMESIGR				Oxidation (M)[5]					Mascot
	1263.682	1263.5878	-0.0942	-75	212	223	GGGVALLLTNIR				Phospho (ST)[9]					Mascot
	1407.7267	1407.597	-0.1297	-92	165	177	FQPATPTFLNSGK		31	0						Mascot
	1407.7267	1407.597	-0.1297	-92	165	177	FQPATPTFLNSGK									Mascot
	1461.7631	1461.6401	-0.123	-84	179	190	QRGEPVSCFLLR				Carbamidomethyl (C)[8]					Mascot
	1510.6396	1510.6488	0.0092	6	339	349	YYEMVDDARIR				Phospho (Y)[1]					Mascot
	1683.6703	1683.6906	0.0203	12	545	556	YTEQVWEPKTDK				Phospho (ST)[2], Phospho (Y)[1]					Mascot
	1683.8336	1683.6906	-0.143	-85	245	259	LLEDSFSYANQLGAR		97	99.999						Mascot
	1763.6366	1763.709	0.0724	41	545	556	YTEQVWEPKTDK				Phospho (ST)[2,10], Phospho (Y)[1]					Mascot
	1763.8633	1763.709	-0.1543	-87	433	448	AMDSPDFAQTIEVAIR		63	95.618						Mascot
	1779.8582	1779.7051	-0.1531	-86	433	448	AMDSPDFAQTIEVAIR				Oxidation (M)[2]					Mascot
	1816.0466	1815.8851	-0.1615	-89	293	309	TLSLGVVIPDITFELAK									Mascot
	1887.7955	1887.7633	-0.0322	-17	706	722	QMALEGTQVENCVSCML				Oxidation (M)[2,16]					Mascot
	1904.9514	1904.7892	-0.1622	-85	260	276	QGAGAVYLHAHHPDIYR		47	0						Mascot
	1904.9514	1904.7892	-0.1622	-85	260	276	QGAGAVYLHAHHPDIYR									Mascot
	1905.8688	1905.762	-0.1068	-56	310	324	KNEDMYLFSPYDVER									Mascot
	1921.8636	1921.6995	-0.1641	-85	310	324	KNEDMYLFSPYDVER				Oxidation (M)[5]					Mascot
	2239.0337	2238.9404	-0.0933	-42	704	722	LRQMALEGTQVENCVSC				Carbamidomethyl (C)[14,17]					Mascot
							ML									
	2509.2734	2508.9763	-0.2971	-118	260	281	QGAGAVYLHAHHPDIYR									Mascot
							FLDTK									
	3337.4463	3337.4795	0.0332	10	45	70	DVQAAREYFLQHVNQNT				Phospho (ST)[17], Phospho (Y)[8]					Mascot
							VFFHNQDEK									
**Gel Idx/Pos**		176/H3					**Instr./Gel Origin**		BA2060/121115A				**Process Status**	Analysis Succeeded		
**Plate [#] Name**		[1] 31598					**Instrument Sample Name**						**Spectra**	8		
**Rank**	**Protein Name**					**Accession No.**	**Protein MW**	**Protein PI**	**Pep. Count**	**Protein Score**	**Protein Score C. I. %**	**Total Ion Score**	**Total Ion C. I. %**			
1	metallopeptidase [Mycobacterium smegmatis str. MC2 155]					gi|118467687	74084.1	4.82	34	718	100	615	100			
	**Peptide Information**															
	**Calc. Mass**	**Obsrv. Mass**	**± da**	**± ppm**	**Start Seq.**	**End Seq.**	**Sequence**		**Ion Score**	**C. I. %**	**Modification**				**Rank**	**Result Type**
	923.4581	923.3793	-0.0788	-85	429	436	GYIVNSDR									Mascot
	1004.537	1004.4501	-0.0869	-87	320	328	TLSGTEQIR									Mascot
	1015.4479	1015.3598	-0.0881	-87	420	427	DDLYGNYR									Mascot
	1060.5455	1060.4547	-0.0908	-86	363	371	MDELVANLR									Mascot
	1080.532	1080.4399	-0.0921	-85	227	235	DADLTYNLR									Mascot
	1109.6102	1109.5143	-0.0959	-86	215	224	IAAAHWDVVK									Mascot
	1171.5491	1171.4482	-0.1009	-86	420	428	DDLYGNYRR									Mascot
	1209.6222	1209.5188	-0.1034	-85	16	26	SGIDLTHVDPR									Mascot
	1236.6332	1236.5618	-0.0714	-58	227	236	DADLTYNLRR									Mascot
		1236.5618														Mascot
	1295.6683	1295.5608	-0.1075	-83	599	608	VFFGWAQVWR		58	87.816						Mascot
	1295.6683	1295.5608	-0.1075	-83	599	608	VFFGWAQVWR									Mascot
	1309.5741	1309.52	-0.0541	-41	399	408	FTPKIGYPAR				Phospho (ST)[2], Phospho (Y)[7]					Mascot
	1354.7325	1354.6163	-0.1162	-86	586	598	EAPVIDGLTGVQR									Mascot
	1364.6957	1364.5779	-0.1178	-86	620	631	LAVDPHSPPEFR		52	44.691						Mascot
	1364.6957	1364.5779	-0.1178	-86	620	631	LAVDPHSPPEFR									Mascot
	1392.6794	1392.563	-0.1164	-84	41	51	WLTEYEIPADR									Mascot
	1392.6794	1392.563	-0.1164	-84	41	51	WLTEYEIPADR		71	99.417						Mascot
	1494.7587	1494.6333	-0.1254	-84	536	547	ALIEQYEQFTPR									Mascot
	1494.7587	1494.6333	-0.1254	-84	536	547	ALIEQYEQFTPR		90	99.993						Mascot
	1502.7883	1502.6533	-0.135	-90	334	348	AVSVVENLMGDALGK									Mascot
	1515.5641	1515.5378	-0.0263	-17	320	330	TLSGTEQIRDR				Phospho (ST)[3], Phospho (ST)[1,5]					Mascot
	1611.7986	1611.6589	-0.1397	-87	27	40	TRPQDDLFGHVNGR									Mascot
	1658.8894	1658.7383	-0.1511	-91	333	348	RAVSVVENLMGDALGK									Mascot
	1691.7649	1691.6487	-0.1162	-69	27	40	TRPQDDLFGHVNGR				Phospho (ST)[1]					Mascot
	1691.7946	1691.6487	-0.1459	-86	376	389	VSIDELDWMTPETR		110	100						Mascot
	1707.7894	1707.6409	-0.1485	-87	376	389	VSIDELDWMTPETR				Oxidation (M)[9]					Mascot
	1724.806	1724.6562	-0.1498	-87	192	207	MFGLIYGGDHTDTAAR									Mascot
	1740.801	1740.646	-0.155	-89	192	207	MFGLIYGGDHTDTAAR				Oxidation (M)[1]					Mascot
	1747.6523	1747.6569	0.0046	3	1	15	MTVQATRGTADATGK				Phospho (ST)[2,6,9]					Mascot
	1763.6472	1763.6487	0.0015	1	1	15	MTVQATRGTADATGK				Oxidation (M)[1], Phospho (ST)[2,6,9]					Mascot
	1890.8676	1890.7026	-0.165	-87	70	88	DLITEAAASGAADGTDEQ									Mascot
							R									
	1897.7987	1897.6338	-0.1649	-87	512	527	YDGDGNLVDWWTDADR									Mascot
	1897.7987	1897.6338	-0.1649	-87	512	527	YDGDGNLVDWWTDADR		73	99.597						Mascot
	1930.9215	1930.7526	-0.1689	-87	89	105	IGDLYASFMDTQTIAER									Mascot
	1946.9164	1946.743	-0.1734	-89	89	105	IGDLYASFMDTQTIAER				Oxidation (M)[9]					Mascot
	1972.9651	1972.7936	-0.1715	-87	302	319	AGLLTDDLVAEDFAFYGR									Mascot
	1972.9651	1972.7936	-0.1715	-87	302	319	AGLLTDDLVAEDFAFYGR		166	100						Mascot
	2010.8878	2010.7128	-0.175	-87	89	105	IGDLYASFMDTQTIAER				Phospho (Y)[5]					Mascot
	2183.0591	2182.8665	-0.1926	-88	156	174	YLLHMGQSGIGLPDESYF									Mascot
							R									
	2199.054	2198.8618	-0.1922	-87	156	174	YLLHMGQSGIGLPDESYF				Oxidation (M)[5]					Mascot
							R									
	2211.0386	2210.9106	-0.128	-58	372	389	EAYRVSIDELDWMTPET									Mascot
							R									
	2617.2336	2617.0022	-0.2314	-88	64	88	AEEQIRDLITEAAASGAA									Mascot
							DGTDEQR									
	2820.2522	2820.0054	-0.2468	-88	638	660	NIDDFYEAFDVTENDELF									Mascot
							LEPER									
	2976.3533	2976.0945	-0.2588	-87	638	661	NIDDFYEAFDVTENDELF									Mascot
		2976.0945														Mascot
							LEPERR									
	2999.6411	2999.373	-0.2681	-89	106	135	GLQPLLDELALIDAAPDA									Mascot
							DALAAVLGGLQR									
	3235.6746	3235.3938	-0.2808	-87	548	579	GLDPSHHVNGAFTVGENI									Mascot
							GDLGGLSIALLAYK									
	3501.7324	3501.4163	-0.3161	-90	237	269	FADLPAEAPGFDWSGWV									Mascot
							QGLGTTPDTVAELVVR									
**Gel Idx/Pos**		177/H4					**Instr./Gel Origin**		BA2060/121115A				**Process Status**	Analysis Succeeded		
**Plate [#] Name**		[1] 31598					**Instrument Sample Name**						**Spectra**	8		
**Rank**	**Protein Name**					**Accession No.**	**Protein MW**	**Protein PI**	**Pep. Count**	**Protein Score**	**Protein Score C. I. %**	**Total Ion Score**	**Total Ion C. I. %**			
1	1-pyrroline-5-carboxylate dehydrogenase [Mycobacterium smegmatis str. MC2 155]					gi|118469637	58397.3	5.49	24	700	100	615	100			
	**Peptide Information**															
	**Calc. Mass**	**Obsrv. Mass**	**± da**	**± ppm**	**Start Seq.**	**End Seq.**	**Sequence**		**Ion Score**	**C. I. %**	**Modification**				**Rank**	**Result Type**
	824.4413	824.3768	-0.0645	-78	151	157	FNVAFAR									Mascot
	836.4049	836.3415	-0.0634	-76	284	289	YHTYPR									Mascot
	873.4424	873.3712	-0.0712	-82	276	283	EVGANIDR									Mascot
	947.4727	947.3994	-0.0733	-77	510	518	AGSAQNLMR									Mascot
	963.4676	963.4557	-0.0119	-12	510	518	AGSAQNLMR				Oxidation (M)[8]					Mascot
	996.3376	996.5126	0.175	176	284	289	YHTYPR				Phospho (Y)[1,4]					Mascot
	1013.4686	1013.3864	-0.0822	-81	318	326	GAFDYQGQK									Mascot
	1085.5739	1085.4886	-0.0853	-79	110	119	AADLLSGPWR		54	64.089						Mascot
	1085.5739	1085.4886	-0.0853	-79	110	119	AADLLSGPWR									Mascot
	1165.5402	1165.6447	0.1045	90	110	119	AADLLSGPWR				Phospho (ST)[6]					Mascot
	1171.6429	1171.5515	-0.0914	-78	466	476	TAVQQALDGLR		26	0						Mascot
	1171.6429	1171.5515	-0.0914	-78	466	476	TAVQQALDGLR									Mascot
	1250.6376	1250.5396	-0.098	-78	454	465	YGLTGAVIADDR									Mascot
	1346.6488	1346.54	-0.1088	-81	93	103	QDWAALPFDQR		90	99.992						Mascot
	1346.6488	1346.54	-0.1088	-81	93	103	QDWAALPFDQR									Mascot
	1425.6678	1425.5527	-0.1151	-81	343	355	MGDDFLSATEALR									Mascot

	1425.6678	1425.5527	-0.1151	-81	343	355	MGDDFLSATEALR		57	82.613					Mascot	
	1441.6627	1441.5455	-0.1172	-81	343	355	MGDDFLSATEALR				Oxidation (M)[1]				Mascot	
	1663.8551	1663.7189	-0.1362	-82	298	312	DFVLAHSSAHPDVLR								Mascot	
	1793.9657	1793.8157	-0.15	-84	158	173	QILAQQPISSPGVWNR								Mascot	
	1793.9657	1793.8157	-0.15	-84	158	173	QILAQQPISSPGVWNR		118	100					Mascot	
	1870.8429	1870.6825	-0.1604	-86	527	542	ETFVPPTNHTYPHMEA								Mascot	
	1886.8378	1886.678	-0.1598	-85	527	542	ETFVPPTNHTYPHMEA				Oxidation (M)[14]				Mascot	
	1913.8876	1913.7261	-0.1615	-84	356	373	YGDVADLTNYGGAVIDD								Mascot	
							R									
	1950.8092	1950.6316	-0.1776	-91	527	542	ETFVPPTNHTYPHMEA				Phospho (Y)[11]				Mascot	
		1950.6316													Mascot	
	1993.8539	1993.8054	-0.0485	-24	356	373	YGDVADLTNYGGAVIDD				Phospho (Y)[1]				Mascot	
							R									
	2043.0559	2042.8778	-0.1781	-87	258	275	LAGIHFTGSTATFQHLWR								Mascot	
	2184.0066	2183.813	-0.1936	-89	133	150	SAYQAEIDAPCELIDFWR				Carbamidomethyl (C)[11]				Mascot	
	2184.0066	2183.813	-0.1936	-89	133	150	SAYQAEIDAPCELIDFWR		150	100	Carbamidomethyl (C)[11]				Mascot	
	2209.1458	2208.9548	-0.191	-86	70	92	LGTLTNAEHADASAAIEA								Mascot	
							AIAAK									
	2282.9548	2282.9783	0.0235	10	258	275	LAGIHFTGSTATFQHLWR				Phospho (ST)[9], Phospho (ST)[7,10]				Mascot	
	2298.1755	2297.9814	-0.1941	-84	298	317	DFVLAHSSAHPDVLRTAL				Phospho (ST)[7]				Mascot	
							IR									
	2405.2571	2405.0427	-0.2144	-89	290	312	LVGETGGKDFVLAHSSA								Mascot	
							HPDVLR									
	2603.3689	2603.1404	-0.2285	-88	28	51	LTTALHDLASTHIDLPHVI								Mascot	
							GGEHR									
	2714.332	2714.0879	-0.2441	-90	477	502	YAAGNFYINDKPTGAVVG		146	100					Mascot	
							QQPFGGSR									
	2714.332	2714.0879	-0.2441	-90	477	502	YAAGNFYINDKPTGAVVG								Mascot	
							QQPFGGSR									
	2724.2571	2724.0105	-0.2466	-91	1	25	MDAITDVPTPANEPIHDY								Mascot	
							APGSQER									
	2740.252	2740.0168	-0.2352	-86	1	25	MDAITDVPTPANEPIHDY				Oxidation (M)[1]				Mascot	
							APGSQER									
	3337.5269	3337.4487	-0.0782	-23	122	150	VAAATMLGQSKSAYQAEI				Carbamidomethyl (C)[22], Oxidation (M)[6],				Mascot	
							DAPCELIDFWR				Phospho (Y)[14]					
	3338.55	3338.4436	-0.1064	-32	28	57	LTTALHDLASTHIDLPHVI				Oxidation (M)[25], Phospho (ST)[2,3]				Mascot	
							GGEHRMGSGAR									
2	delta-1-pyrroline-5-carboxylate dehydrogenase					gi|117928957	59190.1	5.56	10	155	100	146	100			
	[Acidothermus cellulolyticus 11B]															
	**Peptide Information**															
	**Calc. Mass**	**Obsrv. Mass**	**± da**	**± ppm**	**Start**	**End**	**Sequence**		**Ion**	**C. I. %**	**Modification**			**Rank**	**Result Type**	
					**Seq.**	**Seq.**			**Score**							
	824.2739	824.3768	0.1029	125	520	524	WTTTR				Phospho (ST)[2,3]				Mascot	
	873.5152	873.3712	-0.144	-165	33	40	LKDLAASR								Mascot	
	947.4039	947.3994	-0.0045	-5	538	544	YPHMEDR								Mascot	
	963.3989	963.4557	0.0568	59	538	544	YPHMEDR				Oxidation (M)[4]				Mascot	
	1013.4485	1013.3864	-0.0621	-61	468	475	AITLACER				Carbamidomethyl (C)[6], Phospho (ST)[3]				Mascot	
	1250.6232	1250.5396	-0.0836	-67	121	133	AAMAAATMLGQSK								Mascot	
	1346.5844	1346.54	-0.0444	-33	121	133	AAMAAATMLGQSK		8	0	Oxidation (M)[3], Phospho (ST)[12]				Mascot	
	1346.5844	1346.54	-0.0444	-33	121	133	AAMAAATMLGQSK				Oxidation (M)[3], Phospho (ST)[7]				Mascot	
	1776.7354	1776.7889	0.0535	30	13	26	NEPVIDYRPGTAER				Phospho (ST)[11], Phospho (Y)[7]				Mascot	
	1870.6632	1870.6825	0.0193	10	319	333	GAFEYQGQKCSAVSR				Phospho (ST)[11,14], Phospho (Y)[5]				Mascot	
	1908.7694	1908.6544	-0.115	-60	160	174	ILADQPLSTPGTWNR				Phospho (ST)[8], Phospho (ST)[9,12]				Mascot	
	2261.0002	2260.835	-0.1652	-73	134	151	TCYQAEIDAICELADFWR				Carbamidomethyl (C)[2,11]				Mascot	
	2283.9451	2283.9736	0.0285	12	134	151	TCYQAEIDAICELADFWR				Carbamidomethyl (C)[2], Phospho (Y)[3]				Mascot	
	2714.332	2714.0879 #	-0.2441	-90	478	503	YAAGNFYINDKPTGAVVG		146	100					Mascot	Mas
							QQPFGGSR									
	2714.332	2714.0879	-0.2441	-90	478	503	YAAGNFYINDKPTGAVVG								Mascot	
							QQPFGGSR									
3	delta-1-pyrroline-5-carboxylate dehydrogenase					gi|152968421	58115	5.24	7	96	99.793	96	99.998			
	[Kineococcus radiotolerans SRS30216]															
	**Peptide Information**															
	**Calc. Mass**	**Obsrv. Mass**	**± da**	**± ppm**	**Start**	**End**	**Sequence**		**Ion**	**C. I. %**	**Modification**			**Rank**	**Result Type**	
					**Seq.**	**Seq.**			**Score**							
	1250.5251	1250.5396	0.0145	12	122	132	IAAATMLGQSK				Phospho (ST)[10], Phospho (ST)[5]				Mascot	
	1329.6311	1329.5137	-0.1174	-88	374	384	SYARNVAAIER				Phospho (Y)[2]				Mascot	
	2260.9216	2260.835	-0.0866	-38	83	103	AAADAALAAAPEWAAMD				Oxidation (M)[16], Phospho (Y)[18]				Mascot	
							YDDR									
	2603.344	2603.1404	-0.2036	-78	26	49	ERLLAALAEQTANPVELT				Phospho (ST)[11]				Mascot	
							QTIGGK									
	2714.332	2714.0879	-0.2441	-90	480	505	FTAGNFYVNDKPTGAVV		96	99.998					Mascot	
							GQQPFGGSR									
	2714.332	2714.0879	-0.2441	-90	480	505	FTAGNFYVNDKPTGAVV								Mascot	
							GQQPFGGSR									
	2779.9731	2779.6926	-0.2805	-101	351	373	VDSLSYGDVSDLSHFGG				Phospho (ST)[3,5,10,13], Phospho (Y)[6]				Mascot	
							AVIDAR									
	2779.9731	2780.1946	0.2215	80	351	373	VDSLSYGDVSDLSHFGG				Phospho (ST)[3,5,10,13], Phospho (Y)[6]				Mascot	
							AVIDAR									
	3337.1731	3337.4487	0.2756	83	351	377	VDSLSYGDVSDLSHFGG				Phospho (ST)[3,5,10,13], Phospho (Y)[6,25]				Mascot	
							AVIDARSYAR									
4	1-pyrroline-5-carboxylate dehydrogenase					gi|118462320	59760.2	5.82	7	77	84.33	77	99.849			
	[Mycobacterium avium 104]															
	**Peptide Information**															
	**Calc. Mass**	**Obsrv. Mass**	**± da**	**± ppm**	**Start**	**End**	**Sequence**		**Ion**	**C. I. %**	**Modification**			**Rank**	**Result Type**	
					**Seq.**	**Seq.**			**Score**							
	824.4413	824.3768	-0.0645	-78	163	169	FNVAFAR								Mascot	
	963.537	963.4557	-0.0813	-84	70	77	IDVVQPHR								Mascot	
	1013.4686	1013.3864	-0.0822	-81	330	338	GAFDYQGQK								Mascot	
	1250.5251	1250.5396	0.0145	12	134	144	IAAATMLGQSK				Phospho (ST)[10], Phospho (ST)[5]				Mascot	
	1793.9293	1793.8157	-0.1136	-63	170	185	QILAQQPISGPGEWNR								Mascot	
	1793.9293	1793.8157	-0.1136	-63	170	185	QILAQQPISGPGEWNR		77	99.849					Mascot	
	1892.7316	1892.6631	-0.0685	-36	339	353	CSAASRAFIAQSVWR				Phospho (ST)[2,5,12]				Mascot	
	2208.6824	2208.9548	0.2724	123	539	555	ETFVPATDHTYPHMGAD				Phospho (ST)[2,7,10], Phospho (Y)[11]				Mascot	
	2224.6772	2224.9243	0.2471	111	539	555	ETFVPATDHTYPHMGAD				Oxidation (M)[14], Phospho (ST)[2,7,10],				Mascot	
											Phospho (Y)[11]					
**Gel Idx/Pos**		178/H5					**Instr./Gel Origin**		BA2060/121115A				**Process Status**	Analysis Succeeded		
**Plate [#] Name**		[1] 31598					**Instrument Sample Name**						**Spectra**	8		
**Rank**	**Protein Name**					**Accession No.**	**Protein MW**	**Protein PI**	**Pep. Count**	**Protein Score**	**Protein Score C. I. %**	**Total Ion Score**	**Total Ion C. I. %**			
1	ATP-dependent rna helicase, dead/deah box family protein [Mycobacterium smegmatis str. MC2 155]					gi|118470234	63684.6	7.29	20	252	100	217	100			
	**Peptide Information**															
	**Calc. Mass**	**Obsrv. Mass**	**± da**	**± ppm**	**Start**	**End**	**Sequence**		**Ion**	**C. I. %**	**Modification**			**Rank**	**Result Type**	
					**Seq.**	**Seq.**			**Score**							
	850.4451	850.4596	0.0145	17	247	253	KMDALTR				Oxidation (M)[2]				Mascot	
	887.5097	887.4251	-0.0846	-95	239	246	FIQVAGPR								Mascot	
	937.4625	937.38	-0.0825	-88	421	427	LIEDYER								Mascot	
	1045.6	1045.4651	-0.1349	-129	540	548	TLKALENTR								Mascot	
	1086.5691	1086.4663	-0.1028	-95	516	524	NEFGHITIR								Mascot	
	1166.5354	1166.4423	-0.0931	-80	516	524	NEFGHITIR				Phospho (ST)[7]				Mascot	
	1183.6582	1183.5468	-0.1114	-94	357	367	SGHAVLFVTPR								Mascot	
	1303.7004	1303.5739	-0.1265	-97	100	111	ELALQVAEAFGR								Mascot	
	1303.7004	1303.5739	-0.1265	-97	100	111	ELALQVAEAFGR		44	0					Mascot	
	1324.7583	1324.6318	-0.1265	-95	549	560	ISGVLINLQPDR								Mascot	
	1475.804	1475.6548	-0.1492	-101	197	210	QVALFSATMPAAIR								Mascot	
	1491.7988	1491.6517	-0.1471	-99	197	210	QVALFSATMPAAIR				Oxidation (M)[9]				Mascot	

	1543.8326	1543.6813	-0.1513	-98	308	322	DGTIDILIATDVAAR								Mascot
	1616.814	1616.6598	-0.1542	-95	284	299	GFAAAAINGDINQAQR								Mascot
		1616.6598													Mascot
	1719.8547	1719.7057	-0.149	-87	404	419	DSISDSLNAPGIDLFR								Mascot
	1719.8547	1719.7057	-0.149	-87	404	419	DSISDSLNAPGIDLFR		75	99.739					Mascot
	1735.8934	1735.7223	-0.1711	-99	254	268	ILEVEEGDAMIVFVR				Oxidation (M)[10]				Mascot
	1753.9456	1753.7728	-0.1728	-99	498	515	VGPGHIVGAIANEGGLHR								Mascot
	1753.9456	1753.7728	-0.1728	-99	498	515	VGPGHIVGAIANEGGLHR		37	0					Mascot
	1782.9232	1782.7469	-0.1763	-99	383	398	LIEAELPSVEDVNAQR								Mascot
	1838.9971	1838.8131	-0.184	-100	428	445	ESNVPLADIAAALAVQTR								Mascot
	1838.9971	1838.8131	-0.184	-100	428	445	ESNVPLADIAAALAVQTR		64	96.338					Mascot
	2023.0243	2022.8251	-0.1992	-98	402	419	FRDSISDSLNAPGIDLFR								Mascot
	2295.1152	2294.8843	-0.2309	-101	329	347	ISHVINYDIPHDTESYVHR								Mascot
	2720.2317	2719.9868	-0.2449	-90	165	187	LDYLVLDEADEMLTMGF				Oxidation (M)[12,15]				Mascot
							AEEVER								
	2905.5205	2905.2136	-0.3069	-106	112	138	YGSHLPQVNVLPIYGGSS								Mascot
							YTVQLSGLR								
**Gel Idx/Pos**		179/H6					**Instr./Gel Origin**		BA2060/121115A				**Process Statu**	Analysis Succeeded	
**Plate [#] Name**		[1] 31598					**Instrument Sample Name**						**Spectra**	8	
**Rank**	**Protein Name**					**Accession No.**	**Protein MW**	**Protein PI**	**Pep. Count**	**Protein Score**	**Protein Score C. I. %**	**otal Ion Score**	**Total Ion C. I. %**		
1	acetyl-CoA acetyltransferase [Mycobacterium					gi|118468168	45205.2	6.13	12	225	100	221	100		
	smegmatis str. MC2 155]														
	**Peptide Information**														
	**Calc. Mass**	**Obsrv. Mass**	**± da**	**± ppm**	**Start Seq.**	**End Seq.**	**Sequence**		**Ion Score**	**C. I. %**	**Modification**			**Rank**	**Result Type**
	811.3709	811.2985	-0.0724	-89	364	370	ALGSIDR				Phospho (ST)[4]				Mascot
	960.5472	960.3844	-0.1628	-169	364	372	ALGSIDRTK								Mascot
	1470.6117	1470.6953	0.0836	57	173	186	TGMSMGEHAAVTAK				Phospho (ST)[1]				Mascot
	1481.7455	1481.5947	-0.1508	-102	192	204	RVEQDELAAASHR								Mascot
	1677.917	1677.7488	-0.1682	-100	157	172	LPASLGVEIPVNSEPR		93	99.996					Mascot
	1677.917	1677.7488	-0.1682	-100	157	172	LPASLGVEIPVNSEPR								Mascot
	1756.9955	1756.8182	-0.1773	-101	46	63	FNLAGEQLGAVIGGAVLK								Mascot
	1793.8866	1793.7445	-0.1421	-79	414	432	GLISICAAGGQGVAAILEA				Phospho (ST)[4]				Mascot
	1896.9674	1896.7751	-0.1923	-101	373	392	LNVNGSSLAAGHPFAAT								Mascot
							GGR								
	2056.9001	2056.8567	-0.0434	-21	373	392	LNVNGSSLAAGHPFAAT				Phospho (ST)[6,7]				Mascot
							GGR								
	2368.105	2367.8633	-0.2417	-102	112	135	YESAAAGGVDTASDAPIA								Mascot
		2367.8633													Mascot
							FGNDLR								
	2368.105	2367.8633	-0.2417	-102	112	135	YESAAAGGVDTASDAPIA		128	100					Mascot
							FGNDLR								
	2458.1304	2457.8787	-0.2517	-102	22	45	SDGAYANASNQDMFTAA								Mascot
							LGGLIDR								
	2474.1252	2473.8738	-0.2514	-102	22	45	SDGAYANASNQDMFTAA				Oxidation (M)[13]				Mascot
							LGGLIDR								
	2553.2119	2552.9509	-0.261	-102	205	227	NMAAAYDAGFFDDLVTP								Mascot
							FLGVYR								
	3118.4746	3118.165	-0.3096	-99	251	282	AGDATMTAGNSTPLTDG								Mascot
							ASVALLAGEDWAQAR								
	3134.4695	3134.1616	-0.3079	-98	251	282	AGDATMTAGNSTPLTDG				Oxidation (M)[6]				Mascot
							ASVALLAGEDWAQAR								
2	acetyl-CoA acetyltransferase [Mycobacterium gilvum					gi|145221015	45663.4	6.13	6	93	99.558	93	99.996		
	PYR-GCK]														
	**Peptide Information**														
	**Calc. Mass**	**Obsrv. Mass**	**± da**	**± ppm**	**Start Seq.**	**End Seq.**	**Sequence**		**Ion Score**	**C. I. %**	**Modification**			**Rank**	**Result Type**
	811.3709	811.2985	-0.0724	-89	365	371	ALGSIDR				Phospho (ST)[4]				Mascot
	1470.6117	1470.6953	0.0836	57	174	187	TGMSMGEHAAVTAK				Phospho (ST)[1]				Mascot
	1677.917	1677.7488	-0.1682	-100	158	173	LPASLGVEIPVNSEPR		93	99.996					Mascot
	1677.917	1677.7488	-0.1682	-100	158	173	LPASLGVEIPVNSEPR								Mascot
	1729.9153	1729.7107	-0.2046	-118	415	433	GLISVCAAGGQGVTAILE								Mascot
							A								
	1896.9674	1896.7751	-0.1923	-101	374	393	LNVNGSSLAAGHPFAAT								Mascot
							GGR								
	2056.9001	2056.8567	-0.0434	-21	374	393	LNVNGSSLAAGHPFAAT				Phospho (ST)[6,7]				Mascot
							GGR								
	2553.3452	2552.9509	-0.3943	-154	408	433	QTGQPVRGLISVCAAGG				Carbamidomethyl (C)[13]				Mascot
							QGVTAILEA								
**Gel Idx/Pos**		180/H7					**Instr./Gel Origin**		BA2060/121115A				**Process Statu**	Analysis Succeeded	
**Plate [#] Name**		[1] 31598					**Instrument Sample Name**						**Spectra**	11	
**Rank**	**Protein Name**					**Accession No.**	**Protein MW**	**Protein PI**	**Pep. Count**	**Protein Score**	**Protein Score C. I. %**	**otal Ion Score**	**Total Ion C. I. %**		
1	S30AE family protein [Mycobacterium smegmatis str.					gi|118473146	26382.2	6.27	15	253	100	217	100		
	MC2 155]														
	**Peptide Information**														
	**Calc. Mass**	**Obsrv. Mass**	**± da**	**± ppm**	**Start Seq.**	**End Seq.**	**Sequence**		**Ion Score**	**C. I. %**	**Modification**			**Rank**	**Result Type**
	1042.5527	1042.4508	-0.1019	-98	147	156	DPLAGLDVSR								Mascot
	1091.5632	1091.46	-0.1032	-95	220	228	HAFDYGLIR		59	86.723					Mascot
	1091.5632	1091.46	-0.1032	-95	220	228	HAFDYGLIR								Mascot
	1112.5483	1112.4415	-0.1068	-96	39	47	NVEVPDHFR								Mascot
	1112.5483	1112.4415	-0.1068	-96	39	47	NVEVPDHFR		44	0					Mascot
	1221.6222	1221.5055	-0.1167	-96	209	218	ETDRPSVVYR								Mascot
	1227.5898	1227.4729	-0.1169	-95	82	91	NCQHVEITAR				Carbamidomethyl (C)[2]				Mascot
	1247.532	1247.5463	0.0143	11	136	146	TPVSLAEATAK				Phospho (ST)[4], Phospho (ST)[1]				Mascot
	1343.5974	1343.4677	-0.1297	-97	163	174	YNDGVAEHEPGR								Mascot
	1355.6508	1355.5553	-0.0955	-70	220	230	HAFDYGLIRLA				Phospho (Y)[5]				Mascot
	1463.6573	1463.6353	-0.022	-15	82	93	NCQHVEITARGR				Phospho (ST)[8]				Mascot
	1649.8169	1649.6581	-0.1588	-96	63	75	TIYLFDVELDHER		114	100					Mascot
	1649.8169	1649.6581	-0.1588	-96	63	75	TIYLFDVELDHER								Mascot
	1699.8246	1699.6577	-0.1669	-98	147	162	DPLAGLDVSREDGEAR								Mascot
	1980.8644	1980.6694	-0.195	-98	99	116	GEACADSFYTAFESAVQ				Carbamidomethyl (C)[4]				Mascot
							K								
	2210.7168	2210.8848	0.168	76	2	18	SSHSMDSSRTMVVEDDR				Oxidation (M)[5,11], Phospho (ST)[1,2,4]				Mascot
	2229.8013	2229.9373	0.136	61	1	18	MSSHSMDSSRTMVVEDD				Phospho (ST)[2,3]				Mascot
							R								
	2853.4048	2853.1213	-0.2835	-99	11	36	TMVVEDDRESTAGTTPE								Mascot
							RPHAEVVVK								
	2869.3997	2869.105	-0.2947	-103	11	36	TMVVEDDRESTAGTTPE				Oxidation (M)[2]				Mascot
							RPHAEVVVK								
2	S30AE family protein [Mycobacterium tuberculosis					gi|15842830	25077.9	9.03	4	111	99.993	111	100		
	CDC1551]														
	**Peptide Information**														
	**Calc. Mass**	**Obsrv. Mass**	**± da**	**± ppm**	**Start Seq.**	**End Seq.**	**Sequence**		**Ion Score**	**C. I. %**	**Modification**			**Rank**	**Result Type**
	1221.6222	1221.5055	-0.1167	-96	198	207	DTERPSVVYR								Mascot
	1343.6256	1343.4677	-0.1579	-118	208	217	RHAYDYGLIR				Phospho (Y)[4]				Mascot
	1649.8169	1649.6581	-0.1588	-96	51	63	TIYLFDVELDHER		114	100					Mascot
	1649.8169	1649.6581	-0.1588	-96	51	63	TIYLFDVELDHER								Mascot
	2396.084	2395.988	-0.096	-40	87	108	GEACADSFYAALESAVV				Phospho (Y)[9]				Mascot
							KLESR								
**Gel Idx/Pos**		181/H8					**Instr./Gel Origin**		BA2060/121115A				**Process Statu**	Analysis Succeeded	

Plate [#] Name	[1] 31598	Instrument Sample Name	Spectra	8

Rank	Protein Name	Accession No.	Protein MW	Protein PI	Pep. Count	Protein Score	Protein Score C. I. %	tal Ion Score	Total Ion C. I. %
1	hypothetical protein MSMEG_5010 [Mycobacterium smegmatis str. MC2 155]	gi|118469659	19502.9	6	18	426	100	293	100

Peptide Information

Calc. Mass	Obsrv. Mass	± da	± ppm	Start Seq.	End Seq.	Sequence	Ion Score	C. I. %	Modification	Rank	Result Type
837.4213	837.348	-0.0733	-88	63	68	FREETR					Mascot
870.4553	870.467	0.0117	13	3	8	HQHHRR					Mascot
917.3876	917.3519	-0.0357	-39	63	68	FREETR			Phospho (ST)[5]		Mascot
931.4995	931.4213	-0.0782	-84	86	93	VSWGVEVR					Mascot
931.4995	931.4213	-0.0782	-84	86	93	VSWGVEVR	28	0			Mascot
1004.5159	1004.428	-0.0879	-88	135	143	SDALAWSVR					Mascot
1004.5159	1004.428	-0.0879	-88	135	143	SDALAWSVR	46	0			Mascot
1017.4907	1017.403	-0.0877	-86	1	7	MRHQHHR			Oxidation (M)[1]		Mascot
1059.5945	1059.5034	-0.0911	-86	85	93	KVSWGVEVR					Mascot
1059.5945	1059.5034	-0.0911	-86	85	93	KVSWGVEVR	40	0			Mascot
1193.5831	1193.5153	-0.0678	-57	158	167	EAMKTVDDLR			Oxidation (M)[3]		Mascot
1228.6168	1228.5126	-0.1042	-85	162	173	TVDDLRAEGPGV					Mascot
1247.6492	1247.5411	-0.1081	-87	133	143	SRSDALAWSVR					Mascot
1277.6233	1277.5984	-0.0249	-19	74	84	IADEAQDRFGR					Mascot
1309.5807	1309.4681	-0.1126	-86	9	20	DASEAAEWFAGR	106	100			Mascot
1309.5807	1309.4681	-0.1126	-86	9	20	DASEAAEWFAGR					Mascot
1356.7482	1356.632	-0.1162	-86	120	132	QVLDTLVDAGVAR					Mascot
1366.7114	1366.5922	-0.1192	-87	144	155	LVGQHADEWLAK	57	85.585			Mascot
1366.7114	1366.5922	-0.1192	-87	144	155	LVGQHADEWLAK					Mascot
1465.6819	1465.5508	-0.1311	-89	8	20	RDASEAAEWFAGR					Mascot
1515.8175	1515.6823	-0.1352	-89	101	113	IMFTHIAVPVMTR					Mascot
1515.8175	1515.6823	-0.1352	-89	101	113	IMFTHIAVPVMTR	44	0			Mascot
1531.8124	1531.6758	-0.1366	-89	101	113	IMFTHIAVPVMTR	17	0	Oxidation (M)[11]		Mascot
1531.8124	1531.6758	-0.1366	-89	101	113	IMFTHIAVPVMTR			Oxidation (M)[2]		Mascot
1547.8073	1547.6664	-0.1409	-91	101	113	IMFTHIAVPVMTR			Oxidation (M)[2,11]		Mascot
1707.7399	1707.6217	-0.1182	-69	101	113	IMFTHIAVPVMTR			Oxidation (M)[2,11], Phospho (ST)[4,12]		Mascot
1787.8962	1787.7352	-0.161	-90	21	36	LPDGWFTGDPTVIVDR					Mascot
2044.0081	2043.8152	-0.1929	-94	37	55	EEITVIGTLPDVEGESEAR					Mascot
2283.907	2283.9561	0.0491	21	37	55	EEITVIGTLPDVEGESEAR			Phospho (ST)[16], Phospho (ST)[4,8]		Mascot
3812.8865	3812.498	-0.3885	-102	21	55	LPDGWFTGDPTVIVDREE					Mascot
						ITVIGTLPDVEGESEAR					

Table S4. Differential Gene Expression Profile of WT PknK and K55M mutant PknK overexpression strains as a function of growth

	Up Regulated Genes[a]				
**Strain_Condition**	**Cellular Processes and Signaling**	**Information Storage and Processing**	**Metabolism**	**Poorly Characterized**	**Total No. of DEG[c]**
WT_{OE} vs VC_24 h	6	21	52	20	99
Mut_{OE} vs VC_24 h	13	24	32	18	87
WT_{OE} vs VC_96 h	103	121	316	192	732
Mut_{OE} vs VC_ 96 h	11	10	38	10	69
	**Down Regulated Genes[b]**				
**Strain_Condition**	**Cellular Processes and Signaling**	**Information Storage and Processing**	**Metabolism**	**Poorly Characterized**	**Total No. of DEG[c]**
WT_{OE} vs VC_24 h	33	37	124	75	269
Mut_{OE} vs VC_24 h	23	29	116	65	233
WT_{OE} vs VC_96 h	43	82	295	140	560
Mut_{OE} vs VC_ 96 h	12	33	43	35	123

[a] *Upregulated - fold greater than 1 (log base$_2$)*

[b] *Downregulated - fold less than 1 (log base$_2$)*

[c] *DEG -Differentially expressed genes*

COG annotation was performed using COGs functional categories classification *(http://www.ncbi.nlm.nih.gov/COG)*

Table S5. Genes Up / Down regulated in LIX 79 (WT PknK Overexpression Strain at t=24 h)

Upregulated

For filtering upregulation we consider Geomean fold >=1 in treated sample.

Downregulated

For filtering downregulation we consider Geomean fold <=-1 in treated sample.

Expression fold values are provided in terms of log base 2

					LIX79 t24-1 vs. LIX70 t24-1		
GeneName	Product	COG_Code	Function	RV Number	Geomean_Fold	P-value	Regulation
MSMEG_0699	hypothetical protein	O	CELLULAR PROCESSES AND SIGNALING		1.17	0.260	Up
MSMEG_3448	two-component system sensor kinase	T	CELLULAR PROCESSES AND SIGNALING		1.01	0.047	Up
MSMEG_2695	hypothetical protein	T	CELLULAR PROCESSES AND SIGNALING	RV2744C	1.18	0.272	Up
sufB	FeS assembly protein SufB	O	CELLULAR PROCESSES AND SIGNALING	RV1461	2.06	0.354	Up
MSMEG_5481	hypothetical protein	O	CELLULAR PROCESSES AND SIGNALING	RV0990C	1.04	0.425	Up
MSMEG_0155	transcriptional regulator	T	CELLULAR PROCESSES AND SIGNALING		1.27	0.490	Up
rplV	50S ribosomal protein L22	J	INFORMATION STORAGE AND PROCESSING	Rv0706	1.13	0.128	Up
MSMEG_4486	ArsR family transcriptional regulator	K	INFORMATION STORAGE AND PROCESSING	RV2358	1.51	0.445	Up
MSMEG_5229	hypothetical protein	J	INFORMATION STORAGE AND PROCESSING		1.37	0.023	Up
MSMEG_0778	transcriptional regulator	K	INFORMATION STORAGE AND PROCESSING		1.26	0.038	Up
MSMEG_4154	transposase, Mutator family protein	L	INFORMATION STORAGE AND PROCESSING	RV3431C	2.60	0.044	Up
MSMEG_2794	GntR family transcriptional regulator	K	INFORMATION STORAGE AND PROCESSING		3.61	0.054	Up
rpsS	30S ribosomal protein S19	J	INFORMATION STORAGE AND PROCESSING	Rv0705	1.06	0.101	Up
MSMEG_4948	Sua5/YciO/YrdC/YwlC family protein	J	INFORMATION STORAGE AND PROCESSING	RV1301	1.07	0.129	Up
MSMEG_3959	transcriptional regulator	K	INFORMATION STORAGE AND PROCESSING		2.05	0.149	Up
MSMEG_1445	30S ribosomal protein S17	J	INFORMATION STORAGE AND PROCESSING	Rv0710	1.01	0.188	Up
MSMEG_3606	transcriptional regulator	K	INFORMATION STORAGE AND PROCESSING		1.02	0.190	Up
MSMEG_3960	transcriptional regulator	K	INFORMATION STORAGE AND PROCESSING		1.85	0.203	Up
MSMEG_4439	acetyltransferase	J	INFORMATION STORAGE AND PROCESSING		1.07	0.203	Up
MSMEG_3121	DNA-binding protein	K	INFORMATION STORAGE AND PROCESSING	RV1460	2.57	0.272	Up
MSMEG_3264	transcriptional regulator	K	INFORMATION STORAGE AND PROCESSING		1.26	0.290	Up
MSMEG_6139	HTH-type transcriptional regulator	K	INFORMATION STORAGE AND PROCESSING	RV2912C	1.39	0.315	Up
MSMEG_4492	amidase	J	INFORMATION STORAGE AND PROCESSING	Rv2363	1.19	0.358	Up
MSMEG_6441	TetR family transcriptional regulator	K	INFORMATION STORAGE AND PROCESSING	Rv3855	2.87	0.371	Up
dinP	DNA polymerase IV	L	INFORMATION STORAGE AND PROCESSING		1.65	0.398	Up
MSMEG_2989	LacI family transcriptional regulator	K	INFORMATION STORAGE AND PROCESSING		1.33	0.404	Up
MSMEG_5405	ArsR family transcriptional regulator	K	INFORMATION STORAGE AND PROCESSING	RV3744	1.00	0.484	Up
MSMEG_1546	coenzyme B12-dependent glycerol dehydrogenase small su	Q	METABOLISM		1.42	0.047	Up
MSMEG_3786	D-amino acid deaminase	E	METABOLISM		1.21	0.055	Up
MSMEG_6591	class V aminotransferase	E	METABOLISM		1.09	0.076	Up
folB	dihydroneopterin aldolase	H	METABOLISM	Rv3607c	1.24	0.129	Up
MSMEG_6802	ABC transporter ATP-binding protein	G	METABOLISM		1.29	0.158	Up
MSMEG_3266	maltose/maltodextrin-binding protein	G	METABOLISM		1.62	0.323	Up
MSMEG_6294	caib/baif family protein	C	METABOLISM		1.33	0.348	Up
MSMEG_2680	amino acid transporter	E	METABOLISM		1.19	0.353	Up
argC	N-acetyl-gamma-glutamyl-phosphate reductase	E	METABOLISM	Rv1652	1.15	0.412	Up
MSMEG_6225	proton antiporter efflux pump	G	METABOLISM		1.42	0.417	Up
MSMEG_2405	MarR family transcriptional regulator	E	METABOLISM		1.05	0.521	Up
MSMEG_3269	sugar ABC transporter ATP-binding protein	G	METABOLISM		1.86	0.525	Up
MSMEG_6804	sugar ABC transporter substrate-binding protein	G	METABOLISM		1.91	0.005	Up
MSMEG_2796	integral membrane protein	P	METABOLISM		1.09	0.020	Up
MSMEG_2911	integral membrane transporter	G	METABOLISM		1.14	0.028	Up
MSMEG_1124	ferredoxin FdxA	C	METABOLISM		1.00	0.028	Up
MSMEG_6846	C4 decarboxylate transporter	G	METABOLISM		1.12	0.042	Up
MSMEG_6046	ABC transporter ATP-binding protein	P	METABOLISM		2.36	0.043	Up
MSMEG_4527	ferredoxin sulfite reductase	P	METABOLISM		1.32	0.043	Up
MSMEG_1566	oxidoreductase	C	METABOLISM		1.06	0.068	Up
MSMEG_4487	ferric uptake regulation protein	P	METABOLISM	Rv2359	1.31	0.075	Up
MSMEG_4785	mce-family protein mce1f	Q	METABOLISM		1.21	0.076	Up
pruA	1-pyrroline-5-carboxylate dehydrogenase	C	METABOLISM	Rv1187	1.13	0.078	Up
MSMEG_1318	polysaccharide deacetylase	G	METABOLISM		2.18	0.092	Up
MSMEG_2471	pyruvate dehydrogenase subunit alpha	C	METABOLISM		1.89	0.115	Up
MSMEG_6050	solute-binding lipoprotein	P	METABOLISM	RV2059	1.03	0.128	Up
MSMEG_6047	cation ABC transporter periplasmic cation-binding protein	P	METABOLISM		2.46	0.142	Up
MSMEG_5995	P450 heme-thiolate protein	Q	METABOLISM	Rv3545c	1.14	0.150	Up
MSMEG_0338	acyl-CoA dehydrogenase	I	METABOLISM		1.07	0.163	Up
MSMEG_6262	FwdC/FmdC family protein	E	METABOLISM		1.06	0.170	Up
MSMEG_4510	peptide synthetase mbtf	Q	METABOLISM	Rv2379c	1.10	0.173	Up
MSMEG_5477	2-hydroxy-3-oxopropionate reductase	I	METABOLISM		1.00	0.193	Up
MSMEG_2795	transmembrane transporter	P	METABOLISM		2.55	0.194	Up
MSMEG_5523	peptidase	E	METABOLISM		1.27	0.199	Up
argJ	bifunctional ornithine acetyltransferase/N-acetylglutamate	E	METABOLISM	Rv1653	1.90	0.201	Up
MSMEG_4036	crotonobetaine/carnitine-CoA ligase	Q	METABOLISM		1.63	0.225	Up
MSMEG_4515	non-ribosomal peptide synthetase	Q	METABOLISM	Rv2383c	1.19	0.247	Up
MSMEG_2172	dicarboxylate-carrier protein	P	METABOLISM		1.12	0.289	Up
MSMEG_6440	monooxygenase, flavin-binding family protein	P	METABOLISM	Rv3854c	2.12	0.322	Up
MSMEG_3389	acetolactate synthase	H	METABOLISM		1.08	0.327	Up
MSMEG_3355	hypothetical protein	G	METABOLISM		1.04	0.333	Up
MSMEG_3268	ABC transporter permease	G	METABOLISM		1.67	0.355	Up
MSMEG_5577	fructokinase	G	METABOLISM		1.16	0.362	Up
MSMEG_1821	acyl-CoA dehydrogenase	I	METABOLISM	Rv3274c	1.12	0.364	Up
MSMEG_6141	N-acyl-D-glutamate amidohydrolase	Q	METABOLISM	RV2913C	1.93	0.427	Up
MSMEG_3267	transporter	G	METABOLISM		1.78	0.433	Up
MSMEG_3198	NUDIX family hydrolase	F	METABOLISM	RV1593C	1.92	0.436	Up
cysD	sulfate adenylyltransferase subunit 2	H	METABOLISM	Rv1285	1.88	0.445	Up
MSMEG_5576	D-mannonate oxidoreductase	G	METABOLISM		1.75	0.445	Up
MSMEG_2323	GntR family transcriptional regulator	E	METABOLISM		1.68	0.479	Up
MSMEG_4886	major facilitator family protein transporter	G	METABOLISM		1.13	0.500	Up
MSMEG_4325	malonyl CoA-ACP transacylase	I	METABOLISM	Rv2243	1.27	0.555	Up
MSMEG_6069	CobW/P47K domain-containing protein	R	POORLY CHARACTERIZED	RV0106	1.72	0.009	Up
MSMEG_6399	antigen 85-C	R	POORLY CHARACTERIZED		1.47	0.078	Up
MSMEG_3879	short chain dehydrogenase	R	POORLY CHARACTERIZED	RV2073C	1.58	0.290	Up
MSMEG_3607	short chain dehydrogenase	R	POORLY CHARACTERIZED		1.21	0.461	Up
mhpB	3-(2,3-dihydroxyphenyl)propionate dioxygenase	S	POORLY CHARACTERIZED		1.04	0.003	Up
MSMEG_0992	2-nitropropane dioxygenase	R	POORLY CHARACTERIZED	RV1533	1.44	0.011	Up
MSMEG_0300	amidohydrolase	R	POORLY CHARACTERIZED		1.48	0.034	Up
MSMEG_1544	PduO protein	R	POORLY CHARACTERIZED		1.66	0.044	Up
MSMEG_3464	alcohol dehydrogenase	R	POORLY CHARACTERIZED		1.07	0.094	Up
MSMEG_4125	oxidoreductase, short chain dehydrogenase/reductase	R	POORLY CHARACTERIZED		1.06	0.144	Up

putP	sodium/proline symporter	R	POORLY CHARACTERIZED		1.19	0.243	Up
MSMEG_2755	hypothetical protein	S	POORLY CHARACTERIZED	RV2707	1.74	0.274	Up
MSMEG_1600	hypothetical protein	R	POORLY CHARACTERIZED	RV3413C	1.04	0.275	Up
MSMEG_4176	hypothetical protein	S	POORLY CHARACTERIZED		1.03	0.293	Up
MSMEG_3265	arabitol-phosphate dehydrogenase	R	POORLY CHARACTERIZED		2.78	0.306	Up
MSMEG_6117	glucarate dehydratase	R	POORLY CHARACTERIZED		1.04	0.359	Up
MSMEG_5565	hypothetical protein	S	POORLY CHARACTERIZED		1.50	0.411	Up
MSMEG_4840	Rieske (2Fe-2S) domain-containing protein	R	POORLY CHARACTERIZED		1.01	0.438	Up
MSMEG_2534	carboxylesterase	R	POORLY CHARACTERIZED		1.13	0.441	Up
MSMEG_3604	sorbitol utilization protein SOU2	R	POORLY CHARACTERIZED		1.25	0.595	Up
					LIX79 t24-1 vs. LIX70 t24-1		
GeneName	Product	COG_Code	Function	RV Number	Geomean_Fold	P-value	Regulation
MSMEG_0064	PPE family protein	N	CELLULAR PROCESSES AND SIGNALING	Rv3873	-1.28	0.002	Down
MSMEG_2804	two-component system sensor kinase	T	CELLULAR PROCESSES AND SIGNALING		-2.00	0.005	Down
MSMEG_6281	N-acetylmuramoyl-L-alanine amidase	M	CELLULAR PROCESSES AND SIGNALING	RV3717	-1.07	0.006	Down
MSMEG_2416	hypothetical protein	D	CELLULAR PROCESSES AND SIGNALING	RV2927C	-1.36	0.007	Down
MSMEG_1097	glycosyl transferase family protein	M	CELLULAR PROCESSES AND SIGNALING		-1.63	0.008	Down
MSMEG_4231	UDP-N-acetylmuramoyl-tripeptide--D-alanyl-D-alanine ligas	M	CELLULAR PROCESSES AND SIGNALING	Rv2157c	-1.58	0.008	Down
MSMEG_1131	tryptophan-rich sensory protein	T	CELLULAR PROCESSES AND SIGNALING		-1.12	0.009	Down
ftsW	cell division protein FtsW	D	CELLULAR PROCESSES AND SIGNALING	RV2154C	-1.16	0.022	Down
MSMEG_2718	(Fe-S)-binding protein	O	CELLULAR PROCESSES AND SIGNALING		-1.29	0.052	Down
MSMEG_1803	RsbW protein	T	CELLULAR PROCESSES AND SIGNALING	Rv3287c	-1.44	0.055	Down
MSMEG_2690	DNA translocase FtsK	D	CELLULAR PROCESSES AND SIGNALING	Rv2748c	-1.13	0.064	Down
MSMEG_0067	hypothetical protein	D	CELLULAR PROCESSES AND SIGNALING		-1.18	0.067	Down
MSMEG_5661	ABC transporter ATP-binding protein	V	CELLULAR PROCESSES AND SIGNALING		-1.23	0.067	Down
MSMEG_6822	beta-lactamase	V	CELLULAR PROCESSES AND SIGNALING		-1.88	0.089	Down
MSMEG_4233	penicillin binding protein transpeptidase domain-containin	M	CELLULAR PROCESSES AND SIGNALING	Rv2163c	-1.36	0.109	Down
MSMEG_6129	hypothetical protein	T	CELLULAR PROCESSES AND SIGNALING		-1.02	0.111	Down
cysT	sulfate ABC transporter permease CysT	O	CELLULAR PROCESSES AND SIGNALING	Rv2399c	-1.13	0.112	Down
MSMEG_3541	cytochrome C biogenesis protein transmembrane region	O	CELLULAR PROCESSES AND SIGNALING	RV2877C	-1.04	0.115	Down
MSMEG_1315	transporter small conductance mechanosensitive ion chann	M	CELLULAR PROCESSES AND SIGNALING		-1.73	0.125	Down
MSMEG_5474	universal stress protein family protein	T	CELLULAR PROCESSES AND SIGNALING		-1.73	0.140	Down
MSMEG_0670	FAD dependent oxidoreductase	O	CELLULAR PROCESSES AND SIGNALING		-1.39	0.154	Down
MSMEG_6935	N-acetylmuramoyl-L-alanine amidase	M	CELLULAR PROCESSES AND SIGNALING	RV3915	-1.04	0.162	Down
MSMEG_6541	anti-sigma factor antagonist	T	CELLULAR PROCESSES AND SIGNALING		-2.55	0.180	Down
MSMEG_1661	D-alanyl-D-alanine carboxypeptidase	M	CELLULAR PROCESSES AND SIGNALING	Rv3330	-1.18	0.180	Down
MSMEG_0449	transporter major facilitator family protein	T	CELLULAR PROCESSES AND SIGNALING		-1.57	0.184	Down
MSMEG_6319	penicillin-binding protein, transpeptidase	M	CELLULAR PROCESSES AND SIGNALING		-1.47	0.189	Down
MSMEG_3477	inv protein	M	CELLULAR PROCESSES AND SIGNALING	RV1566C	-1.29	0.211	Down
MSMEG_0933	hypothetical protein	M	CELLULAR PROCESSES AND SIGNALING	Rv0486	-1.39	0.220	Down
MSMEG_4384	penicillin binding protein transpeptidase domain-containin	M	CELLULAR PROCESSES AND SIGNALING		-1.55	0.223	Down
MSMEG_5496	MscS mechanosensitive ion channel	M	CELLULAR PROCESSES AND SIGNALING		-1.79	0.224	Down
MSMEG_5454	choloylglycine hydrolase	M	CELLULAR PROCESSES AND SIGNALING		-1.27	0.249	Down
MSMEG_1854	valosin containing protein-1	O	CELLULAR PROCESSES AND SIGNALING		-1.16	0.285	Down
MSMEG_4207	universal stress protein family protein	T	CELLULAR PROCESSES AND SIGNALING		-1.54	0.339	Down
MSMEG_6356	AraC family transcriptional regulator	K	INFORMATION STORAGE AND PROCESSING		-1.78	0.004	Down
MSMEG_0265	uracil DNA glycosylase superfamily protein	L	INFORMATION STORAGE AND PROCESSING		-2.06	0.009	Down
MSMEG_1970	sigma factor	K	INFORMATION STORAGE AND PROCESSING		-3.36	0.045	Down
MSMEG_1804	RNA polymerase sigma factor SigF	K	INFORMATION STORAGE AND PROCESSING	Rv3286c	-1.48	0.053	Down
MSMEG_1771	methylase	J	INFORMATION STORAGE AND PROCESSING		-1.86	0.063	Down
MSMEG_0209	ribonuclease inhibitor	K	INFORMATION STORAGE AND PROCESSING		-1.28	0.069	Down
MSMEG_1909	HTH-type transcriptional regulator YnfL	K	INFORMATION STORAGE AND PROCESSING		-1.66	0.073	Down
MSMEG_0523	DNA-binding protein	K	INFORMATION STORAGE AND PROCESSING		-1.79	0.074	Down
MSMEG_3885	DEAD/DEAH box helicase	L	INFORMATION STORAGE AND PROCESSING	Rv2092c	-1.43	0.075	Down
MSMEG_3410	TetR family transcriptional regulator	K	INFORMATION STORAGE AND PROCESSING		-1.53	0.077	Down
MSMEG_1090	amidase	J	INFORMATION STORAGE AND PROCESSING		-1.18	0.091	Down
MSMEG_3274	MerR family transcriptional regulator	K	INFORMATION STORAGE AND PROCESSING		-1.10	0.123	Down
MSMEG_1741	TetR family transcriptional regulator	K	INFORMATION STORAGE AND PROCESSING		-1.00	0.134	Down
MSMEG_5570	ATP-dependent DNA ligase	L	INFORMATION STORAGE AND PROCESSING	RV0938	-1.27	0.143	Down
MSMEG_4394	LysR family transcriptional regulator	K	INFORMATION STORAGE AND PROCESSING		-1.13	0.144	Down
MSMEG_0673	hypothetical protein	A	INFORMATION STORAGE AND PROCESSING	RV1435C	-1.57	0.149	Down
MSMEG_6903	PadR family transcriptional regulator	K	INFORMATION STORAGE AND PROCESSING	RV0047C	-1.22	0.165	Down
MSMEG_3822	GntR family transcriptional regulator	K	INFORMATION STORAGE AND PROCESSING		-1.49	0.166	Down
MSMEG_4234	hypothetical protein	A	INFORMATION STORAGE AND PROCESSING	RV2164C	-1.40	0.173	Down
MSMEG_6736	LacI family transcriptional regulator	K	INFORMATION STORAGE AND PROCESSING		-1.10	0.177	Down
MSMEG_5987	two-component regulator	K	INFORMATION STORAGE AND PROCESSING		-1.22	0.187	Down
MSMEG_4620	NAD-dependent deacetylase 1	K	INFORMATION STORAGE AND PROCESSING		-1.02	0.188	Down
rnhA	ribonuclease H	L	INFORMATION STORAGE AND PROCESSING		-1.07	0.192	Down
MSMEG_0211	ABC transporter ATP-binding protein	L	INFORMATION STORAGE AND PROCESSING		-1.07	0.198	Down
MSMEG_1902	transcriptional regulator	K	INFORMATION STORAGE AND PROCESSING		-1.05	0.205	Down
MSMEG_1784	type I topoisomerase	L	INFORMATION STORAGE AND PROCESSING		-1.08	0.227	Down
ung	uracil-DNA glycosylase	L	INFORMATION STORAGE AND PROCESSING	Rv2976c	-1.26	0.236	Down
MSMEG_5399	ATP-dependent DNA helicase RecQ	L	INFORMATION STORAGE AND PROCESSING		-1.34	0.252	Down
MSMEG_1681	endoribonuclease L-PSP superfamily protein	J	INFORMATION STORAGE AND PROCESSING		-1.50	0.288	Down
MSMEG_1288	hypothetical protein	L	INFORMATION STORAGE AND PROCESSING		-1.05	0.293	Down
xerC	site-specific tyrosine recombinase XerC	L	INFORMATION STORAGE AND PROCESSING	Rv2894c	-1.06	0.295	Down
selB	selenocysteine-specific translation elongation factor	J	INFORMATION STORAGE AND PROCESSING		-1.14	0.337	Down
MSMEG_3363	TetR family transcriptional regulator	K	INFORMATION STORAGE AND PROCESSING		-1.16	0.342	Down
MSMEG_6807	TetR family transcriptional regulator	K	INFORMATION STORAGE AND PROCESSING		-1.05	0.362	Down
MSMEG_2105	ATP dependent DNA ligase	L	INFORMATION STORAGE AND PROCESSING		-1.10	0.393	Down
MSMEG_0786	serine/threonine protein kinase	L	INFORMATION STORAGE AND PROCESSING	Rv0410c	-1.60	0.422	Down
MSMEG_1317	transcriptional regulator	K	INFORMATION STORAGE AND PROCESSING		-1.00	0.460	Down
MSMEG_4565	oxidoreductase	C	METABOLISM		-1.91	0.002	Down
MSMEG_6467	starvation-induced DNA protecting protein	P	METABOLISM		-1.24	0.003	Down
MSMEG_0374	glycolate oxidase subunit	C	METABOLISM		-1.90	0.007	Down
MSMEG_0232	sugar transporter family protein	G	METABOLISM	RV0191	-1.11	0.009	Down
MSMEG_1775	cytochrome P450 monooxygenase	Q	METABOLISM		-2.42	0.012	Down
MSMEG_0968	cytochrome P450	Q	METABOLISM		-1.31	0.015	Down
MSMEG_0357	transmembrane transporter	G	METABOLISM		-2.77	0.015	Down
MSMEG_3421	inner membrane metabolite transporter YdfJ	G	METABOLISM		-1.03	0.015	Down
MSMEG_0685	oxidoreductase, molybdopterin-binding subunit	C	METABOLISM	RV0375C	-1.97	0.018	Down
MSMEG_4095	monooxygenase	C	METABOLISM		-1.85	0.022	Down
MSMEG_3848	para-nitrobenzyl esterase	I	METABOLISM	Rv2045c	-1.13	0.025	Down
MSMEG_3279	polyamine ABC transporter permease	E	METABOLISM		-1.02	0.031	Down
MSMEG_3364	RhtB family protein transporter	E	METABOLISM		-1.16	0.033	Down
MSMEG_0267	esterase	I	METABOLISM		-2.76	0.035	Down
MSMEG_2927	ABC transporter permease	E	METABOLISM		-1.43	0.038	Down
MSMEG_5962	glycosyl transferase family protein	G	METABOLISM		-2.19	0.041	Down
MSMEG_4355	peptide ABC transporter permease	P	METABOLISM		-2.08	0.044	Down
MSMEG_1846	polysulfide reductase NrfD	P	METABOLISM		-2.20	0.045	Down
MSMEG_5721	acetyl-CoA acetyltransferase	I	METABOLISM	Rv0859	-1.47	0.045	Down

MSMEG_5990	lipid-transfer protein	I	METABOLISM	Rv3540c	-1.10	0.049	Down
MSMEG_5857	oxidoreductase	I	METABOLISM	RV0770	-1.12	0.051	Down
MSMEG_1292	FAD binding domain-containing protein	C	METABOLISM		-1.31	0.053	Down
MSMEG_4356	ABC transporter	P	METABOLISM	Rv3664c	-1.65	0.056	Down
MSMEG_2347	phytoene dehydrogenase	Q	METABOLISM		-2.05	0.057	Down
MSMEG_2103	5,10-methylenetetrahydromethanopterin reductase	C	METABOLISM		-1.07	0.059	Down
treS	trehalose synthase	G	METABOLISM	Rv0126	-1.22	0.060	Down
MSMEG_3314	transporter	G	METABOLISM		-1.16	0.060	Down
MSMEG_6869	regulatory protein	G	METABOLISM		-1.57	0.061	Down
cysA	sulfate ABC transporter ATP-binding protein	P	METABOLISM		-1.76	0.063	Down
MSMEG_5251	hypothetical protein	P	METABOLISM		-1.52	0.065	Down
MSMEG_4501	sodium:dicarboxylate symporter	C	METABOLISM		-1.03	0.065	Down
metX	homoserine O-acetyltransferase	E	METABOLISM		-1.05	0.072	Down
MSMEG_3007	succinate-semialdehyde dehydrogenase	C	METABOLISM		-1.62	0.078	Down
MSMEG_6514	trehalose synthase-fused maltokinase	G	METABOLISM	RV0127	-1.21	0.084	Down
MSMEG_6615	hypothetical protein	Q	METABOLISM		-2.06	0.085	Down
xfp	phosphoketolase	G	METABOLISM		-1.04	0.085	Down
MSMEG_3986	acetyl-coenzyme A synthetase	I	METABOLISM		-1.30	0.086	Down
MSMEG_4618	isochorismatase	Q	METABOLISM		-1.22	0.086	Down
MSMEG_3460	ferric uptake regulation protein	P	METABOLISM		-1.23	0.087	Down
MSMEG_6727	amino acid permease	E	METABOLISM		-1.84	0.089	Down
MSMEG_6740	1-aminocyclopropane-1-carboxylate deaminase	E	METABOLISM		-1.20	0.092	Down
MSMEG_1847	4Fe-4S ferredoxin	C	METABOLISM		-1.57	0.094	Down
ehuD	ectoine/hydroxyectoine ABC transporter permease EhuD	E	METABOLISM		-1.29	0.101	Down
MSMEG_0782	class III aminotransferase	E	METABOLISM		-1.11	0.102	Down
MSMEG_5153	hypothetical protein	I	METABOLISM	RV1157C	-2.09	0.103	Down
MSMEG_0375	phospholipase D	I	METABOLISM		-2.28	0.104	Down
glgB	glycogen branching protein	G	METABOLISM	Rv1326c	-1.34	0.106	Down
MSMEG_1296	uricase	Q	METABOLISM		-2.50	0.106	Down
MSMEG_0637	iron-sulfur binding oxidoreductase	E	METABOLISM		-2.53	0.107	Down
MSMEG_1980	hypothetical protein	C	METABOLISM	RV0575C	-1.54	0.109	Down
MSMEG_4916	alpha-amylase	G	METABOLISM	Rv1327c	-1.49	0.114	Down
MSMEG_0210	LprO protein	G	METABOLISM	Rv0179c	-1.66	0.116	Down
nadE	NAD synthetase	H	METABOLISM	Rv2438c	-1.36	0.118	Down
MSMEG_2925	permease membrane component	E	METABOLISM		-1.14	0.119	Down
MSMEG_3536	sugar transporter	G	METABOLISM		-1.41	0.125	Down
MSMEG_3304	succinate semialdehyde dehydrogenase	C	METABOLISM		-2.15	0.127	Down
MSMEG_1886	Fatty acid desaturase	I	METABOLISM	Rv3229c	-1.74	0.128	Down
MSMEG_0684	aldehyde oxidase	C	METABOLISM		-2.07	0.129	Down
MSMEG_0686	oxidoreductase	C	METABOLISM		-2.26	0.129	Down
MSMEG_0432	bifunctional uroporphyrinogen-III synthetase/response reg	H	METABOLISM	RV0260C	-1.16	0.130	Down
MSMEG_3005	3-hydroxyacyl-CoA dehydrogenase	I	METABOLISM		-1.59	0.130	Down
MSMEG_2346	phytoene synthase	I	METABOLISM		-1.66	0.132	Down
MSMEG_0816	flavin-binding monooxygenase	P	METABOLISM	RV1393C	-1.12	0.134	Down
MSMEG_6671	sulfate/thiosulfate import ATP-binding protein CysA	E	METABOLISM		-1.12	0.137	Down
MSMEG_5473	transporter	E	METABOLISM	Rv0522	-1.45	0.137	Down
MSMEG_6664	methylenetetrahydrofolate reductase	E	METABOLISM		-1.77	0.139	Down
MSMEG_3618	hypothetical protein	I	METABOLISM	Rv1860	-1.02	0.141	Down
MSMEG_1742	oxidoreductase	C	METABOLISM		-2.26	0.144	Down
malQ	4-alpha-glucanotransferase	G	METABOLISM	Rv1781c	-2.05	0.148	Down
MSMEG_1794	dehydrogenase	C	METABOLISM		-2.21	0.148	Down
MSMEG_6769	transporter monovalent cation:proton antiporter-2 (CPA2)	P	METABOLISM		-2.81	0.148	Down
MSMEG_6768	halogenase	C	METABOLISM		-2.44	0.150	Down
MSMEG_2855	virulence factor Mce family protein	Q	METABOLISM		-1.62	0.154	Down
MSMEG_4003	hypothetical protein	E	METABOLISM		-1.43	0.159	Down
MSMEG_1062	O-succinylbenzoic acid--CoA ligase	Q	METABOLISM	Rv0542c	-1.45	0.159	Down
katA	catalase KatA	P	METABOLISM		-2.17	0.165	Down
fni	isopentenyl pyrophosphate isomerase	C	METABOLISM		-2.07	0.165	Down
MSMEG_2289	cytochrome p450	Q	METABOLISM	Rv3059	-1.34	0.169	Down
MSMEG_6663	C5-O-methyltransferase	H	METABOLISM		-1.51	0.170	Down
MSMEG_5826	pyruvate decarboxylase	H	METABOLISM		-2.05	0.173	Down
MSMEG_3999	ABC transporter periplasmic protein	G	METABOLISM		-1.21	0.174	Down
MSMEG_5463	6-phosphogluconate dehydrogenase	I	METABOLISM		-1.58	0.175	Down
MSMEG_5799	nucleoside-diphosphate-sugar epimerase	G	METABOLISM		-1.35	0.179	Down
MSMEG_2728	glutamate transport ATP-binding protein GluA	E	METABOLISM		-1.12	0.181	Down
MSMEG_2122	dihydroxyacetone kinase subunit DhaL	G	METABOLISM		-1.03	0.182	Down
MSMEG_2112	hypothetical protein	E	METABOLISM		-1.87	0.183	Down
MSMEG_5358	acetamidase/formamidase	C	METABOLISM		-1.30	0.184	Down
MSMEG_5877	hypothetical protein	G	METABOLISM	RV3096	-1.02	0.192	Down
MSMEG_2035	amine oxidase	E	METABOLISM	Rv3170	-1.28	0.197	Down
MSMEG_6213	manganese containing catalase	P	METABOLISM		-2.07	0.200	Down
aspA	aspartate ammonia-lyase	E	METABOLISM		-1.88	0.202	Down
MSMEG_1019	ribonucleotide-diphosphate reductase subunit alpha	F	METABOLISM	Rv3051c	-1.22	0.211	Down
MSMEG_1989	phenoxybenzoate dioxygenase subunit beta	C	METABOLISM		-1.53	0.212	Down
MSMEG_5605	cytochrome bd ubiquinol oxidase subunit I	C	METABOLISM		-1.21	0.216	Down
MSMEG_0264	transmembrane transporter	G	METABOLISM		-1.39	0.220	Down
MSMEG_0764	Na+/H+ antiporter	P	METABOLISM		-1.49	0.221	Down
MSMEG_5189	oxidoreductase	G	METABOLISM		-1.03	0.223	Down
MSMEG_5559	metabolite/sugar transporter	G	METABOLISM	Rv3331	-2.55	0.225	Down
MSMEG_6582	pyridine nucleotide-disulfide oxidoreductase domain-conta	C	METABOLISM		-1.21	0.227	Down
MSMEG_1112	aconitate hydratase	C	METABOLISM		-1.82	0.232	Down
MSMEG_2205	acyl-CoA dehydrogenase	I	METABOLISM		-1.20	0.234	Down
MSMEG_5400	dehydrogenase	Q	METABOLISM		-2.75	0.244	Down
MSMEG_4000	ABC transporter ATP-binding protein	G	METABOLISM		-1.09	0.249	Down
MSMEG_3992	integral membrane transporter	G	METABOLISM		-1.04	0.255	Down
MSMEG_4318	hypothetical protein	P	METABOLISM		-1.72	0.256	Down
MSMEG_1695	phosphoglucomutase/phosphomannomutase	G	METABOLISM	Rv3308	-1.17	0.263	Down
MSMEG_0206	acyltransferase	I	METABOLISM		-1.09	0.264	Down
MSMEG_1885	2Fe-2S iron-sulfur cluster binding domain-containing protei	C	METABOLISM	RV3230C	-2.06	0.265	Down
MSMEG_5908	acyl-CoA synthetase	Q	METABOLISM	Rv3506	-1.23	0.272	Down
catA	catechol 1,2-dioxygenase	Q	METABOLISM		-1.05	0.274	Down
MSMEG_2470	acyl-CoA thioesterase	I	METABOLISM		-1.13	0.279	Down
MSMEG_0266	arginine decarboxylase	E	METABOLISM	RV2531C	-1.43	0.284	Down
MSMEG_4379	isochorismatase hydrolase	Q	METABOLISM		-1.39	0.287	Down
MSMEG_0614	methyltransferase	Q	METABOLISM	RV0281	-1.30	0.298	Down
MSMEG_1477	major facilitator superfamily protein	G	METABOLISM		-1.57	0.302	Down
MSMEG_1682	flavin-containing monooxygenase FMO	P	METABOLISM		-1.26	0.307	Down
treZ	malto-oligosyltrehalose trehalohydrolase	G	METABOLISM	Rv1562c	-1.09	0.319	Down
glgX	glycogen debranching protein GlgX	G	METABOLISM		-1.07	0.333	Down
MSMEG_1986	tartrate dehydrogenase	E	METABOLISM		-1.08	0.349	Down
MSMEG_3930	[NiFe] hydrogenase subunit gamma	C	METABOLISM		-1.09	0.367	Down
MSMEG_4038	vanillin dehydrogenase	C	METABOLISM		-1.09	0.376	Down
cysW	sulfate ABC transporter permease CysW	P	METABOLISM	Rv2398c	-1.64	0.397	Down
MSMEG_2037	hypothetical protein	C	METABOLISM		-1.18	0.398	Down

MSMEG_3954	trehalose 6-phosphate phosphorylase	G	METABOLISM		-1.05	0.461	Down
MSMEG_3689	sodium:solute symporter	R	POORLY CHARACTERIZED		-1.22	0.005	Down
MSMEG_6151	alpha/beta hydrolase	R	POORLY CHARACTERIZED	RV1190	-1.72	0.007	Down
MSMEG_4295	alpha/beta hydrolase	R	POORLY CHARACTERIZED	RV2223C	-1.08	0.009	Down
MSMEG_0674	ErfK/YbiS/YcfS/YnhG family protein	S	POORLY CHARACTERIZED	RV1433	-1.46	0.011	Down
MSMEG_6542	B12-binding domain-containing protein	R	POORLY CHARACTERIZED		-2.14	0.013	Down
MSMEG_6116	OHCU decarboxylase	S	POORLY CHARACTERIZED		-1.05	0.015	Down
MSMEG_5616	glyoxalase/bleomycin resistance protein/dioxygenase	R	POORLY CHARACTERIZED	RV0911	-1.58	0.016	Down
MSMEG_1768	hypothetical protein	R	POORLY CHARACTERIZED	RV0925C	-1.91	0.030	Down
MSMEG_1908	benzoate 1,2-dioxygenase subunit alpha	R	POORLY CHARACTERIZED		-1.48	0.032	Down
MSMEG_3290	regulatory protein	R	POORLY CHARACTERIZED		-1.24	0.044	Down
MSMEG_3581	FabG protein	R	POORLY CHARACTERIZED		-1.55	0.045	Down
MSMEG_3593	hypothetical protein	S	POORLY CHARACTERIZED		-2.68	0.050	Down
mraZ	cell division protein MraZ	S	POORLY CHARACTERIZED		-1.36	0.054	Down
MSMEG_3615	zinc-binding alcohol dehydrogenase	R	POORLY CHARACTERIZED	Rv1862	-1.26	0.063	Down
MSMEG_4570	hypothetical protein	S	POORLY CHARACTERIZED	RV2411C	-1.10	0.067	Down
MSMEG_5237	hypothetical protein	R	POORLY CHARACTERIZED	RV1101C	-1.84	0.069	Down
MSMEG_0536	intracellular protease PfpI family protein	R	POORLY CHARACTERIZED		-1.23	0.072	Down
MSMEG_1806	hypothetical protein	R	POORLY CHARACTERIZED	RV0345	-1.29	0.076	Down
MSMEG_3312	hemerythrin HHE cation binding domain-containing protein	S	POORLY CHARACTERIZED		-2.01	0.082	Down
MSMEG_0249	integral membrane protein	R	POORLY CHARACTERIZED	RV0205	-1.54	0.082	Down
MSMEG_1772	hypothetical protein	S	POORLY CHARACTERIZED		-1.98	0.083	Down
MSMEG_3289	gp61 protein	R	POORLY CHARACTERIZED		-3.25	0.085	Down
MSMEG_3459	hypothetical protein	S	POORLY CHARACTERIZED	RV3737	-1.09	0.093	Down
MSMEG_0600	dehydrogenase	R	POORLY CHARACTERIZED		-1.87	0.097	Down
MSMEG_4743	hypothetical protein	S	POORLY CHARACTERIZED		-1.27	0.099	Down
MSMEG_6233	hypothetical protein	R	POORLY CHARACTERIZED		-1.83	0.107	Down
MSMEG_1932	MmpS3 protein	S	POORLY CHARACTERIZED	RV3209	-1.75	0.110	Down
MSMEG_1295	transthyretin	R	POORLY CHARACTERIZED		-2.01	0.111	Down
MSMEG_5218	hypothetical protein	R	POORLY CHARACTERIZED		-1.66	0.112	Down
MSMEG_1910	muconate cycloisomerase	R	POORLY CHARACTERIZED		-1.55	0.115	Down
MSMEG_5590	carboxylate-amine ligase	S	POORLY CHARACTERIZED		-2.56	0.119	Down
MSMEG_0671	S-(hydroxymethyl)glutathione dehydrogenase	R	POORLY CHARACTERIZED	RV1895	-1.85	0.122	Down
MSMEG_3254	RDD family protein	S	POORLY CHARACTERIZED		-2.27	0.125	Down
MSMEG_6667	hypothetical protein	S	POORLY CHARACTERIZED		-2.99	0.125	Down
MSMEG_3288	LysM domain-containing protein	S	POORLY CHARACTERIZED		-2.92	0.126	Down
MSMEG_4247	integral membrane protein	S	POORLY CHARACTERIZED	RV2181	-1.48	0.127	Down
MSMEG_5644	membrane protein	S	POORLY CHARACTERIZED	RV0180C	-1.38	0.127	Down
MSMEG_5961	glycosyl transferase family protein	R	POORLY CHARACTERIZED		-1.41	0.131	Down
MSMEG_6354	serine esterase, cutinase	R	POORLY CHARACTERIZED		-2.52	0.131	Down
MSMEG_4063	amidohydrolase	R	POORLY CHARACTERIZED		-1.60	0.132	Down
MSMEG_1001	acetyltransferase, gnat family protein	R	POORLY CHARACTERIZED		-1.49	0.132	Down
MSMEG_3255	DoxX subfamily protein	S	POORLY CHARACTERIZED		-2.05	0.133	Down
MSMEG_4707	non-heme bromoperoxidase BPO-A2	R	POORLY CHARACTERIZED		-1.71	0.133	Down
MSMEG_1782	oxidoreductase, short chain dehydrogenase/reductase	R	POORLY CHARACTERIZED		-2.43	0.134	Down
MSMEG_0066	early secretory antigenic target, 6 kDa	S	POORLY CHARACTERIZED	Rv3875	-1.58	0.135	Down
MSMEG_5402	dehydrogenase DhgA	R	POORLY CHARACTERIZED		-2.11	0.136	Down
MSMEG_5981	glycosyl transferase family protein	S	POORLY CHARACTERIZED		-1.52	0.148	Down
MSMEG_6569	hypothetical protein	S	POORLY CHARACTERIZED		-1.01	0.149	Down
MSMEG_0763	antibiotic transporter	R	POORLY CHARACTERIZED		-1.81	0.151	Down
MSMEG_2808	short-chain dehydrogenase/reductase SDR	R	POORLY CHARACTERIZED		-1.04	0.156	Down
MSMEG_3019	hypothetical protein	S	POORLY CHARACTERIZED	RV2568C	-1.36	0.159	Down
MSMEG_0382	transporter	R	POORLY CHARACTERIZED	Rv0450c	-1.38	0.162	Down
MSMEG_3580	antigen 85-C	R	POORLY CHARACTERIZED	Rv0129c	-1.65	0.166	Down
MSMEG_0697	integral membrane protein	S	POORLY CHARACTERIZED		-2.37	0.167	Down
MSMEG_2914	L-idonate 5-dehydrogenase	R	POORLY CHARACTERIZED		-1.21	0.168	Down
MSMEG_6616	S-(hydroxymethyl)glutathione dehydrogenase	R	POORLY CHARACTERIZED		-1.11	0.168	Down
MSMEG_3287	alpha/beta hydrolase	R	POORLY CHARACTERIZED		-1.52	0.169	Down
MSMEG_3374	hypothetical protein	S	POORLY CHARACTERIZED		-1.16	0.182	Down
wrbA	NAD(P)H:quinone oxidoreductase, type IV	R	POORLY CHARACTERIZED		-2.15	0.185	Down
MSMEG_2837	nitrate reductase NarB	R	POORLY CHARACTERIZED		-1.32	0.192	Down
MSMEG_6583	antigen 85-C	R	POORLY CHARACTERIZED		-1.17	0.193	Down
MSMEG_3420	gluconate 5-dehydrogenase	R	POORLY CHARACTERIZED		-1.17	0.195	Down
MSMEG_1299	oxidoreductase, 2OG-Fe(II) oxygenase	R	POORLY CHARACTERIZED		-1.18	0.197	Down
MSMEG_3022	transglycosylase associated protein	S	POORLY CHARACTERIZED		-1.64	0.204	Down
MSMEG_6610	hypothetical protein	R	POORLY CHARACTERIZED		-2.00	0.210	Down
MSMEG_3309	tryptophan synthase subunit beta	R	POORLY CHARACTERIZED		-1.42	0.222	Down
MSMEG_3083	nucleoside-diphosphate sugar epimerase	R	POORLY CHARACTERIZED		-1.39	0.236	Down
trmB	tRNA (guanine-N(7)-)-methyltransferase	R	POORLY CHARACTERIZED		-1.11	0.260	Down
MSMEG_1679	AmiB protein	R	POORLY CHARACTERIZED		-1.77	0.286	Down
MSMEG_5027	glyoxalase	S	POORLY CHARACTERIZED		-1.11	0.289	Down
MSMEG_5179	DoxX subfamily protein	S	POORLY CHARACTERIZED		-1.16	0.308	Down
MSMEG_2913	hydrolase	R	POORLY CHARACTERIZED		-1.46	0.329	Down
MSMEG_1379	hypothetical protein	R	POORLY CHARACTERIZED		-1.13	0.331	Down
MSMEG_2157	hypothetical protein	S	POORLY CHARACTERIZED		-1.81	0.335	Down
MSMEG_1680	hypothetical protein	S	POORLY CHARACTERIZED		-1.21	0.339	Down

Table S6. Genes Up / Down regulated in LIX80 (K55M Mut PknK Overexpression Strain at t=24 h)

Upregulated

For filtering upregulation we consider Geomean fold >=1 in treated sample.

Downregulated

For filtering downregulation we consider Geomean fold <=-1 in treated sample.

Expression fold values are provided in terms of log base 2

					LIX80 t24-1 vs. LIX70 t24-1		
GeneName	Product	COG_Code	Function	RV Number	Geomean_Fold	P-value	Regulation
sufB	FeS assembly protein SufB	O	CELLULAR PROCESSES AND SIGNALING	RV1461	2.34	0.262	Up
ftsZ	cell division protein FtsZ	D	CELLULAR PROCESSES AND SIGNALING	Rv2150c	1.07	0.003	Up
MSMEG_4253	glycoside hydrolase family protein	M	CELLULAR PROCESSES AND SIGNALING	Rv0557	1.04	0.003	Up
dnaK	molecular chaperone DnaK	O	CELLULAR PROCESSES AND SIGNALING	Rv0350	1.02	0.034	Up
MSMEG_3162	beta-lactamase	V	CELLULAR PROCESSES AND SIGNALING	Rv1497	1.11	0.212	Up
groEL	molecular chaperone GroEL	O	CELLULAR PROCESSES AND SIGNALING		1.56	0.252	Up
MSMEG_4309	low molecular weight protein-tyrosin	T	CELLULAR PROCESSES AND SIGNALING	Rv2234	1.00	0.269	Up
MSMEG_5611	spore protein	O	CELLULAR PROCESSES AND SIGNALING		1.21	0.292	Up
lexA	LexA repressor	T	CELLULAR PROCESSES AND SIGNALING	Rv2720	1.34	0.318	Up
groES	co-chaperonin GroES	O	CELLULAR PROCESSES AND SIGNALING		1.46	0.333	Up
MSMEG_5733	universal stress protein family protei	T	CELLULAR PROCESSES AND SIGNALING		1.09	0.359	Up
MSMEG_5372	sensor protein KdpD	T	CELLULAR PROCESSES AND SIGNALING	Rv1028c	1.10	0.535	Up
MSMEG_0787	extracellular solute-binding protein	T	CELLULAR PROCESSES AND SIGNALING	Rv0411c	1.08	0.573	Up
MSMEG_4154	transposase, Mutator family protein	L	INFORMATION STORAGE AND PROCESSING	RV3431C	3.17	0.010	Up
MSMEG_3121	DNA-binding protein	K	INFORMATION STORAGE AND PROCESSING	RV1460	2.95	0.175	Up
MSMEG_3959	transcriptional regulator	K	INFORMATION STORAGE AND PROCESSING		2.79	0.240	Up
MSMEG_5229	hypothetical protein	J	INFORMATION STORAGE AND PROCESSING		1.09	0.244	Up
MSMEG_6441	TetR family transcriptional regulator	K	INFORMATION STORAGE AND PROCESSING	Rv3855	3.23	0.253	Up
MSMEG_4492	amidase	J	INFORMATION STORAGE AND PROCESSING	Rv2363	1.14	0.330	Up
MSMEG_3960	transcriptional regulator	K	INFORMATION STORAGE AND PROCESSING		1.43	0.331	Up
MSMEG_6139	HTH-type transcriptional regulator	K	INFORMATION STORAGE AND PROCESSING	RV2912C	1.19	0.387	Up
MSMEG_2794	GntR family transcriptional regulator	K	INFORMATION STORAGE AND PROCESSING		1.68	0.417	Up
MSMEG_2989	LacI family transcriptional regulator	K	INFORMATION STORAGE AND PROCESSING		1.67	0.430	Up
dinP	DNA polymerase IV	L	INFORMATION STORAGE AND PROCESSING		1.52	0.453	Up
MSMEG_0471	LysR family transcriptional regulator	K	INFORMATION STORAGE AND PROCESSING		1.08	0.012	Up
MSMEG_4622	TetR family transcriptional regulator	K	INFORMATION STORAGE AND PROCESSING		1.09	0.125	Up
MSMEG_6077	CarD family transcriptional regulator	K	INFORMATION STORAGE AND PROCESSING	RV3583C	1.37	0.159	Up
MSMEG_2030	TetR family transcriptional regulator	K	INFORMATION STORAGE AND PROCESSING		1.37	0.168	Up
ruvC	Holliday junction resolvase	L	INFORMATION STORAGE AND PROCESSING	Rv2594c	1.43	0.176	Up
MSMEG_6009	TetR family transcriptional regulator	K	INFORMATION STORAGE AND PROCESSING	RV3557C	1.88	0.198	Up
MSMEG_2606	ArsR family transcriptional regulator	K	INFORMATION STORAGE AND PROCESSING		1.58	0.210	Up
ruvA	Holliday junction DNA helicase RuvA	L	INFORMATION STORAGE AND PROCESSING	Rv2593c	1.57	0.221	Up
MSMEG_5669	ISMsm2, transposase	L	INFORMATION STORAGE AND PROCESSING		1.13	0.246	Up
MSMEG_0296	MarR family transcriptional regulator	K	INFORMATION STORAGE AND PROCESSING		1.21	0.258	Up
MSMEG_6731	transcriptional regulatory protein	K	INFORMATION STORAGE AND PROCESSING		1.16	0.403	Up
MSMEG_5563	AraC family transcriptional regulator	K	INFORMATION STORAGE AND PROCESSING		1.28	0.419	Up
MSMEG_6223	TetR family transcriptional regulator	K	INFORMATION STORAGE AND PROCESSING		1.01	0.423	Up
MSMEG_5995	P450 heme-thiolate protein	Q	METABOLISM	Rv3545c	1.10	0.072	Up
MSMEG_3389	acetolactate synthase	H	METABOLISM		1.06	0.084	Up
MSMEG_2323	GntR family transcriptional regulator	E	METABOLISM		2.38	0.230	Up
MSMEG_3198	NUDIX family hydrolase	F	METABOLISM	RV1593C	2.23	0.232	Up
MSMEG_1821	acyl-CoA dehydrogenase	I	METABOLISM	Rv3274c	1.22	0.304	Up
MSMEG_4325	malonyl CoA-ACP transacylase	I	METABOLISM	Rv2243	2.01	0.321	Up
MSMEG_3269	sugar ABC transporter ATP-binding pr	G	METABOLISM		1.30	0.322	Up
MSMEG_3267	transporter	G	METABOLISM		1.24	0.327	Up
MSMEG_6225	proton antiporter efflux pump	G	METABOLISM		1.79	0.346	Up
MSMEG_6141	N-acyl-D-glutamate amidohydrolase	Q	METABOLISM	RV2913C	1.47	0.389	Up
MSMEG_5576	D-mannonate oxidoreductase	G	METABOLISM		1.17	0.459	Up
MSMEG_6440	monooxygenase, flavin-binding family	P	METABOLISM	Rv3854c	1.55	0.461	Up
cysD	sulfate adenylyltransferase subunit 2	H	METABOLISM	Rv1285	1.54	0.621	Up
MSMEG_6867	oligopeptide ABC transporter integra	P	METABOLISM	Rv3664c	1.41	0.002	Up
MSMEG_4646	pyruvate synthase	C	METABOLISM	RV2455C	1.01	0.003	Up
MSMEG_6330	prephenate dehydrogenase	E	METABOLISM	Rv3754	1.11	0.046	Up
MSMEG_4757	fatty acid synthase	I	METABOLISM	Rv2524c	1.50	0.115	Up
ald	alanine dehydrogenase	E	METABOLISM	Rv2780	1.15	0.126	Up
MSMEG_4386	ABC transporter permease	P	METABOLISM		1.55	0.170	Up
MSMEG_5491	acyl-CoA dehydrogenase	I	METABOLISM	Rv0975c	1.01	0.176	Up
MSMEG_3583	monooxygenase	C	METABOLISM		1.14	0.205	Up
thyA	thymidylate synthase	F	METABOLISM	Rv2764c	1.13	0.236	Up
MSMEG_2242	coniferyl aldehyde dehydrogenase	C	METABOLISM	RV0147	1.18	0.258	Up
pyrB	aspartate carbamoyltransferase	F	METABOLISM	Rv1380	1.07	0.258	Up
MSMEG_1511	oxidoreductase	C	METABOLISM		1.20	0.311	Up
MSMEG_1129	D-amino-acid dehydrogenase	E	METABOLISM		1.56	0.315	Up
MSMEG_3270	sn-glycerol-3-phosphate ABC transpo	G	METABOLISM		1.07	0.360	Up
MSMEG_5732	monooxygenase	C	METABOLISM		1.28	0.400	Up
MSMEG_4525	coproporphyrinogen III oxidase	H	METABOLISM	Rv2388c	1.04	0.425	Up
MSMEG_5572	sugar ABC transporter permease	G	METABOLISM		1.05	0.488	Up
MSMEG_4151	hypothetical protein	C	METABOLISM		1.02	0.503	Up
MSMEG_0305	acyltransferase domain-containing pr	I	METABOLISM	RV1428C	1.00	0.547	Up
MSMEG_0992	2-nitropropane dioxygenase	R	POORLY CHARACTERIZED	RV1533	2.05	0.057	Up
MSMEG_2755	hypothetical protein	S	POORLY CHARACTERIZED	RV2707	1.45	0.408	Up
MSMEG_5565	hypothetical protein	S	POORLY CHARACTERIZED		2.57	0.437	Up
MSMEG_3265	arabitol-phosphate dehydrogenase	R	POORLY CHARACTERIZED		1.03	0.474	Up
MSMEG_5329	hypothetical protein	S	POORLY CHARACTERIZED	RV3848	1.43	0.144	Up
MSMEG_6672	type I phosphodiesterase / nucleotid	R	POORLY CHARACTERIZED		1.20	0.156	Up
murQ	N-acetylmuramic acid 6-phosphate et	R	POORLY CHARACTERIZED		1.30	0.167	Up
MSMEG_6722	hypothetical protein	S	POORLY CHARACTERIZED	RV2237	1.16	0.188	Up
MSMEG_6742	hypothetical protein	R	POORLY CHARACTERIZED		1.62	0.206	Up
MSMEG_0530	short chain dehydrogenase	R	POORLY CHARACTERIZED		1.07	0.273	Up
MSMEG_5816	hypothetical protein	S	POORLY CHARACTERIZED		1.54	0.317	Up
MSMEG_1278	death-on-curing protein	R	POORLY CHARACTERIZED		1.94	0.328	Up
MSMEG_4029	hypothetical protein	R	POORLY CHARACTERIZED		1.15	0.340	Up
MSMEG_1045	integral membrane protein	S	POORLY CHARACTERIZED		1.41	0.355	Up

MSMEG_2975	metallo-beta-lactamase	R	POORLY CHARACTERIZED	RV2581C	1.07	0.370	Up
MSMEG_2032	hypothetical protein	R	POORLY CHARACTERIZED		1.64	0.410	Up
MSMEG_6870	creatinine amidohydrolase	R	POORLY CHARACTERIZED		1.03	0.559	Up
MSMEG_5479	type I antifreeze protein	S	POORLY CHARACTERIZED	RV0991C	1.40	0.581	Up
					LIX80 t24-1 vs. LIX70 t24-1		
GeneName	Product	COG_Code	Function	RV Number	Geomean_Fold	P-value	Regulation
MSMEG_1803	RsbW protein	T	CELLULAR PROCESSES AND SIGNALING	Rv3287c	-1.58	0.006	Down
MSMEG_0670	FAD dependent oxidoreductase	O	CELLULAR PROCESSES AND SIGNALING		-1.85	0.017	Down
cysT	sulfate ABC transporter permease Cys	O	CELLULAR PROCESSES AND SIGNALING	Rv2399c	-1.01	0.041	Down
MSMEG_1097	glycosyl transferase family protein	M	CELLULAR PROCESSES AND SIGNALING		-2.05	0.062	Down
MSMEG_6822	beta-lactamase	V	CELLULAR PROCESSES AND SIGNALING		-1.84	0.062	Down
MSMEG_3541	cytochrome C biogenesis protein tran	O	CELLULAR PROCESSES AND SIGNALING	RV2877C	-1.33	0.069	Down
MSMEG_1131	tryptophan-rich sensory protein	T	CELLULAR PROCESSES AND SIGNALING		-1.23	0.087	Down
MSMEG_0933	hypothetical protein	M	CELLULAR PROCESSES AND SIGNALING	Rv0486	-1.94	0.136	Down
MSMEG_1315	transporter small conductance mecha	M	CELLULAR PROCESSES AND SIGNALING		-2.18	0.138	Down
MSMEG_5661	ABC transporter ATP-binding protein	V	CELLULAR PROCESSES AND SIGNALING		-1.03	0.146	Down
MSMEG_6541	anti-sigma factor antagonist	T	CELLULAR PROCESSES AND SIGNALING		-2.26	0.164	Down
MSMEG_0449	transporter major facilitator family pr	T	CELLULAR PROCESSES AND SIGNALING		-1.98	0.189	Down
MSMEG_6129	hypothetical protein	T	CELLULAR PROCESSES AND SIGNALING		-1.21	0.194	Down
MSMEG_5496	MscS mechanosensitive ion channel	M	CELLULAR PROCESSES AND SIGNALING		-1.97	0.240	Down
MSMEG_5474	universal stress protein family protein	T	CELLULAR PROCESSES AND SIGNALING		-1.73	0.260	Down
MSMEG_2804	two-component system sensor kinase	T	CELLULAR PROCESSES AND SIGNALING		-1.76	0.281	Down
MSMEG_2924	permease binding-protein componer	M	CELLULAR PROCESSES AND SIGNALING		-1.11	0.029	Down
MSMEG_2798	hypothetical protein	O	CELLULAR PROCESSES AND SIGNALING		-1.27	0.030	Down
MSMEG_4211	two component system histidine kina	T	CELLULAR PROCESSES AND SIGNALING		-1.30	0.039	Down
MSMEG_5660	ABC transporter ATP-binding protein	V	CELLULAR PROCESSES AND SIGNALING		-1.15	0.122	Down
MSMEG_5659	ABC transporter ATP-binding protein	V	CELLULAR PROCESSES AND SIGNALING	RV0194	-1.37	0.210	Down
MSMEG_2433	D-alanyl-D-alanine carboxypeptidase	M	CELLULAR PROCESSES AND SIGNALING		-1.38	0.217	Down
MSMEG_5554	ANTAR domain-containing protein	T	CELLULAR PROCESSES AND SIGNALING		-1.03	0.680	Down
MSMEG_0523	DNA-binding protein	K	INFORMATION STORAGE AND PROCESSING		-2.05	0.000	Down
MSMEG_6903	PadR family transcriptional regulator	K	INFORMATION STORAGE AND PROCESSING	RV0047C	-1.18	0.003	Down
MSMEG_1771	methylase	J	INFORMATION STORAGE AND PROCESSING		-2.44	0.005	Down
MSMEG_0265	uracil DNA glycosylase superfamily pr	L	INFORMATION STORAGE AND PROCESSING		-2.37	0.008	Down
MSMEG_4620	NAD-dependent deacetylase 1	K	INFORMATION STORAGE AND PROCESSING		-1.75	0.009	Down
MSMEG_1909	HTH-type transcriptional regulator Yn	K	INFORMATION STORAGE AND PROCESSING		-1.96	0.016	Down
MSMEG_6356	AraC family transcriptional regulator	K	INFORMATION STORAGE AND PROCESSING		-1.99	0.024	Down
MSMEG_1804	RNA polymerase sigma factor SigF	K	INFORMATION STORAGE AND PROCESSING	Rv3286c	-1.85	0.033	Down
MSMEG_1784	type I topoisomerase	L	INFORMATION STORAGE AND PROCESSING		-1.76	0.097	Down
MSMEG_3274	MerR family transcriptional regulator	K	INFORMATION STORAGE AND PROCESSING		-1.57	0.122	Down
ung	uracil-DNA glycosylase	L	INFORMATION STORAGE AND PROCESSING	Rv2976c	-1.39	0.131	Down
MSMEG_3885	DEAD/DEAH box helicase	L	INFORMATION STORAGE AND PROCESSING	Rv2092c	-1.34	0.136	Down
MSMEG_5570	ATP-dependent DNA ligase	L	INFORMATION STORAGE AND PROCESSING	RV0938	-1.52	0.152	Down
MSMEG_5399	ATP-dependent DNA helicase RecQ	L	INFORMATION STORAGE AND PROCESSING		-1.58	0.163	Down
MSMEG_6807	TetR family transcriptional regulator	K	INFORMATION STORAGE AND PROCESSING		-1.25	0.232	Down
MSMEG_2105	ATP dependent DNA ligase	L	INFORMATION STORAGE AND PROCESSING		-1.36	0.345	Down
phoP	DNA-binding response regulator PhoP	K	INFORMATION STORAGE AND PROCESSING	Rv0757	-1.20	0.008	Down
MSMEG_0535	GntR family transcriptional regulator	K	INFORMATION STORAGE AND PROCESSING		-1.78	0.009	Down
MSMEG_3191	hypothetical protein	J	INFORMATION STORAGE AND PROCESSING	RV1571	-1.09	0.015	Down
MSMEG_5542	transcriptional regulator	K	INFORMATION STORAGE AND PROCESSING		-1.73	0.022	Down
MSMEG_4912	helicase	L	INFORMATION STORAGE AND PROCESSING	Rv1329c	-1.11	0.052	Down
MSMEG_5019	regulatory protein	K	INFORMATION STORAGE AND PROCESSING		-1.26	0.100	Down
MSMEG_2849	transcriptional regulatory protein	K	INFORMATION STORAGE AND PROCESSING		-1.13	0.141	Down
MSMEG_5177	TetR family transcriptional regulator	K	INFORMATION STORAGE AND PROCESSING		-1.29	0.176	Down
MSMEG_6888	transposase subunit	L	INFORMATION STORAGE AND PROCESSING		-1.52	0.240	Down
MSMEG_1494	transcriptional regulatory protein De	K	INFORMATION STORAGE AND PROCESSING		-1.15	0.446	Down
MSMEG_5784	transcriptional regulatory protein	K	INFORMATION STORAGE AND PROCESSING	RV0818	-1.12	0.473	Down
MSMEG_0886	serine/threonine protein kinase	L	INFORMATION STORAGE AND PROCESSING		-1.15	0.489	Down
MSMEG_2128	malonyl CoA decarboxylase	J	INFORMATION STORAGE AND PROCESSING		-1.34	0.546	Down
MSMEG_4618	isochorismatase	Q	METABOLISM		-1.87	0.012	Down
MSMEG_6467	starvation-induced DNA protecting pr	P	METABOLISM		-1.77	0.023	Down
MSMEG_0686	oxidoreductase	C	METABOLISM		-2.84	0.026	Down
treS	trehalose synthase	G	METABOLISM	Rv0126	-1.71	0.026	Down
MSMEG_6769	transporter monovalent cation:proto	P	METABOLISM		-2.96	0.029	Down
MSMEG_2855	virulence factor Mce family protein	Q	METABOLISM		-1.84	0.029	Down
MSMEG_0374	glycolate oxidase subunit	C	METABOLISM		-1.19	0.032	Down
MSMEG_4565	oxidoreductase	C	METABOLISM		-2.36	0.032	Down
MSMEG_0764	Na+/H+ antiporter	P	METABOLISM		-2.10	0.035	Down
MSMEG_0685	oxidoreductase, molybdopterin-bind	C	METABOLISM	RV0375C	-2.53	0.040	Down
MSMEG_5559	metabolite/sugar transporter	G	METABOLISM	Rv3331	-2.96	0.041	Down
MSMEG_1794	dehydrogenase	C	METABOLISM		-2.79	0.041	Down
cysA	sulfate ABC transporter ATP-binding p	P	METABOLISM		-2.19	0.044	Down
MSMEG_1847	4Fe-4S ferredoxin	C	METABOLISM		-1.58	0.044	Down
MSMEG_4355	peptide ABC transporter permease	P	METABOLISM		-2.11	0.047	Down
MSMEG_1062	O-succinylbenzoic acid--CoA ligase	Q	METABOLISM	Rv0542c	-1.66	0.048	Down
MSMEG_2925	permease membrane component	E	METABOLISM		-1.32	0.048	Down
MSMEG_1695	phosphoglucomutase/phosphomann	G	METABOLISM	Rv3308	-1.85	0.053	Down
MSMEG_6663	C5-O-methyltransferase	H	METABOLISM		-1.75	0.058	Down
MSMEG_0637	iron-sulfur binding oxidoreductase	E	METABOLISM		-2.96	0.061	Down
MSMEG_5721	acetyl-CoA acetyltransferase	I	METABOLISM	Rv0859	-1.73	0.064	Down
MSMEG_2927	ABC transporter permease	E	METABOLISM		-1.51	0.067	Down
MSMEG_6727	amino acid permease	E	METABOLISM		-1.98	0.067	Down
MSMEG_0267	esterase	I	METABOLISM		-2.57	0.070	Down
MSMEG_5251	hypothetical protein	P	METABOLISM		-1.54	0.074	Down
MSMEG_2346	phytoene synthase	I	METABOLISM		-1.74	0.075	Down
MSMEG_1775	cytochrome P450 monooxygenase	Q	METABOLISM		-2.46	0.076	Down
MSMEG_6768	halogenase	C	METABOLISM		-2.54	0.088	Down
MSMEG_3536	sugar transporter	G	METABOLISM		-1.57	0.095	Down
MSMEG_5473	transporter	E	METABOLISM	Rv0522	-1.10	0.098	Down
MSMEG_2347	phytoene dehydrogenase	Q	METABOLISM		-2.21	0.103	Down
MSMEG_3460	ferric uptake regulation protein	P	METABOLISM		-1.81	0.105	Down
glgB	glycogen branching protein	G	METABOLISM	Rv1326c	-1.47	0.105	Down
MSMEG_2112	hypothetical protein	E	METABOLISM		-2.43	0.109	Down
fni	isopentenyl pyrophosphate isomeras	C	METABOLISM		-2.30	0.111	Down
MSMEG_0264	transmembrane transporter	G	METABOLISM		-1.32	0.119	Down
MSMEG_6514	trehalose synthase-fused maltokinase	G	METABOLISM	RV0127	-1.61	0.125	Down

MSMEG_0375	phospholipase D	I	METABOLISM		-2.51	0.125	Down
MSMEG_1296	uricase	Q	METABOLISM		-2.13	0.134	Down
MSMEG_6664	methylenetetrahydrofolate reductas	E	METABOLISM		-2.20	0.142	Down
MSMEG_0968	cytochrome P450	Q	METABOLISM		-2.53	0.143	Down
MSMEG_5799	nucleoside-diphosphate-sugar epime	G	METABOLISM		-1.53	0.145	Down
MSMEG_3421	inner membrane metabolite transpor	G	METABOLISM		-1.33	0.149	Down
MSMEG_6213	manganese containing catalase	P	METABOLISM		-2.51	0.155	Down
MSMEG_1477	major facilitator superfamily protein	G	METABOLISM		-2.18	0.160	Down
malQ	4-alpha-glucanotransferase	G	METABOLISM	Rv1781c	-2.29	0.161	Down
MSMEG_6615	hypothetical protein	Q	METABOLISM		-2.29	0.161	Down
glgX	glycogen debranching protein GlgX	G	METABOLISM		-1.81	0.165	Down
katA	catalase KatA	P	METABOLISM		-2.43	0.166	Down
xfp	phosphoketolase	G	METABOLISM		-1.53	0.167	Down
MSMEG_5189	oxidoreductase	G	METABOLISM		-1.39	0.168	Down
MSMEG_5358	acetamidase/formamidase	C	METABOLISM		-1.63	0.171	Down
MSMEG_2035	amine oxidase	E	METABOLISM	Rv3170	-1.48	0.172	Down
MSMEG_5400	dehydrogenase	Q	METABOLISM		-3.19	0.179	Down
MSMEG_2122	dihydroxyacetone kinase subunit Dha	G	METABOLISM		-1.42	0.191	Down
MSMEG_4916	alpha-amylase	G	METABOLISM	Rv1327c	-1.57	0.191	Down
MSMEG_1112	aconitate hydratase	C	METABOLISM		-2.37	0.197	Down
cysW	sulfate ABC transporter permease Cys	P	METABOLISM	Rv2398c	-2.38	0.203	Down
MSMEG_5826	pyruvate decarboxylase	H	METABOLISM		-2.37	0.210	Down
MSMEG_0684	aldehyde oxidase	C	METABOLISM		-1.90	0.210	Down
MSMEG_0266	arginine decarboxylase	E	METABOLISM	RV2531C	-1.92	0.214	Down
MSMEG_3304	succinate semialdehyde dehydrogena	C	METABOLISM		-2.15	0.215	Down
MSMEG_0357	transmembrane transporter	G	METABOLISM		-2.15	0.217	Down
MSMEG_4318	hypothetical protein	P	METABOLISM		-2.08	0.218	Down
MSMEG_6740	1-aminocyclopropane-1-carboxylate	E	METABOLISM		-1.16	0.221	Down
MSMEG_4356	ABC transporter	P	METABOLISM	Rv3664c	-1.78	0.229	Down
MSMEG_5605	cytochrome bd ubiquinol oxidase sub	C	METABOLISM		-1.38	0.251	Down
MSMEG_2037	hypothetical protein	C	METABOLISM		-1.56	0.266	Down
MSMEG_0782	class III aminotransferase	E	METABOLISM		-1.08	0.281	Down
catA	catechol 1,2-dioxygenase	Q	METABOLISM		-1.03	0.325	Down
MSMEG_0432	bifunctional uroporphyrinogen-III syn	H	METABOLISM	RV0260C	-1.10	0.335	Down
MSMEG_1846	polysulfide reductase NrfD	P	METABOLISM		-1.39	0.346	Down
MSMEG_0614	methyltransferase	Q	METABOLISM	RV0281	-1.44	0.366	Down
MSMEG_1292	FAD binding domain-containing prote	C	METABOLISM		-1.26	0.367	Down
MSMEG_1886	Fatty acid desaturase	I	METABOLISM	Rv3229c	-1.16	0.369	Down
MSMEG_1742	oxidoreductase	C	METABOLISM		-1.57	0.389	Down
MSMEG_3007	succinate-semialdehyde dehydrogena	C	METABOLISM		-1.32	0.407	Down
glgC	glucose-1-phosphate adenylyltransfer	G	METABOLISM	Rv1213	-1.30	0.000	Down
MSMEG_1432	acetylcholinesterase	I	METABOLISM		-1.28	0.002	Down
MSMEG_2926	glycine betaine/carnitine/choline tra	E	METABOLISM		-1.14	0.020	Down
MSMEG_5341	dipeptidyl aminopeptidase/acylamin	I	METABOLISM		-1.08	0.041	Down
MSMEG_4034	NAD dependent epimerase/dehydrat	G	METABOLISM		-1.25	0.043	Down
MSMEG_6230	acyltransferase	I	METABOLISM		-1.59	0.048	Down
MSMEG_0604	glyoxylate reductase	E	METABOLISM		-1.00	0.051	Down
MSMEG_5504	hypothetical protein	G	METABOLISM		-1.17	0.065	Down
MSMEG_3108	ABC transporter ATPase	G	METABOLISM		-1.12	0.074	Down
oppC	ABC transporter permease	P	METABOLISM	RV1282C	-1.11	0.088	Down
MSMEG_6235	thiopurine S-methyltransferase (tpmt	H	METABOLISM	RV3699	-1.22	0.090	Down
MSMEG_1780	hypothetical protein	P	METABOLISM		-1.11	0.091	Down
MSMEG_3273	glutamyl aminopeptidase, M42 family	G	METABOLISM		-1.33	0.102	Down
MSMEG_4719	peptidase S9, prolyl oligopeptidase	E	METABOLISM		-1.20	0.105	Down
MSMEG_3444	choline dehydrogenase	E	METABOLISM		-1.46	0.115	Down
MSMEG_1361	alpha-mannosidase	G	METABOLISM	RV0648	-1.27	0.116	Down
MSMEG_0762	cytochrome P450	Q	METABOLISM		-1.19	0.116	Down
MSMEG_1132	FAD binding domain-containing prote	C	METABOLISM	RV0561C	-1.33	0.126	Down
MSMEG_6471	glycine/D-amino acid oxidase	E	METABOLISM		-1.05	0.137	Down
MSMEG_1572	GntR family transcriptional regulator	E	METABOLISM		-1.29	0.141	Down
MSMEG_6312	cytochrome P450	Q	METABOLISM		-1.12	0.153	Down
MSMEG_5715	hypothetical protein	C	METABOLISM		-1.16	0.161	Down
MSMEG_4708	methyltransferase	H	METABOLISM	RV0089	-1.04	0.170	Down
MSMEG_6321	glycerol dehydratase large subunit	Q	METABOLISM		-1.08	0.176	Down
MSMEG_4857	hypothetical protein	C	METABOLISM		-1.05	0.181	Down
MSMEG_5592	hypothetical protein	C	METABOLISM		-1.28	0.184	Down
MSMEG_2982	periplasmic binding protein	E	METABOLISM		-1.45	0.193	Down
MSMEG_6735	amino acid permease	E	METABOLISM		-1.26	0.223	Down
MSMEG_5163	glycine/betaine ABC transporter peri	E	METABOLISM		-1.30	0.283	Down
MSMEG_4077	enoyl-CoA hydratase	I	METABOLISM		-1.06	0.296	Down
phoU	phosphate transporter regulatory pro	P	METABOLISM		-1.05	0.302	Down
MSMEG_3620	hypothetical protein	C	METABOLISM	RV1855C	-1.06	0.306	Down
MSMEG_4076	butyryl-CoA dehydrogenase	I	METABOLISM		-1.69	0.311	Down
sthA	soluble pyridine nucleotide transhyd	C	METABOLISM	Rv2713	-1.03	0.313	Down
MSMEG_0818	transporter major facilitator family pr	G	METABOLISM		-1.23	0.322	Down
MSMEG_1204	3-oxoacyl-ACP synthase	Q	METABOLISM		-1.07	0.361	Down
MSMEG_2208	acyl-CoA dehydrogenase	I	METABOLISM		-1.06	0.388	Down
MSMEG_1705	D-xylose transport ATP-binding prote	G	METABOLISM		-1.09	0.392	Down
MSMEG_5022	flavin-containing monooxygenase FM	P	METABOLISM		-1.08	0.491	Down
MSMEG_4570	hypothetical protein	S	POORLY CHARACTERIZED	RV2411C	-1.49	0.002	Down
MSMEG_6667	hypothetical protein	S	POORLY CHARACTERIZED		-3.38	0.010	Down
MSMEG_5616	glyoxalase/bleomycin resistance prot	R	POORLY CHARACTERIZED	RV0911	-1.95	0.011	Down
MSMEG_3689	sodium:solute symporter	R	POORLY CHARACTERIZED		-1.31	0.015	Down
MSMEG_5590	carboxylate-amine ligase	S	POORLY CHARACTERIZED		-2.97	0.016	Down
MSMEG_4247	integral membrane protein	S	POORLY CHARACTERIZED	RV2181	-1.84	0.017	Down
MSMEG_5218	hypothetical protein	R	POORLY CHARACTERIZED		-2.08	0.021	Down
MSMEG_0249	integral membrane protein	R	POORLY CHARACTERIZED	RV0205	-1.76	0.024	Down
MSMEG_3289	gp61 protein	R	POORLY CHARACTERIZED		-3.40	0.027	Down
MSMEG_5237	hypothetical protein	R	POORLY CHARACTERIZED	RV1101C	-1.69	0.034	Down
MSMEG_1806	hypothetical protein	R	POORLY CHARACTERIZED	RV0345	-1.63	0.035	Down
MSMEG_1295	transthyretin	R	POORLY CHARACTERIZED		-1.83	0.040	Down
MSMEG_0600	dehydrogenase	R	POORLY CHARACTERIZED		-2.32	0.042	Down
MSMEG_3255	DoxX subfamily protein	S	POORLY CHARACTERIZED		-2.79	0.043	Down
MSMEG_3083	nucleoside-diphosphate sugar epime	R	POORLY CHARACTERIZED		-1.70	0.045	Down
MSMEG_0697	integral membrane protein	S	POORLY CHARACTERIZED		-3.16	0.057	Down
MSMEG_3593	hypothetical protein	S	POORLY CHARACTERIZED		-3.08	0.060	Down
MSMEG_5402	dehydrogenase DhgA	R	POORLY CHARACTERIZED		-2.62	0.061	Down

MSMEG_1768	hypothetical protein	R	POORLY CHARACTERIZED	RV0925C	-2.37	0.066	Down
MSMEG_6116	OHCU decarboxylase	S	POORLY CHARACTERIZED		-1.74	0.071	Down
MSMEG_1782	oxidoreductase, short chain dehydrog	R	POORLY CHARACTERIZED		-3.08	0.071	Down
MSMEG_6542	B12-binding domain-containing prote	R	POORLY CHARACTERIZED		-1.95	0.072	Down
MSMEG_6233	hypothetical protein	R	POORLY CHARACTERIZED		-2.15	0.072	Down
MSMEG_0671	S-(hydroxymethyl)glutathione dehydı	R	POORLY CHARACTERIZED	RV1895	-2.26	0.082	Down
MSMEG_0536	intracellular protease PfpI family prot	R	POORLY CHARACTERIZED		-1.63	0.095	Down
MSMEG_1001	acetyltransferase, gnat family protein	R	POORLY CHARACTERIZED		-2.11	0.096	Down
MSMEG_3287	alpha/beta hydrolase	R	POORLY CHARACTERIZED		-1.44	0.097	Down
MSMEG_3022	transglycosylase associated protein	S	POORLY CHARACTERIZED		-2.46	0.099	Down
MSMEG_2837	nitrate reductase NarB	R	POORLY CHARACTERIZED		-1.70	0.108	Down
MSMEG_1772	hypothetical protein	S	POORLY CHARACTERIZED		-2.05	0.108	Down
MSMEG_6151	alpha/beta hydrolase	R	POORLY CHARACTERIZED	RV1190	-2.29	0.113	Down
MSMEG_3312	hemerythrin HHE cation binding dom	S	POORLY CHARACTERIZED		-1.96	0.114	Down
MSMEG_0763	antibiotic transporter	R	POORLY CHARACTERIZED		-2.24	0.116	Down
MSMEG_4707	non-heme bromoperoxidase BPO-A2	R	POORLY CHARACTERIZED		-1.96	0.144	Down
MSMEG_1910	muconate cycloisomerase	R	POORLY CHARACTERIZED		-1.55	0.145	Down
MSMEG_3420	gluconate 5-dehydrogenase	R	POORLY CHARACTERIZED		-1.28	0.147	Down
MSMEG_6616	S-(hydroxymethyl)glutathione dehydı	R	POORLY CHARACTERIZED		-1.17	0.150	Down
MSMEG_3254	RDD family protein	S	POORLY CHARACTERIZED		-2.61	0.159	Down
wrbA	NAD(P)H:quinone oxidoreductase, ty	R	POORLY CHARACTERIZED		-2.65	0.169	Down
MSMEG_6354	serine esterase, cutinase	R	POORLY CHARACTERIZED		-2.88	0.178	Down
MSMEG_2808	short-chain dehydrogenase/reductas	R	POORLY CHARACTERIZED		-1.10	0.196	Down
MSMEG_6610	hypothetical protein	R	POORLY CHARACTERIZED		-2.25	0.238	Down
MSMEG_4743	hypothetical protein	S	POORLY CHARACTERIZED		-1.14	0.248	Down
MSMEG_3288	LysM domain-containing protein	S	POORLY CHARACTERIZED		-2.89	0.310	Down
MSMEG_2157	hypothetical protein	S	POORLY CHARACTERIZED		-2.20	0.365	Down
MSMEG_6614	integral membrane protein	R	POORLY CHARACTERIZED		-1.06	0.003	Down
MSMEG_0356	acetyltransferase	R	POORLY CHARACTERIZED		-1.08	0.024	Down
MSMEG_1817	transmembrane protein	S	POORLY CHARACTERIZED	RV3277	-1.61	0.033	Down
MSMEG_2870	oxygenase KshA	R	POORLY CHARACTERIZED		-1.10	0.046	Down
MSMEG_1655	alpha/beta hydrolase	R	POORLY CHARACTERIZED		-1.47	0.048	Down
moxR	ATPase, MoxR family protein	R	POORLY CHARACTERIZED	Rv1479	-1.43	0.060	Down
MSMEG_2942	glyoxalase/bleomycin resistance prot	R	POORLY CHARACTERIZED		-1.53	0.085	Down
MSMEG_6000	short chain dehydrogenase	R	POORLY CHARACTERIZED	RV3549C	-1.01	0.094	Down
MSMEG_6069	CobW/P47K domain-containing prote	R	POORLY CHARACTERIZED	RV0106	-1.34	0.096	Down
MSMEG_2589	hypothetical protein	S	POORLY CHARACTERIZED		-1.16	0.101	Down
MSMEG_5834	metallo-beta-lactamase superfamily p	R	POORLY CHARACTERIZED	RV0786C	-1.28	0.116	Down
MSMEG_2066	hypothetical protein	R	POORLY CHARACTERIZED		-1.12	0.138	Down
MSMEG_6150	4-carboxymuconolactone decarboxyl	S	POORLY CHARACTERIZED		-1.01	0.143	Down
MSMEG_0780	phosphotransferase enzyme family pr	R	POORLY CHARACTERIZED		-1.88	0.203	Down
MSMEG_2947	polyketide cyclase/dehydrase	R	POORLY CHARACTERIZED	RV1716	-1.19	0.262	Down
MSMEG_1844	hypothetical protein	S	POORLY CHARACTERIZED		-1.32	0.308	Down
MSMEG_1297	hydroxydechloroatrazine ethylamino	R	POORLY CHARACTERIZED		-1.07	0.356	Down
MSMEG_2076	hypothetical protein	S	POORLY CHARACTERIZED	Rv1631	-1.14	0.404	Down
MSMEG_5020	acetoin(diacetyl) reductase	R	POORLY CHARACTERIZED		-1.07	0.420	Down
MSMEG_0437	hypothetical protein	S	POORLY CHARACTERIZED		-1.12	0.461	Down

Table S7. Genes Up / Down regulated in LIX 79 (WT PknK Overexpression Strain at t=96 h)

Upregulated
For filtering upregulation we consider Geomean fold >=1 in treated sample.

Downregulated
For filtering downregulation we consider Geomean fold <=-1 in treated sample.

Expression fold values are provided in terms of log base 2

					LIX79 t96-1 vs. LIX70 t96-1		
GeneName	Product	COG_Code	Function	RV Number	Geomean_Fold	P-value	Regulation
MSMEG_3246	response regulator	T	CELLULAR PROCESSES AND SIGNALING		1.42	0.002	Up
MSMEG_2798	hypothetical protein	O	CELLULAR PROCESSES AND SIGNALING		1.32	0.002	Up
MSMEG_3895	proteasome subunit beta	O	CELLULAR PROCESSES AND SIGNALING	Rv2110c	1.17	0.003	Up
MSMEG_4303	methyltransferase	O	CELLULAR PROCESSES AND SIGNALING		1.89	0.005	Up
MSMEG_0877	dihydrodipicolinate synthase	M	CELLULAR PROCESSES AND SIGNALING		1.50	0.006	Up
MSMEG_3235	amino acid ABC transporter	T	CELLULAR PROCESSES AND SIGNALING		1.09	0.012	Up
MSMEG_0311	glycosyltransferase	M	CELLULAR PROCESSES AND SIGNALING	RV0225	1.27	0.012	Up
MSMEG_3647	forkhead-associated protein	T	CELLULAR PROCESSES AND SIGNALING	RV1827	1.20	0.024	Up
MSMEG_6366	O-antigen export system, ATP-binding protei	M	CELLULAR PROCESSES AND SIGNALING	Rv3781	1.48	0.028	Up
MSMEG_0699	hypothetical protein	O	CELLULAR PROCESSES AND SIGNALING		2.13	0.037	Up
MSMEG_5738	hypothetical protein	O	CELLULAR PROCESSES AND SIGNALING		1.39	0.041	Up
MSMEG_0234	metallopeptidase	O	CELLULAR PROCESSES AND SIGNALING	RV0198C	1.11	0.060	Up
rfbD	dTDP-4-dehydrorhamnose reductase	M	CELLULAR PROCESSES AND SIGNALING	Rv3266c	1.64	0.060	Up
MSMEG_2421	hypothetical protein	O	CELLULAR PROCESSES AND SIGNALING	RV2923C	2.45	0.075	Up
MSMEG_6091	negative regulator of genetic competence Cl	O	CELLULAR PROCESSES AND SIGNALING		1.25	0.076	Up
MSMEG_4231	UDP-N-acetylmuramoyl-tripeptide--D-alanyl-	M	CELLULAR PROCESSES AND SIGNALING	Rv2157c	1.71	0.094	Up
MSMEG_5554	ANTAR domain-containing protein	T	CELLULAR PROCESSES AND SIGNALING		1.20	0.095	Up
MSMEG_5009	ABC transporter	V	CELLULAR PROCESSES AND SIGNALING	RV1272C	1.06	0.095	Up
MSMEG_1661	D-alanyl-D-alanine carboxypeptidase	M	CELLULAR PROCESSES AND SIGNALING	Rv3330	1.17	0.106	Up
MSMEG_3884	hypothetical protein	D	CELLULAR PROCESSES AND SIGNALING	RV2091C	1.37	0.106	Up
MSMEG_2806	two-component system response regulator	T	CELLULAR PROCESSES AND SIGNALING		1.38	0.107	Up
MSMEG_6554	ABC transporter ATP-binding protein	V	CELLULAR PROCESSES AND SIGNALING	RV1348	1.26	0.117	Up
MSMEG_2665	hypothetical protein	O	CELLULAR PROCESSES AND SIGNALING	RV2772C	1.46	0.125	Up
MSMEG_4968	two-component system sensor kinase	T	CELLULAR PROCESSES AND SIGNALING		1.47	0.127	Up
MSMEG_2416	hypothetical protein	D	CELLULAR PROCESSES AND SIGNALING	RV2927C	2.91	0.128	Up
secG	preprotein translocase subunit SecG	U	CELLULAR PROCESSES AND SIGNALING	Rv1440	1.04	0.129	Up
MSMEG_3780	adenylate and guanylate cyclase catalytic dor	T	CELLULAR PROCESSES AND SIGNALING	RV1647	1.21	0.132	Up
MSMEG_2433	D-alanyl-D-alanine carboxypeptidase	M	CELLULAR PROCESSES AND SIGNALING		1.20	0.138	Up
MSMEG_6105	cell division protein	O	CELLULAR PROCESSES AND SIGNALING	Rv3610c	1.31	0.140	Up
MSMEG_0100	phosphotyrosine protein phosphatase ptpb	T	CELLULAR PROCESSES AND SIGNALING	Rv0153c	1.80	0.153	Up
MSMEG_3477	inv protein	M	CELLULAR PROCESSES AND SIGNALING	RV1566C	1.24	0.159	Up
MSMEG_5006	phosphohistidine phosphatase	T	CELLULAR PROCESSES AND SIGNALING	RV1276C	1.48	0.164	Up
MSMEG_5259	hypothetical protein	O	CELLULAR PROCESSES AND SIGNALING	RV1084	1.09	0.166	Up
secD	preprotein translocase subunit SecD	U	CELLULAR PROCESSES AND SIGNALING	Rv2587c	1.59	0.170	Up
MSMEG_2731	DNA repair ATPase	D	CELLULAR PROCESSES AND SIGNALING	RV2731	2.08	0.176	Up
MSMEG_2804	two-component system sensor kinase	T	CELLULAR PROCESSES AND SIGNALING		1.63	0.176	Up
MSMEG_6238	two-component system sensor kinase	T	CELLULAR PROCESSES AND SIGNALING		1.10	0.177	Up
MSMEG_2087	transporter small conductance mechanosens	M	CELLULAR PROCESSES AND SIGNALING	RV3104C	1.16	0.178	Up
sppA	signal peptide peptidase SppA, 67K type	U	CELLULAR PROCESSES AND SIGNALING	Rv0724	1.59	0.182	Up
MSMEG_1854	valosin containing protein-1	O	CELLULAR PROCESSES AND SIGNALING		2.39	0.185	Up
MSMEG_3902	ATPase AAA	O	CELLULAR PROCESSES AND SIGNALING		1.58	0.186	Up
lgt	prolipoprotein diacylglyceryl transferase	M	CELLULAR PROCESSES AND SIGNALING		1.73	0.193	Up
crcB	camphor resistance protein CrcB	D	CELLULAR PROCESSES AND SIGNALING		1.82	0.196	Up
MSMEG_0067	hypothetical protein	D	CELLULAR PROCESSES AND SIGNALING		1.24	0.199	Up
MSMEG_3213	hypothetical protein	V	CELLULAR PROCESSES AND SIGNALING	RV3263	1.70	0.208	Up
MSMEG_0064	PPE family protein	N	CELLULAR PROCESSES AND SIGNALING	Rv3873	1.83	0.213	Up
MSMEG_4217	DivIVA protein	D	CELLULAR PROCESSES AND SIGNALING		1.04	0.214	Up
MSMEG_3146	invasin 1	M	CELLULAR PROCESSES AND SIGNALING	RV1478	1.42	0.223	Up
MSMEG_0617	ftsk/SpoIIIE family protein	D	CELLULAR PROCESSES AND SIGNALING		2.40	0.226	Up
msrB	methionine-R-sulfoxide reductase	O	CELLULAR PROCESSES AND SIGNALING	RV2674	1.47	0.234	Up
MSMEG_5641	glycoside hydrolase family protein	M	CELLULAR PROCESSES AND SIGNALING		1.10	0.236	Up
MSMEG_3239	two-component system sensor kinase	T	CELLULAR PROCESSES AND SIGNALING		1.66	0.248	Up
MSMEG_0993	histidine kinase	T	CELLULAR PROCESSES AND SIGNALING		2.20	0.252	Up
MSMEG_3119	ABC transporter ATP-binding protein	V	CELLULAR PROCESSES AND SIGNALING	RV1458C	2.02	0.255	Up
murD	UDP-N-acetylmuramoyl-L-alanyl-D-glutamate	M	CELLULAR PROCESSES AND SIGNALING	Rv2155c	1.96	0.273	Up
MSMEG_4225	cell division protein FtsQ	M	CELLULAR PROCESSES AND SIGNALING	Rv2151c	1.71	0.282	Up
MSMEG_0032	cell cycle protein, FtsW/RodA/SpoVE family	D	CELLULAR PROCESSES AND SIGNALING	Rv2154c	1.10	0.286	Up
dacB	D-alanyl-D-alanine carboxypeptidase/D-alany	M	CELLULAR PROCESSES AND SIGNALING	RV3627C	1.52	0.288	Up
MSMEG_4253	glycoside hydrolase family protein	M	CELLULAR PROCESSES AND SIGNALING	Rv0557	2.05	0.291	Up
MSMEG_6087	beta-lactamase	V	CELLULAR PROCESSES AND SIGNALING	Rv3593	1.17	0.293	Up
MSMEG_3622	hypothetical protein	O	CELLULAR PROCESSES AND SIGNALING	Rv1853	1.54	0.296	Up
hypE	hydrogenase expression/formation protein H	O	CELLULAR PROCESSES AND SIGNALING		1.29	0.298	Up
MSMEG_3029	peptidase, A24 (type IV prepilin peptidase) fa	U	CELLULAR PROCESSES AND SIGNALING	RV2551C	1.97	0.300	Up
MSMEG_6900	penicillin-binding protein 1	M	CELLULAR PROCESSES AND SIGNALING	Rv0050	1.48	0.308	Up
MSMEG_1096	urease accessory protein UreD	O	CELLULAR PROCESSES AND SIGNALING		1.46	0.309	Up
MSMEG_4349	hypothetical protein	O	CELLULAR PROCESSES AND SIGNALING	RV2264C	1.05	0.318	Up
MSMEG_2793	sensor-type histidine kinase PrrB	T	CELLULAR PROCESSES AND SIGNALING		1.46	0.327	Up
MSMEG_5695	glutathione S-transferase	O	CELLULAR PROCESSES AND SIGNALING		1.36	0.336	Up
MSMEG_4576	SpfH domain-containing protein	O	CELLULAR PROCESSES AND SIGNALING		1.90	0.337	Up
alr	alanine racemase	M	CELLULAR PROCESSES AND SIGNALING	Rv3423c	1.51	0.351	Up
lspA	lipoprotein signal peptidase	U	CELLULAR PROCESSES AND SIGNALING	Rv1539	1.07	0.356	Up
MSMEG_6133	5-dehydro-4-deoxyglucarate dehydratase	M	CELLULAR PROCESSES AND SIGNALING		1.22	0.366	Up
lexA	LexA repressor	T	CELLULAR PROCESSES AND SIGNALING	Rv2720	2.16	0.379	Up
MSMEG_3761	Clp protease subunit	O	CELLULAR PROCESSES AND SIGNALING		1.03	0.384	Up
ftsZ	cell division protein FtsZ	D	CELLULAR PROCESSES AND SIGNALING	Rv2150c	1.25	0.384	Up
MSMEG_4233	penicillin binding protein transpeptidase dor	M	CELLULAR PROCESSES AND SIGNALING	Rv2163c	1.68	0.409	Up
MSMEG_6171	hypothetical protein	D	CELLULAR PROCESSES AND SIGNALING	RV3660C	1.86	0.415	Up
MSMEG_0627	glycosyl transferase family protein	M	CELLULAR PROCESSES AND SIGNALING		1.74	0.420	Up
glnD	PII uridylyl-transferase	O	CELLULAR PROCESSES AND SIGNALING	Rv2918c	2.10	0.429	Up
MSMEG_5076	ABC transporter	M	CELLULAR PROCESSES AND SIGNALING	RV1217C	1.02	0.439	Up
MSMEG_3145	secreted cell wall-associated hydrolase	M	CELLULAR PROCESSES AND SIGNALING	RV1477	1.40	0.443	Up
MSMEG_2584	penicillin-binding protein	M	CELLULAR PROCESSES AND SIGNALING	RV2864C	1.64	0.452	Up
MSMEG_1624	universal stress protein family protein	T	CELLULAR PROCESSES AND SIGNALING		1.80	0.452	Up
smc	chromosome segregation protein SMC	D	CELLULAR PROCESSES AND SIGNALING	Rv2922c	1.65	0.470	Up
MSMEG_0973	hypothetical protein	O	CELLULAR PROCESSES AND SIGNALING	RV0528	1.37	0.473	Up
MSMEG_4279	adenylate and guanylate cyclase catalytic dor	T	CELLULAR PROCESSES AND SIGNALING	RV2212	1.72	0.481	Up
MSMEG_4256	NLP/P60 family protein	M	CELLULAR PROCESSES AND SIGNALING	RV2190C	1.59	0.481	Up
MSMEG_2739	hypothetical protein	M	CELLULAR PROCESSES AND SIGNALING	RV2721C	1.12	0.482	Up
MSMEG_3860	polyprenol-monophosphomannose synthase	M	CELLULAR PROCESSES AND SIGNALING	Rv2051c	1.55	0.498	Up
MSMEG_6318	diol dehydratase reactivation protein	D	CELLULAR PROCESSES AND SIGNALING		1.56	0.498	Up
murG	UDPdiphospho-muramoylpentapeptide beta	M	CELLULAR PROCESSES AND SIGNALING	Rv2153c	1.66	0.498	Up
MSMEG_5075	ABC transporter ATP-binding protein	V	CELLULAR PROCESSES AND SIGNALING	RV1218C	1.73	0.500	Up
MSMEG_0972	cytochrome C biogenesis protein transmemb	O	CELLULAR PROCESSES AND SIGNALING	Rv0527	1.12	0.511	Up
MSMEG_1536	ftsk/SpoIIIE family protein	D	CELLULAR PROCESSES AND SIGNALING		1.49	0.515	Up

MSMEG_2181	cell filamentation protein	D	CELLULAR PROCESSES AND SIGNALING	Rv3641c	1.25	0.515	Up
murE	UDP-N-acetylmuramoylalanyl-D-glutamate--2	M	CELLULAR PROCESSES AND SIGNALING		1.15	0.536	Up
ftsW	cell division protein FtsW	D	CELLULAR PROCESSES AND SIGNALING	RV2154C	1.21	0.555	Up
MSMEG_3480	streptomycin 6-kinase	V	CELLULAR PROCESSES AND SIGNALING		1.41	0.555	Up
MSMEG_6168	type II secretion system protein F	U	CELLULAR PROCESSES AND SIGNALING	RV3658C	1.38	0.561	Up
MSMEG_0680	UDP-glucose 6-dehydrogenase	M	CELLULAR PROCESSES AND SIGNALING	Rv0322	1.35	0.612	Up
MSMEG_0619	PPE family protein	N	CELLULAR PROCESSES AND SIGNALING	Rv0286	1.04	0.640	Up
MSMEG_1533	subtilase	O	CELLULAR PROCESSES AND SIGNALING	Rv3449	1.60	0.644	Up
mraY	phospho-N-acetylmuramoyl-pentapeptide-tr	M	CELLULAR PROCESSES AND SIGNALING		1.21	0.646	Up
MSMEG_2489	GntR family transcriptional regulator	K	INFORMATION STORAGE AND PROCESSING		2.16	0.007	Up
MSMEG_5451	alkylated DNA repair protein	L	INFORMATION STORAGE AND PROCESSING	RV1000C	1.09	0.012	Up
MSMEG_1648	transcriptional regulator	K	INFORMATION STORAGE AND PROCESSING		1.01	0.016	Up
MSMEG_4339	hypothetical protein	K	INFORMATION STORAGE AND PROCESSING		1.12	0.020	Up
MSMEG_4925	transcriptional regulator, Ada family protein/	L	INFORMATION STORAGE AND PROCESSING		1.52	0.023	Up
MSMEG_0244	two component response transcriptional reg	K	INFORMATION STORAGE AND PROCESSING		1.05	0.027	Up
MSMEG_3630	CopY family transcriptional regulator protein	K	INFORMATION STORAGE AND PROCESSING		1.42	0.038	Up
rplV	50S ribosomal protein L22	J	INFORMATION STORAGE AND PROCESSING	Rv0706	1.05	0.042	Up
MSMEG_0092	transcriptional regulatory protein	K	INFORMATION STORAGE AND PROCESSING	RV0144	1.14	0.045	Up
MSMEG_6125	transcriptional regulatory protein	K	INFORMATION STORAGE AND PROCESSING		1.49	0.045	Up
greA	transcription elongation factor GreA	K	INFORMATION STORAGE AND PROCESSING	Rv1080c	1.31	0.051	Up
MSMEG_4659	GntR family transcriptional regulator	K	INFORMATION STORAGE AND PROCESSING		1.87	0.056	Up
MSMEG_3173	L-asparaginase	J	INFORMATION STORAGE AND PROCESSING	Rv1538c	2.39	0.074	Up
MSMEG_2553	TetR family transcriptional regulator	K	INFORMATION STORAGE AND PROCESSING		1.23	0.077	Up
MSMEG_4449	transcriptional regulator	K	INFORMATION STORAGE AND PROCESSING		1.62	0.078	Up
MSMEG_6191	translation initiation inhibitor	J	INFORMATION STORAGE AND PROCESSING	RV3678C	1.55	0.080	Up
MSMEG_5019	regulatory protein	K	INFORMATION STORAGE AND PROCESSING		2.16	0.086	Up
uvrB	excinuclease ABC subunit B	L	INFORMATION STORAGE AND PROCESSING	Rv1633	1.14	0.086	Up
MSMEG_5706	DNA or RNA helicase of superfamily protein II	L	INFORMATION STORAGE AND PROCESSING	Rv0861c	2.07	0.098	Up
ksgA	dimethyladenosine transferase	J	INFORMATION STORAGE AND PROCESSING		1.21	0.102	Up
MSMEG_5309	hypothetical protein	K	INFORMATION STORAGE AND PROCESSING		1.05	0.111	Up
MSMEG_6077	CarD family transcriptional regulator	K	INFORMATION STORAGE AND PROCESSING	RV3583C	1.28	0.116	Up
MSMEG_2507		K	INFORMATION STORAGE AND PROCESSING		1.19	0.116	Up
MSMEG_6044	ligand-binding /sugar binding domain-contair	K	INFORMATION STORAGE AND PROCESSING	RV3575C	1.38	0.117	Up
MSMEG_2105	ATP dependent DNA ligase	L	INFORMATION STORAGE AND PROCESSING		1.41	0.118	Up
MSMEG_5025	transcriptional regulator	K	INFORMATION STORAGE AND PROCESSING		1.26	0.120	Up
MSMEG_1939	6-O-methylguanine DNA methyltransferase	L	INFORMATION STORAGE AND PROCESSING	RV3204	1.09	0.123	Up
MSMEG_3191	hypothetical protein	J	INFORMATION STORAGE AND PROCESSING	RV1571	2.30	0.127	Up
ruvC	Holliday junction resolvase	L	INFORMATION STORAGE AND PROCESSING	Rv2594c	1.79	0.131	Up
MSMEG_6275	DNA polymerase III subunit epsilon	L	INFORMATION STORAGE AND PROCESSING	Rv3711c	2.39	0.136	Up
MSMEG_4394	LysR family transcriptional regulator	K	INFORMATION STORAGE AND PROCESSING		1.14	0.140	Up
MSMEG_5375	GntR family transcriptional regulator	K	INFORMATION STORAGE AND PROCESSING		1.13	0.141	Up
MSMEG_4174	IclR family transcriptional regulator	K	INFORMATION STORAGE AND PROCESSING		1.25	0.145	Up
MSMEG_4969	two-component system response regulator	K	INFORMATION STORAGE AND PROCESSING		1.06	0.151	Up
mnmA	tRNA-specific 2-thiouridylase MnmA	J	INFORMATION STORAGE AND PROCESSING		1.98	0.153	Up
MSMEG_6083	base excision DNA repair protein, HhH-GPD fa	L	INFORMATION STORAGE AND PROCESSING	Rv3589	1.41	0.156	Up
MSMEG_1139	transcriptional regulator	K	INFORMATION STORAGE AND PROCESSING		1.06	0.159	Up
MSMEG_6864	transcriptional regulator	K	INFORMATION STORAGE AND PROCESSING		1.33	0.159	Up
MSMEG_6931	RNA polymerase sigma-70 factor	K	INFORMATION STORAGE AND PROCESSING	Rv3911	2.17	0.165	Up
MSMEG_5193	AraC family transcriptional regulator	K	INFORMATION STORAGE AND PROCESSING		1.61	0.170	Up
MSMEG_4626	ribonuclease, Rne/Rng family protein	J	INFORMATION STORAGE AND PROCESSING	Rv2444c	1.37	0.171	Up
MSMEG_3822	GntR family transcriptional regulator	K	INFORMATION STORAGE AND PROCESSING		1.35	0.175	Up
MSMEG_3677	serine/threonine protein kinase	L	INFORMATION STORAGE AND PROCESSING	Rv1746	1.53	0.177	Up
MSMEG_6174	IclR family transcriptional regulator	K	INFORMATION STORAGE AND PROCESSING		1.91	0.177	Up
MSMEG_3644	MerR family transcriptional regulator	K	INFORMATION STORAGE AND PROCESSING	RV1830	1.39	0.178	Up
MSMEG_5673	transcriptional regulator	K	INFORMATION STORAGE AND PROCESSING		2.13	0.180	Up
MSMEG_5696	cold-shock DNA-binding domain-containing p	K	INFORMATION STORAGE AND PROCESSING	Rv0871	1.40	0.186	Up
mtrA	DNA-binding response regulator MtrA	K	INFORMATION STORAGE AND PROCESSING	Rv3246c	1.30	0.194	Up
xerC	site-specific tyrosine recombinase XerC	L	INFORMATION STORAGE AND PROCESSING	Rv2894c	1.90	0.195	Up
MSMEG_2106	transcriptional regulator	K	INFORMATION STORAGE AND PROCESSING		1.99	0.195	Up
MSMEG_5282	transcriptional regulator	K	INFORMATION STORAGE AND PROCESSING		1.03	0.197	Up
MSMEG_4898	hypothetical protein	K	INFORMATION STORAGE AND PROCESSING	RV1343C	1.52	0.201	Up
MSMEG_0028	serine-threonine protein kinase	L	INFORMATION STORAGE AND PROCESSING	Rv0014c	1.31	0.205	Up
ruvA	Holliday junction DNA helicase RuvA	L	INFORMATION STORAGE AND PROCESSING	Rv2593c	1.43	0.206	Up
MSMEG_0207	MarR family transcriptional regulator	K	INFORMATION STORAGE AND PROCESSING		1.17	0.206	Up
pth	peptidyl-tRNA hydrolase	J	INFORMATION STORAGE AND PROCESSING	Rv1014c	1.05	0.207	Up
gatA	glutamyl-tRNA(Gln)/aspartyl-tRNA(Asn) amid	J	INFORMATION STORAGE AND PROCESSING	Rv3011c	1.59	0.225	Up
dnaE	DNA polymerase III subunit alpha	L	INFORMATION STORAGE AND PROCESSING		1.51	0.229	Up
MSMEG_2120	hypothetical protein	K	INFORMATION STORAGE AND PROCESSING		2.48	0.229	Up
MSMEG_5838	TetR family transcriptional regulator	K	INFORMATION STORAGE AND PROCESSING		1.49	0.231	Up
MSMEG_3240	LuxR family transcriptional regulator	K	INFORMATION STORAGE AND PROCESSING		1.08	0.233	Up
nth	endonuclease III	L	INFORMATION STORAGE AND PROCESSING	Rv3674c	1.26	0.242	Up
alaS	alanyl-tRNA synthetase	J	INFORMATION STORAGE AND PROCESSING	Rv2555c	1.38	0.243	Up
selB	selenocysteine-specific translation elongatio	J	INFORMATION STORAGE AND PROCESSING		2.79	0.245	Up
MSMEG_6096	pantothenate kinase	K	INFORMATION STORAGE AND PROCESSING		2.45	0.248	Up
MSMEG_6128	two-component system response regulator	K	INFORMATION STORAGE AND PROCESSING		1.66	0.250	Up
MSMEG_2182	transcriptional regulator	K	INFORMATION STORAGE AND PROCESSING	RV0258C	1.43	0.256	Up
lysS	lysyl-tRNA synthetase	J	INFORMATION STORAGE AND PROCESSING		1.26	0.270	Up
rph	ribonuclease PH	J	INFORMATION STORAGE AND PROCESSING		1.34	0.276	Up
hrpA	ATP-dependent helicase HrpA	L	INFORMATION STORAGE AND PROCESSING		2.16	0.283	Up
MSMEG_6926	tRNA adenylyltransferase	J	INFORMATION STORAGE AND PROCESSING		2.01	0.293	Up
MSMEG_5987	two-component regulator	K	INFORMATION STORAGE AND PROCESSING		1.49	0.293	Up
MSMEG_4912	helicase	L	INFORMATION STORAGE AND PROCESSING	Rv1329c	1.68	0.299	Up
MSMEG_2453	GntR family transcriptional regulator	K	INFORMATION STORAGE AND PROCESSING		1.21	0.311	Up
MSMEG_4964	TetR family transcriptional regulator	K	INFORMATION STORAGE AND PROCESSING		1.13	0.311	Up
MSMEG_6682	RNA polymerase sigma-70 factor	K	INFORMATION STORAGE AND PROCESSING		1.09	0.311	Up
rimM	16S rRNA-processing protein RimM	J	INFORMATION STORAGE AND PROCESSING	Rv2907c	1.24	0.334	Up
MSMEG_0592	rhamnose catabolism operon transcriptional	K	INFORMATION STORAGE AND PROCESSING		1.66	0.337	Up
MSMEG_3505	6-aminohexanoate-cyclic-dimer hydrolase	J	INFORMATION STORAGE AND PROCESSING		2.19	0.343	Up
MSMEG_0105	LuxR family transcriptional regulator	K	INFORMATION STORAGE AND PROCESSING	Rv0844c	1.37	0.350	Up
MSMEG_1681	endoribonuclease L-PSP superfamily protein	J	INFORMATION STORAGE AND PROCESSING		1.45	0.350	Up
MSMEG_3885	DEAD/DEAH box helicase	L	INFORMATION STORAGE AND PROCESSING	Rv2092c	2.13	0.355	Up
MSMEG_6327	cytidine and deoxycytidylate deaminase	J	INFORMATION STORAGE AND PROCESSING	RV3752C	1.36	0.363	Up
MSMEG_1902	transcriptional regulator	K	INFORMATION STORAGE AND PROCESSING		1.59	0.366	Up
recO	DNA repair protein RecO	L	INFORMATION STORAGE AND PROCESSING	Rv2362c	1.21	0.379	Up
MSMEG_4486	ArsR family transcriptional regulator	K	INFORMATION STORAGE AND PROCESSING	RV2358	1.57	0.400	Up
MSMEG_4572	hypothetical protein	L	INFORMATION STORAGE AND PROCESSING	RV2413C	1.44	0.404	Up
atzF	allophanate hydrolase	J	INFORMATION STORAGE AND PROCESSING		1.36	0.407	Up
MSMEG_3026	Holliday junction resolvase-like protein	L	INFORMATION STORAGE AND PROCESSING	RV2554C	1.10	0.408	Up
xseB	exodeoxyribonuclease VII small subunit	L	INFORMATION STORAGE AND PROCESSING	Rv1107c	1.17	0.410	Up
MSMEG_6903	PadR family transcriptional regulator	K	INFORMATION STORAGE AND PROCESSING	RV0047C	1.14	0.419	Up
ung	uracil-DNA glycosylase	L	INFORMATION STORAGE AND PROCESSING	Rv2976c	1.45	0.421	Up
MSMEG_1646	ribosomal RNA adenine dimethylase	J	INFORMATION STORAGE AND PROCESSING		1.98	0.431	Up
MSMEG_3790	TrmH family RNA methyltransferase	J	INFORMATION STORAGE AND PROCESSING	Rv1644	1.13	0.438	Up
MSMEG_0691	transcriptional regulatory protein	K	INFORMATION STORAGE AND PROCESSING	RV0339C	1.44	0.439	Up
MSMEG_4304	regulatory protein	K	INFORMATION STORAGE AND PROCESSING		1.64	0.461	Up
MSMEG_2472	AsnC family transcriptional regulator	K	INFORMATION STORAGE AND PROCESSING		1.06	0.462	Up
MSMEG_1692	ECF-family protein RNA polymerase sigma fact	K	INFORMATION STORAGE AND PROCESSING		1.51	0.465	Up
MSMEG_6673	6-aminohexanoate-cyclic-dimer hydrolase	J	INFORMATION STORAGE AND PROCESSING		1.08	0.479	Up

MSMEG_2576	deoxyribodipyrimidine photo-lyase	L	INFORMATION STORAGE AND PROCESSING		1.07	0.488	Up
MSMEG_6421	transcriptional regulator	K	INFORMATION STORAGE AND PROCESSING	RV3840	1.47	0.489	Up
MSMEG_5513	serine/threonine protein kinase	L	INFORMATION STORAGE AND PROCESSING		1.35	0.493	Up
MSMEG_1901	DNA-binding HTH domain-containing protein	K	INFORMATION STORAGE AND PROCESSING		1.32	0.502	Up
MSMEG_4205	transcriptional regulator	K	INFORMATION STORAGE AND PROCESSING		1.11	0.503	Up
MSMEG_5074	transcriptional regulatory protein	K	INFORMATION STORAGE AND PROCESSING	RV1219C	2.02	0.505	Up
dnaG	DNA primase	L	INFORMATION STORAGE AND PROCESSING	Rv2343c	1.15	0.505	Up
MSMEG_1700	TetR family transcriptional regulator	K	INFORMATION STORAGE AND PROCESSING		1.41	0.507	Up
MSMEG_5004	DNA repair exonuclease	L	INFORMATION STORAGE AND PROCESSING	RV1277	1.24	0.508	Up
MSMEG_4517	TetR family transcriptional regulator	K	INFORMATION STORAGE AND PROCESSING		1.45	0.511	Up
rho	transcription termination factor Rho	K	INFORMATION STORAGE AND PROCESSING	Rv1297	1.45	0.519	Up
MSMEG_3193	TetR family transcriptional regulator	K	INFORMATION STORAGE AND PROCESSING		1.55	0.520	Up
MSMEG_0367	O-demethylpuromycin-O-methyltransferase	J	INFORMATION STORAGE AND PROCESSING		1.51	0.526	Up
MSMEG_4022	TetR family transcriptional regulator	K	INFORMATION STORAGE AND PROCESSING		1.16	0.534	Up
MSMEG_3856	CadC family transcriptional regulator	K	INFORMATION STORAGE AND PROCESSING		1.04	0.550	Up
MSMEG_4366	serine/threonine protein kinase	L	INFORMATION STORAGE AND PROCESSING		1.10	0.553	Up
MSMEG_3172	DNA polymerase IV	L	INFORMATION STORAGE AND PROCESSING		1.50	0.556	Up
trmD	tRNA (guanine-N(1)-)-methyltransferase	J	INFORMATION STORAGE AND PROCESSING	Rv2906c	1.01	0.570	Up
MSMEG_1941	helicase, UvrD/Rep family protein	L	INFORMATION STORAGE AND PROCESSING	RV3202C	1.65	0.604	Up
MSMEG_1943	ATP-dependent DNA helicase	L	INFORMATION STORAGE AND PROCESSING	RV3201C	1.09	0.612	Up
priA	primosome assembly protein PriA	L	INFORMATION STORAGE AND PROCESSING	Rv1402	1.65	0.666	Up
MSMEG_1540	ATP-dependent RNA helicase	J	INFORMATION STORAGE AND PROCESSING		1.24	0.709	Up
eutC	ethanolamine ammonia-lyase small subunit	E	METABOLISM		2.28	0.006	Up
MSMEG_4908	endo-type 6-aminohexanoate oligomer hydro	Q	METABOLISM	RV1333	1.70	0.009	Up
MSMEG_2422	acylphosphatase	C	METABOLISM	Rv2922A	1.61	0.010	Up
MSMEG_3108	ABC transporter ATPase	G	METABOLISM		1.24	0.010	Up
MSMEG_3266	maltose/maltodextrin-binding protein	G	METABOLISM		1.10	0.012	Up
phhB	pterin-4-alpha-carbinolamine dehydratase	H	METABOLISM		2.19	0.016	Up
MSMEG_2597	aldehyde dehydrogenase	C	METABOLISM	Rv2858c	1.90	0.022	Up
etfA	electron transfer flavoprotein subunit alpha	C	METABOLISM		1.26	0.023	Up
add	adenosine deaminase	F	METABOLISM	Rv3313c	1.31	0.024	Up
trpB	tryptophan synthase subunit beta	E	METABOLISM	Rv1612	1.51	0.027	Up
MSMEG_3187	acyltransferase domain-containing protein	I	METABOLISM	RV1565C	1.11	0.029	Up
MSMEG_6195	ion-transporting ATPase	P	METABOLISM	RV3680	1.21	0.029	Up
MSMEG_5163	glycine/betaine ABC transporter periplasmic	E	METABOLISM		1.63	0.035	Up
MSMEG_6041	acyl-CoA dehydrogenase	I	METABOLISM	Rv3573c	1.33	0.036	Up
pgm	phosphoglucomutase	G	METABOLISM	Rv3068c	1.04	0.040	Up
MSMEG_2342	glycosyltransferase	C	METABOLISM		1.39	0.041	Up
MSMEG_2469	AMP-dependent synthetase/ligase	Q	METABOLISM		2.92	0.041	Up
MSMEG_3848	para-nitrobenzyl esterase	I	METABOLISM	Rv2045c	1.45	0.042	Up
MSMEG_2488	[NADP+] succinate-semialdehyde dehydroge	C	METABOLISM		2.40	0.042	Up
MSMEG_5988	hypothetical protein	I	METABOLISM		1.30	0.044	Up
MSMEG_4662	deoxyribose-phosphate aldolase superfamily	G	METABOLISM		1.49	0.047	Up
MSMEG_5404	propionate--CoA ligase	I	METABOLISM		1.26	0.048	Up
cbiQ	cobalt ABC transporter permease	P	METABOLISM		1.71	0.048	Up
MSMEG_6483	methyltransferase type 11	H	METABOLISM		1.36	0.051	Up
MSMEG_2632	SAM-dependent methyltransferase	H	METABOLISM		2.02	0.053	Up
MSMEG_2648	Sfp-type phosphopantetheinyl transferase	Q	METABOLISM	RV2794C	1.24	0.054	Up
ggt	gamma-glutamyltranspeptidase	E	METABOLISM	Rv0773c	1.63	0.055	Up
MSMEG_2878	acyl-CoA dehydrogenase	I	METABOLISM		1.38	0.055	Up
leuB	3-isopropylmalate dehydrogenase	E	METABOLISM	Rv2995c	1.49	0.055	Up
MSMEG_4519	NrtC protein	I	METABOLISM		2.13	0.057	Up
dut	deoxyuridine 5'-triphosphate nucleotidohyd	F	METABOLISM	Rv2697c	1.89	0.057	Up
MSMEG_4579	phosphoglycerate mutase	G	METABOLISM	RV2419C	2.30	0.059	Up
MSMEG_6250	glutamate--cysteine ligase	H	METABOLISM	Rv3704c	1.54	0.062	Up
MSMEG_0741	hypothetical protein	C	METABOLISM		1.06	0.063	Up
MSMEG_3125	cysteine desulfurase	E	METABOLISM	Rv1464	1.21	0.063	Up
MSMEG_4474	acyl-CoA oxidase	I	METABOLISM		1.60	0.063	Up
MSMEG_0200	hypothetical protein	C	METABOLISM		1.09	0.064	Up
MSMEG_2760	polyphosphate glucokinase	G	METABOLISM	Rv2702	1.03	0.065	Up
MSMEG_0762	cytochrome P450	Q	METABOLISM		1.00	0.066	Up
MSMEG_5970	glutamate-1-semialdehyde 2,1-aminomutase	H	METABOLISM		1.51	0.067	Up
MSMEG_2941	NAD dependent epimerase/dehydratase	G	METABOLISM		1.06	0.075	Up
MSMEG_2503	hypothetical protein	P	METABOLISM		1.04	0.077	Up
MSMEG_5863	cytochrome P450	Q	METABOLISM	Rv0764c	1.22	0.078	Up
MSMEG_5906	acyl-CoA dehydrogenase	I	METABOLISM	Rv3504	1.05	0.078	Up
sodC	copper/zinc superoxide dismutase	P	METABOLISM	Rv0432	1.17	0.079	Up
MSMEG_0662	putrescine transport ATP-binding protein Pot	E	METABOLISM		1.39	0.082	Up
MSMEG_6591	class V aminotransferase	E	METABOLISM		1.05	0.084	Up
MSMEG_1245	phosphoadenosine phosphosulfate reductase	H	METABOLISM		1.04	0.088	Up
MSMEG_0667	butyryl-CoA dehydrogenase	I	METABOLISM		1.71	0.091	Up
MSMEG_6914	hypothetical protein	G	METABOLISM		1.69	0.091	Up
MSMEG_2726	glutamate permease	E	METABOLISM		1.18	0.093	Up
MSMEG_5699	ThiS family protein	H	METABOLISM	Rv0868c	2.19	0.094	Up
MSMEG_1062	O-succinylbenzoic acid--CoA ligase	Q	METABOLISM	Rv0542c	1.08	0.094	Up
MSMEG_5619	acyl-CoA dehydrogenase	I	METABOLISM		1.41	0.099	Up
MSMEG_6533	hypothetical protein	I	METABOLISM		1.41	0.100	Up
otsB	trehalose-phosphatase	G	METABOLISM		1.53	0.103	Up
MSMEG_4328	3-oxoacyl-ACP synthase	Q	METABOLISM	Rv2246	1.25	0.106	Up
MSMEG_2249	hypothetical protein	C	METABOLISM		1.11	0.110	Up
MSMEG_1049	UbiE/COQ5 family methyltransferase	H	METABOLISM	RV3038C	1.27	0.117	Up
manB	phosphomannomutase	G	METABOLISM		1.02	0.117	Up
MSMEG_4717	carboxyl transferase domain-containing prote	I	METABOLISM	Rv2502c	1.30	0.121	Up
MSMEG_0127	oxidoreductase, zinc-binding dehydrogenase	C	METABOLISM	Rv0162c	1.09	0.123	Up
MSMEG_4977	3'(2'),5'-bisphosphate nucleotidase	P	METABOLISM		1.15	0.124	Up
MSMEG_5485	molybdopterin biosynthesis protein	H	METABOLISM	Rv0984	1.69	0.124	Up
MSMEG_3786	D-amino acid deaminase	E	METABOLISM		1.93	0.127	Up
coaD	phosphopantetheine adenylyltransferase	H	METABOLISM		1.29	0.128	Up
MSMEG_4653	AP endonuclease, family protein 2 superfami	G	METABOLISM		1.48	0.131	Up
ispG	4-hydroxy-3-methylbut-2-en-1-yl diphosphat	I	METABOLISM		1.13	0.133	Up
MSMEG_6392	polyketide synthase	Q	METABOLISM	Rv3800c	2.06	0.138	Up
corA	magnesium and cobalt transporter CorA	P	METABOLISM	Rv1239c	1.78	0.144	Up
MSMEG_1945	ion channel membrane protein	P	METABOLISM	RV3200C	1.92	0.145	Up
MSMEG_5853	cytochrome P450	Q	METABOLISM		1.36	0.147	Up
MSMEG_3463	esterase	Q	METABOLISM	Rv3298c	1.31	0.148	Up
MSMEG_4334	flavoprotein	C	METABOLISM	RV2250.1	1.38	0.149	Up
MSMEG_3618	hypothetical protein	I	METABOLISM	Rv1860	1.67	0.150	Up
MSMEG_2699	hypothetical protein	C	METABOLISM	RV2739C	1.21	0.150	Up
trpD	anthranilate phosphoribosyltransferase	E	METABOLISM	Rv2192c	2.04	0.150	Up
MSMEG_0435	allophanate hydrolase subunit 2	E	METABOLISM	RV0263C	1.74	0.154	Up
MSMEG_5639	enoyl-CoA hydratase	I	METABOLISM	Rv0905	1.09	0.157	Up
MSMEG_6802	ABC transporter ATP-binding protein	G	METABOLISM		1.05	0.157	Up
cobM	precorrin-4 C(11)-methyltransferase	H	METABOLISM	Rv2071c	1.60	0.161	Up
carA	carbamoyl phosphate synthase small subunit	F	METABOLISM	Rv1383	1.32	0.165	Up
MSMEG_2698	hypothetical protein	Q	METABOLISM	RV2740	3.23	0.166	Up
MSMEG_1219	ABC transporter permease	E	METABOLISM		1.05	0.166	Up
MSMEG_1292	FAD binding domain-containing protein	C	METABOLISM		1.97	0.166	Up
MSMEG_1497	acyl-CoA dehydrogenase	I	METABOLISM	Rv0752c	1.12	0.167	Up

nuoD	NADH dehydrogenase subunit D	C	METABOLISM	Rv3148	1.67	0.168	Up
MSMEG_3471	hypothetical protein	H	METABOLISM		1.26	0.168	Up
MSMEG_3634	inosine 5-monophosphate dehydrogenase	F	METABOLISM	RV1843C	1.35	0.170	Up
MSMEG_1762	piperideine-6-carboxylic acid dehydrogenase	C	METABOLISM	Rv3293	1.71	0.171	Up
MSMEG_5857	oxidoreductase	I	METABOLISM	RV0770	1.20	0.171	Up
MSMEG_0358	ribonucleotide-diphosphate reductase subur	F	METABOLISM	RV0233	1.33	0.177	Up
MSMEG_2490	decarboxylase	Q	METABOLISM		1.57	0.178	Up
MSMEG_5470	molybdopterin biosynthesis protein MoeA 1	H	METABOLISM	Rv0994	1.82	0.178	Up
MSMEG_6866	peptide ABC transporter permease	P	METABOLISM		1.48	0.179	Up
MSMEG_6416	phosphoglycerate mutase	G	METABOLISM	RV3837C	1.38	0.180	Up
moaC	molybdenum cofactor biosynthesis protein M	H	METABOLISM	Rv0864	1.26	0.180	Up
thiE	thiamine-phosphate pyrophosphorylase	H	METABOLISM	Rv0414c	1.74	0.182	Up
MSMEG_4644	molybdopterin-guanine dinucleotide biosynt	H	METABOLISM	Rv2453c	1.24	0.182	Up
MSMEG_6408	acyltransferase	I	METABOLISM	RV3814C	1.67	0.182	Up
MSMEG_6173	morphological differentiation-associated pro	E	METABOLISM	RV3661	1.53	0.182	Up
MSMEG_2680	amino acid transporter	E	METABOLISM		1.13	0.189	Up
MSMEG_1905	acyl-CoA dehydrogenase	I	METABOLISM		1.17	0.195	Up
MSMEG_0889	succinate-semialdehyde dehydrogenase	C	METABOLISM		1.70	0.196	Up
MSMEG_6473	tetracenomycin polyketide synthesis O-meth	Q	METABOLISM		1.37	0.196	Up
MSMEG_6175	2-keto-3-deoxy-galactonokinase	G	METABOLISM		1.17	0.197	Up
MSMEG_3269	sugar ABC transporter ATP-binding protein	G	METABOLISM		1.05	0.197	Up
MSMEG_5512	magnesium chelatase	H	METABOLISM	RV0958	1.07	0.198	Up
MSMEG_6290	DNA-binding protein	Q	METABOLISM		1.30	0.202	Up
MSMEG_2762	inositol-1-monophosphatase	G	METABOLISM	Rv2701c	1.48	0.206	Up
MSMEG_1340	(3R)-hydroxyacyl-ACP dehydratase subunit Ha	I	METABOLISM	RV0635	1.11	0.207	Up
MSMEG_2405	MarR family transcriptional regulator	E	METABOLISM		1.94	0.207	Up
thrC	threonine synthase	E	METABOLISM	Rv1295	2.07	0.208	Up
dxs	1-deoxy-D-xylulose-5-phosphate synthase	I	METABOLISM		1.23	0.208	Up
ispF	2-C-methyl-D-erythritol 2,4-cyclodiphosphat	I	METABOLISM	Rv3581c	1.18	0.210	Up
MSMEG_2070	acyl-CoA dehydrogenase	I	METABOLISM		1.01	0.212	Up
MSMEG_5442	glutamate dehydrogenase	E	METABOLISM		1.53	0.214	Up
MSMEG_6203	cysteine synthase/cystathionine beta-syntha	E	METABOLISM	RV3684	1.14	0.215	Up
MSMEG_0985	sugar transporter family protein	G	METABOLISM	Rv3476c	2.39	0.216	Up
MSMEG_0971	hypothetical protein	C	METABOLISM	RV0526	1.60	0.217	Up
MSMEG_2511	siderophore utilization protein	P	METABOLISM	RV2895C	1.20	0.218	Up
MSMEG_5064	malyl-CoA lyase	G	METABOLISM		1.67	0.223	Up
MSMEG_1366	ABC transporter ATP-binding protein	Q	METABOLISM	Rv0655	1.41	0.229	Up
MSMEG_1665	gamma-aminobutyraldehyde dehydrogenase	C	METABOLISM		1.46	0.231	Up
pstA	phosphate ABC transporter permease	P	METABOLISM	Rv0930	1.05	0.232	Up
xylB	xylulokinase	G	METABOLISM		2.06	0.232	Up
MSMEG_6585	acyl-CoA dehydrogenase	I	METABOLISM		2.76	0.235	Up
MSMEG_6179	acetyl-coenzyme A synthetase	I	METABOLISM		1.06	0.235	Up
MSMEG_5059	ABC transporter permease	G	METABOLISM	Rv1237	1.79	0.235	Up
pyrC	dihydroorotase	F	METABOLISM		1.09	0.236	Up
MSMEG_2875	esterase	I	METABOLISM		1.48	0.243	Up
MSMEG_5883	2Fe-2S-binding domain-containing protein	C	METABOLISM		1.66	0.244	Up
MSMEG_0968	cytochrome P450	Q	METABOLISM		2.08	0.246	Up
cobA	uroporphyrin-III C-methyltransferase	H	METABOLISM	Rv2847c	1.24	0.248	Up
ilvE	branched-chain amino acid aminotransferase	H	METABOLISM	Rv2210c	1.43	0.253	Up
MSMEG_3116	inositol-1-monophosphatase	G	METABOLISM		2.11	0.254	Up
MSMEG_5538	[NADP+] succinate-semialdehyde dehydroge	C	METABOLISM		1.12	0.254	Up
folE	GTP cyclohydrolase I	H	METABOLISM	Rv3609c	1.75	0.255	Up
glpK	glycerol kinase	C	METABOLISM	Rv3696c	1.18	0.255	Up
MSMEG_0422	transferase	G	METABOLISM	RV1998C	1.00	0.259	Up
cobD	cobalamin biosynthesis protein	H	METABOLISM	Rv2236c	1.70	0.259	Up
rocD	ornithine--oxo-acid transaminase	E	METABOLISM	Rv2321c	2.13	0.261	Up
MSMEG_2111	chorismate mutase	E	METABOLISM	RV1885C	1.26	0.262	Up
MSMEG_3532	serine/threonine dehydratase	E	METABOLISM		1.21	0.263	Up
MSMEG_3568	hypothetical protein	P	METABOLISM		1.20	0.264	Up
MSMEG_5678	glyoxalase/bleomycin resistance protein/dio	E	METABOLISM		1.32	0.264	Up
MSMEG_1046	molybdenum ABC transporter ATPase	P	METABOLISM	RV3041C	1.28	0.264	Up
MSMEG_6294	caib/baif family protein	C	METABOLISM		1.31	0.264	Up
MSMEG_6688	regulatory protein	Q	METABOLISM		1.24	0.265	Up
MSMEG_6020	sugar ABC transporter periplasmic protein	G	METABOLISM		1.10	0.267	Up
MSMEG_3465	fatty-acid--CoA ligase	Q	METABOLISM	Rv1925	1.31	0.269	Up
MSMEG_3608	acetyl-CoA acetyltransferase	I	METABOLISM	RV1867	1.47	0.272	Up
MSMEG_6430	hypothetical protein	C	METABOLISM		1.18	0.273	Up
MSMEG_0087	glucitol operon repressor	G	METABOLISM		1.76	0.275	Up
MSMEG_2117	beta-glucoside-specific EII permease	G	METABOLISM		1.17	0.279	Up
MSMEG_5884	3-hydroxyisobutyrate dehydrogenase	I	METABOLISM		1.50	0.279	Up
MSMEG_4719	peptidase S9, prolyl oligopeptidase	E	METABOLISM		1.68	0.280	Up
MSMEG_4906	ThiS family protein	H	METABOLISM		1.36	0.281	Up
MSMEG_5153	hypothetical protein	I	METABOLISM	RV1157C	1.56	0.282	Up
MSMEG_1475	hypothetical protein	C	METABOLISM		1.32	0.285	Up
MSMEG_0098	methyltransferase	H	METABOLISM	RV0839	1.18	0.288	Up
lysA	diaminopimelate decarboxylase	E	METABOLISM	Rv1293	1.30	0.291	Up
MSMEG_4641	salicylate hydroxylase	C	METABOLISM		1.19	0.292	Up
MSMEG_0634	PAP2 superfamily protein	I	METABOLISM	RV0308	1.66	0.301	Up
MSMEG_4301	fatty-acid--CoA ligase	Q	METABOLISM		1.14	0.301	Up
MSMEG_1433	thioesterase	Q	METABOLISM		1.06	0.301	Up
MSMEG_0436	allophanate hydrolase subunit 1	E	METABOLISM	RV0264C	1.36	0.305	Up
MSMEG_0970	phosphoglycerate mutase	G	METABOLISM	RV0525	2.28	0.317	Up
MSMEG_3668	acyl-CoA dehydrogenase	I	METABOLISM		2.15	0.317	Up
MSMEG_3852	aliphatic sulfonate binding protein	P	METABOLISM		2.30	0.317	Up
malQ	4-alpha-glucanotransferase	G	METABOLISM	Rv1781c	1.73	0.318	Up
MSMEG_3598	periplasmic sugar-binding proteins	G	METABOLISM		1.34	0.318	Up
MSMEG_5339	nitrile hydratase regulator1	E	METABOLISM		1.16	0.318	Up
MSMEG_1573	carbohydrate kinase	G	METABOLISM	RV3433C	1.76	0.318	Up
MSMEG_2769	TrkB protein	P	METABOLISM		1.14	0.318	Up
MSMEG_1432	acetylcholinesterase	I	METABOLISM		1.68	0.319	Up
MSMEG_2017	MerR family transcriptional regulator	H	METABOLISM		1.50	0.319	Up
serB	phosphoserine phosphatase	E	METABOLISM	Rv3042c	1.56	0.324	Up
MSMEG_0316	NagC regulator	G	METABOLISM		1.47	0.324	Up
MSMEG_0991	NAD-dependent epimerase/dehydratase	G	METABOLISM	Rv0536	1.79	0.324	Up
MSMEG_6409	acyltransferase	I	METABOLISM	RV3816C	1.11	0.325	Up
thiS	sulfur carrier protein ThiS	H	METABOLISM	Rv0416	1.11	0.329	Up
MSMEG_5589	manganese transporter MntH	P	METABOLISM	Rv0924c	1.75	0.330	Up
MSMEG_5220	esterase/lipase/thioesterase	I	METABOLISM		1.50	0.337	Up
MSMEG_6320	diol dehydrase subunit gamma	Q	METABOLISM		1.66	0.340	Up
MSMEG_3059	esterase	I	METABOLISM	Rv1400c	1.57	0.340	Up
MSMEG_2353	hypothetical protein	G	METABOLISM		1.87	0.341	Up
MSMEG_5199	acyl-CoA dehydrogenase	I	METABOLISM		1.01	0.341	Up
MSMEG_0319	acyltransferase	I	METABOLISM	RV0228	1.19	0.343	Up
MSMEG_4524	salicylate synthase MbtI	H	METABOLISM	Rv2386c	2.02	0.346	Up
MSMEG_1546	coenzyme B12-dependent glycerol dehydrog	Q	METABOLISM		1.42	0.347	Up
gabD2	succinate-semialdehyde dehydrogenase	C	METABOLISM	Rv1731	2.06	0.348	Up
MSMEG_4873	enoyl-CoA hydratase/isomerase	I	METABOLISM		1.77	0.348	Up
cysM	cysteine synthase B	E	METABOLISM	Rv1336	2.25	0.349	Up

MSMEG_6072	citrate transporter	P	METABOLISM	Rv3578	1.78	0.352	Up
MSMEG_0862	molybdopterin biosynthesis protein MoeA	H	METABOLISM	Rv0866	1.05	0.357	Up
aroE	shikimate 5-dehydrogenase	E	METABOLISM	Rv2552c	1.39	0.362	Up
panC	pantoate--beta-alanine ligase	H	METABOLISM	Rv3602c	1.34	0.363	Up
argC	N-acetyl-gamma-glutamyl-phosphate reducta	E	METABOLISM	Rv1652	1.44	0.365	Up
MSMEG_6582	pyridine nucleotide-disulfide oxidoreductase	C	METABOLISM		2.04	0.366	Up
ureB	urease subunit beta	E	METABOLISM	Rv1849	1.24	0.368	Up
MSMEG_4962	hypothetical protein	C	METABOLISM	RV1751	1.45	0.370	Up
MSMEG_0871	aldehyde or xanthine dehydrogenase, molyb	C	METABOLISM		1.23	0.371	Up
MSMEG_2501	nitrate/sulfonate/bicarbonate ABC transport	P	METABOLISM		1.03	0.371	Up
MSMEG_0361	glycosyl hydrolase	G	METABOLISM	Rv0237	1.29	0.371	Up
MSMEG_5533	4Fe-4S ferredoxin	C	METABOLISM		1.71	0.372	Up
psd	phosphatidylserine decarboxylase	I	METABOLISM	Rv0437c	1.68	0.372	Up
MSMEG_3203	transporter LysE family protein	E	METABOLISM		1.88	0.373	Up
aroC	chorismate synthase	E	METABOLISM		1.11	0.374	Up
hisF	imidazole glycerol phosphate synthase subun	E	METABOLISM	Rv1605	1.42	0.375	Up
MSMEG_6520	orotate phosphoribosyltransferase	F	METABOLISM	Rv0382c	1.33	0.376	Up
MSMEG_2122	dihydroxyacetone kinase subunit DhaL	G	METABOLISM		1.69	0.380	Up
MSMEG_2357	cysteine desulfurase	E	METABOLISM	Rv3025c	1.69	0.386	Up
MSMEG_0935	2,3-bisphosphoglycerate-independent phosp	G	METABOLISM		1.21	0.386	Up
MSMEG_5491	acyl-CoA dehydrogenase	I	METABOLISM	Rv0975c	1.21	0.390	Up
thiO	glycine oxidase ThiO	E	METABOLISM	Rv0415	1.51	0.395	Up
guaA	GMP synthase	F	METABOLISM		1.09	0.396	Up
MSMEG_5251	hypothetical protein	P	METABOLISM		1.53	0.397	Up
MSMEG_5994	acyl-CoA dehydrogenase	I	METABOLISM	Rv3544c	1.47	0.398	Up
MSMEG_5640	amylo-alpha-1,6-glucosidase	G	METABOLISM		1.59	0.401	Up
MSMEG_1374	ribose ABC transporter periplasmic binding p	G	METABOLISM		1.74	0.404	Up
MSMEG_6584	acyl-CoA dehydrogenase	I	METABOLISM		1.33	0.404	Up
MSMEG_3057	metal ABC transporter permease	P	METABOLISM		1.23	0.406	Up
MSMEG_6231	hypothetical protein	H	METABOLISM	RV1978	1.53	0.408	Up
MSMEG_4305	bifunctional RNase H/acid phosphatase	G	METABOLISM	RV2228C	1.94	0.411	Up
MSMEG_4984	hypothetical protein	Q	METABOLISM	RV1532C	1.39	0.412	Up
MSMEG_0357	transmembrane transporter	G	METABOLISM		1.03	0.413	Up
MSMEG_2359	methionine synthase, vitamin-B12 independ	E	METABOLISM	RV3015C	1.17	0.414	Up
uppS	UDP pyrophosphate synthase	I	METABOLISM	RV1086	1.40	0.421	Up
MSMEG_1940	alpha/beta hydrolase	I	METABOLISM	Rv3203	1.71	0.423	Up
MSMEG_1572	GntR family transcriptional regulator	E	METABOLISM		1.58	0.426	Up
MSMEG_0232	sugar transporter family protein	G	METABOLISM	RV0191	1.42	0.430	Up
MSMEG_2493	aminotransferase, class I and II family protein	E	METABOLISM		1.73	0.431	Up
MSMEG_4299	enoyl-CoA hydratase/isomerase	I	METABOLISM		1.89	0.431	Up
MSMEG_5693	transporter major facilitator family protein	G	METABOLISM	RV0876C	1.80	0.434	Up
MSMEG_3506	amino acid decarboxylase	E	METABOLISM		1.42	0.436	Up
MSMEG_2426	nitrogen regulatory protein P-II	E	METABOLISM	Rv2919c	1.85	0.442	Up
MSMEG_5795	4-amino-4-deoxychorismate lyase	H	METABOLISM	RV0812	1.05	0.444	Up
MSMEG_1378	1-phosphofructokinase	G	METABOLISM		2.34	0.445	Up
MSMEG_5413	exopolyphosphatase	P	METABOLISM	RV1026	1.52	0.446	Up
MSMEG_6437	copper resistance protein D	P	METABOLISM		1.79	0.450	Up
MSMEG_3524	linalool 8-monooxygenase	Q	METABOLISM		1.05	0.451	Up
cobH	precorrin-8X methylmutase	H	METABOLISM		1.47	0.454	Up
MSMEG_6519	pyridoxamine 5'-phosphate oxidase	P	METABOLISM		1.47	0.454	Up
moaE	molybdopterin converting factor subunit 2	H	METABOLISM	Rv0866	1.21	0.455	Up
MSMEG_4976	isochorismatase hydrolase	Q	METABOLISM		1.18	0.457	Up
MSMEG_5848	amino acid permease	E	METABOLISM		1.42	0.457	Up
hemL	glutamate-1-semialdehyde aminotransferase	H	METABOLISM	Rv0524	1.80	0.459	Up
MSMEG_2787	hypothetical protein	H	METABOLISM	Rv2671	1.59	0.462	Up
MSMEG_4250	membrane transport ATPase	P	METABOLISM	RV2184C	1.10	0.463	Up
MSMEG_1574	glutamate decarboxylase	E	METABOLISM	Rv3432c	1.12	0.467	Up
MSMEG_4896	fatty-acid--CoA ligase	Q	METABOLISM		1.53	0.467	Up
MSMEG_1069	amino acid permease	E	METABOLISM		1.07	0.469	Up
MSMEG_4469	cobalt transporter	P	METABOLISM	RV2325C	1.94	0.469	Up
cobG	precorrin-3B synthase	P	METABOLISM	Rv2064	1.09	0.470	Up
MSMEG_3873	cobalamin biosynthesis protein cobIJ	H	METABOLISM		1.24	0.473	Up
MSMEG_0580	hypothetical protein	I	METABOLISM		1.55	0.474	Up
MSMEG_4545	extracellular solute-binding protein	E	METABOLISM	Rv3666c	1.26	0.479	Up
MSMEG_3007	succinate-semialdehyde dehydrogenase	C	METABOLISM		1.76	0.479	Up
MSMEG_1589	hypothetical protein	C	METABOLISM		1.03	0.481	Up
cobB	cobyrinic acid a,c-diamide synthase	H	METABOLISM	Rv2848c	1.14	0.486	Up
ptsI	phosphoenolpyruvate-protein phosphotrans	G	METABOLISM		1.40	0.486	Up
pdxH	pyridoxamine 5'-phosphate oxidase	H	METABOLISM	Rv2607	1.07	0.488	Up
MSMEG_3671	integral membrane protein	G	METABOLISM		1.02	0.490	Up
MSMEG_6095	D-amino acid deaminase	E	METABOLISM		1.32	0.493	Up
MSMEG_4467	ABC transporter permease	G	METABOLISM	Rv2317	1.35	0.493	Up
MSMEG_1764	L-lysine aminotransferase	E	METABOLISM	Rv3290c	1.04	0.500	Up
MSMEG_6230	acyltransferase	I	METABOLISM		1.81	0.502	Up
MSMEG_1491	histidinol-phosphate aminotransferase 2	E	METABOLISM		1.06	0.503	Up
MSMEG_1371	hypothetical protein	G	METABOLISM		1.44	0.504	Up
mdcB	triphosphoribosyl-dephospho-CoA synthase	H	METABOLISM		1.27	0.505	Up
rpe	ribulose-phosphate 3-epimerase	G	METABOLISM	Rv1408	1.41	0.507	Up
edd	phosphogluconate dehydratase	G	METABOLISM		1.30	0.507	Up
MSMEG_0318	AMP-dependent synthetase/ligase	Q	METABOLISM		1.58	0.508	Up
MSMEG_3571	thioesterase	Q	METABOLISM		1.57	0.508	Up
MSMEG_0812	amino acid transporter	E	METABOLISM		1.19	0.513	Up
MSMEG_2470	acyl-CoA thioesterase	I	METABOLISM		1.42	0.515	Up
MSMEG_3783	acyl-CoA dehydrogenase	I	METABOLISM	Rv2789c	1.48	0.517	Up
MSMEG_3693	cytochrome P450 monooxygenase	Q	METABOLISM		1.24	0.517	Up
MSMEG_4348	hypothetical protein	H	METABOLISM	Rv3509c	1.38	0.526	Up
MSMEG_5908	acyl-CoA synthetase	Q	METABOLISM	Rv3506	1.53	0.533	Up
MSMEG_0503	DeoR family transcriptional regulator	G	METABOLISM		1.14	0.534	Up
MSMEG_2130	acyl-CoA dehydrogenase	I	METABOLISM		1.09	0.543	Up
folB	dihydroneopterin aldolase	H	METABOLISM	Rv3607c	1.08	0.544	Up
MSMEG_0932	ROK family protein	G	METABOLISM	RV0485	1.09	0.546	Up
bioB	biotin synthase	H	METABOLISM	Rv1589	1.66	0.548	Up
MSMEG_2116	PTS system, glucose-specific IIBC component	G	METABOLISM		1.54	0.549	Up
MSMEG_5674	membrane transporter	G	METABOLISM		1.27	0.553	Up
pyrD	dihydroorotate dehydrogenase 2	F	METABOLISM	Rv2139	1.26	0.555	Up
bioA	adenosylmethionine-8-amino-7-oxononanoa	H	METABOLISM	Rv1568	1.26	0.555	Up
MSMEG_5045	D-2-hydroxyacid dehydrogenase	C	METABOLISM	RV0161	1.45	0.558	Up
MSMEG_2431	amidohydrolase	Q	METABOLISM	RV2915C	1.09	0.558	Up
MSMEG_0470	para-nitrobenzyl esterase	I	METABOLISM		1.11	0.562	Up
MSMEG_6225	proton antiporter efflux pump	G	METABOLISM		1.74	0.562	Up
cobT	nicotinate-nucleotide--dimethylbenzimidazo	H	METABOLISM	Rv2207	1.84	0.565	Up
MSMEG_1463	gamma-glutamyltranspeptidase	E	METABOLISM		1.05	0.570	Up
MSMEG_1581	hypothetical protein	Q	METABOLISM		1.78	0.570	Up
cobN	cobaltochelatase subunit CobN	H	METABOLISM	Rv2062c	1.10	0.571	Up
MSMEG_1899	succinyl-CoA:3-ketoacid-coenzyme A transfer	I	METABOLISM	Rv2503c	1.63	0.577	Up
MSMEG_2121	multiphosphoryl transfer protein (MTP)	G	METABOLISM		1.28	0.580	Up
MSMEG_1906	toluate 1,2-dioxygenase electron transfer co	C	METABOLISM		1.13	0.589	Up
MSMEG_0810	pyrimidine permease RutG	F	METABOLISM		1.61	0.589	Up

MSMEG_0276	aldehyde-alcohol dehydrogenase	C	METABOLISM		1.42	0.593	Up
MSMEG_3870	alpha-ketoglutarate-dependent taurine dioxy	Q	METABOLISM		1.58	0.593	Up
MSMEG_1376	xylulose kinase	G	METABOLISM	Rv0729	1.07	0.595	Up
MSMEG_2492	D-lactate dehydrogenase	C	METABOLISM		1.05	0.599	Up
MSMEG_3670	transporter small multidrug resistance (SMR)	P	METABOLISM		1.02	0.606	Up
MSMEG_1701	purine nucleoside phosphorylase	F	METABOLISM		1.04	0.608	Up
MSMEG_4511	linear gramicidin synthetase subunit B	Q	METABOLISM	Rv2380c	1.04	0.610	Up
MSMEG_2738	biotin sulfoxide reductase	C	METABOLISM	Rv1442	1.06	0.610	Up
MSMEG_1214	oxidoreductase	G	METABOLISM		1.03	0.617	Up
MSMEG_3058	lipoprotein, nlpa family protein	P	METABOLISM		1.08	0.624	Up
mdcH	malonate decarboxylase subunit epsilon	I	METABOLISM		1.19	0.633	Up
MSMEG_1903	caib/baif family protein	C	METABOLISM	RV1866	1.02	0.639	Up
MSMEG_5684	phosphoserine aminotransferase	E	METABOLISM	Rv0884c	1.22	0.652	Up
purN	phosphoribosylglycinamide formyltransferase	F	METABOLISM	Rv0956	1.21	0.669	Up
MSMEG_6399	antigen 85-C	R	POORLY CHARACTERIZED		1.75	0.000	Up
MSMEG_1684	hypothetical protein	R	POORLY CHARACTERIZED	RV3311	1.69	0.008	Up
MSMEG_1563	short-chain dehydrogenase/reductase SDR	R	POORLY CHARACTERIZED		1.24	0.013	Up
fbiC	FO synthase	R	POORLY CHARACTERIZED		1.28	0.017	Up
MSMEG_1932	MmpS3 protein	S	POORLY CHARACTERIZED	RV3209	2.05	0.025	Up
MSMEG_2940	hypothetical protein	S	POORLY CHARACTERIZED	RV2603C	1.68	0.028	Up
MSMEG_0657	Rieske (2Fe-2S) domain-containing protein	R	POORLY CHARACTERIZED	RV3161C	1.05	0.030	Up
murQ	N-acetylmuramic acid 6-phosphate etherase	R	POORLY CHARACTERIZED		1.23	0.034	Up
MSMEG_0101	hypothetical protein	S	POORLY CHARACTERIZED	RV1890C	1.28	0.037	Up
MSMEG_6126	D-isomer specific 2-hydroxyacid dehydrogena	R	POORLY CHARACTERIZED		1.13	0.039	Up
MSMEG_1638	roadblock/LC7 domain-contain protein	R	POORLY CHARACTERIZED	RV3364C	1.31	0.041	Up
MSMEG_1382	MmpL5 protein	R	POORLY CHARACTERIZED		1.48	0.045	Up
MSMEG_4470	ABC transporter	R	POORLY CHARACTERIZED	RV2326C	2.39	0.047	Up
MSMEG_0527	2-hydroxycyclohexnecarboxyl-CoA dehydrog	R	POORLY CHARACTERIZED		1.10	0.052	Up
MSMEG_5234	short-chain dehydrogenase/reductase SDR	R	POORLY CHARACTERIZED		1.68	0.053	Up
MSMEG_0332	2-nitropropane dioxygenase	R	POORLY CHARACTERIZED		1.29	0.054	Up
MSMEG_0141	mce associated transmembrane protein	S	POORLY CHARACTERIZED	RV0176	1.09	0.055	Up
MSMEG_1954	ABC transporter	R	POORLY CHARACTERIZED	RV3197	1.60	0.058	Up
trmB	tRNA (guanine-N(7)-)-methyltransferase	R	POORLY CHARACTERIZED		1.09	0.058	Up
MSMEG_3528	ErfK/YbiS/YcfS/YnhG family protein	S	POORLY CHARACTERIZED	RV0116C	1.08	0.065	Up
MSMEG_2159	hypothetical protein	S	POORLY CHARACTERIZED		1.54	0.066	Up
MSMEG_2157	hypothetical protein	S	POORLY CHARACTERIZED		1.73	0.068	Up
MSMEG_0399	hypothetical protein	S	POORLY CHARACTERIZED	Rv2377c	2.16	0.069	Up
MSMEG_3781	hypothetical protein	S	POORLY CHARACTERIZED	RV1645C	1.49	0.074	Up
MSMEG_1829	F420-0--gamma-glutamyl ligase	S	POORLY CHARACTERIZED		1.80	0.075	Up
MSMEG_1425	creatininase subfamily protein	R	POORLY CHARACTERIZED	RV0695	1.04	0.076	Up
MSMEG_5466	oxidoreductase	R	POORLY CHARACTERIZED		1.24	0.079	Up
MSMEG_5258	steroid delta-isomerase	R	POORLY CHARACTERIZED		1.25	0.081	Up
MSMEG_0863	short chain dehydrogenase	R	POORLY CHARACTERIZED	RV0439C	1.63	0.082	Up
MSMEG_5183	3-hydroxyacyl-CoA dehydrogenase	R	POORLY CHARACTERIZED	RV1144	1.21	0.084	Up
MSMEG_4685	oxidoreductase	R	POORLY CHARACTERIZED		2.97	0.086	Up
MSMEG_2361	phosphoribosylglycinamide formyltransferase	S	POORLY CHARACTERIZED		1.64	0.089	Up
MSMEG_3127	hypothetical protein	R	POORLY CHARACTERIZED	RV1466	1.74	0.091	Up
MSMEG_4220	hypothetical protein	R	POORLY CHARACTERIZED	RV2148C	1.60	0.091	Up
MSMEG_1891	hypothetical protein	S	POORLY CHARACTERIZED	RV3226C	1.56	0.093	Up
MSMEG_5762	zinc-binding oxidoreductase	R	POORLY CHARACTERIZED		1.08	0.094	Up
MSMEG_1210	hypothetical protein	S	POORLY CHARACTERIZED		1.47	0.095	Up
MSMEG_6398	antigen 85-A	R	POORLY CHARACTERIZED	Rv1886c	1.78	0.096	Up
MSMEG_4975	flavin-nucleotide-binding protein	R	POORLY CHARACTERIZED		1.48	0.096	Up
MSMEG_3616	integral membrane protein	S	POORLY CHARACTERIZED	RV1861	1.70	0.102	Up
MSMEG_5511	von Willebrand factor A	R	POORLY CHARACTERIZED	RV0959	1.82	0.104	Up
MSMEG_4284	hypothetical protein	R	POORLY CHARACTERIZED	RV2216	1.20	0.107	Up
MSMEG_5210	hydrolase	R	POORLY CHARACTERIZED		1.00	0.110	Up
MSMEG_0836	carboxylate-amine ligase	S	POORLY CHARACTERIZED	RV0433	1.48	0.111	Up
MSMEG_4632	saccharopine dehydrogenase	S	POORLY CHARACTERIZED	RV2449C	1.11	0.113	Up
clpS	ATP-dependent Clp protease adaptor protein	S	POORLY CHARACTERIZED		2.19	0.115	Up
MSMEG_2402	dihydroxyacetone kinase	R	POORLY CHARACTERIZED	RV2974C	1.86	0.127	Up
MSMEG_6662	short chain dehydrogenase	R	POORLY CHARACTERIZED		1.52	0.128	Up
MSMEG_5204	oxidoreductase, short chain dehydrogenase/	R	POORLY CHARACTERIZED		1.21	0.130	Up
MSMEG_6796	hypothetical protein	R	POORLY CHARACTERIZED		1.13	0.133	Up
MSMEG_5858	short chain dehydrogenase	R	POORLY CHARACTERIZED	RV0769	1.41	0.151	Up
MSMEG_0216	3-hydroxyacyl-CoA dehydrogenase	R	POORLY CHARACTERIZED		1.79	0.155	Up
MSMEG_4308	5'-nucleotidase	R	POORLY CHARACTERIZED	RV2232	1.20	0.158	Up
MSMEG_3534	4-hydroxybenzoyl-CoA thioesterase	R	POORLY CHARACTERIZED		1.68	0.158	Up
MSMEG_2642	hypothetical protein	S	POORLY CHARACTERIZED		1.55	0.158	Up
MSMEG_1760	short-chain dehydrogenase/reductase SDR	R	POORLY CHARACTERIZED	RV0851C	1.82	0.161	Up
MSMEG_5430	retinol dehydrogenase 13	R	POORLY CHARACTERIZED		1.61	0.172	Up
MSMEG_2942	glyoxalase/bleomycin resistance protein/dio	R	POORLY CHARACTERIZED		1.69	0.176	Up
MSMEG_3027	hypothetical protein	R	POORLY CHARACTERIZED	RV2553C	1.24	0.178	Up
MSMEG_3515	3-alpha-(or 20-beta)-hydroxysteroid dehydro	R	POORLY CHARACTERIZED	Rv2002	1.32	0.181	Up
MSMEG_3570	hypothetical protein	S	POORLY CHARACTERIZED		1.84	0.182	Up
MSMEG_6098	chalcone/stilbene synthase	S	POORLY CHARACTERIZED	RV3603C	1.09	0.182	Up
MSMEG_6592	short-chain dehydrogenase/reductase SDR	R	POORLY CHARACTERIZED		1.14	0.185	Up
MSMEG_0737	dehydrogenase	R	POORLY CHARACTERIZED	RV1544	1.08	0.185	Up
MSMEG_4496	metalloprotease	R	POORLY CHARACTERIZED	RV2367C	1.03	0.186	Up
MSMEG_5761	cupin	S	POORLY CHARACTERIZED		1.21	0.187	Up
MSMEG_5862	short chain dehydrogenase	R	POORLY CHARACTERIZED	RV0765C	1.09	0.189	Up
MSMEG_6583	antigen 85-C	R	POORLY CHARACTERIZED		2.31	0.192	Up
MSMEG_5644	membrane protein	S	POORLY CHARACTERIZED	RV0180C	1.79	0.196	Up
MSMEG_0250	membrane protein, MmpL family protein	R	POORLY CHARACTERIZED	Rv0206c	1.04	0.197	Up
MSMEG_1379	hypothetical protein	R	POORLY CHARACTERIZED		1.40	0.202	Up
MSMEG_3508	alpha/beta hydrolase	R	POORLY CHARACTERIZED	RV1191	1.01	0.205	Up
MSMEG_1149	hypothetical protein	S	POORLY CHARACTERIZED	RV2390C	1.03	0.205	Up
MSMEG_3843	hypothetical protein	R	POORLY CHARACTERIZED	RV1628C	1.61	0.210	Up
MSMEG_3825	3-oxoacyl-ACP reductase	R	POORLY CHARACTERIZED		1.02	0.210	Up
MSMEG_4382	dehydrogenase/reductase SDR family protein	R	POORLY CHARACTERIZED		1.31	0.218	Up
MSMEG_0321	transcriptional regulator	R	POORLY CHARACTERIZED		1.68	0.221	Up
MSMEG_1702	amidohydrolase	R	POORLY CHARACTERIZED	Rv3306c	1.03	0.227	Up
MSMEG_3817	hypothetical protein	S	POORLY CHARACTERIZED	RV1632C	1.11	0.229	Up
MSMEG_4208	integral membrane protein	S	POORLY CHARACTERIZED		1.30	0.229	Up
obgE	GTPase ObgE	R	POORLY CHARACTERIZED		1.09	0.231	Up
MSMEG_2581	acetyltransferase	R	POORLY CHARACTERIZED	RV2867C	1.34	0.232	Up
MSMEG_4463	CalR5 protein	R	POORLY CHARACTERIZED	RV2314C	1.42	0.243	Up
MSMEG_0066	early secretory antigenic target, 6 kDa	S	POORLY CHARACTERIZED	Rv3875	1.56	0.243	Up
MSMEG_5885	short chain dehydrogenase	R	POORLY CHARACTERIZED	RV3485C	1.09	0.248	Up
MSMEG_2033	oxidoreductase, zinc-binding dehydrogenase	R	POORLY CHARACTERIZED	Rv3141	2.04	0.252	Up
MSMEG_3580	antigen 85-C	R	POORLY CHARACTERIZED	Rv0129c	2.63	0.252	Up
MSMEG_5681	ferredoxin/ferredoxin--NADP reductase	R	POORLY CHARACTERIZED	Rv0886	1.56	0.255	Up
ahpD	alkylhydroperoxidase	S	POORLY CHARACTERIZED	Rv2429	1.43	0.257	Up
MSMEG_4221	hypothetical protein	S	POORLY CHARACTERIZED	RV2149C	1.20	0.260	Up
MSMEG_2349	glycosyl hydrolase family protein	S	POORLY CHARACTERIZED	RV3031	1.02	0.261	Up
MSMEG_4477	hydrolase, alpha/beta hydrolase fold family p	R	POORLY CHARACTERIZED	Rv1900c	2.14	0.272	Up
MSMEG_4464	modulator of DNA gyrase	R	POORLY CHARACTERIZED	RV2315C	1.16	0.273	Up
MSMEG_0231	hypothetical protein	S	POORLY CHARACTERIZED	RV1898	2.23	0.275	Up

MSMEG_3254	RDD family protein	S	POORLY CHARACTERIZED		1.39	0.276	Up
MSMEG_0923	hypothetical protein	S	POORLY CHARACTERIZED	RV0479C	1.47	0.276	Up
MSMEG_3581	FabG protein	R	POORLY CHARACTERIZED		1.50	0.281	Up
MSMEG_4219	hypothetical protein	S	POORLY CHARACTERIZED		1.01	0.286	Up
MSMEG_0901	hypothetical protein	S	POORLY CHARACTERIZED	RV0459	1.15	0.290	Up
MSMEG_3607	short chain dehydrogenase	R	POORLY CHARACTERIZED		1.89	0.291	Up
MSMEG_6190	metallo-beta-lactamase	R	POORLY CHARACTERIZED	RV3677C	1.25	0.293	Up
MSMEG_6645	2-methylcitrate dehydratase	R	POORLY CHARACTERIZED	RV1130	1.02	0.299	Up
MSMEG_4702	ABC transporter permease	S	POORLY CHARACTERIZED	RV0102	1.18	0.304	Up
MSMEG_6929	hypothetical protein	R	POORLY CHARACTERIZED	RV3910	1.02	0.310	Up
MSMEG_5547	hypothetical protein	S	POORLY CHARACTERIZED		1.70	0.316	Up
MSMEG_6221	integral membrane protein	S	POORLY CHARACTERIZED	RV3694C	1.94	0.316	Up
MSMEG_5850	TetR family transcriptional regulator	S	POORLY CHARACTERIZED	RV0775	1.09	0.317	Up
MSMEG_5073	O-methyltransferase, family protein 3	R	POORLY CHARACTERIZED	RV1220C	1.51	0.317	Up
MSMEG_3081	hypothetical protein	S	POORLY CHARACTERIZED	Rv1423	1.50	0.322	Up
MSMEG_1577	hypothetical protein	R	POORLY CHARACTERIZED	RV3422C	1.47	0.323	Up
MSMEG_3484	cupin	S	POORLY CHARACTERIZED		1.17	0.326	Up
MSMEG_3241	hypothetical protein	S	POORLY CHARACTERIZED		2.75	0.327	Up
MSMEG_3252	hypothetical protein	S	POORLY CHARACTERIZED	RV2639C	1.42	0.337	Up
MSMEG_5851	esterase	R	POORLY CHARACTERIZED	RV0774C	1.66	0.337	Up
MSMEG_0763	antibiotic transporter	R	POORLY CHARACTERIZED		1.96	0.343	Up
MSMEG_1114	short chain dehydrogenase	R	POORLY CHARACTERIZED		1.08	0.345	Up
MSMEG_3542	hypothetical protein	S	POORLY CHARACTERIZED		1.34	0.346	Up
MSMEG_3079	hypothetical protein	R	POORLY CHARACTERIZED	RV1421	1.29	0.346	Up
MSMEG_0283	short chain dehydrogenase	R	POORLY CHARACTERIZED		1.13	0.349	Up
MSMEG_0984	trap transporter 4tm/12tm fusion protein	R	POORLY CHARACTERIZED		1.86	0.350	Up
MSMEG_0999	integral membrane protein	S	POORLY CHARACTERIZED	RV0541C	1.62	0.354	Up
MSMEG_5066	integral membrane protein	S	POORLY CHARACTERIZED	RV1231C	1.49	0.355	Up
MSMEG_1894	short chain dehydrogenase	R	POORLY CHARACTERIZED	RV3224	1.77	0.356	Up
MSMEG_0996	hypothetical protein	S	POORLY CHARACTERIZED	RV0540	2.60	0.365	Up
MSMEG_5830	hypothetical protein	S	POORLY CHARACTERIZED	Rv0798c	1.09	0.368	Up
MSMEG_3244	hypothetical protein	S	POORLY CHARACTERIZED		1.10	0.372	Up
rimI	ribosomal-protein-alanine acetyltransferase	R	POORLY CHARACTERIZED	Rv3420c	1.71	0.376	Up
MSMEG_2596	peptidase C26	R	POORLY CHARACTERIZED	RV2859C	1.04	0.382	Up
MSMEG_2936	hydrolase, nudix family protein	S	POORLY CHARACTERIZED	RV2609C	1.13	0.385	Up
MSMEG_1660	oxidoreductase, 2-nitropropane dioxygenase	R	POORLY CHARACTERIZED	RV0021C	1.33	0.385	Up
MSMEG_3255	DoxX subfamily protein	S	POORLY CHARACTERIZED		1.62	0.393	Up
MSMEG_3842	esterase	R	POORLY CHARACTERIZED		1.49	0.397	Up
MSMEG_2566	3-alpha-(or 20-beta)-hydroxysteroid dehydro	R	POORLY CHARACTERIZED		1.01	0.398	Up
MSMEG_3303	carboxylic ester hydrolase	R	POORLY CHARACTERIZED		1.45	0.401	Up
engA	GTP-binding protein EngA	R	POORLY CHARACTERIZED	Rv1713	1.23	0.402	Up
MSMEG_4462	sodium:solute symporter	R	POORLY CHARACTERIZED		1.20	0.409	Up
MSMEG_0865	membrane flanked domain-containing protei	S	POORLY CHARACTERIZED	RV1226C	1.40	0.410	Up
MSMEG_0929	ErfK/YbiS/YcfS/YnhG family protein	S	POORLY CHARACTERIZED	RV0483	2.01	0.412	Up
MSMEG_0930	serine 3-dehydrogenase	R	POORLY CHARACTERIZED	RV0484C	1.34	0.420	Up
MSMEG_4971	oxidoreductase	R	POORLY CHARACTERIZED	RV2781C	1.44	0.424	Up
MSMEG_3879	short chain dehydrogenase	R	POORLY CHARACTERIZED	RV2073C	1.71	0.430	Up
MSMEG_5203	DoxX subfamily protein	S	POORLY CHARACTERIZED		1.06	0.434	Up
MSMEG_2788	ATP/GTP-binding integral membrane protein	R	POORLY CHARACTERIZED	RV2670C	1.80	0.436	Up
MSMEG_1643	hypothetical protein	S	POORLY CHARACTERIZED		1.28	0.437	Up
MSMEG_6219	ATPase AAA	R	POORLY CHARACTERIZED	Rv3692	1.37	0.439	Up
MSMEG_2278	membrane-bound oxidoreductase	R	POORLY CHARACTERIZED		1.03	0.443	Up
MSMEG_5237	hypothetical protein	R	POORLY CHARACTERIZED	RV1101C	1.41	0.443	Up
MSMEG_3290	regulatory protein	R	POORLY CHARACTERIZED		1.97	0.451	Up
MSMEG_0089	chromosome condensation protein	R	POORLY CHARACTERIZED		1.20	0.452	Up
MSMEG_6069	CobW/P47K domain-containing protein	R	POORLY CHARACTERIZED	RV0106	1.26	0.463	Up
MSMEG_0090	hypothetical protein	S	POORLY CHARACTERIZED		1.04	0.464	Up
MSMEG_1070	3-oxoacyl-ACP reductase	R	POORLY CHARACTERIZED		1.21	0.467	Up
MSMEG_2767	hypothetical protein	R	POORLY CHARACTERIZED	RV2695	1.08	0.469	Up
MSMEG_1698	ammonia monooxygenase	R	POORLY CHARACTERIZED		1.08	0.469	Up
MSMEG_5383	dehydrogenase/reductase SDR family protei	R	POORLY CHARACTERIZED		1.12	0.470	Up
MSMEG_1641	hypothetical protein	S	POORLY CHARACTERIZED	RV3361C	1.36	0.471	Up
MSMEG_4577	DegV family protein	S	POORLY CHARACTERIZED	RV2417C	1.48	0.473	Up
MSMEG_5235	short chain dehydrogenase	R	POORLY CHARACTERIZED		1.56	0.477	Up
MSMEG_3619	short chain dehydrogenase	R	POORLY CHARACTERIZED	RV1856C	1.02	0.481	Up
MSMEG_2279	3-hydroxyacyl-CoA dehydrogenase	R	POORLY CHARACTERIZED		1.48	0.487	Up
MSMEG_1001	acetyltransferase, gnat family protein	R	POORLY CHARACTERIZED		1.33	0.488	Up
MSMEG_4894	hypothetical protein	S	POORLY CHARACTERIZED		1.40	0.491	Up
MSMEG_4502	16S ribosomal RNA methyltransferase RsmE	S	POORLY CHARACTERIZED		1.04	0.497	Up
MSMEG_1640	ATP/GTP-binding protein	R	POORLY CHARACTERIZED	RV3362C	1.35	0.498	Up
MSMEG_3062	hypothetical protein	S	POORLY CHARACTERIZED		1.91	0.502	Up
MSMEG_3170	hypothetical protein	S	POORLY CHARACTERIZED		1.09	0.503	Up
MSMEG_6160	ATP-dependent rna helicase, dead/deah box	R	POORLY CHARACTERIZED	RV3649	1.28	0.505	Up
MSMEG_1199	hypothetical protein	S	POORLY CHARACTERIZED	RV0585C	1.59	0.512	Up
MSMEG_2188	integral membrane protein	R	POORLY CHARACTERIZED		1.30	0.512	Up
MSMEG_5910	quinone binding protein	S	POORLY CHARACTERIZED		1.26	0.515	Up
MSMEG_0997	hypothetical protein	R	POORLY CHARACTERIZED		1.25	0.515	Up
MSMEG_6099	hypothetical protein	S	POORLY CHARACTERIZED	RV3604C	1.41	0.515	Up
MSMEG_0809	isoprenylcysteine carboxyl methyltransferase	S	POORLY CHARACTERIZED	RV1139C	1.50	0.522	Up
MSMEG_0224	O-methyltransferase MdmC	R	POORLY CHARACTERIZED	RV0187	1.30	0.535	Up
MSMEG_0621	low molecular weight protein antigen 7	S	POORLY CHARACTERIZED		1.36	0.537	Up
MSMEG_3083	nucleoside-diphosphate sugar epimerase	R	POORLY CHARACTERIZED		1.17	0.545	Up
MSMEG_5591	hypothetical protein	R	POORLY CHARACTERIZED	RV0923C	1.27	0.553	Up
MSMEG_4307	hypothetical protein	S	POORLY CHARACTERIZED	RV2230C	1.13	0.556	Up
MSMEG_1460	pyridine nucleotide-disulfide oxidoreductase	R	POORLY CHARACTERIZED		1.46	0.559	Up
MSMEG_4583	ATPase AAA	R	POORLY CHARACTERIZED	RV2426C	1.12	0.575	Up
MSMEG_5287	dehydrogenase	R	POORLY CHARACTERIZED		1.15	0.584	Up
MSMEG_2355	hypothetical protein	R	POORLY CHARACTERIZED	RV3027C	1.12	0.587	Up
MSMEG_3867	ATPase AAA	R	POORLY CHARACTERIZED	RV3163C	1.45	0.588	Up
MSMEG_3289	gp61 protein	R	POORLY CHARACTERIZED		1.35	0.602	Up
MSMEG_6220	lipoprotein	R	POORLY CHARACTERIZED	RV3693	1.41	0.603	Up
MSMEG_1689	3-oxoacyl-ACP reductase	R	POORLY CHARACTERIZED		1.90	0.604	Up
MSMEG_1195	hypothetical protein	S	POORLY CHARACTERIZED		1.00	0.620	Up
recX	recombination regulator RecX	R	POORLY CHARACTERIZED	Rv2736c	1.32	0.633	Up
MSMEG_1571	integral membrane protein	R	POORLY CHARACTERIZED		1.03	0.637	Up
MSMEG_2733	hypothetical protein	S	POORLY CHARACTERIZED	RV2728C	1.04	0.672	Up
MSMEG_0320	phosphotriesterase	R	POORLY CHARACTERIZED	RV0230C	1.10	0.672	Up
MSMEG_4375	3-oxoacyl-ACP reductase	R	POORLY CHARACTERIZED		1.02	0.690	Up

					LIX79 t96-1 vs. LIX70 t96-1		
GeneName	Product	COG_Code	Function	RV Number	Geomean_Fold	P-value	Regulation
MSMEG_1910	muconate cycloisomerase	R	POORLY CHARACTERIZED		-1.14	0.045	Down
MSMEG_2112	hypothetical protein	E	METABOLISM		-1.11	0.052	Down
MSMEG_1782	oxidoreductase, short chain dehydrogenas	R	POORLY CHARACTERIZED		-1.03	0.059	Down
MSMEG_3022	transglycosylase associated protein	S	POORLY CHARACTERIZED		-1.03	0.064	Down
MSMEG_6935	N-acetylmuramoyl-L-alanine amidase	M	CELLULAR PROCESSES AND SIGNALING	RV3915	-1.14	0.081	Down
MSMEG_5661	ABC transporter ATP-binding protein	V	CELLULAR PROCESSES AND SIGNALING		-1.62	0.109	Down

**MSMEG_0266**	arginine decarboxylase	E	METABOLISM	**RV2531C**	-1.15	0.122	Down
**MSMEG_1775**	cytochrome P450 monooxygenase	Q	METABOLISM		-1.50	0.148	Down
**MSMEG_0375**	phospholipase D	I	METABOLISM		-2.35	0.165	Down
**MSMEG_0670**	FAD dependent oxidoreductase	O	CELLULAR PROCESSES AND SIGNALING		-1.21	0.166	Down
**MSMEG_0536**	intracellular protease PfpI family protein	R	POORLY CHARACTERIZED		-1.33	0.233	Down
**MSMEG_5616**	glyoxalase/bleomycin resistance protein/d	R	POORLY CHARACTERIZED	**RV0911**	-1.28	0.274	Down
**MSMEG_6768**	halogenase	C	METABOLISM		-1.39	0.276	Down
**MSMEG_6769**	transporter monovalent cation:proton anti	P	METABOLISM		-1.03	0.298	Down
**MSMEG_3364**	RhtB family protein transporter	E	METABOLISM		-1.44	0.305	Down
**MSMEG_4618**	isochorismatase	Q	METABOLISM		-1.60	0.312	Down
**MSMEG_3930**	[NiFe] hydrogenase subunit gamma	C	METABOLISM		-2.17	0.344	Down
**MSMEG_3536**	sugar transporter	G	METABOLISM		-1.38	0.355	Down
**MSMEG_5605**	cytochrome bd ubiquinol oxidase subunit I	C	METABOLISM		-1.60	0.370	Down
**MSMEG_2855**	virulence factor Mce family protein	Q	METABOLISM		-1.41	0.387	Down
**MSMEG_4318**	hypothetical protein	P	METABOLISM		-1.02	0.400	Down
**MSMEG_3954**	trehalose 6-phosphate phosphorylase	G	METABOLISM		-1.65	0.439	Down
**wrbA**	NAD(P)H:quinone oxidoreductase, type IV	R	POORLY CHARACTERIZED		-1.08	0.459	Down
**MSMEG_4063**	amidohydrolase	R	POORLY CHARACTERIZED		-1.08	0.573	Down
**MSMEG_2563**	cytochrome P450	Q	METABOLISM		-1.21	0.005	Down
**MSMEG_5922**	lipid-transfer protein	I	METABOLISM	**Rv3522**	-1.27	0.007	Down
**MSMEG_6734**	dibenzothiophene desulfurization enzyme	C	METABOLISM		-2.01	0.008	Down
**MSMEG_3726**	alcohol dehydrogenase	G	METABOLISM		-1.76	0.011	Down
**MSMEG_6885**	MmcI protein	C	METABOLISM		-1.93	0.012	Down
**MSMEG_4822**	hypothetical protein	C	METABOLISM		-1.25	0.013	Down
**MSMEG_1392**	alcohol dehydrogenase	C	METABOLISM		-1.65	0.015	Down
**recC**	exodeoxyribonuclease V subunit gamma	L	INFORMATION STORAGE AND PROCESSING	**Rv0631c**	-1.14	0.017	Down
**anmK**	anhydro-N-acetylmuramic acid kinase	O	CELLULAR PROCESSES AND SIGNALING		-1.56	0.018	Down
**dnaA**	chromosomal replication initiation protein	L	INFORMATION STORAGE AND PROCESSING	**Rv0001**	-1.19	0.019	Down
**cheR**	chemotaxis protein CheR	T	CELLULAR PROCESSES AND SIGNALING		-1.47	0.019	Down
**MSMEG_6264**	oxidoreductase	E	METABOLISM		-1.44	0.020	Down
**MSMEG_2098**	fumarylacetoacetate hydrolase	Q	METABOLISM		-1.15	0.022	Down
**MSMEG_1972**	methane monooxygenase component C	C	METABOLISM		-1.12	0.023	Down
**MSMEG_2846**	ABC transporter permease	P	METABOLISM		-1.10	0.023	Down
**MSMEG_3362**	enoyl-CoA hydratase	I	METABOLISM		-1.39	0.027	Down
**MSMEG_1228**	sulfatase	P	METABOLISM		-1.03	0.028	Down
**MSMEG_2539**	thiopurine S-methyltransferase (tpmt) sup	J	INFORMATION STORAGE AND PROCESSING	**RV0560C**	-1.27	0.030	Down
**MSMEG_4814**	hypothetical protein	I	METABOLISM		-1.27	0.033	Down
**MSMEG_3318**	oxidoreductase	C	METABOLISM		-1.29	0.037	Down
**MSMEG_1570**	carboxylesterase	R	POORLY CHARACTERIZED		-1.02	0.037	Down
**MSMEG_2208**	acyl-CoA dehydrogenase	I	METABOLISM		-1.02	0.037	Down
**MSMEG_6710**	alpha/beta hydrolase	I	METABOLISM		-1.17	0.038	Down
**MSMEG_3994**	short chain dehydrogenase	R	POORLY CHARACTERIZED		-1.07	0.038	Down
**MSMEG_1319**	Asp/Glu racemase	Q	METABOLISM		-1.09	0.044	Down
**MSMEG_4420**	cupin	S	POORLY CHARACTERIZED		-1.89	0.044	Down
**MSMEG_3042**	bifunctional pyrimidine regulatory protein	F	METABOLISM	**Rv1379**	-1.15	0.044	Down
**MSMEG_3934**	phosphoenolpyruvate synthase	G	METABOLISM		-1.99	0.045	Down
**MSMEG_5124**	2,4-dienoyl-CoA reductase	C	METABOLISM	**Rv1175c**	-1.41	0.045	Down
**MSMEG_2146**	gp15 protein	S	POORLY CHARACTERIZED		-1.07	0.048	Down
**MSMEG_1303**	transcriptional regulator	K	INFORMATION STORAGE AND PROCESSING		-1.10	0.049	Down
**MSMEG_0447**	hypothetical protein	O	CELLULAR PROCESSES AND SIGNALING		-1.28	0.050	Down
**MSMEG_4646**	pyruvate synthase	C	METABOLISM	**RV2455C**	-1.15	0.050	Down
**MSMEG_3095**	D-ribose-binding periplasmic protein	G	METABOLISM		-1.08	0.050	Down
**MSMEG_4387**	ABC transporter permease	P	METABOLISM		-1.52	0.052	Down
**MSMEG_4066**	hypothetical protein	Q	METABOLISM		-1.12	0.054	Down
**pcaG**	protocatechuate 3,4-dioxygenase subunit a	Q	METABOLISM		-1.04	0.055	Down
**MSMEG_0635**	hypothetical protein	S	POORLY CHARACTERIZED	**RV0309**	-1.68	0.055	Down
**MSMEG_1448**	integral membrane transporter	P	METABOLISM		-1.36	0.056	Down
**pqqB**	pyrroloquinoline quinone biosynthesis prot	R	POORLY CHARACTERIZED		-1.55	0.059	Down
**ltaE**	L-threonine aldolase	E	METABOLISM		-1.04	0.061	Down
**MSMEG_0351**	virulence factor mce family protein	Q	METABOLISM		-1.41	0.063	Down
**MSMEG_6367**	glycosyl transferase family protein	R	POORLY CHARACTERIZED	**Rv3782**	-1.26	0.063	Down
**MSMEG_5198**	carnitinyl-CoA dehydratase	I	METABOLISM		-1.43	0.065	Down
**MSMEG_6763**	oxidoreductase	R	POORLY CHARACTERIZED	**RV0077C**	-1.70	0.065	Down
**MSMEG_2194**	MerR family transcriptional regulator	K	INFORMATION STORAGE AND PROCESSING		-1.04	0.067	Down
**MSMEG_0517**	sugar binding-protein dependent transport	G	METABOLISM	**RV2039C**	-1.32	0.067	Down
**MSMEG_0474**	glucosamine--fructose-6-phosphate amino	M	CELLULAR PROCESSES AND SIGNALING		-1.48	0.069	Down
**MSMEG_5130**	extracellular solute-binding protein	E	METABOLISM	**Rv1166**	-1.35	0.070	Down
**MSMEG_1797**	salicylate esterase	R	POORLY CHARACTERIZED		-1.11	0.072	Down
**MSMEG_1963**	transcriptional regulatory protein	R	POORLY CHARACTERIZED		-1.27	0.073	Down
**MSMEG_6686**	glutaryl-CoA dehydrogenase	I	METABOLISM		-1.06	0.074	Down
**MSMEG_0288**	FAD dependent oxidoreductase	E	METABOLISM		-1.39	0.075	Down
**MSMEG_5372**	sensor protein KdpD	T	CELLULAR PROCESSES AND SIGNALING	**Rv1028c**	-1.05	0.076	Down
**MSMEG_1086**	ABC transporter permease	P	METABOLISM		-1.67	0.076	Down
**MSMEG_1297**	hydroxydechloroatrazine ethylaminohydrol	R	POORLY CHARACTERIZED		-1.21	0.079	Down
**MSMEG_0734**	Rieske (2Fe-2S) domain-containing protei	R	POORLY CHARACTERIZED		-1.02	0.081	Down
**MSMEG_4393**	carboxyvinyl-carboxyphosphonate phospho	G	METABOLISM		-1.37	0.081	Down
**MSMEG_3929**	[NiFe] hydrogenase subunit delta	C	METABOLISM		-1.07	0.083	Down
**MSMEG_1151**	DNA-binding protein	K	INFORMATION STORAGE AND PROCESSING		-1.34	0.083	Down
**MSMEG_4110**	3-hydroxyacyl-CoA dehydrogenase	I	METABOLISM		-1.49	0.083	Down
**MSMEG_2254**	oxalate decarboxylase OxdC	R	POORLY CHARACTERIZED		-1.12	0.085	Down
**kdpB**	potassium-transporting ATPase subunit B	P	METABOLISM	**Rv1030**	-1.04	0.085	Down
**MSMEG_1034**	esterase	R	POORLY CHARACTERIZED		-1.18	0.086	Down
**cysE**	serine O-acetyltransferase	E	METABOLISM	**Rv2335**	-1.03	0.086	Down
**MSMEG_1486**	RNA polymerase sigma factor SigL	K	INFORMATION STORAGE AND PROCESSING		-1.07	0.089	Down
**argB**	acetylglutamate kinase	E	METABOLISM	**Rv1654**	-1.20	0.090	Down
**MSMEG_4606**	hypothetical protein	D	CELLULAR PROCESSES AND SIGNALING		-1.75	0.092	Down
**MSMEG_2081**	acyl-CoA dehydrogenase	I	METABOLISM	**Rv3139**	-1.21	0.096	Down
**MSMEG_0902**	cyclopropane-fatty-acyl-phospholipid synth	M	CELLULAR PROCESSES AND SIGNALING	**Rv0644c**	-1.27	0.099	Down
**MSMEG_4879**	hypothetical protein	C	METABOLISM		-1.49	0.100	Down
**MSMEG_3579**	transmembrane protein	M	CELLULAR PROCESSES AND SIGNALING	**RV2434C**	-1.36	0.100	Down
**MSMEG_3809**	hypothetical protein	C	METABOLISM		-1.43	0.102	Down
**MSMEG_3427**	metal dependent hydrolase	R	POORLY CHARACTERIZED		-1.67	0.102	Down
**MSMEG_6725**	ABC transporter ATP-binding protein	R	POORLY CHARACTERIZED		-1.03	0.104	Down
**MSMEG_4809**	Rieske (2Fe-2S) domain-containing protei	R	POORLY CHARACTERIZED		-1.65	0.109	Down
**MSMEG_4077**	enoyl-CoA hydratase	I	METABOLISM		-1.12	0.110	Down
**MSMEG_6718**	transporter major facilitator family protei	G	METABOLISM		-1.03	0.114	Down
**MSMEG_2102**	nitrate transport ATP-binding protein NrtD	P	METABOLISM		-1.19	0.114	Down
**MSMEG_2229**	enoyl-CoA hydratase	I	METABOLISM		-1.55	0.115	Down
**MSMEG_1796**	membrane protein	C	METABOLISM		-1.72	0.117	Down
**MSMEG_4853**	peptidase, M24 family protein	E	METABOLISM		-1.18	0.117	Down
**MSMEG_3017**	hypothetical protein	S	POORLY CHARACTERIZED	**RV2567**	-1.27	0.119	Down
**MSMEG_4731**	acyl-CoA synthetase	Q	METABOLISM	**Rv1521**	-1.32	0.119	Down
**MSMEG_3090**	ribose transporter permease RbsC	G	METABOLISM		-1.10	0.120	Down
**MSMEG_3970**	glutamyl-tRNA(Gln) amidotransferase sub	J	INFORMATION STORAGE AND PROCESSING	**RV3175**	-1.13	0.120	Down
**MSMEG_2209**	GntR family transcriptional regulator	K	INFORMATION STORAGE AND PROCESSING		-1.63	0.121	Down
**MSMEG_2845**	ABC transporter permease	P	METABOLISM		-1.29	0.122	Down
**purH**	bifunctional phosphoribosylaminoimidazol	F	METABOLISM	**Rv0957**	-1.16	0.125	Down
**MSMEG_3378**	beta-lactamase	V	CELLULAR PROCESSES AND SIGNALING		-1.80	0.126	Down

MSMEG_0125	integral membrane protein	S	POORLY CHARACTERIZED		-1.41	0.129	Down
MSMEG_5579	MarR family transcriptional regulator	K	INFORMATION STORAGE AND PROCESSING		-1.26	0.130	Down
MSMEG_1155	carnitinyl-CoA dehydratase	I	METABOLISM		-1.27	0.131	Down
MSMEG_4521	Tat (twin-arginine translocation) pathway	P	METABOLISM		-1.24	0.132	Down
MSMEG_1249	ISMsm7, transposase orfB	L	INFORMATION STORAGE AND PROCESSING		-1.04	0.133	Down
MSMEG_0506	ABC transporter permease	G	METABOLISM		-1.37	0.133	Down
MSMEG_0487	ABC transporter permease	P	METABOLISM		-1.00	0.134	Down
MSMEG_5520	hypothetical protein	C	METABOLISM	RV0953C	-1.15	0.134	Down
MSMEG_3948	acyltransferase	R	POORLY CHARACTERIZED		-2.45	0.139	Down
MSMEG_1964	mitomycin radical oxidase	C	METABOLISM	RV1774	-1.08	0.139	Down
MSMEG_3657	hypothetical protein	C	METABOLISM	RV1817	-1.35	0.139	Down
MSMEG_6389	arabinosyltransferase A	M	CELLULAR PROCESSES AND SIGNALING	Rv3795	-1.37	0.140	Down
MSMEG_0534	permease, major facilitator family protein	G	METABOLISM		-1.34	0.140	Down
MSMEG_4422	oxidoreductase	C	METABOLISM		-1.08	0.147	Down
MSMEG_2096	integral membrane protein	R	POORLY CHARACTERIZED		-2.15	0.148	Down
MSMEG_4135	lysine decarboxylase superfamily protein	R	POORLY CHARACTERIZED		-1.16	0.149	Down
MSMEG_0293	Rieske (2Fe-2S) domain-containing protei	R	POORLY CHARACTERIZED		-1.08	0.150	Down
MSMEG_1137	amino acid permease	E	METABOLISM		-1.35	0.151	Down
MSMEG_2069	phosphotransferase enzyme family protei	R	POORLY CHARACTERIZED		-1.00	0.152	Down
MSMEG_6263	glutamate synthase	E	METABOLISM		-1.08	0.154	Down
MSMEG_2108	hypothetical protein	R	POORLY CHARACTERIZED		-1.09	0.155	Down
MSMEG_0149	thiamine biosynthesis protein ThiC	H	METABOLISM	Rv0423c	-1.04	0.156	Down
MSMEG_2810	major facilitator superfamily protein	G	METABOLISM		-1.12	0.156	Down
MSMEG_6712	maleylacetate reductase	C	METABOLISM		-1.36	0.156	Down
MSMEG_5609	carotenoid oxygenase	Q	METABOLISM	RV0913C	-1.22	0.158	Down
MSMEG_3357	metal-dependent phosphohydrolase	R	POORLY CHARACTERIZED		-1.79	0.162	Down
MSMEG_2005	sugar phosphate isomerase/epimerase	G	METABOLISM		-1.33	0.164	Down
MSMEG_2807	two-component system response regulato	K	INFORMATION STORAGE AND PROCESSING		-1.43	0.164	Down
MSMEG_3557	amino acid permease	E	METABOLISM		-1.09	0.166	Down
MSMEG_5593	pyruvate dehydrogenase	H	METABOLISM		-1.24	0.166	Down
MSMEG_2909	starvation-sensing protein RspA	R	POORLY CHARACTERIZED		-1.20	0.166	Down
MSMEG_5954	cell surface polysaccharide biosynthesis	M	CELLULAR PROCESSES AND SIGNALING		-1.30	0.167	Down
MSMEG_3346	hydroxyacid aldolase	G	METABOLISM		-1.63	0.168	Down
MSMEG_0174	inner membrane protein	S	POORLY CHARACTERIZED		-1.14	0.169	Down
MSMEG_4386	ABC transporter permease	P	METABOLISM		-2.23	0.170	Down
MSMEG_2657	peptidase, M16 family protein	R	POORLY CHARACTERIZED	RV2782C	-1.19	0.170	Down
MSMEG_5021	alcohol dehydrogenase	R	POORLY CHARACTERIZED		-1.33	0.171	Down
MSMEG_0170	transmembrane transporter	G	METABOLISM		-1.77	0.172	Down
MSMEG_5509	hypothetical protein	R	POORLY CHARACTERIZED		-1.07	0.174	Down
MSMEG_5364	amidohydrolase	R	POORLY CHARACTERIZED		-1.29	0.178	Down
MSMEG_5420	Tat-translocated enzyme	P	METABOLISM		-1.15	0.178	Down
MSMEG_6872	beta-lactamase	V	CELLULAR PROCESSES AND SIGNALING		-1.41	0.179	Down
MSMEG_4062	glutamyl-tRNA(Gln) amidotransferase sub	J	INFORMATION STORAGE AND PROCESSING		-1.19	0.179	Down
MSMEG_3991	cyclase	R	POORLY CHARACTERIZED		-1.12	0.179	Down
MSMEG_4049	sugar transporter permease	G	METABOLISM		-1.31	0.184	Down
rpsO	30S ribosomal protein S15	J	INFORMATION STORAGE AND PROCESSING	Rv2785c	-1.06	0.185	Down
MSMEG_2464	2Fe-2S-binding domain-containing protein	C	METABOLISM		-1.65	0.186	Down
MSMEG_6745	GntR family transcriptional regulator	K	INFORMATION STORAGE AND PROCESSING		-1.07	0.187	Down
MSMEG_2067	methyltransferase type 12	H	METABOLISM	RV2675C	-1.30	0.187	Down
MSMEG_4093	oxidoreductase, aldo/keto reductase	C	METABOLISM	RV2298	-1.25	0.188	Down
MSMEG_0654	hypothetical protein	C	METABOLISM		-1.03	0.188	Down
MSMEG_4092	alkanal monooxygenase	C	METABOLISM		-1.05	0.188	Down
MSMEG_1506	TetR family transcriptional regulator	S	POORLY CHARACTERIZED		-1.21	0.189	Down
MSMEG_6296	5-exo-alcohol dehydrogenase	R	POORLY CHARACTERIZED		-1.41	0.190	Down
MSMEG_0438	periplasmic binding protein	P	METABOLISM	RV0265C	-1.23	0.192	Down
MSMEG_4793	virulence factor Mce family protein	Q	METABOLISM		-1.10	0.196	Down
MSMEG_2981	branched-chain amino acid ABC transporte	E	METABOLISM		-1.62	0.197	Down
MSMEG_1756	endonuclease VIII	L	INFORMATION STORAGE AND PROCESSING	Rv3297	-2.36	0.197	Down
cadA	cadmium-translocating P-type ATPase	P	METABOLISM	Rv1469	-1.07	0.198	Down
MSMEG_3517	HAD-superfamily hydrolase	E	METABOLISM		-1.14	0.200	Down
MSMEG_4456	hypothetical protein	S	POORLY CHARACTERIZED	RV2313C	-1.16	0.200	Down
MSMEG_0339	FMN-dependent monooxygenase	C	METABOLISM	RV3520C	-1.26	0.200	Down
MSMEG_6795	enoyl-CoA hydratase/isomerase	I	METABOLISM		-1.91	0.202	Down
MSMEG_2772	amino acid permease	E	METABOLISM	RV2690C	-1.35	0.202	Down
argS	arginyl-tRNA synthetase	J	INFORMATION STORAGE AND PROCESSING	Rv1292	-1.37	0.202	Down
MSMEG_5839	protease 2	E	METABOLISM	Rv0781	-1.12	0.203	Down
MSMEG_3411	MOSC domain-containing protein	S	POORLY CHARACTERIZED		-1.03	0.205	Down
MSMEG_3367	short-chain dehydrogenase/reductase SDR	R	POORLY CHARACTERIZED		-1.17	0.205	Down
tgt	queuine tRNA-ribosyltransferase	J	INFORMATION STORAGE AND PROCESSING		-1.07	0.207	Down
MSMEG_0126	mandelate racemase/muconate lactonizir	R	POORLY CHARACTERIZED		-1.29	0.208	Down
MSMEG_4949	HemK family modification methylase	J	INFORMATION STORAGE AND PROCESSING	Rv1300	-1.23	0.208	Down
MSMEG_6237	hypothetical protein	H	METABOLISM		-1.22	0.208	Down
MSMEG_6908	HTH-type transcriptional regulator	K	INFORMATION STORAGE AND PROCESSING	RV0043C	-1.09	0.208	Down
MSMEG_4020	enoyl-CoA hydratase/isomerase	I	METABOLISM		-1.11	0.212	Down
MSMEG_4610	IgiC	Q	METABOLISM		-1.07	0.212	Down
glyS	glycyl-tRNA synthetase	J	INFORMATION STORAGE AND PROCESSING		-1.34	0.213	Down
MSMEG_3931	[NiFe] hydrogenase subunit beta	C	METABOLISM		-2.43	0.215	Down
MSMEG_5979	transferase	J	INFORMATION STORAGE AND PROCESSING		-1.10	0.215	Down
MSMEG_0205	tetracenomycin polyketide synthesis hydro	S	POORLY CHARACTERIZED		-1.67	0.216	Down
MSMEG_4008	oxidoreductase, 2OG-Fe(II) oxygenase	R	POORLY CHARACTERIZED		-1.30	0.217	Down
MSMEG_6833	alcohol dehydrogenase	R	POORLY CHARACTERIZED		-2.49	0.217	Down
MSMEG_5671	prolyl-tRNA synthetase	S	POORLY CHARACTERIZED		-1.12	0.218	Down
MSMEG_1452	sulfatase-modifying factor 1	S	POORLY CHARACTERIZED		-1.20	0.219	Down
MSMEG_3556	integral membrane transporter	H	METABOLISM		-1.40	0.222	Down
MSMEG_0461	CinZ protein	S	POORLY CHARACTERIZED		-1.53	0.225	Down
MSMEG_3731	dipeptidase 2	E	METABOLISM		-1.40	0.226	Down
MSMEG_0568	radical SAM domain-containing protein	R	POORLY CHARACTERIZED		-3.52	0.227	Down
MSMEG_6317	lipolytic protein G-D-S-L	E	METABOLISM		-1.18	0.227	Down
MSMEG_6660	permease, cytosine/purines, uracil, thiami	F	METABOLISM		-1.09	0.229	Down
MSMEG_1231	inner membrane protein YidH	S	POORLY CHARACTERIZED	RV2272	-1.06	0.230	Down
MSMEG_6639	GntR family transcriptional regulator	K	INFORMATION STORAGE AND PROCESSING		-1.03	0.230	Down
MSMEG_0545	LuxR family transcriptional regulator	T	CELLULAR PROCESSES AND SIGNALING	RV0386	-1.32	0.231	Down
MSMEG_5818	virulence factor Mce family protein	Q	METABOLISM	Rv0169	-1.29	0.231	Down
MSMEG_4056	hypothetical protein	G	METABOLISM		-1.06	0.231	Down
MSMEG_3812	acyl-CoA thioesterase	I	METABOLISM		-1.05	0.232	Down
MSMEG_0157	oxalyl-CoA decarboxylase	H	METABOLISM	Rv0118c	-1.25	0.232	Down
MSMEG_5812	short chain dehydrogenase	R	POORLY CHARACTERIZED		-1.23	0.232	Down
MSMEG_4082	monoxygenase	C	METABOLISM		-1.33	0.233	Down
MSMEG_3563	drug transporter	G	METABOLISM	RV1877	-1.19	0.235	Down
MSMEG_5416	LpqU protein	M	CELLULAR PROCESSES AND SIGNALING	Rv1022	-1.05	0.237	Down
MSMEG_1154	formyl-coenzyme A transferase	C	METABOLISM		-1.25	0.238	Down
MSMEG_3273	glutamyl aminopeptidase, M42 family pro	G	METABOLISM		-1.20	0.241	Down
MSMEG_2172	dicarboxylate-carrier protein	P	METABOLISM		-1.03	0.241	Down
MSMEG_3960	transcriptional regulator	K	INFORMATION STORAGE AND PROCESSING		-1.08	0.241	Down
MSMEG_0281	choline dehydrogenase	E	METABOLISM		-1.60	0.243	Down
rpsF	30S ribosomal protein S6	J	INFORMATION STORAGE AND PROCESSING	Rv0053	-1.00	0.244	Down
MSMEG_4844	acyl-CoA dehydrogenase	I	METABOLISM		-1.09	0.244	Down
MSMEG_4736	hypothetical protein	S	POORLY CHARACTERIZED		-1.18	0.245	Down

MSMEG_5615	hypothetical protein	S	POORLY CHARACTERIZED		-1.17	0.245	Down
MSMEG_2165	transketolase	G	METABOLISM		-1.39	0.247	Down
MSMEG_6459	ferredoxin-dependent glutamate synthase	E	METABOLISM	Rv3859c	-1.02	0.247	Down
MSMEG_2525	amino acid permease	E	METABOLISM		-1.19	0.249	Down
MSMEG_0535	GntR family transcriptional regulator	K	INFORMATION STORAGE AND PROCESSING		-1.39	0.249	Down
MSMEG_5948	glycosyl transferase family protein	M	CELLULAR PROCESSES AND SIGNALING		-1.23	0.249	Down
MSMEG_0546	hypothetical protein	H	METABOLISM		-1.30	0.249	Down
MSMEG_2544	LysR family transcriptional regulator	K	INFORMATION STORAGE AND PROCESSING		-1.42	0.250	Down
MSMEG_4154	transposase, Mutator family protein	L	INFORMATION STORAGE AND PROCESSING	RV3431C	-1.53	0.250	Down
MSMEG_0376	AraC family transcriptional regulator	K	INFORMATION STORAGE AND PROCESSING		-1.20	0.250	Down
mhpA	3-(3-hydroxyphenyl)propionate hydroxylase	C	METABOLISM		-1.31	0.250	Down
MSMEG_5984	UDP-phosphate galactosephosphotransfer	M	CELLULAR PROCESSES AND SIGNALING		-1.06	0.250	Down
MSMEG_4123	3-hydroxyisobutyrate dehydrogenase	I	METABOLISM		-1.03	0.252	Down
MSMEG_2007	HpcE protein	Q	METABOLISM		-1.13	0.252	Down
MSMEG_0511	sugar isomerase	M	CELLULAR PROCESSES AND SIGNALING		-1.02	0.252	Down
MSMEG_4075	CoA-binding protein	C	METABOLISM		-1.28	0.253	Down
hypB	hydrogenase nickel incorporation protein H	K	INFORMATION STORAGE AND PROCESSING		-1.00	0.253	Down
MSMEG_0350	virulence factor Mce family protein	Q	METABOLISM	Rv1970	-1.02	0.254	Down
MSMEG_3382	ArsR family transcriptional regulator	K	INFORMATION STORAGE AND PROCESSING		-1.04	0.255	Down
MSMEG_3932	hypothetical protein	O	CELLULAR PROCESSES AND SIGNALING	Rv2031c	-3.02	0.256	Down
MSMEG_0778	transcriptional regulator	K	INFORMATION STORAGE AND PROCESSING		-1.50	0.258	Down
MSMEG_6705	regulatory protein	K	INFORMATION STORAGE AND PROCESSING		-1.28	0.258	Down
MSMEG_4103	alkanesulfonate monooxygenase	C	METABOLISM		-1.24	0.258	Down
MSMEG_2982	periplasmic binding protein	E	METABOLISM		-1.02	0.259	Down
MSMEG_2538	MarR family transcriptional regulator	K	INFORMATION STORAGE AND PROCESSING	RV2887	-1.46	0.259	Down
MSMEG_2844	ABC transporter ATP-binding protein	R	POORLY CHARACTERIZED		-1.59	0.261	Down
MSMEG_2918	short-chain dehydrogenase/reductase SDR	R	POORLY CHARACTERIZED		-1.18	0.261	Down
MSMEG_6880	hydrophobic amino acid ABC transporter	E	METABOLISM		-1.01	0.262	Down
MSMEG_0117	hydrolase	R	POORLY CHARACTERIZED	RV1190	-1.28	0.263	Down
MSMEG_2011	LacI family transcriptional regulator	K	INFORMATION STORAGE AND PROCESSING		-1.01	0.265	Down
MSMEG_0341	hypothetical protein	C	METABOLISM		-1.63	0.266	Down
MSMEG_0475	nucleotide-sugar dehydrogenase	M	CELLULAR PROCESSES AND SIGNALING		-1.33	0.266	Down
MSMEG_3373	major facilitator superfamily protein	G	METABOLISM		-1.42	0.266	Down
MSMEG_3544	hypothetical protein	C	METABOLISM		-1.19	0.267	Down
coaBC	bifunctional phosphopantothenoylcysteine	H	METABOLISM		-2.11	0.267	Down
MSMEG_5810	monooxygenase	C	METABOLISM		-1.84	0.267	Down
MSMEG_0531	acyl-CoA dehydrogenase	I	METABOLISM		-1.31	0.268	Down
MSMEG_4396	isochorismatase hydrolase	Q	METABOLISM		-1.21	0.268	Down
MSMEG_1133	bifunctional short chain isoprenyl diphosph	H	METABOLISM	Rv0562	-1.33	0.269	Down
MSMEG_4023	oxidoreductase	C	METABOLISM	RV0794C	-1.24	0.270	Down
MSMEG_6242	alcohol dehydrogenase	C	METABOLISM		-1.81	0.271	Down
MSMEG_1222	ISMsm6, transposase	L	INFORMATION STORAGE AND PROCESSING		-1.35	0.271	Down
MSMEG_0881	hypothetical protein	S	POORLY CHARACTERIZED		-1.10	0.272	Down
rpsT	30S ribosomal protein S20	J	INFORMATION STORAGE AND PROCESSING	Rv2412	-1.06	0.272	Down
sucD	succinyl-CoA synthetase subunit alpha	C	METABOLISM	Rv0952	-1.03	0.273	Down
MSMEG_2297	glutaredoxin	O	CELLULAR PROCESSES AND SIGNALING		-1.22	0.273	Down
MSMEG_3157	hypothetical protein	S	POORLY CHARACTERIZED	RV1491C	-1.05	0.273	Down
hypF	[NiFe] hydrogenase maturation protein Hy	O	CELLULAR PROCESSES AND SIGNALING		-1.24	0.273	Down
MSMEG_6358	mesocentin	S	POORLY CHARACTERIZED		-1.15	0.274	Down
MSMEG_2908	2-keto-3-deoxygluconate kinase	G	METABOLISM		-1.04	0.275	Down
MSMEG_6340	short-chain dehydrogenase/reductase SDR	R	POORLY CHARACTERIZED		-1.12	0.275	Down
MSMEG_2924	permease binding-protein component	M	CELLULAR PROCESSES AND SIGNALING		-1.21	0.276	Down
MSMEG_2926	glycine betaine/carnitine/choline transpor	E	METABOLISM		-1.10	0.276	Down
MSMEG_5443	dehydrogenase	C	METABOLISM		-1.10	0.276	Down
MSMEG_0781	amino acid permease	E	METABOLISM		-1.46	0.277	Down
MSMEG_0326	AMP-dependent synthetase/ligase	Q	METABOLISM		-1.50	0.278	Down
MSMEG_6939	Soj family protein	D	CELLULAR PROCESSES AND SIGNALING	Rv3918c	-1.29	0.278	Down
MSMEG_1458	tena/thi-4 family protein	H	METABOLISM		-1.32	0.278	Down
MSMEG_4855	amidohydrolase	R	POORLY CHARACTERIZED		-1.40	0.281	Down
hisG	ATP phosphoribosyltransferase	E	METABOLISM	Rv2121c	-1.05	0.282	Down
MSMEG_3715	linear gramicidin synthetase subunit C	Q	METABOLISM		-1.56	0.282	Down
MSMEG_5394	potassium-transporting ATPase subunit C	P	METABOLISM	Rv1031	-1.01	0.282	Down
MSMEG_0147	C-5 sterol desaturase	I	METABOLISM		-1.43	0.283	Down
MSMEG_4074	peroxisomal trans-2-enoyl-CoA reductase	R	POORLY CHARACTERIZED		-1.13	0.283	Down
MSMEG_3987	FAD dependent oxidoreductase	C	METABOLISM		-1.30	0.283	Down
MSMEG_4819	hypothetical protein	C	METABOLISM		-1.13	0.284	Down
MSMEG_4541	ABC transporter ATP-binding protein	R	POORLY CHARACTERIZED		-1.49	0.284	Down
MSMEG_2166	transketolase	G	METABOLISM	Rv3379c	-1.15	0.284	Down
MSMEG_3358	YaeQ protein	S	POORLY CHARACTERIZED		-1.16	0.286	Down
MSMEG_6668	ABC transporter periplasmic protein	P	METABOLISM		-1.16	0.286	Down
MSMEG_4119	3-hydroxybutyryl-CoA dehydratase	I	METABOLISM		-1.20	0.287	Down
MSMEG_0151	PntAB protein	C	METABOLISM	Rv0156	-1.40	0.287	Down
MSMEG_6851	phosphatidylethanolamine-binding protein	R	POORLY CHARACTERIZED	RV1910C	-1.44	0.287	Down
MSMEG_4357	ABC transporter ATP-binding protein	R	POORLY CHARACTERIZED		-1.32	0.288	Down
MSMEG_0514	alpha-galactosidase	G	METABOLISM		-1.25	0.289	Down
MSMEG_0270	aminoglycoside phosphotransferase	R	POORLY CHARACTERIZED		-1.06	0.289	Down
hydA	phenylhydantoinase	F	METABOLISM		-1.33	0.289	Down
MSMEG_6695	cytochrome P450	Q	METABOLISM		-1.11	0.289	Down
MSMEG_4551	monoxygenase	C	METABOLISM		-1.01	0.291	Down
MSMEG_2545	ribosomal RNA large subunit methyltransf	R	POORLY CHARACTERIZED		-2.38	0.291	Down
MSMEG_2236	thiolase	I	METABOLISM		-1.29	0.293	Down
MSMEG_3216	peroxiredoxin Q	O	CELLULAR PROCESSES AND SIGNALING	Rv1608c	-1.08	0.294	Down
MSMEG_6911	ABC transporter ATP-binding protein	E	METABOLISM		-1.30	0.294	Down
MSMEG_0338	acyl-CoA dehydrogenase	I	METABOLISM		-1.58	0.294	Down
MSMEG_4859	oxidoreductase, short chain dehydrogenas	R	POORLY CHARACTERIZED		-1.80	0.294	Down
MSMEG_3006	Fe-dependent alcohol dehydrogenase	C	METABOLISM		-1.18	0.296	Down
MSMEG_0185	MmpL6 protein	R	POORLY CHARACTERIZED		-1.10	0.296	Down
MSMEG_1138	alcohol dehydrogenase	C	METABOLISM		-1.01	0.296	Down
MSMEG_5595	MarR family transcriptional regulator	K	INFORMATION STORAGE AND PROCESSING		-1.00	0.296	Down
araB	ribulokinase	C	METABOLISM		-1.66	0.297	Down
MSMEG_3664	transporter monovalent cation:proton anti	P	METABOLISM	RV3236C	-1.01	0.298	Down
MSMEG_4118	acyl-CoA dehydrogenase	I	METABOLISM		-1.48	0.298	Down
MSMEG_2554	phosphotransferase enzyme family protei	R	POORLY CHARACTERIZED		-1.04	0.299	Down
glnT	glutamine synthetase	E	METABOLISM		-1.12	0.300	Down
MSMEG_4533	sulfate-binding protein	P	METABOLISM	Rv2400c	-2.10	0.300	Down
MSMEG_3936	universal stress protein family protein	T	CELLULAR PROCESSES AND SIGNALING		-2.34	0.302	Down
MSMEG_1177	cytosine/purines uracil thiamine allantoin	F	METABOLISM		-1.45	0.303	Down
kdpA	potassium-transporting ATPase A	P	METABOLISM	Rv1029	-1.05	0.304	Down
MSMEG_4850	short-chain dehydrogenase/reductase SDR	R	POORLY CHARACTERIZED		-1.12	0.304	Down
MSMEG_3730	Fis family transcriptional regulator	K	INFORMATION STORAGE AND PROCESSING		-1.21	0.305	Down
MSMEG_6708	epoxide hydrolase	R	POORLY CHARACTERIZED	Rv0134	-1.00	0.305	Down
MSMEG_1152	citrate-proton symporter	G	METABOLISM		-1.44	0.307	Down
MSMEG_3982	acyl-CoA dehydrogenase	I	METABOLISM		-1.64	0.307	Down
MSMEG_6838	esterase	R	POORLY CHARACTERIZED		-1.15	0.311	Down
MSMEG_4039	aryl-alcohol dehydrogenase	C	METABOLISM		-1.35	0.312	Down
MSMEG_2311	hypothetical protein	R	POORLY CHARACTERIZED		-1.33	0.312	Down
MSMEG_2681	proline imino-peptidase	R	POORLY CHARACTERIZED	Rv0840c	-1.14	0.313	Down
purC	phosphoribosylaminoimidazole-succinocar	F	METABOLISM	Rv0780	-1.06	0.314	Down

MSMEG_0481	FAD dependent oxidoreductase	C	METABOLISM		-1.52	0.316	Down
MSMEG_1123	cobalamin synthesis protein	R	POORLY CHARACTERIZED		-1.09	0.316	Down
MSMEG_4801	carveol dehydrogenase	R	POORLY CHARACTERIZED		-1.05	0.317	Down
MSMEG_6525	hypothetical protein	A	INFORMATION STORAGE AND PROCESSING		-1.56	0.317	Down
MSMEG_1330	MarR family transcriptional regulator	K	INFORMATION STORAGE AND PROCESSING		-1.13	0.318	Down
MSMEG_0212	lyase	S	POORLY CHARACTERIZED		-1.02	0.319	Down
MSMEG_1117	transcriptional regulator	K	INFORMATION STORAGE AND PROCESSING		-1.15	0.319	Down
MSMEG_5024	T/U mismatch-specific DNA glycosylase	L	INFORMATION STORAGE AND PROCESSING		-1.62	0.320	Down
MSMEG_5581	hypothetical protein	S	POORLY CHARACTERIZED		-1.04	0.320	Down
MSMEG_3400	glutamyl-tRNA(Gln) amidotransferase sub	J	INFORMATION STORAGE AND PROCESSING	Rv3375	-1.31	0.321	Down
MSMEG_0508	glycerol-phosphate porter	G	METABOLISM	RV2038C	-1.01	0.322	Down
MSMEG_4112	cyclohexanecarboxylate-CoA ligase	Q	METABOLISM		-1.58	0.323	Down
MSMEG_6790	AP endonuclease, family protein 2	G	METABOLISM		-1.02	0.323	Down
MSMEG_3578	cyclase	T	CELLULAR PROCESSES AND SIGNALING	RV2435C	-1.07	0.324	Down
MSMEG_1451	arylsulfatase	P	METABOLISM	Rv0711	-1.23	0.325	Down
MSMEG_2549	major facilitator superfamily protein	G	METABOLISM	RV1672C	-1.25	0.326	Down
MSMEG_4740	glycosyltransferase 28	C	METABOLISM		-1.25	0.326	Down
MSMEG_0524	short chain dehydrogenase	R	POORLY CHARACTERIZED		-1.00	0.326	Down
MSMEG_6835	Fatty acid desaturase	I	METABOLISM		-1.44	0.326	Down
MSMEG_0476	chitin synthase	M	CELLULAR PROCESSES AND SIGNALING		-1.10	0.327	Down
narH	nitrate reductase subunit beta	C	METABOLISM	Rv1162	-1.35	0.330	Down
MSMEG_0400	peptide synthetase	Q	METABOLISM		-3.13	0.330	Down
MSMEG_0349	virulence factor mce family protein	Q	METABOLISM	Rv1969	-1.32	0.332	Down
MSMEG_2217	AMP-dependent synthetase/ligase	Q	METABOLISM		-1.55	0.332	Down
MSMEG_2912	inner membrane metabolite transporter Y	G	METABOLISM		-1.34	0.332	Down
MSMEG_1795	2-deoxy-scyllo-inosamine dehydrogenase	R	POORLY CHARACTERIZED		-1.35	0.333	Down
MSMEG_2812	C-5 sterol desaturase	I	METABOLISM		-1.23	0.333	Down
MSMEG_2677	large membrane protein	R	POORLY CHARACTERIZED		-1.52	0.333	Down
rbsR	ribose operon repressor	K	INFORMATION STORAGE AND PROCESSING		-1.40	0.334	Down
MSMEG_1390	enoyl-CoA hydratase	I	METABOLISM	Rv0675	-1.05	0.334	Down
MSMEG_3945	universal stress protein family protein	T	CELLULAR PROCESSES AND SIGNALING		-2.57	0.334	Down
MSMEG_1798	major facilitator superfamily protein	G	METABOLISM		-1.02	0.335	Down
MSMEG_0486	ABC transporter periplasmic protein	P	METABOLISM		-1.30	0.336	Down
MSMEG_5626	luciferase	C	METABOLISM		-1.13	0.337	Down
pstS	phosphate ABC transporter substrate-bind	P	METABOLISM		-1.09	0.337	Down
MSMEG_2537	transporter protein	G	METABOLISM		-1.14	0.337	Down
MSMEG_1840	rubredoxin	C	METABOLISM	Rv3251c	-1.02	0.338	Down
MSMEG_3436	hypothetical protein	S	POORLY CHARACTERIZED	RV0366C	-1.02	0.339	Down
MSMEG_3434	peptidyl-prolyl cis-trans isomerase domain	O	CELLULAR PROCESSES AND SIGNALING		-1.04	0.341	Down
MSMEG_3953	hypothetical protein	I	METABOLISM	Rv0533c	-2.22	0.342	Down
MSMEG_4472	multidrug-efflux transporter protein	G	METABOLISM		-1.09	0.342	Down
MSMEG_2996	hypothetical protein	C	METABOLISM		-1.24	0.343	Down
MSMEG_2856	virulence factor Mce family protein	Q	METABOLISM		-1.32	0.343	Down
MSMEG_0655	glucose 1-dehydrogenase	R	POORLY CHARACTERIZED		-1.09	0.343	Down
MSMEG_1220	ABC transporter permease	E	METABOLISM		-1.47	0.344	Down
ampC	beta-lactamase	V	CELLULAR PROCESSES AND SIGNALING		-1.56	0.345	Down
MSMEG_1082	response regulator	K	INFORMATION STORAGE AND PROCESSING		-1.41	0.345	Down
MSMEG_3349	trap dicarboxylate transporter dctq subuni	Q	METABOLISM		-1.41	0.348	Down
MSMEG_6874	aldehyde dehydrogenase	C	METABOLISM		-1.35	0.348	Down
MSMEG_1607	tautomerase	R	POORLY CHARACTERIZED		-1.15	0.349	Down
MSMEG_5728	polysaccharide deacetylase	G	METABOLISM		-1.00	0.350	Down
MSMEG_1780	hypothetical protein	P	METABOLISM		-1.37	0.350	Down
MSMEG_0530	short chain dehydrogenase	R	POORLY CHARACTERIZED		-1.46	0.351	Down
MSMEG_0343	TetR family transcriptional regulator	K	INFORMATION STORAGE AND PROCESSING		-1.07	0.352	Down
MSMEG_0543	hypothetical protein	C	METABOLISM		-1.18	0.353	Down
MSMEG_2212	short chain dehydrogenase	R	POORLY CHARACTERIZED	RV2263	-1.15	0.353	Down
MSMEG_0158	formyl-coenzyme A transferase	C	METABOLISM		-1.14	0.354	Down
MSMEG_1140	NAD(P)H-dependent glycerol-3-phosphate	C	METABOLISM		-1.35	0.354	Down
MSMEG_0515	sugar transporter sugar binding lipoprotei	G	METABOLISM		-1.79	0.355	Down
MSMEG_1513	hypothetical protein	C	METABOLISM		-1.07	0.356	Down
MSMEG_6309	ABC transporter ATP-binding protein	E	METABOLISM		-1.28	0.356	Down
MSMEG_3040	beta-lactamase	V	CELLULAR PROCESSES AND SIGNALING	RV1367C	-1.30	0.358	Down
MSMEG_3805	oxidoreductase, short chain dehydrogenas	R	POORLY CHARACTERIZED		-1.16	0.358	Down
pcaH	protocatechuate 3,4-dioxygenase subunit I	Q	METABOLISM		-1.20	0.359	Down
MSMEG_3951	hypothetical protein	I	METABOLISM		-1.45	0.360	Down
pqqE	pyrroloquinoline quinone biosynthesis prot	R	POORLY CHARACTERIZED		-1.13	0.360	Down
MSMEG_0824	hypothetical protein	R	POORLY CHARACTERIZED	RV0421C	-1.15	0.361	Down
MSMEG_4389	monooxygenase, NtaA/SnaA/SoxA family	C	METABOLISM		-1.18	0.361	Down
MSMEG_0442	tetracycline repressor domain-containing	K	INFORMATION STORAGE AND PROCESSING		-1.08	0.362	Down
MSMEG_1751	norsolorinic acid reductase	C	METABOLISM	RV2298	-1.20	0.363	Down
MSMEG_6765	ABC transporter	V	CELLULAR PROCESSES AND SIGNALING		-1.15	0.363	Down
MSMEG_2894	steroid monooxygenase	P	METABOLISM		-1.38	0.365	Down
MSMEG_4765	MerR family transcriptional regulator	K	INFORMATION STORAGE AND PROCESSING		-1.30	0.365	Down
MSMEG_5953	hypothetical protein	E	METABOLISM		-1.06	0.367	Down
MSMEG_6854	3-isopropylmalate dehydratase large subu	E	METABOLISM		-1.80	0.367	Down
MSMEG_0576	MmpL4 protein	R	POORLY CHARACTERIZED		-1.50	0.368	Down
MSMEG_4785	mce-family protein mce1f	Q	METABOLISM		-1.25	0.369	Down
MSMEG_3944	two component transcriptional regulatory	K	INFORMATION STORAGE AND PROCESSING	Rv3133c	-1.49	0.370	Down
MSMEG_1979	antibiotic biosynthesis monooxygenase	S	POORLY CHARACTERIZED		-1.37	0.370	Down
MSMEG_1301	hypothetical protein	K	INFORMATION STORAGE AND PROCESSING		-1.35	0.371	Down
MSMEG_6774	enoyl-CoA hydratase	I	METABOLISM		-1.34	0.373	Down
MSMEG_5494	acyl-CoA dehydrogenase	I	METABOLISM	Rv0972c	-1.23	0.374	Down
MSMEG_4087	major facilitator superfamily protein	G	METABOLISM		-1.26	0.374	Down
MSMEG_0333	carboxyl transferase domain-containing pr	I	METABOLISM		-1.11	0.375	Down
MSMEG_0284	ribosyldihydronicotinamide dehydrogenase	R	POORLY CHARACTERIZED		-1.30	0.375	Down
MSMEG_0484	formamidase	C	METABOLISM		-1.28	0.376	Down
MSMEG_0772	phytase	S	POORLY CHARACTERIZED		-1.32	0.378	Down
MSMEG_5573	sugar ABC transporter permease	G	METABOLISM		-1.14	0.379	Down
rplM	50S ribosomal protein L13	J	INFORMATION STORAGE AND PROCESSING	Rv3443c	-1.03	0.379	Down
MSMEG_3449	DNA-binding protein	H	METABOLISM		-1.16	0.380	Down
soxR	redox-sensitive transcriptional activator So	K	INFORMATION STORAGE AND PROCESSING		-1.32	0.380	Down
MSMEG_1085	dipeptide transporter permease DppB	P	METABOLISM		-1.13	0.381	Down
MSMEG_4448	transcriptional modulator of MazE	T	CELLULAR PROCESSES AND SIGNALING		-1.01	0.381	Down
MSMEG_3248	branched-chain amino acid ABC transporte	E	METABOLISM		-1.10	0.381	Down
MSMEG_2042	phosphotransferase enzyme family protei	R	POORLY CHARACTERIZED	RV3168	-1.29	0.381	Down
MSMEG_3583	monooxygenase	C	METABOLISM		-1.11	0.382	Down
MSMEG_4048	cyclase	R	POORLY CHARACTERIZED		-1.43	0.383	Down
MSMEG_2239	TetR family transcriptional regulator	K	INFORMATION STORAGE AND PROCESSING		-1.45	0.384	Down
MSMEG_6469	hypothetical protein	S	POORLY CHARACTERIZED	RV0140	-1.27	0.386	Down
MSMEG_4450	alpha/beta hydrolase	E	METABOLISM		-1.11	0.386	Down
MSMEG_3947	6-phosphofructokinase	G	METABOLISM	Rv2029c	-2.23	0.386	Down
MSMEG_5109	hypothetical protein	R	POORLY CHARACTERIZED		-1.06	0.387	Down
MSMEG_6669	ABC transporter permease	R	POORLY CHARACTERIZED		-1.07	0.389	Down
MSMEG_5565	hypothetical protein	S	POORLY CHARACTERIZED		-1.35	0.390	Down
MSMEG_1306	aldehyde dehydrogenase	C	METABOLISM		-1.53	0.391	Down
MSMEG_2171	L-carnitine dehydratase/bile acid-inducible	C	METABOLISM		-1.44	0.392	Down
MSMEG_5802	oxidoreductase, short chain dehydrogenas	R	POORLY CHARACTERIZED		-1.21	0.393	Down
MSMEG_5194	integral membrane protein	G	METABOLISM		-1.01	0.394	Down

MSMEG_2978	ABC transporter ATP-binding protein	E	METABOLISM		-1.33	0.394	Down
MSMEG_4335	diacylglycerol kinase	R	POORLY CHARACTERIZED		-1.06	0.396	Down
MSMEG_5523	peptidase	E	METABOLISM		-1.05	0.398	Down
MSMEG_6496	nitrate/sulfonate/bicarbonate ABC transpo	P	METABOLISM		-2.91	0.401	Down
MSMEG_6564	TetR family transcriptional regulator	K	INFORMATION STORAGE AND PROCESSING		-1.45	0.401	Down
MSMEG_0148	TetR family transcriptional regulator	K	INFORMATION STORAGE AND PROCESSING		-1.41	0.402	Down
MSMEG_6941	R3H domain-containing protein	R	POORLY CHARACTERIZED	RV3920C	-1.23	0.402	Down
MSMEG_0128	thioesterase	R	POORLY CHARACTERIZED	RV0163	-1.41	0.404	Down
MSMEG_4397	MerR family transcriptional regulator	K	INFORMATION STORAGE AND PROCESSING		-1.11	0.405	Down
hybA	hydrogenase-2, small subunit	C	METABOLISM		-1.39	0.406	Down
narI	respiratory nitrate reductase subunit gam	C	METABOLISM	Rv1164	-1.78	0.407	Down
MSMEG_1977	alcohol dehydrogenase	R	POORLY CHARACTERIZED		-1.32	0.407	Down
MSMEG_3905	hypothetical protein	S	POORLY CHARACTERIZED	RV2117	-1.01	0.407	Down
MSMEG_4140	GntR family transcriptional regulator	E	METABOLISM		-1.38	0.408	Down
MSMEG_1281	hypothetical protein	S	POORLY CHARACTERIZED		-1.07	0.408	Down
rpoZ	DNA-directed RNA polymerase subunit om	K	INFORMATION STORAGE AND PROCESSING	Rv1390	-1.11	0.408	Down
MSMEG_3942	hypothetical protein	S	POORLY CHARACTERIZED	RV2004C	-2.29	0.409	Down
MSMEG_2252	flavin-type hydroxylase	C	METABOLISM		-1.31	0.409	Down
MSMEG_2879	acyl-CoA dehydrogenase	I	METABOLISM		-1.31	0.409	Down
MSMEG_2162	MmcJ protein	C	METABOLISM		-1.72	0.410	Down
MSMEG_3301	hypothetical protein	C	METABOLISM		-1.40	0.410	Down
MSMEG_4798	L-carnitine dehydratase/bile acid-inducible	C	METABOLISM		-1.01	0.410	Down
MSMEG_0485	amidase	J	INFORMATION STORAGE AND PROCESSING		-1.14	0.411	Down
MSMEG_5632	oxidoreductase, short chain dehydrogenas	R	POORLY CHARACTERIZED		-1.23	0.413	Down
MSMEG_4102	hypothetical protein	I	METABOLISM		-1.58	0.414	Down
MSMEG_1173	ArsR family transcriptional regulator	K	INFORMATION STORAGE AND PROCESSING	RV2642	-1.25	0.414	Down
MSMEG_0526	oxidoreductase, GMC family protein	E	METABOLISM		-1.51	0.414	Down
oppD	ABC transporter ATP-binding protein	P	METABOLISM	RV1281C	-1.30	0.414	Down
MSMEG_0817	LysR family transcriptional regulator	K	INFORMATION STORAGE AND PROCESSING		-1.01	0.415	Down
MSMEG_2241	acyl-CoA synthetase	Q	METABOLISM		-1.03	0.416	Down
MSMEG_4155	transposase A	L	INFORMATION STORAGE AND PROCESSING		-1.20	0.417	Down
MSMEG_1125	ArsR family transcriptional regulator	K	INFORMATION STORAGE AND PROCESSING	RV2621C	-1.14	0.418	Down
MSMEG_1178	transcriptional regulator	K	INFORMATION STORAGE AND PROCESSING		-1.44	0.418	Down
MSMEG_1509	TetR family transcriptional regulator	K	INFORMATION STORAGE AND PROCESSING		-1.17	0.421	Down
MSMEG_2456	5,10-methylenetetrahydromethanopterin r	C	METABOLISM		-1.12	0.424	Down
MSMEG_6700	regulatory protein	K	INFORMATION STORAGE AND PROCESSING		-1.27	0.425	Down
MSMEG_1211	Fatty acid desaturase	I	METABOLISM		-1.40	0.427	Down
MSMEG_0839	ATP-dependent protease La	R	POORLY CHARACTERIZED	RV0434	-1.14	0.429	Down
MSMEG_0912	3-hydroxybutyryl-CoA dehydrogenase	I	METABOLISM	Rv0468	-1.15	0.429	Down
MSMEG_0428	nitrite reductase [NAD(P)H] small subunit	R	POORLY CHARACTERIZED	Rv0253	-1.40	0.429	Down
MSMEG_6780	phenazine biosynthesis protein PhzF	R	POORLY CHARACTERIZED		-1.47	0.431	Down
MSMEG_6776	LysR family transcriptional regulator	K	INFORMATION STORAGE AND PROCESSING		-1.02	0.431	Down
mtnN	MTA/SAH nucleosidase	F	METABOLISM		-1.32	0.431	Down
MSMEG_5807	D-methionine transport ATP-binding prote	P	METABOLISM		-1.11	0.434	Down
MSMEG_3369	transporter	G	METABOLISM		-1.12	0.434	Down
MSMEG_5479	type I antifreeze protein	S	POORLY CHARACTERIZED	RV0991C	-1.08	0.435	Down
MSMEG_2598	short chain dehydrogenase	R	POORLY CHARACTERIZED	RV2857C	-1.86	0.436	Down
MSMEG_2039	transcriptional regulator	K	INFORMATION STORAGE AND PROCESSING		-1.02	0.439	Down
MSMEG_5112	hypothetical protein	A	INFORMATION STORAGE AND PROCESSING		-1.08	0.439	Down
MSMEG_6037	2-hydroxy-6-ketonona-2,4-dienedioic acid	R	POORLY CHARACTERIZED		-1.21	0.440	Down
MSMEG_5968	polysaccharide biosynthesis protein	R	POORLY CHARACTERIZED		-1.22	0.440	Down
dcd	deoxycytidine triphosphate deaminase	F	METABOLISM	Rv0321	-1.35	0.441	Down
MSMEG_0532	TetR family transcriptional regulator	K	INFORMATION STORAGE AND PROCESSING		-1.06	0.441	Down
MSMEG_5395	sensor protein KdpD	T	CELLULAR PROCESSES AND SIGNALING	Rv1028c	-1.31	0.442	Down
MSMEG_2666	multimeric flavodoxin WrbA	R	POORLY CHARACTERIZED	RV2771C	-1.99	0.442	Down
MSMEG_4823	cytochrome p450	Q	METABOLISM		-1.11	0.444	Down
MSMEG_1158	5-oxovalerate dehydrogenase	C	METABOLISM		-1.39	0.446	Down
MSMEG_6510	multidrug ABC transporter	V	CELLULAR PROCESSES AND SIGNALING		-1.10	0.447	Down
MSMEG_4002	oxidoreductase, zinc-binding dehydrogena	R	POORLY CHARACTERIZED		-1.21	0.449	Down
MSMEG_4858	TetR family transcriptional regulator	K	INFORMATION STORAGE AND PROCESSING		-1.08	0.449	Down
MSMEG_3513	hypothetical protein	R	POORLY CHARACTERIZED	Rv2416c	-1.36	0.450	Down
MSMEG_3402	cytosine permease	F	METABOLISM		-1.29	0.450	Down
MSMEG_1975	amidohydrolase	R	POORLY CHARACTERIZED		-1.22	0.453	Down
MSMEG_2952	transporter	G	METABOLISM		-1.01	0.453	Down
MSMEG_2193	TetR family transcriptional regulator	K	INFORMATION STORAGE AND PROCESSING		-1.35	0.455	Down
MSMEG_3398	integral membrane transporter	G	METABOLISM		-1.16	0.456	Down
MSMEG_2600	regulatory protein	K	INFORMATION STORAGE AND PROCESSING		-1.66	0.457	Down
MSMEG_0196	dehydrogenase	C	METABOLISM		-1.30	0.458	Down
MSMEG_1074	polysaccharide deacetylase	G	METABOLISM		-1.08	0.459	Down
MSMEG_3348	extracellular solute-binding protein	G	METABOLISM		-1.05	0.462	Down
MSMEG_5140	nitrate reductase subunit alpha	C	METABOLISM	Rv1161	-1.06	0.462	Down
MSMEG_1298	guanine deaminase	R	POORLY CHARACTERIZED		-1.17	0.468	Down
MSMEG_4004	3-oxoacyl-ACP reductase	R	POORLY CHARACTERIZED		-1.29	0.469	Down
MSMEG_5778	alcohol dehydrogenase	R	POORLY CHARACTERIZED		-1.19	0.484	Down
MSMEG_5931	short chain dehydrogenase	R	POORLY CHARACTERIZED	RV3530C	-1.05	0.484	Down
MSMEG_6300	GntR family transcriptional regulator	K	INFORMATION STORAGE AND PROCESSING		-1.36	0.488	Down
MSMEG_5317	hypothetical protein	A	INFORMATION STORAGE AND PROCESSING	RV0314C	-1.22	0.489	Down
MSMEG_5184	alpha-methylacyl-CoA racemase	C	METABOLISM	Rv1143	-1.34	0.490	Down
MSMEG_4886	major facilitator family protein transporte	G	METABOLISM		-1.72	0.492	Down
MSMEG_0469	PadR family transcriptional regulator	K	INFORMATION STORAGE AND PROCESSING		-1.78	0.493	Down
MSMEG_4439	acetyltransferase	J	INFORMATION STORAGE AND PROCESSING		-1.38	0.493	Down
MSMEG_3823	3-oxoacyl-ACP reductase	R	POORLY CHARACTERIZED		-1.48	0.493	Down
MSMEG_4795	ABC transporter	Q	METABOLISM		-1.10	0.494	Down
MSMEG_6643	DNA-binding protein	R	POORLY CHARACTERIZED	RV1129C	-1.13	0.494	Down
MSMEG_6870	creatinine amidohydrolase	R	POORLY CHARACTERIZED		-1.75	0.494	Down
MSMEG_3927	peptidase M52, hydrogen uptake protein	C	METABOLISM		-1.23	0.495	Down
MSMEG_1351	cyclopropane-fatty-acyl-phospholipid synth	M	CELLULAR PROCESSES AND SIGNALING	Rv3392c	-1.36	0.495	Down
MSMEG_0217	alcohol dehydrogenase	C	METABOLISM		-1.14	0.497	Down
MSMEG_1100	DGPFAETKE domain-containing protein	S	POORLY CHARACTERIZED		-1.36	0.500	Down
MSMEG_2286	aminoglycoside phosphotransferase	R	POORLY CHARACTERIZED		-1.03	0.500	Down
MSMEG_0195	steroid monooxygenase	P	METABOLISM		-1.29	0.505	Down
MSMEG_5366	hypothetical protein	G	METABOLISM		-1.09	0.506	Down
MSMEG_4014	N-carbamoyl-L-amino acid amidohydrolas	E	METABOLISM		-1.07	0.506	Down
MSMEG_1235	sulfate permease	P	METABOLISM		-1.02	0.515	Down
MSMEG_4005	calcium-binding protein	G	METABOLISM		-1.20	0.516	Down
MSMEG_5475	acetate operon repressor	K	INFORMATION STORAGE AND PROCESSING		-1.09	0.517	Down
MSMEG_1254	DEAD/DEAH box helicase	R	POORLY CHARACTERIZED		-1.10	0.522	Down
MSMEG_5291	long-chain-fatty-acid--CoA ligase	Q	METABOLISM	Rv1058	-1.15	0.524	Down
MSMEG_1745	transcriptional regulator	K	INFORMATION STORAGE AND PROCESSING		-1.08	0.526	Down
MSMEG_3940	universal stress protein family protein	T	CELLULAR PROCESSES AND SIGNALING		-1.92	0.529	Down
MSMEG_1823	hypothetical protein	I	METABOLISM	RV3268	-1.10	0.530	Down
MSMEG_4001	ribose transporter permease RbsC	G	METABOLISM		-1.07	0.534	Down
MSMEG_5950	colanic acid biosynthesis acetyltransferase	R	POORLY CHARACTERIZED		-1.40	0.537	Down
MSMEG_3965	hypothetical protein	R	POORLY CHARACTERIZED		-1.26	0.538	Down
MSMEG_0777	F420-dependent glucose-6-phosphate deh	C	METABOLISM	Rv0407	-1.09	0.539	Down
MSMEG_3939	universal stress protein family protein	T	CELLULAR PROCESSES AND SIGNALING	RV2624C	-1.82	0.542	Down
MSMEG_5458	cyclic nucleotide-binding protein	T	CELLULAR PROCESSES AND SIGNALING	RV0998	-1.03	0.543	Down
MSMEG_6002	coenzyme A transferase subunit A	I	METABOLISM	RV3551	-1.23	0.544	Down

**MSMEG_0390**	acyltransferase	I	METABOLISM		**-1.15**	**0.546**	**Down**
**MSMEG_6716**	AP endonuclease, family protein 2	G	METABOLISM		**-1.42**	**0.551**	**Down**
**ltrA**	low temperature requirement protein LtrA	S	POORLY CHARACTERIZED		**-1.36**	**0.552**	**Down**
**MSMEG_4070**	TetR family transcriptional regulator	K	INFORMATION STORAGE AND PROCESSING		**-1.21**	**0.555**	**Down**
**MSMEG_4006**	CdaR family transcriptional regulator	Q	METABOLISM		**-1.24**	**0.556**	**Down**
**ackA**	acetate kinase	C	METABOLISM	**Rv0409**	**-1.26**	**0.568**	**Down**
**MSMEG_2247**	dihydrokaempferol 4-reductase	G	METABOLISM		**-1.60**	**0.569**	**Down**
**MSMEG_5612**	amino-acid acetyltransferase	J	INFORMATION STORAGE AND PROCESSING		**-1.49**	**0.573**	**Down**
**MSMEG_0513**	integral membrane protein	S	POORLY CHARACTERIZED		**-1.52**	**0.577**	**Down**
**MSMEG_1192**	M23 peptidase domain-containing protein	M	CELLULAR PROCESSES AND SIGNALING		**-1.04**	**0.587**	**Down**
**MSMEG_3933**	hypothetical protein	R	POORLY CHARACTERIZED		**-1.22**	**0.587**	**Down**
**MSMEG_0175**	FAD dependent oxidoreductase	E	METABOLISM		**-1.09**	**0.604**	**Down**
**MSMEG_6738**	transcriptional regulator YdhC	K	INFORMATION STORAGE AND PROCESSING		**-1.15**	**0.695**	**Down**
**MSMEG_5732**	monooxygenase	C	METABOLISM		**-1.00**	**0.767**	**Down**

Table S8. Genes Up / Down regulated in LIX80 (K55M Mut PknK Overexpression Strain at t=96 h)

Upregulated
For filtering upregulation we consider Geomean fold >=1 in treated sample.

Downregulated
For filtering downregulation we consider Geomean fold <=-1 in treated sample.

Expression fold values are provided in terms of log base 2

					LIX80 t96-1 vs. LIX70 t96-1		
GeneName	Product	COG_Code	Function	RV Number	Geomean_Fold	P-value	Regulation
dnaK	molecular chaperone DnaK	O	CELLULAR PROCESSES AND SIGNALING	Rv0350	1.49	0.293	Up
MSMEG_5611	spore protein	O	CELLULAR PROCESSES AND SIGNALING		1.54	0.531	Up
sppA	signal peptide peptidase SppA, 67K type	U	CELLULAR PROCESSES AND SIGNALING	Rv0724	1.33	0.000	Up
msrB	methionine-R-sulfoxide reductase	O	CELLULAR PROCESSES AND SIGNALING	RV2674	1.27	0.010	Up
MSMEG_4303	methyltransferase	O	CELLULAR PROCESSES AND SIGNALING		1.14	0.181	Up
MSMEG_0699	hypothetical protein	O	CELLULAR PROCESSES AND SIGNALING		1.55	0.221	Up
MSMEG_6366	O-antigen export system, ATP-binding protein	M	CELLULAR PROCESSES AND SIGNALING	Rv3781	1.11	0.444	Up
MSMEG_1765	restriction endonuclease	V	CELLULAR PROCESSES AND SIGNALING		1.28	0.115	Up
grpE	co-chaperone GrpE	O	CELLULAR PROCESSES AND SIGNALING	Rv0351	1.51	0.195	Up
clpP	ATP-dependent Clp protease proteolytic subunit	U	CELLULAR PROCESSES AND SIGNALING	Rv2460c	1.50	0.197	Up
MSMEG_0732	chaperone ClpB	O	CELLULAR PROCESSES AND SIGNALING	Rv0384c	1.38	0.260	Up
MSMEG_2553	TetR family transcriptional regulator	K	INFORMATION STORAGE AND PROCESSING		1.08	0.344	Up
MSMEG_2652	iron repressor protein	K	INFORMATION STORAGE AND PROCESSING	Rv2788	1.10	0.049	Up
MSMEG_1168	transcriptional regulator	K	INFORMATION STORAGE AND PROCESSING		1.07	0.150	Up
MSMEG_0916	TetR family transcriptional regulator	K	INFORMATION STORAGE AND PROCESSING	RV0472C	1.32	0.180	Up
MSMEG_1025	TetR family transcriptional regulator	K	INFORMATION STORAGE AND PROCESSING		1.08	0.183	Up
MSMEG_5651	LuxR family transcriptional regulator	K	INFORMATION STORAGE AND PROCESSING		1.28	0.287	Up
MSMEG_6565	hypothetical protein	L	INFORMATION STORAGE AND PROCESSING	RV0730	1.02	0.328	Up
MSMEG_5935	ATP-dependent DNA helicase	L	INFORMATION STORAGE AND PROCESSING		1.05	0.334	Up
MSMEG_5723	endoribonuclease L-psp family protein	J	INFORMATION STORAGE AND PROCESSING		1.39	0.382	Up
MSMEG_1632	hypothetical protein	L	INFORMATION STORAGE AND PROCESSING		1.05	0.410	Up
MSMEG_4474	acyl-CoA oxidase	I	METABOLISM		1.63	0.002	Up
add	adenosine deaminase	F	METABOLISM	Rv3313c	1.66	0.044	Up
MSMEG_0889	succinate-semialdehyde dehydrogenase	C	METABOLISM		1.44	0.044	Up
MSMEG_6020	sugar ABC transporter periplasmic protein	G	METABOLISM		1.36	0.047	Up
MSMEG_5538	[NADP+] succinate-semialdehyde dehydrogenase	C	METABOLISM		1.14	0.107	Up
MSMEG_1665	gamma-aminobutyraldehyde dehydrogenase	C	METABOLISM		1.24	0.161	Up
MSMEG_2017	MerR family transcriptional regulator	H	METABOLISM		2.20	0.164	Up
manB	phosphomannomutase	G	METABOLISM		1.07	0.168	Up
rocD	ornithine--oxo-acid transaminase	E	METABOLISM	Rv2321c	1.53	0.174	Up
MSMEG_5442	glutamate dehydrogenase	E	METABOLISM		1.51	0.181	Up
MSMEG_5699	ThiS family protein	H	METABOLISM	Rv0868c	1.06	0.181	Up
MSMEG_5485	molybdopterin biosynthesis protein	H	METABOLISM	Rv0984	1.13	0.191	Up
MSMEG_3125	cysteine desulfurase	E	METABOLISM	Rv1464	1.13	0.207	Up
MSMEG_3568	hypothetical protein	P	METABOLISM		1.39	0.307	Up
MSMEG_6250	glutamate--cysteine ligase	H	METABOLISM	Rv3704c	1.08	0.363	Up
cysM	cysteine synthase B	E	METABOLISM	Rv1336	1.32	0.464	Up
MSMEG_1710	ribose transporter permease RbsC	G	METABOLISM		1.15	0.014	Up
MSMEG_1712	ABC transporter periplasmic protein	G	METABOLISM		1.03	0.059	Up
argD	acetylornithine aminotransferase	E	METABOLISM	Rv1655	1.52	0.073	Up
nuoG	NADH dehydrogenase subunit G	C	METABOLISM	Rv3151	1.28	0.159	Up
MSMEG_0444	agmatine deiminase	E	METABOLISM		1.04	0.170	Up
MSMEG_1711	ABC transporter ATP-binding protein	G	METABOLISM		1.45	0.171	Up
MSMEG_5734	alpha-methylacyl-CoA racemase	C	METABOLISM	Rv0855	1.16	0.176	Up
MSMEG_5310	SAM-dependent methyltransferase	H	METABOLISM	RV2622	1.33	0.184	Up
MSMEG_2466	glutaryl-CoA dehydrogenase	I	METABOLISM	Rv0400c	1.17	0.228	Up
MSMEG_1926	acid phosphatase	G	METABOLISM	Rv3214	1.21	0.239	Up
MSMEG_1180	allantoate amidohydrolase	E	METABOLISM		1.15	0.242	Up
cysA	sulfate ABC transporter ATP-binding protein	P	METABOLISM		1.25	0.274	Up
MSMEG_1387	acyl-CoA dehydrogenase	I	METABOLISM	Rv0672	1.26	0.307	Up
MSMEG_5145	extracellular solute-binding protein	G	METABOLISM		1.35	0.316	Up
MSMEG_0894	dihydrodipicolinate reductase	E	METABOLISM		1.23	0.321	Up
nuoK	NADH dehydrogenase subunit K	C	METABOLISM	Rv3155	1.06	0.342	Up
MSMEG_5014	ATPase P	P	METABOLISM		1.19	0.391	Up
MSMEG_1124	ferredoxin FdxA	C	METABOLISM		1.27	0.415	Up
MSMEG_0159	formate dehydrogenase subunit gamma	C	METABOLISM		1.50	0.442	Up
ehuC	ectoine/hydroxyectoine ABC transporter permease	E	METABOLISM		1.03	0.453	Up
MSMEG_5366	hypothetical protein	G	METABOLISM		1.39	0.516	Up
MSMEG_5608	acetyl-CoA acetyltransferase	I	METABOLISM	RV0914C	1.00	0.633	Up
MSMEG_0836	carboxylate-amine ligase	S	POORLY CHARACTERIZED	RV0433	1.19	0.030	Up
ahpD	alkylhydroperoxidase	S	POORLY CHARACTERIZED	Rv2429	1.20	0.074	Up
MSMEG_0399	hypothetical protein	S	POORLY CHARACTERIZED	Rv2377c	1.25	0.260	Up
MSMEG_6399	antigen 85-C	R	POORLY CHARACTERIZED		1.70	0.363	Up
MSMEG_5511	von Willebrand factor A	R	POORLY CHARACTERIZED	RV0959	1.07	0.423	Up
MSMEG_2026	short chain dehydrogenase	R	POORLY CHARACTERIZED		1.15	0.025	Up
MSMEG_1984	haloacetate dehalogenase H-1	R	POORLY CHARACTERIZED	Rv0134	1.45	0.113	Up
MSMEG_2254	oxalate decarboxylase OxdC	R	POORLY CHARACTERIZED		1.17	0.118	Up
MSMEG_1959	hypothetical protein	S	POORLY CHARACTERIZED	RV3193C	1.02	0.139	Up
MSMEG_6278	metallo-beta-lactamase superfamily protein	R	POORLY CHARACTERIZED		1.03	0.283	Up

					LIX80 t96-1 vs. LIX70 t96-1		
GeneName	Product	COG_Code	Function	RV Number	Geomean_Fold	P-value	Regulation
MSMEG_5661	ABC transporter ATP-binding protein	V	CELLULAR PROCESSES AND SIGNALING		-1.18	0.357	Down
MSMEG_2855	virulence factor Mce family protein	Q	METABOLISM		-1.91	0.472	Down
MSMEG_4743	hypothetical protein	S	POORLY CHARACTERIZED		-1.28	0.022	Down
MSMEG_5177	TetR family transcriptional regulator	K	INFORMATION STORAGE AND PROCESSING		-1.03	0.134	Down
MSMEG_0782	class III aminotransferase	E	METABOLISM		-1.05	0.500	Down
MSMEG_3460	ferric uptake regulation protein	P	METABOLISM		-1.13	0.524	Down
MSMEG_1742	oxidoreductase	C	METABOLISM		-1.29	0.578	Down
MSMEG_6854	3-isopropylmalate dehydratase large subunit	E	METABOLISM		-1.03	0.009	Down
rpoZ	DNA-directed RNA polymerase subunit omega	K	INFORMATION STORAGE AND PROCESSING	Rv1390	-1.25	0.020	Down
MSMEG_3427	metal dependent hydrolase	R	POORLY CHARACTERIZED		-1.19	0.062	Down

MSMEG_3357	metal-dependent phosphohydrolase	R	POORLY CHARACTERIZED		-1.25	0.158	Down
MSMEG_3936	universal stress protein family protein	T	CELLULAR PROCESSES AND SIGNALING		-1.98	0.231	Down
MSMEG_3933	hypothetical protein	R	POORLY CHARACTERIZED		-2.20	0.235	Down
MSMEG_0568	radical SAM domain-containing protein	R	POORLY CHARACTERIZED		-2.30	0.287	Down
ampC	beta-lactamase	V	CELLULAR PROCESSES AND SIGNALING		-1.21	0.327	Down
MSMEG_3953	hypothetical protein	I	METABOLISM	Rv0533c	-2.74	0.345	Down
MSMEG_3939	universal stress protein family protein	T	CELLULAR PROCESSES AND SIGNALING	RV2624C	-1.90	0.354	Down
MSMEG_6833	alcohol dehydrogenase	R	POORLY CHARACTERIZED		-1.70	0.360	Down
MSMEG_4533	sulfate-binding protein	P	METABOLISM	Rv2400c	-1.73	0.391	Down
MSMEG_1756	endonuclease VIII	L	INFORMATION STORAGE AND PROCESSING	Rv3297	-1.84	0.399	Down
MSMEG_3948	acyltransferase	R	POORLY CHARACTERIZED		-1.09	0.407	Down
coaBC	bifunctional phosphopantothenoylcysteine decarbo	H	METABOLISM		-1.82	0.415	Down
MSMEG_4386	ABC transporter permease	P	METABOLISM		-1.02	0.428	Down
MSMEG_4859	oxidoreductase, short chain dehydrogenase/reducta	R	POORLY CHARACTERIZED		-1.76	0.437	Down
MSMEG_1192	M23 peptidase domain-containing protein	M	CELLULAR PROCESSES AND SIGNALING		-1.23	0.438	Down
MSMEG_3954	trehalose 6-phosphate phosphorylase	G	METABOLISM		-1.21	0.443	Down
MSMEG_3945	universal stress protein family protein	T	CELLULAR PROCESSES AND SIGNALING		-2.08	0.458	Down
MSMEG_0400	peptide synthetase	Q	METABOLISM		-2.62	0.464	Down
MSMEG_2598	short chain dehydrogenase	R	POORLY CHARACTERIZED	RV2857C	-1.46	0.468	Down
MSMEG_2545	ribosomal RNA large subunit methyltransferase N	R	POORLY CHARACTERIZED		-2.15	0.475	Down
MSMEG_6496	nitrate/sulfonate/bicarbonate ABC transporter ATPa	P	METABOLISM		-1.92	0.478	Down
MSMEG_3940	universal stress protein family protein	T	CELLULAR PROCESSES AND SIGNALING		-2.29	0.494	Down
MSMEG_3930	[NiFe] hydrogenase subunit gamma	C	METABOLISM		-1.40	0.498	Down
MSMEG_3947	6-phosphofructokinase	G	METABOLISM	Rv2029c	-2.20	0.503	Down
MSMEG_3931	[NiFe] hydrogenase subunit beta	C	METABOLISM		-1.35	0.544	Down
MSMEG_1249	ISMsm7, transposase orfB	L	INFORMATION STORAGE AND PROCESSING		-1.14	0.549	Down
MSMEG_5732	monooxygenase	C	METABOLISM		-1.59	0.552	Down
MSMEG_6738	transcriptional regulator YdhC	K	INFORMATION STORAGE AND PROCESSING		-1.91	0.567	Down
MSMEG_2247	dihydrokaempferol 4-reductase	G	METABOLISM		-1.38	0.570	Down
MSMEG_3944	two component transcriptional regulatory protein d	K	INFORMATION STORAGE AND PROCESSING	Rv3133c	-1.11	0.573	Down
MSMEG_3942	hypothetical protein	S	POORLY CHARACTERIZED	RV2004C	-2.63	0.584	Down
MSMEG_3932	hypothetical protein	O	CELLULAR PROCESSES AND SIGNALING	Rv2031c	-1.93	0.620	Down
MSMEG_6870	creatinine amidohydrolase	R	POORLY CHARACTERIZED		-1.01	0.733	Down
rpmD	50S ribosomal protein L30	J	INFORMATION STORAGE AND PROCESSING	Rv0722	-1.11	0.009	Down
MSMEG_6853	3-isopropylmalate dehydratase small subunit	E	METABOLISM		-1.48	0.011	Down
MSMEG_3845	hypothetical protein	S	POORLY CHARACTERIZED		-1.01	0.028	Down
MSMEG_5221	N-acetyltransferase GCN5	J	INFORMATION STORAGE AND PROCESSING		-1.13	0.048	Down
MSMEG_5603	ArsR family transcriptional regulator	K	INFORMATION STORAGE AND PROCESSING	Rv1994c	-1.05	0.078	Down
MSMEG_2651	alkanesulfonate monooxygenase	C	METABOLISM		-1.77	0.084	Down
MSMEG_2264	peptidase M52, hydrogen uptake protein	C	METABOLISM		-1.01	0.088	Down
rplN	50S ribosomal protein L14	J	INFORMATION STORAGE AND PROCESSING	Rv0714	-1.03	0.092	Down
MSMEG_1348	RNA polymerase ECF-subfamily protein sigma factor	K	INFORMATION STORAGE AND PROCESSING		-1.27	0.095	Down
MSMEG_3297	CadC family transcriptional regulator	K	INFORMATION STORAGE AND PROCESSING		-1.16	0.112	Down
MSMEG_5920	FMN-dependent monooxygenase	C	METABOLISM	RV3520C	-1.27	0.124	Down
scpA	segregation and condensation protein A	S	POORLY CHARACTERIZED		-1.23	0.126	Down
rplO	50S ribosomal protein L15	J	INFORMATION STORAGE AND PROCESSING	Rv0723	-1.57	0.134	Down
MSMEG_6195	ion-transporting ATPase	P	METABOLISM	RV3680	-1.09	0.135	Down
MSMEG_5373	nitrilase 2	R	POORLY CHARACTERIZED		-1.31	0.166	Down
MSMEG_4984	hypothetical protein	Q	METABOLISM	RV1532C	-1.19	0.175	Down
MSMEG_4033	TetR family transcriptional regulator	K	INFORMATION STORAGE AND PROCESSING		-1.27	0.178	Down
MSMEG_4126	hydrolase, isochorismatase	Q	METABOLISM		-1.39	0.203	Down
MSMEG_1445	30S ribosomal protein S17	J	INFORMATION STORAGE AND PROCESSING	Rv0710	-1.23	0.220	Down
MSMEG_3843	hypothetical protein	R	POORLY CHARACTERIZED	RV1628C	-1.08	0.222	Down
MSMEG_0502	glucosidase	G	METABOLISM		-1.10	0.247	Down
MSMEG_2857	virulence factor Mce family protein	Q	METABOLISM		-1.05	0.277	Down
MSMEG_5851	esterase	R	POORLY CHARACTERIZED	RV0774C	-1.10	0.300	Down
MSMEG_2994	amidohydrolase	R	POORLY CHARACTERIZED		-1.16	0.303	Down
MSMEG_0283	short chain dehydrogenase	R	POORLY CHARACTERIZED		-1.04	0.304	Down
MSMEG_4463	CalR5 protein	R	POORLY CHARACTERIZED	RV2314C	-1.25	0.308	Down
MSMEG_0649	phosphonate-binding periplasmic protein	P	METABOLISM		-1.23	0.309	Down
MSMEG_1914	RNA polymerase sigma-70 factor, family protein	K	INFORMATION STORAGE AND PROCESSING		-1.24	0.311	Down
MSMEG_2794	GntR family transcriptional regulator	K	INFORMATION STORAGE AND PROCESSING		-1.69	0.340	Down
MSMEG_2279	3-hydroxyacyl-CoA dehydrogenase	R	POORLY CHARACTERIZED		-1.25	0.345	Down
lysS	lysyl-tRNA synthetase	J	INFORMATION STORAGE AND PROCESSING		-1.06	0.376	Down
rplC	50S ribosomal protein L3	J	INFORMATION STORAGE AND PROCESSING	Rv0701	-1.27	0.389	Down
rpsI	30S ribosomal protein S9	J	INFORMATION STORAGE AND PROCESSING	Rv3442c	-1.15	0.397	Down
MSMEG_1148	mce related protein	Q	METABOLISM		-1.33	0.401	Down
MSMEG_1501	methyltransferase, , family protein	Q	METABOLISM	RV2751	-1.27	0.419	Down
MSMEG_4737	hypothetical protein	S	POORLY CHARACTERIZED		-1.20	0.426	Down
rnpA	ribonuclease P protein component	J	INFORMATION STORAGE AND PROCESSING	Rv3923c	-1.34	0.429	Down
MSMEG_3306	zinc-binding alcohol dehydrogenase	R	POORLY CHARACTERIZED	Rv1912c	-1.18	0.432	Down
cobS	cobalamin synthase	H	METABOLISM	Rv2208	-1.34	0.432	Down
pdxH	pyridoxamine 5'-phosphate oxidase	H	METABOLISM	Rv2607	-1.26	0.443	Down
MSMEG_6396	antigen 85-C	R	POORLY CHARACTERIZED		-1.17	0.456	Down
MSMEG_4987	glycosyl transferase family protein	M	CELLULAR PROCESSES AND SIGNALING		-1.65	0.459	Down
MSMEG_0970	phosphoglycerate mutase	G	METABOLISM	RV0525	-1.29	0.464	Down
MSMEG_1144	virulence factor Mce family protein	Q	METABOLISM		-1.03	0.467	Down
MSMEG_5120	hypothetical protein	Q	METABOLISM	RV1186C	-1.04	0.471	Down
rplT	50S ribosomal protein L20	J	INFORMATION STORAGE AND PROCESSING	Rv1643	-1.10	0.473	Down
MSMEG_1230	hypothetical protein	S	POORLY CHARACTERIZED	RV2273	-1.10	0.473	Down
MSMEG_1595	oxidoreductase	R	POORLY CHARACTERIZED		-1.06	0.475	Down
MSMEG_5536	chorismate mutase	E	METABOLISM	RV0948C	-1.13	0.483	Down
rplP	50S ribosomal protein L16	J	INFORMATION STORAGE AND PROCESSING	Rv0708	-1.57	0.485	Down
infA	translation initiation factor IF-1	J	INFORMATION STORAGE AND PROCESSING	Rv3462c	-1.01	0.487	Down
MSMEG_5882	carbon monoxide dehydrogenase	C	METABOLISM		-1.13	0.489	Down
MSMEG_5733	universal stress protein family protein	T	CELLULAR PROCESSES AND SIGNALING		-1.80	0.504	Down
MSMEG_6182	hypothetical protein	S	POORLY CHARACTERIZED	RV3669	-1.09	0.512	Down
MSMEG_6422	ferritin family protein	P	METABOLISM	Rv3841	-1.74	0.514	Down
MSMEG_1818	membrane flanked domain-containing protein	S	POORLY CHARACTERIZED	RV3278C	-1.39	0.516	Down
MSMEG_5897	virulence factor mce family protein	Q	METABOLISM	Rv3496c	-1.14	0.516	Down
MSMEG_1640	ATP/GTP-binding protein	R	POORLY CHARACTERIZED	RV3362C	-1.11	0.519	Down
MSMEG_1679	AmiB protein	R	POORLY CHARACTERIZED		-1.46	0.527	Down
rpmJ	50S ribosomal protein L36	J	INFORMATION STORAGE AND PROCESSING	Rv3461c	-1.36	0.531	Down
MSMEG_6223	TetR family transcriptional regulator	K	INFORMATION STORAGE AND PROCESSING		-1.19	0.538	Down
MSMEG_5245	universal stress protein family protein	T	CELLULAR PROCESSES AND SIGNALING	RV2028C	-1.06	0.545	Down
lipA	lipoyl synthase	H	METABOLISM	Rv2218	-1.10	0.548	Down
MSMEG_1942	integral membrane protein	S	POORLY CHARACTERIZED		-1.04	0.548	Down

MSMEG_1680	hypothetical protein	S	POORLY CHARACTERIZED		-1.09	0.556	Down
rpmA	50S ribosomal protein L27	J	INFORMATION STORAGE AND PROCESSING	Rv2441c	-1.04	0.563	Down
rpmC	50S ribosomal protein L29	J	INFORMATION STORAGE AND PROCESSING	Rv0709	-1.64	0.563	Down
rpsG	30S ribosomal protein S7	J	INFORMATION STORAGE AND PROCESSING	Rv0683	-1.37	0.564	Down
MSMEG_5243	hypothetical protein	R	POORLY CHARACTERIZED		-2.72	0.571	Down
MSMEG_3950	universal stress protein family protein	T	CELLULAR PROCESSES AND SIGNALING	RV2026C	-2.38	0.576	Down
rho	transcription termination factor Rho	K	INFORMATION STORAGE AND PROCESSING	Rv1297	-1.27	0.586	Down
MSMEG_5469	GNAT family acetyltransferase	J	INFORMATION STORAGE AND PROCESSING	Rv0995	-1.05	0.611	Down
MSMEG_2728	glutamate transport ATP-binding protein GluA	E	METABOLISM		-1.45	0.612	Down
MSMEG_3280	polyamine-binding lipoprotein	E	METABOLISM		-1.10	0.617	Down
MSMEG_0639	oligopeptide transport ATP-binding protein AppF	E	METABOLISM		-1.25	0.633	Down
aspA	aspartate ammonia-lyase	E	METABOLISM		-1.19	0.653	Down
MSMEG_1681	endoribonuclease L-PSP superfamily protein	J	INFORMATION STORAGE AND PROCESSING		-1.22	0.654	Down
MSMEG_4568	ribonuclease Z	R	POORLY CHARACTERIZED		-1.06	0.678	Down
MSMEG_5244	DevR family transcriptional regulator	K	INFORMATION STORAGE AND PROCESSING		-1.76	0.683	Down
MSMEG_5136	hypothetical protein	R	POORLY CHARACTERIZED	RV0080	-1.07	0.707	Down

Table S9: List of Genes that are Up/Down regulated in LIX79 (WT PknK Overexpression Strain) but not in LIX80 (K55M Mutant PknK Overexpression Strain) at 96 hr post induction with Acetamide

GeoMean-Fold Values of 2 Replicates is provided

Cut off used to filter up and down regulated genes

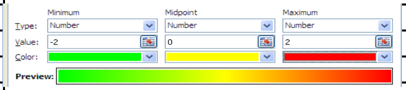

Upregulated

For filtering upregulation we consider Geomean fold >=1 in treated sample.

Downregulated

For filtering downregulation we consider Geomean fold <=-1 in treated sample.

Expression fold values are provided in terms of log base 2

GeneName	COG_Code	RV Number	Geofold Mean LIX79/70	Geofold Mean LIX80/70	Product
MSMEG_2698	Q	RV2740	3.23	-0.77	hypothetical protein
MSMEG_4685	R		2.97	-0.17	oxidoreductase
MSMEG_2469	Q		2.92	-0.04	AMP-dependent synthetase/ligase
MSMEG_2416	D	RV2927C	2.91	0.76	hypothetical protein
selB	J		2.79	-0.18	selenocysteine-specific translation elongation factor
MSMEG_6585	I		2.76	0.42	acyl-CoA dehydrogenase
MSMEG_3241	S		2.75	0.19	hypothetical protein
MSMEG_3580	R	Rv0129c	2.63	-0.03	antigen 85-C
MSMEG_0996	S	RV0540	2.60	-0.43	hypothetical protein
MSMEG_2120	K		2.48	-0.47	hypothetical protein
MSMEG_2421	O	RV2923C	2.45	-0.12	hypothetical protein
MSMEG_6096	K		2.45	-0.06	pantothenate kinase
MSMEG_0617	D		2.40	-0.29	ftsk/SpoIIIE family protein
MSMEG_2488	C		2.40	-0.23	[NADP+] succinate-semialdehyde dehydrogenase
MSMEG_0985	G	Rv3476c	2.39	-0.36	sugar transporter family protein
MSMEG_6275	L	Rv3711c	2.39	-0.24	DNA polymerase III subunit epsilon
MSMEG_3173	J	Rv1538c	2.39	0.33	L-asparaginase
MSMEG_1854	O		2.39	-0.02	valosin containing protein-1
MSMEG_4470	R	RV2326C	2.39	-0.87	ABC transporter
MSMEG_1378	G		2.34	0.06	1-phosphofructokinase
MSMEG_6583	R		2.31	-0.39	antigen 85-C
MSMEG_4579	G	RV2419C	2.30	-0.80	phosphoglycerate mutase
MSMEG_3852	P		2.30	-0.09	aliphatic sulfonate binding protein
MSMEG_3191	J	RV1571	2.30	-0.87	hypothetical protein
MSMEG_0970	G	RV0525	2.28	-1.29	phosphoglycerate mutase
eutC	E		2.28	0.01	ethanolamine ammonia-lyase small subunit
MSMEG_0231	S	RV1898	2.23	-0.01	hypothetical protein
MSMEG_0993	T		2.20	-0.10	histidine kinase
phhB	H		2.19	0.06	pterin-4-alpha-carbinolamine dehydratase
clpS	S		2.19	0.18	ATP-dependent Clp protease adaptor protein ClpS
MSMEG_3505	J		2.19	-0.40	6-aminohexanoate-cyclic-dimer hydrolase
MSMEG_6931	K	Rv3911	2.17	-0.41	RNA polymerase sigma-70 factor
lexA	T	Rv2720	2.16	-0.33	LexA repressor

**MSMEG_5019**	K		2.16	0.88	regulatory protein					
**MSMEG_2489**	K		2.16	0.14	GntR family transcriptional regulator					
**hrpA**	L		2.16	-0.58	ATP-dependent helicase HrpA					
**MSMEG_3668**	I		2.15	-0.34	acyl-CoA dehydrogenase					
**MSMEG_4477**	R	Rv1900c	2.14	0.23	hydrolase, alpha/beta hydrolase fold family protein					
**MSMEG_5673**	K		2.13	-0.17	transcriptional regulator					
**MSMEG_3885**	L	Rv2092c	2.13	0.27	DEAD/DEAH box helicase					
**MSMEG_4519**	I		2.13	0.66	NrtC protein					
**MSMEG_3116**	G		2.11	0.07	inositol-1-monophosphatase					
**glnD**	O	Rv2918c	2.10	-0.13	PII uridylyl-transferase					
**MSMEG_2731**	D	RV2731	2.08	-0.12	DNA repair ATPase					
**MSMEG_0968**	Q		2.08	-0.22	cytochrome P450					
**MSMEG_5706**	L	Rv0861c	2.07	-0.35	DNA or RNA helicase of superfamily protein II					
**thrC**	E	Rv1295	2.07	-0.91	threonine synthase					
**gabD2**	C	Rv1731	2.06	-0.11	succinate-semialdehyde dehydrogenase					
**MSMEG_6392**	Q	Rv3800c	2.06	-0.47	polyketide synthase					
**xylB**	G		2.06	0.52	xylulokinase					
**MSMEG_1932**	S	RV3209	2.05	-0.51	MmpS3 protein					
**MSMEG_4253**	M	Rv0557	2.05	0.47	glycoside hydrolase family protein					
**MSMEG_2033**	R	Rv3141	2.04	0.48	oxidoreductase, zinc-binding dehydrogenase					
**trpD**	E	Rv2192c	2.04	-0.03	anthranilate phosphoribosyltransferase					
**MSMEG_6582**	C		2.04	-0.53	pyridine nucleotide-disulfide oxidoreductase domain-containing protein					
**MSMEG_2632**	H		2.02	0.68	SAM-dependent methyltransferase					
**MSMEG_5074**	K	RV1219C	2.02	0.48	transcriptional regulatory protein					
**MSMEG_4524**	H	Rv2386c	2.02	0.22	salicylate synthase MbtI					
**MSMEG_3119**	V	RV1458C	2.02	-0.14	ABC transporter ATP-binding protein					
**MSMEG_0929**	S	RV0483	2.01	0.44	ErfK/YbiS/YcfS/YnhG family protein					
**MSMEG_6926**	J		2.01	0.46	tRNA adenylyltransferase					
**MSMEG_2106**	K		1.99	-0.17	transcriptional regulator					
**mnmA**	J		1.98	0.74	tRNA-specific 2-thiouridylase MnmA					
**MSMEG_1646**	J		1.98	-0.01	ribosomal RNA adenine dimethylase					
**MSMEG_3029**	U	RV2551C	1.97	-0.12	peptidase, A24 (type IV prepilin peptidase) family protein					
**MSMEG_3290**	R		1.97	-0.62	regulatory protein					
**MSMEG_1292**	C		1.97	-0.05	FAD binding domain-containing protein					
**MSMEG_0763**	R		1.96	-0.14	antibiotic transporter					
**murD**	M	Rv2155c	1.96	-0.28	UDP-N-acetylmuramoyl-L-alanyl-D-glutamate synthetase					
**MSMEG_4469**	P	RV2325C	1.94	0.07	cobalt transporter					
**MSMEG_2405**	E		1.94	0.38	MarR family transcriptional regulator					
**MSMEG_4305**	G	RV2228C	1.94	0.28	bifunctional RNase H/acid phosphatase					
**MSMEG_6221**	S	RV3694C	1.94	-0.11	integral membrane protein					
**MSMEG_3786**	E		1.93	0.42	D-amino acid deaminase					
**MSMEG_1945**	P	RV3200C	1.92	-0.05	ion channel membrane protein					
**MSMEG_3062**	S		1.91	-0.58	hypothetical protein					
**MSMEG_6174**	K		1.91	0.21	IclR family transcriptional regulator					
**MSMEG_4576**	O		1.90	-0.88	SpfH domain-containing protein					
**MSMEG_2597**	C	Rv2858c	1.90	-0.48	aldehyde dehydrogenase					
**xerC**	L	Rv2894c	1.90	-0.21	site-specific tyrosine recombinase XerC					
**MSMEG_1689**	R		1.90	-0.35	3-oxoacyl-ACP reductase					
**MSMEG_3607**	R		1.89	0.67	short chain dehydrogenase					

**dut**	F	Rv2697c	1.89	-0.35	deoxyuridine 5'-triphosphate nucleotidohydrolase					
**MSMEG_4299**	I		1.89	-0.23	enoyl-CoA hydratase/isomerase					
**MSMEG_3203**	E		1.88	-0.33	transporter LysE family protein					
**MSMEG_4659**	K		1.87	-0.45	GntR family transcriptional regulator					
**MSMEG_2353**	G		1.87	-0.03	hypothetical protein					
**MSMEG_6171**	D	RV3660C	1.86	0.20	hypothetical protein					
**MSMEG_2402**	R	RV2974C	1.86	0.50	dihydroxyacetone kinase					
**MSMEG_0984**	R		1.86	-0.38	trap transporter 4tm/12tm fusion protein					
**MSMEG_2426**	E	Rv2919c	1.85	0.49	nitrogen regulatory protein P-II					
**MSMEG_3570**	S		1.84	-0.02	hypothetical protein					
**cobT**	H	Rv2207	1.84	-0.17	nicotinate-nucleotide--dimethylbenzimidazole phosphoribosyltransferase					
**MSMEG_0064**	N	Rv3873	1.83	0.11	PPE family protein					
**MSMEG_5470**	H	Rv0994	1.82	-0.60	molybdopterin biosynthesis protein MoeA 1					
**crcB**	D		1.82	0.74	camphor resistance protein CrcB					
**MSMEG_1760**	R	RV0851C	1.82	0.34	short-chain dehydrogenase/reductase SDR					
**MSMEG_6230**	I		1.81	-0.69	acyltransferase					
**MSMEG_1829**	S		1.80	0.32	F420-0--gamma-glutamyl ligase					
**MSMEG_2788**	R	RV2670C	1.80	-0.09	ATP/GTP-binding integral membrane protein					
**MSMEG_0100**	T	Rv0153c	1.80	0.14	phosphotyrosine protein phosphatase ptpb					
**hemL**	H	Rv0524	1.80	-0.17	glutamate-1-semialdehyde aminotransferase					
**MSMEG_1624**	T		1.80	0.56	universal stress protein family protein					
**MSMEG_5693**	G	RV0876C	1.80	0.39	transporter major facilitator family protein					
**MSMEG_5059**	G	Rv1237	1.79	0.00	ABC transporter permease					
**ruvC**	L	Rv2594c	1.79	0.66	Holliday junction resolvase					
**MSMEG_6437**	P		1.79	-0.29	copper resistance protein D					
**MSMEG_5644**	S	RV0180C	1.79	0.13	membrane protein					
**MSMEG_0216**	R		1.79	0.77	3-hydroxyacyl-CoA dehydrogenase					
**MSMEG_0991**	G	Rv0536	1.79	0.65	NAD-dependent epimerase/dehydratase					
**MSMEG_1581**	Q		1.78	0.19	hypothetical protein					
**corA**	P	Rv1239c	1.78	-0.82	magnesium and cobalt transporter CorA					
**MSMEG_6398**	R	Rv1886c	1.78	0.49	antigen 85-A					
**MSMEG_6072**	P	Rv3578	1.78	0.65	citrate transporter					
**MSMEG_1894**	R	RV3224	1.77	0.13	short chain dehydrogenase					
**MSMEG_4873**	I		1.77	-0.64	enoyl-CoA hydratase/isomerase					
**MSMEG_3007**	C		1.76	-0.12	succinate-semialdehyde dehydrogenase					
**MSMEG_1573**	G	RV3433C	1.76	0.70	carbohydrate kinase					
**MSMEG_0087**	G		1.76	-0.49	glucitol operon repressor					
**MSMEG_5589**	P	Rv0924c	1.75	0.66	manganese transporter MntH					
**folE**	H	Rv3609c	1.75	0.27	GTP cyclohydrolase I					
**MSMEG_0627**	M		1.74	-0.69	glycosyl transferase family protein					
**MSMEG_6225**	G		1.74	-0.28	proton antiporter efflux pump					
**MSMEG_3127**	R	RV1466	1.74	0.90	hypothetical protein					
**thiE**	H	Rv0414c	1.74	-0.02	thiamine-phosphate pyrophosphorylase					
**MSMEG_0435**	E	RV0263C	1.74	0.90	allophanate hydrolase subunit 2					
**MSMEG_1374**	G		1.74	-0.63	ribose ABC transporter periplasmic binding protein					
**malQ**	G	Rv1781c	1.73	-0.82	4-alpha-glucanotransferase					
**MSMEG_2493**	E		1.73	-0.22	aminotransferase, class I and II family protein					
**MSMEG_5075**	V	RV1218C	1.73	-0.16	ABC transporter ATP-binding protein					
**lgt**	M		1.73	-0.27	prolipoprotein diacylglyceryl transferase					

**MSMEG_2157**	S		1.73	0.87	hypothetical protein				
**MSMEG_4279**	T	RV2212	1.72	-0.34	adenylate and guanylate cyclase catalytic domain-containing protein				
**MSMEG_1940**	I	Rv3203	1.71	0.35	alpha/beta hydrolase				
**MSMEG_4231**	M	Rv2157c	1.71	-0.09	UDP-N-acetylmuramoyl-tripeptide--D-alanyl-D-alanine ligase				
**MSMEG_5533**	C		1.71	-0.13	4Fe-4S ferredoxin				
**cbiQ**	P		1.71	0.51	cobalt ABC transporter permease				
**MSMEG_4225**	M	Rv2151c	1.71	0.17	cell division protein FtsQ				
**MSMEG_1762**	C	Rv3293	1.71	-0.10	piperideine-6-carboxylic acid dehydrogenase				
**MSMEG_3879**	R	RV2073C	1.71	-0.06	short chain dehydrogenase				
**rimI**	R	Rv3420c	1.71	0.98	ribosomal-protein-alanine acetyltransferase				
**MSMEG_0667**	I		1.71	-0.20	butyryl-CoA dehydrogenase				
**MSMEG_4908**	Q	RV1333	1.70	0.45	endo-type 6-aminohexanoate oligomer hydrolase				
**cobD**	H	Rv2236c	1.70	-0.30	cobalamin biosynthesis protein				
**MSMEG_5547**	S		1.70	0.01	hypothetical protein				
**MSMEG_3616**	S	RV1861	1.70	0.39	integral membrane protein				
**MSMEG_3213**	V	RV3263	1.70	-0.65	hypothetical protein				
**MSMEG_6914**	G		1.69	0.08	hypothetical protein				
**MSMEG_2357**	E	Rv3025c	1.69	-0.60	cysteine desulfurase				
**MSMEG_2942**	R		1.69	0.87	glyoxalase/bleomycin resistance protein/dioxygenase superfamily protein				
**MSMEG_1684**	R	RV3311	1.69	-0.21	hypothetical protein				
**MSMEG_2122**	G		1.69	0.34	dihydroxyacetone kinase subunit DhaL				
**MSMEG_4912**	L	Rv1329c	1.68	-0.09	helicase				
**MSMEG_5234**	R		1.68	-0.37	short-chain dehydrogenase/reductase SDR				
**psd**	I	Rv0437c	1.68	-0.14	phosphatidylserine decarboxylase				
**MSMEG_2940**	S	RV2603C	1.68	-0.04	hypothetical protein				
**MSMEG_4719**	E		1.68	-0.74	peptidase S9, prolyl oligopeptidase				
**MSMEG_0321**	R		1.68	-0.72	transcriptional regulator				
**MSMEG_1432**	I		1.68	0.27	acetylcholinesterase				
**MSMEG_4233**	M	Rv2163c	1.68	0.42	penicillin binding protein transpeptidase domain-containing protein				
**MSMEG_3534**	R		1.68	0.69	4-hydroxybenzoyl-CoA thioesterase				
**MSMEG_6408**	I	RV3814C	1.67	0.18	acyltransferase				
**MSMEG_5064**	G		1.67	0.56	malyl-CoA lyase				
**nuoD**	C	Rv3148	1.67	0.81	NADH dehydrogenase subunit D				
**MSMEG_3618**	I	Rv1860	1.67	0.48	hypothetical protein				
**MSMEG_5851**	R	RV0774C	1.66	-1.10	esterase				
**MSMEG_3239**	T		1.66	0.15	two-component system sensor kinase				
**MSMEG_6320**	Q		1.66	0.26	diol dehydrase subunit gamma				
**MSMEG_0592**	K		1.66	-0.01	rhamnose catabolism operon transcriptional regulator				
**MSMEG_5883**	C		1.66	0.00	2Fe-2S-binding domain-containing protein				
**murG**	M	Rv2153c	1.66	-0.09	UDPdiphospho-muramoylpentapeptide beta-N- acetylglucosaminyltransferase				
**MSMEG_6128**	K		1.66	-0.79	two-component system response regulator				
**MSMEG_0634**	I	RV0308	1.66	-0.84	PAP2 superfamily protein				
**bioB**	H	Rv1589	1.66	0.12	biotin synthase				
**priA**	L	Rv1402	1.65	-0.42	primosome assembly protein PriA				
**smc**	D	Rv2922c	1.65	0.01	chromosome segregation protein SMC				
**MSMEG_1941**	L	RV3202C	1.65	0.03	helicase, UvrD/Rep family protein				
**MSMEG_4304**	K		1.64	-0.31	regulatory protein				
**MSMEG_2361**	S		1.64	0.10	phosphoribosylglycinamide formyltransferase				
**rfbD**	M	Rv3266c	1.64	-0.44	dTDP-4-dehydrorhamnose reductase				

**MSMEG_2584**	M	RV2864C	1.64	0.34	penicillin-binding protein				
**MSMEG_2804**	T		1.63	0.17	two-component system sensor kinase				
**ggt**	E	Rv0773c	1.63	0.17	gamma-glutamyltranspeptidase				
**MSMEG_5163**	E		1.63	-0.36	glycine/betaine ABC transporter periplasmic protein				
**MSMEG_0863**	R	RV0439C	1.63	-0.06	short chain dehydrogenase				
**MSMEG_1899**	I	Rv2503c	1.63	0.20	succinyl-CoA:3-ketoacid-coenzyme A transferase subunit B				
**MSMEG_3255**	S		1.62	0.29	DoxX subfamily protein				
**MSMEG_4449**	K		1.62	0.11	transcriptional regulator				
**MSMEG_0999**	S	RV0541C	1.62	0.17	integral membrane protein				
**MSMEG_2422**	C	Rv2922A	1.61	0.17	acylphosphatase				
**MSMEG_3843**	R	RV1628C	1.61	-1.08	hypothetical protein				
**MSMEG_0810**	F		1.61	0.32	pyrimidine permease RutG				
**MSMEG_5193**	K		1.61	0.30	AraC family transcriptional regulator				
**MSMEG_5430**	R		1.61	0.26	retinol dehydrogenase 13				
**MSMEG_4220**	R	RV2148C	1.60	-0.27	hypothetical protein				
**cobM**	H	Rv2071c	1.60	-0.51	precorrin-4 C(11)-methyltransferase				
**MSMEG_0971**	C	RV0526	1.60	0.06	hypothetical protein				
**MSMEG_1533**	O	Rv3449	1.60	0.02	subtilase				
**MSMEG_1954**	R	RV3197	1.60	-0.43	ABC transporter				
**secD**	U	Rv2587c	1.59	0.39	preprotein translocase subunit SecD				
**MSMEG_5640**	G		1.59	0.67	amylo-alpha-1,6-glucosidase				
**MSMEG_1902**	K		1.59	-0.20	transcriptional regulator				
**MSMEG_1199**	S	RV0585C	1.59	0.67	hypothetical protein				
**MSMEG_4256**	M	RV2190C	1.59	-0.16	NLP/P60 family protein				
**MSMEG_2787**	H	Rv2671	1.59	-0.22	hypothetical protein				
**gatA**	J	Rv3011c	1.59	0.27	glutamyl-tRNA(Gln)/aspartyl-tRNA(Asn) amidotransferase subunit alpha				
**MSMEG_0318**	Q		1.58	0.45	AMP-dependent synthetase/ligase				
**MSMEG_3870**	Q		1.58	0.20	alpha-ketoglutarate-dependent taurine dioxygenase				
**MSMEG_1572**	E		1.58	0.70	GntR family transcriptional regulator				
**MSMEG_3902**	O		1.58	0.86	ATPase AAA				
**MSMEG_4486**	K	RV2358	1.57	-0.42	ArsR family transcriptional regulator				
**MSMEG_2490**	Q		1.57	0.10	decarboxylase				
**MSMEG_3571**	Q		1.57	0.16	thioesterase				
**MSMEG_3059**	I	Rv1400c	1.57	-0.54	esterase				
**MSMEG_6318**	D		1.56	0.00	diol dehydratase reactivation protein				
**MSMEG_5235**	R		1.56	0.24	short chain dehydrogenase				
**MSMEG_1891**	S	RV3226C	1.56	0.86	hypothetical protein				
**serB**	E	Rv3042c	1.56	-0.42	phosphoserine phosphatase				
**MSMEG_5681**	R	Rv0886	1.56	0.36	ferredoxin/ferredoxin--NADP reductase				
**MSMEG_5153**	I	RV1157C	1.56	0.32	hypothetical protein				
**MSMEG_0066**	S	Rv3875	1.56	-0.85	early secretory antigenic target, 6 kDa				
**MSMEG_2642**	S		1.55	0.37	hypothetical protein				
**MSMEG_0580**	I		1.55	0.55	hypothetical protein				
**MSMEG_3193**	K		1.55	-0.08	TetR family transcriptional regulator				
**MSMEG_3860**	M	Rv2051c	1.55	0.12	polyprenol-monophosphomannose synthase Ppm1				
**MSMEG_6191**	J	RV3678C	1.55	0.74	translation initiation inhibitor				
**MSMEG_3622**	O	Rv1853	1.54	0.81	hypothetical protein				
**MSMEG_2116**	G		1.54	-0.64	PTS system, glucose-specific IIBC component				
**MSMEG_2159**	S		1.54	0.77	hypothetical protein				

**MSMEG_6231**	H	RV1978	1.53	-0.25	hypothetical protein				
**MSMEG_5908**	Q	Rv3506	1.53	0.41	acyl-CoA synthetase				
**otsB**	G		1.53	-0.28	trehalose-phosphatase				
**MSMEG_6173**	E	RV3661	1.53	0.83	morphological differentiation-associated protein				
**MSMEG_3677**	L	Rv1746	1.53	-0.05	serine/threonine protein kinase				
**MSMEG_4896**	Q		1.53	-0.03	fatty-acid--CoA ligase				
**MSMEG_5251**	P		1.53	0.41	hypothetical protein				
**MSMEG_6662**	R		1.52	0.46	short chain dehydrogenase				
**MSMEG_5413**	P	RV1026	1.52	0.30	exopolyphosphatase				
**MSMEG_4925**	L		1.52	0.19	transcriptional regulator, Ada family protein/DNA-3-methyladenine glycosylase II				
**dacB**	M	RV3627C	1.52	0.18	D-alanyl-D-alanine carboxypeptidase/D-alanyl-D-alanine-endopeptidase				
**MSMEG_4898**	K	RV1343C	1.52	0.25	hypothetical protein				
**trpB**	E	Rv1612	1.51	-0.37	tryptophan synthase subunit beta				
**MSMEG_0367**	J		1.51	0.03	O-demethylpuromycin-O-methyltransferase				
**MSMEG_5073**	R	RV1220C	1.51	-0.55	O-methyltransferase, family protein 3				
**MSMEG_1692**	K		1.51	-0.69	ECF-family protein RNA polymerase sigma factor				
**alr**	M	Rv3423c	1.51	-0.20	alanine racemase				
**thiO**	E	Rv0415	1.51	-0.08	glycine oxidase ThiO				
**MSMEG_5970**	H		1.51	-0.26	glutamate-1-semialdehyde 2,1-aminomutase				
**dnaE**	L		1.51	0.43	DNA polymerase III subunit alpha				
**MSMEG_0809**	S	RV1139C	1.50	-0.72	isoprenylcysteine carboxyl methyltransferase				
**MSMEG_5884**	I		1.50	-0.09	3-hydroxyisobutyrate dehydrogenase				
**MSMEG_0877**	M		1.50	-0.51	dihydrodipicolinate synthase				
**MSMEG_3081**	S	Rv1423	1.50	0.35	hypothetical protein				
**MSMEG_3172**	L		1.50	-0.08	DNA polymerase IV				
**MSMEG_5220**	I		1.50	0.39	esterase/lipase/thioesterase				
**MSMEG_3581**	R		1.50	0.34	FabG protein				
**MSMEG_6125**	K		1.49	-0.01	transcriptional regulatory protein				
**MSMEG_3781**	S	RV1645C	1.49	0.46	hypothetical protein				
**MSMEG_3842**	R		1.49	0.46	esterase				
**MSMEG_5987**	K		1.49	0.11	two-component regulator				
**leuB**	E	Rv2995c	1.49	-0.37	3-isopropylmalate dehydrogenase				
**MSMEG_4662**	G		1.49	0.39	deoxyribose-phosphate aldolase superfamily protein				
**MSMEG_1536**	D		1.49	-0.29	ftsk/SpoIIIE family protein				
**MSMEG_5066**	S	RV1231C	1.49	0.17	integral membrane protein				
**MSMEG_5838**	K		1.49	0.79	TetR family transcriptional regulator				
**MSMEG_1382**	R		1.48	-0.33	MmpL5 protein				
**MSMEG_4653**	G		1.48	0.44	AP endonuclease, family protein 2 superfamily protein				
**MSMEG_2279**	R		1.48	-1.25	3-hydroxyacyl-CoA dehydrogenase				
**MSMEG_3783**	I	Rv2789c	1.48	0.04	acyl-CoA dehydrogenase				
**MSMEG_2875**	I		1.48	-0.13	esterase				
**MSMEG_4577**	S	RV2417C	1.48	0.61	DegV family protein				
**MSMEG_5006**	T	RV1276C	1.48	-0.36	phosphohistidine phosphatase				
**MSMEG_6900**	M	Rv0050	1.48	-0.08	penicillin-binding protein 1				
**MSMEG_4975**	R		1.48	0.01	flavin-nucleotide-binding protein				
**MSMEG_2762**	G	Rv2701c	1.48	-0.35	inositol-1-monophosphatase				
**MSMEG_6866**	P		1.48	0.18	peptide ABC transporter permease				
**MSMEG_5994**	I	Rv3544c	1.47	0.47	acyl-CoA dehydrogenase				
**cobH**	H		1.47	0.17	precorrin-8X methylmutase				

**MSMEG_0316**	G		1.47	0.28	NagC regulator				
**MSMEG_1210**	S		1.47	-0.38	hypothetical protein				
**MSMEG_0923**	S	RV0479C	1.47	-0.40	hypothetical protein				
**MSMEG_6519**	P		1.47	-0.09	pyridoxamine 5'-phosphate oxidase				
**MSMEG_3608**	I	RV1867	1.47	-0.38	acetyl-CoA acetyltransferase				
**MSMEG_4968**	T		1.47	0.59	two-component system sensor kinase				
**MSMEG_6421**	K	RV3840	1.47	-0.23	transcriptional regulator				
**MSMEG_1577**	R	RV3422C	1.47	0.11	hypothetical protein				
**MSMEG_2793**	T		1.46	0.63	sensor-type histidine kinase PrrB				
**MSMEG_2665**	O	RV2772C	1.46	0.23	hypothetical protein				
**MSMEG_1460**	R		1.46	0.16	pyridine nucleotide-disulfide oxidoreductase				
**MSMEG_1096**	O		1.46	-0.31	urease accessory protein UreD				
**MSMEG_5045**	C	RV0161	1.45	-0.13	D-2-hydroxyacid dehydrogenase				
**MSMEG_4962**	C	RV1751	1.45	0.71	hypothetical protein				
**rho**	K	Rv1297	1.45	-1.27	transcription termination factor Rho				
**MSMEG_3867**	R	RV3163C	1.45	-0.86	ATPase AAA				
**MSMEG_1681**	J		1.45	-1.22	endoribonuclease L-PSP superfamily protein				
**MSMEG_3303**	R		1.45	-0.25	carboxylic ester hydrolase				
**MSMEG_4517**	K		1.45	0.13	TetR family transcriptional regulator				
**MSMEG_3848**	I	Rv2045c	1.45	0.11	para-nitrobenzyl esterase				
**ung**	L	Rv2976c	1.45	0.02	uracil-DNA glycosylase				
**MSMEG_1371**	G		1.44	0.12	hypothetical protein				
**MSMEG_4572**	L	RV2413C	1.44	-0.24	hypothetical protein				
**MSMEG_0691**	K	RV0339C	1.44	-0.11	transcriptional regulatory protein				
**MSMEG_4971**	R	RV2781C	1.44	-0.17	oxidoreductase				
**argC**	E	Rv1652	1.44	0.63	N-acetyl-gamma-glutamyl-phosphate reductase				
**ruvA**	L	Rv2593c	1.43	-0.20	Holliday junction DNA helicase RuvA				
**MSMEG_2182**	K	RV0258C	1.43	-0.23	transcriptional regulator				
**ilvE**	H	Rv2210c	1.43	0.05	branched-chain amino acid aminotransferase				
**MSMEG_5848**	E		1.42	-0.03	amino acid permease				
**MSMEG_2470**	I		1.42	-0.39	acyl-CoA thioesterase				
**MSMEG_4463**	R	RV2314C	1.42	-1.25	CalR5 protein				
**MSMEG_3506**	E		1.42	0.05	amino acid decarboxylase				
**MSMEG_3252**	S	RV2639C	1.42	0.03	hypothetical protein				
**hisF**	E	Rv1605	1.42	0.18	imidazole glycerol phosphate synthase subunit HisF				
**MSMEG_1546**	Q		1.42	-0.33	coenzyme B12-dependent glycerol dehydrogenase small subunit				
**MSMEG_3146**	M	RV1478	1.42	0.56	invasin 1				
**MSMEG_3246**	T		1.42	0.60	response regulator				
**MSMEG_0232**	G	RV0191	1.42	-0.22	sugar transporter family protein				
**MSMEG_0276**	C		1.42	0.22	aldehyde-alcohol dehydrogenase				
**MSMEG_3630**	K		1.42	0.66	CopY family transcriptional regulator protein				
**MSMEG_2105**	L		1.41	0.04	ATP dependent DNA ligase				
**MSMEG_6533**	I		1.41	0.40	hypothetical protein				
**MSMEG_3480**	V		1.41	-0.18	streptomycin 6-kinase				
**MSMEG_6083**	L	Rv3589	1.41	0.86	base excision DNA repair protein, HhH-GPD family protein				
**rpe**	G	Rv1408	1.41	-0.13	ribulose-phosphate 3-epimerase				
**MSMEG_5619**	I		1.41	-0.12	acyl-CoA dehydrogenase				
**MSMEG_1700**	K		1.41	-0.33	TetR family transcriptional regulator				
**MSMEG_6099**	S	RV3604C	1.41	-0.67	hypothetical protein				

**MSMEG_5858**	R	RV0769	**1.41**	**-0.08**	short chain dehydrogenase					
**MSMEG_6220**	R	RV3693	**1.41**	**0.01**	lipoprotein					
**MSMEG_1366**	Q	Rv0655	**1.41**	**0.59**	ABC transporter ATP-binding protein					
**MSMEG_5237**	R	RV1101C	**1.41**	**-0.05**	hypothetical protein					
**MSMEG_3145**	M	RV1477	**1.40**	**0.21**	secreted cell wall-associated hydrolase					
**uppS**	I	RV1086	**1.40**	**-0.29**	UDP pyrophosphate synthase					
**MSMEG_0865**	S	RV1226C	**1.40**	**-0.27**	membrane flanked domain-containing protein					
**MSMEG_1379**	R		**1.40**	**0.11**	hypothetical protein					
**MSMEG_4894**	S		**1.40**	**-0.72**	hypothetical protein					
**MSMEG_5696**	K	Rv0871	**1.40**	**-0.24**	cold-shock DNA-binding domain-containing protein					
**ptsI**	G		**1.40**	**0.12**	phosphoenolpyruvate-protein phosphotransferase					
**MSMEG_3254**	S		**1.39**	**0.60**	RDD family protein					
**MSMEG_4984**	Q	RV1532C	**1.39**	**-1.19**	hypothetical protein					
**MSMEG_3644**	K	RV1830	**1.39**	**-0.66**	MerR family transcriptional regulator					
**MSMEG_2342**	C		**1.39**	**-0.55**	glycosyltransferase					
**MSMEG_5738**	O		**1.39**	**-0.62**	hypothetical protein					
**MSMEG_0662**	E		**1.39**	**0.95**	putrescine transport ATP-binding protein PotG					
**aroE**	E	Rv2552c	**1.39**	**0.39**	shikimate 5-dehydrogenase					
**MSMEG_2806**	T		**1.38**	**0.49**	two-component system response regulator					
**MSMEG_4348**	H	Rv3509c	**1.38**	**-0.51**	hypothetical protein					
**alaS**	J	Rv2555c	**1.38**	**-0.06**	alanyl-tRNA synthetase					
**MSMEG_2878**	I		**1.38**	**0.27**	acyl-CoA dehydrogenase					
**MSMEG_4334**	C	RV2250.1	**1.38**	**0.32**	flavoprotein					
**MSMEG_6416**	G	RV3837C	**1.38**	**0.26**	phosphoglycerate mutase					
**MSMEG_6168**	U	RV3658C	**1.38**	**0.78**	type II secretion system protein F					
**MSMEG_6044**	K	RV3575C	**1.38**	**0.07**	ligand-binding /sugar binding domain-containing protein					
**MSMEG_3884**	D	RV2091C	**1.37**	**0.19**	hypothetical protein					
**MSMEG_0973**	O	RV0528	**1.37**	**-0.75**	hypothetical protein					
**MSMEG_0105**	K	Rv0844c	**1.37**	**-0.30**	LuxR family transcriptional regulator					
**MSMEG_6219**	R	Rv3692	**1.37**	**-0.11**	ATPase AAA					
**MSMEG_4626**	J	Rv2444c	**1.37**	**0.25**	ribonuclease, Rne/Rng family protein					
**MSMEG_6473**	Q		**1.37**	**0.41**	tetracenomycin polyketide synthesis O-methyltransferase TcmP					
**MSMEG_4906**	H		**1.36**	**0.16**	ThiS family protein					
**MSMEG_6327**	J	RV3752C	**1.36**	**-0.13**	cytidine and deoxycytidylate deaminase					
**MSMEG_0621**	S		**1.36**	**0.00**	low molecular weight protein antigen 7					
**atzF**	J		**1.36**	**0.24**	allophanate hydrolase					
**MSMEG_1641**	S	RV3361C	**1.36**	**-0.53**	hypothetical protein					
**MSMEG_5853**	Q		**1.36**	**0.11**	cytochrome P450					
**MSMEG_5695**	O		**1.36**	**-0.10**	glutathione S-transferase					
**MSMEG_6483**	H		**1.36**	**-0.09**	methyltransferase type 11					
**MSMEG_0436**	E	RV0264C	**1.36**	**0.13**	allophanate hydrolase subunit 1					
**MSMEG_3822**	K		**1.35**	**0.51**	GntR family transcriptional regulator					
**MSMEG_3289**	R		**1.35**	**-0.07**	gp61 protein					
**MSMEG_3634**	F	RV1843C	**1.35**	**0.35**	inosine 5-monophosphate dehydrogenase					
**MSMEG_4467**	G	Rv2317	**1.35**	**0.58**	ABC transporter permease					
**MSMEG_1640**	R	RV3362C	**1.35**	**-1.11**	ATP/GTP-binding protein					
**MSMEG_5513**	L		**1.35**	**-0.54**	serine/threonine protein kinase					
**MSMEG_0680**	M	Rv0322	**1.35**	**-0.28**	UDP-glucose 6-dehydrogenase					
**MSMEG_3598**	G		**1.34**	**0.66**	periplasmic sugar-binding proteins					

**rph**	J		1.34	-0.26	ribonuclease PH				
**MSMEG_3542**	S		1.34	0.54	hypothetical protein				
**MSMEG_0930**	R	RV0484C	1.34	0.40	serine 3-dehydrogenase				
**panC**	H	Rv3602c	1.34	0.33	pantoate--beta-alanine ligase				
**MSMEG_2581**	R	RV2867C	1.34	0.21	acetyltransferase				
**MSMEG_6520**	F	Rv0382c	1.33	0.36	orotate phosphoribosyltransferase				
**MSMEG_0358**	F	RV0233	1.33	0.68	ribonucleotide-diphosphate reductase subunit beta				
**MSMEG_6864**	K		1.33	-0.74	transcriptional regulator				
**MSMEG_1001**	R		1.33	0.51	acetyltransferase, gnat family protein				
**MSMEG_1660**	R	RV0021C	1.33	0.32	oxidoreductase, 2-nitropropane dioxygenase				
**MSMEG_6584**	I		1.33	0.01	acyl-CoA dehydrogenase				
**MSMEG_6041**	I	Rv3573c	1.33	0.40	acyl-CoA dehydrogenase				
**MSMEG_1475**	C		1.32	-0.88	hypothetical protein				
**recX**	R	Rv2736c	1.32	-0.12	recombination regulator RecX				
**MSMEG_2798**	O		1.32	-0.10	hypothetical protein				
**carA**	F	Rv1383	1.32	-0.06	carbamoyl phosphate synthase small subunit				
**MSMEG_1901**	K		1.32	-0.55	DNA-binding HTH domain-containing protein				
**MSMEG_5678**	E		1.32	-0.15	glyoxalase/bleomycin resistance protein/dioxygenase				
**MSMEG_6095**	E		1.32	0.37	D-amino acid deaminase				
**MSMEG_3515**	R	Rv2002	1.32	0.08	3-alpha-(or 20-beta)-hydroxysteroid dehydrogenase				
**MSMEG_6105**	O	Rv3610c	1.31	0.90	cell division protein				
**greA**	K	Rv1080c	1.31	-0.09	transcription elongation factor GreA				
**MSMEG_1638**	R	RV3364C	1.31	-0.77	roadblock/LC7 domain-contain protein				
**MSMEG_3463**	Q	Rv3298c	1.31	-0.44	esterase				
**MSMEG_3465**	Q	Rv1925	1.31	0.21	fatty-acid--CoA ligase				
**MSMEG_4382**	R		1.31	0.29	dehydrogenase/reductase SDR family protein member 10				
**MSMEG_6294**	C		1.31	0.64	caib/baif family protein				
**MSMEG_0028**	L	Rv0014c	1.31	0.02	serine-threonine protein kinase				
**MSMEG_2188**	R		1.30	-0.27	integral membrane protein				
**lysA**	E	Rv1293	1.30	-0.24	diaminopimelate decarboxylase				
**MSMEG_4717**	I	Rv2502c	1.30	0.30	carboxyl transferase domain-containing protein				
**MSMEG_5988**	I		1.30	-0.31	hypothetical protein				
**mtrA**	K	Rv3246c	1.30	0.05	DNA-binding response regulator MtrA				
**MSMEG_4208**	S		1.30	-0.27	integral membrane protein				
**MSMEG_0224**	R	RV0187	1.30	-0.11	O-methyltransferase MdmC				
**MSMEG_6290**	Q		1.30	0.11	DNA-binding protein				
**edd**	G		1.30	0.10	phosphogluconate dehydratase				
**coaD**	H		1.29	-0.68	phosphopantetheine adenylyltransferase				
**MSMEG_0332**	R		1.29	0.06	2-nitropropane dioxygenase				
**MSMEG_0361**	G	Rv0237	1.29	-0.49	glycosyl hydrolase				
**hypE**	O		1.29	0.03	hydrogenase expression/formation protein HypE				
**MSMEG_3079**	R	RV1421	1.29	-0.32	hypothetical protein				
**MSMEG_0101**	S	RV1890C	1.28	-0.25	hypothetical protein				
**MSMEG_1046**	P	RV3041C	1.28	0.05	molybdenum ABC transporter ATPase				
**MSMEG_1643**	S		1.28	-0.20	hypothetical protein				
**MSMEG_6160**	R	RV3649	1.28	0.11	ATP-dependent rna helicase, dead/deah box family protein				
**MSMEG_2121**	G		1.28	-0.20	multiphosphoryl transfer protein (MTP)				
**MSMEG_6077**	K	RV3583C	1.28	0.16	CarD family transcriptional regulator				
**fbiC**	R		1.28	-0.03	FO synthase				

**MSMEG_0311**	M	RV0225	**1.27**	**-0.73**	glycosyltransferase				
**MSMEG_5591**	R	RV0923C	**1.27**	**0.47**	hypothetical protein				
**mdcB**	H		**1.27**	**0.24**	triphosphoribosyl-dephospho-CoA synthase				
**MSMEG_5674**	G		**1.27**	**0.64**	membrane transporter				
**MSMEG_1049**	H	RV3038C	**1.27**	**0.08**	UbiE/COQ5 family methyltransferase				
**pyrD**	F	Rv2139	**1.26**	**-0.47**	dihydroorotate dehydrogenase 2				
**bioA**	H	Rv1568	**1.26**	**0.30**	adenosylmethionine-8-amino-7-oxononanoate aminotransferase				
**nth**	L	Rv3674c	**1.26**	**-0.60**	endonuclease III				
**MSMEG_5404**	I		**1.26**	**0.34**	propionate--CoA ligase				
**MSMEG_4545**	E	Rv3666c	**1.26**	**0.30**	extracellular solute-binding protein				
**lysS**	J		**1.26**	**-1.06**	lysyl-tRNA synthetase				
**MSMEG_2111**	E	RV1885C	**1.26**	**-0.79**	chorismate mutase				
**MSMEG_6554**	V	RV1348	**1.26**	**-0.57**	ABC transporter ATP-binding protein				
**MSMEG_5025**	K		**1.26**	**-0.60**	transcriptional regulator				
**MSMEG_6069**	R	RV0106	**1.26**	**0.41**	CobW/P47K domain-containing protein				
**MSMEG_5910**	S		**1.26**	**0.02**	quinone binding protein				
**etfA**	C		**1.26**	**0.57**	electron transfer flavoprotein subunit alpha				
**MSMEG_3471**	H		**1.26**	**0.33**	hypothetical protein				
**moaC**	H	Rv0864	**1.26**	**-0.23**	molybdenum cofactor biosynthesis protein MoaC				
**MSMEG_4328**	Q	Rv2246	**1.25**	**-0.26**	3-oxoacyl-ACP synthase				
**MSMEG_6190**	R	RV3677C	**1.25**	**0.10**	metallo-beta-lactamase				
**MSMEG_5258**	R		**1.25**	**-0.16**	steroid delta-isomerase				
**MSMEG_0997**	R		**1.25**	**-0.02**	hypothetical protein				
**MSMEG_2181**	D	Rv3641c	**1.25**	**-0.03**	cell filamentation protein				
**MSMEG_4174**	K		**1.25**	**0.78**	IclR family transcriptional regulator				
**MSMEG_6091**	O		**1.25**	**0.69**	negative regulator of genetic competence ClpC/mecB				
**ftsZ**	D	Rv2150c	**1.25**	**0.05**	cell division protein FtsZ				
**MSMEG_4644**	H	Rv2453c	**1.24**	**0.01**	molybdopterin-guanine dinucleotide biosynthesis protein A				
**MSMEG_1540**	J		**1.24**	**-0.07**	ATP-dependent RNA helicase				
**MSMEG_5466**	R		**1.24**	**-0.26**	oxidoreductase				
**MSMEG_1563**	R		**1.24**	**-0.36**	short-chain dehydrogenase/reductase SDR				
**MSMEG_3693**	Q		**1.24**	**-0.51**	cytochrome P450 monooxygenase				
**cobA**	H	Rv2847c	**1.24**	**0.02**	uroporphyrin-III C-methyltransferase				
**MSMEG_0067**	D		**1.24**	**-0.05**	hypothetical protein				
**MSMEG_5004**	L	RV1277	**1.24**	**0.10**	DNA repair exonuclease				
**rimM**	J	Rv2907c	**1.24**	**-0.07**	16S rRNA-processing protein RimM				
**MSMEG_6688**	Q		**1.24**	**-0.14**	regulatory protein				
**ureB**	E	Rv1849	**1.24**	**-0.49**	urease subunit beta				
**MSMEG_3477**	M	RV1566C	**1.24**	**-0.20**	inv protein				
**MSMEG_3108**	G		**1.24**	**0.05**	ABC transporter ATPase				
**MSMEG_3873**	H		**1.24**	**-0.09**	cobalamin biosynthesis protein cobIJ				
**MSMEG_2648**	Q	RV2794C	**1.24**	**0.21**	Sfp-type phosphopantetheinyl transferase				
**MSMEG_3027**	R	RV2553C	**1.24**	**0.01**	hypothetical protein				
**MSMEG_3057**	P		**1.23**	**-0.06**	metal ABC transporter permease				
**MSMEG_0871**	C		**1.23**	**0.14**	aldehyde or xanthine dehydrogenase, molybdopterin binding subunit protein				
**engA**	R	Rv1713	**1.23**	**-0.59**	GTP-binding protein EngA				
**murQ**	R		**1.23**	**-0.34**	N-acetylmuramic acid 6-phosphate etherase				
**dxs**	I		**1.23**	**0.13**	1-deoxy-D-xylulose-5-phosphate synthase				
**MSMEG_6133**	M		**1.22**	**0.16**	5-dehydro-4-deoxyglucarate dehydratase				

**MSMEG_5684**	E	Rv0884c	**1.22**	**-0.30**	phosphoserine aminotransferase				
**MSMEG_5863**	Q	Rv0764c	**1.22**	**0.26**	cytochrome P450				
**MSMEG_2699**	C	RV2739C	**1.21**	**0.09**	hypothetical protein				
**MSMEG_6195**	P	RV3680	**1.21**	**-1.09**	ion-transporting ATPase				
**MSMEG_3780**	T	RV1647	**1.21**	**0.21**	adenylate and guanylate cyclase catalytic domain-containing protein				
**recO**	L	Rv2362c	**1.21**	**0.29**	DNA repair protein RecO				
**MSMEG_5204**	R		**1.21**	**0.58**	oxidoreductase, short chain dehydrogenase/reductase				
**MSMEG_2453**	K		**1.21**	**-0.06**	GntR family transcriptional regulator				
**MSMEG_1070**	R		**1.21**	**-0.33**	3-oxoacyl-ACP reductase				
**purN**	F	Rv0956	**1.21**	**0.21**	phosphoribosylglycinamide formyltransferase				
**ftsW**	D	RV2154C	**1.21**	**0.20**	cell division protein FtsW				
**mraY**	M		**1.21**	**0.27**	phospho-N-acetylmuramoyl-pentapeptide-transferase				
**MSMEG_5491**	I	Rv0975c	**1.21**	**0.59**	acyl-CoA dehydrogenase				
**MSMEG_5761**	S		**1.21**	**0.02**	cupin				
**MSMEG_3532**	E		**1.21**	**-0.27**	serine/threonine dehydratase				
**MSMEG_0935**	G		**1.21**	**-0.05**	2,3-bisphosphoglycerate-independent phosphoglycerate mutase				
**ksgA**	J		**1.21**	**0.33**	dimethyladenosine transferase				
**MSMEG_5183**	R	RV1144	**1.21**	**0.83**	3-hydroxyacyl-CoA dehydrogenase				
**moaE**	H	Rv0866	**1.21**	**-0.55**	molybdopterin converting factor subunit 2				
**MSMEG_4308**	R	RV2232	**1.20**	**-0.47**	5'-nucleotidase				
**MSMEG_2433**	M		**1.20**	**0.35**	D-alanyl-D-alanine carboxypeptidase				
**MSMEG_3647**	T	RV1827	**1.20**	**0.14**	forkhead-associated protein				
**MSMEG_4284**	R	RV2216	**1.20**	**0.83**	hypothetical protein				
**MSMEG_2511**	P	RV2895C	**1.20**	**0.44**	siderophore utilization protein				
**MSMEG_4221**	S	RV2149C	**1.20**	**-0.08**	hypothetical protein				
**MSMEG_5857**	I	RV0770	**1.20**	**-0.28**	oxidoreductase				
**MSMEG_0089**	R		**1.20**	**0.51**	chromosome condensation protein				
**MSMEG_4462**	R		**1.20**	**-0.18**	sodium:solute symporter				
**MSMEG_5554**	T		**1.20**	**0.28**	ANTAR domain-containing protein				
**MSMEG_0812**	E		**1.19**	**0.29**	amino acid transporter				
**MSMEG_0319**	I	RV0228	**1.19**	**-0.25**	acyltransferase				
**MSMEG_2507**	K		**1.19**	**-0.36**	IclR-family protein transcriptional regulator				
**mdcH**	I		**1.19**	**-0.12**	malonate decarboxylase subunit epsilon				
**MSMEG_4641**	C		**1.19**	**-0.15**	salicylate hydroxylase				
**ispF**	I	Rv3581c	**1.18**	**0.76**	2-C-methyl-D-erythritol 2,4-cyclodiphosphate synthase				
**MSMEG_0098**	H	RV0839	**1.18**	**0.14**	methyltransferase				
**glpK**	C	Rv3696c	**1.18**	**0.06**	glycerol kinase				
**MSMEG_2726**	E		**1.18**	**-0.26**	glutamate permease				
**MSMEG_6430**	C		**1.18**	**-0.53**	hypothetical protein				
**MSMEG_4702**	S	RV0102	**1.18**	**0.18**	ABC transporter permease				
**MSMEG_4976**	Q		**1.18**	**-0.35**	isochorismatase hydrolase				
**MSMEG_3083**	R		**1.17**	**-0.12**	nucleoside-diphosphate sugar epimerase				
**MSMEG_3895**	O	Rv2110c	**1.17**	**0.58**	proteasome subunit beta				
**MSMEG_2359**	E	RV3015C	**1.17**	**0.26**	methionine synthase, vitamin-B12 independent				
**MSMEG_0207**	K		**1.17**	**-0.70**	MarR family transcriptional regulator				
**MSMEG_1905**	I		**1.17**	**-0.04**	acyl-CoA dehydrogenase				
**MSMEG_6087**	V	Rv3593	**1.17**	**-0.32**	beta-lactamase				
**MSMEG_6175**	G		**1.17**	**0.05**	2-keto-3-deoxy-galactonokinase				
**sodC**	P	Rv0432	**1.17**	**0.48**	copper/zinc superoxide dismutase				

**MSMEG_2117**	G		**1.17**	**0.11**	beta-glucoside-specific EII permease				
**xseB**	L	Rv1107c	**1.17**	**0.03**	exodeoxyribonuclease VII small subunit				
**MSMEG_1661**	M	Rv3330	**1.17**	**-0.34**	D-alanyl-D-alanine carboxypeptidase				
**MSMEG_3484**	S		**1.17**	**-0.35**	cupin				
**MSMEG_5339**	E		**1.16**	**-0.26**	nitrile hydratase regulator1				
**MSMEG_2087**	M	RV3104C	**1.16**	**0.61**	transporter small conductance mechanosensitive ion channel (MscS) family protein				
**MSMEG_4464**	R	RV2315C	**1.16**	**0.29**	modulator of DNA gyrase				
**MSMEG_4022**	K		**1.16**	**-0.10**	TetR family transcriptional regulator				
**MSMEG_0901**	S	RV0459	**1.15**	**0.86**	hypothetical protein				
**murE**	M		**1.15**	**-0.48**	UDP-N-acetylmuramoylalanyl-D-glutamate--2,6-diaminopimelate ligase				
**MSMEG_5287**	R		**1.15**	**0.34**	dehydrogenase				
**MSMEG_4977**	P		**1.15**	**0.01**	3'(2'),5'-bisphosphate nucleotidase				
**dnaG**	L	Rv2343c	**1.15**	**-0.81**	DNA primase				
**MSMEG_4394**	K		**1.14**	**-0.64**	LysR family transcriptional regulator				
**MSMEG_2769**	P		**1.14**	**-0.34**	TrkB protein				
**MSMEG_4301**	Q		**1.14**	**0.04**	fatty-acid--CoA ligase				
**MSMEG_0092**	K	RV0144	**1.14**	**-0.21**	transcriptional regulatory protein				
**cobB**	H	Rv2848c	**1.14**	**0.06**	cobyrinic acid a,c-diamide synthase				
**MSMEG_6592**	R		**1.14**	**-0.55**	short-chain dehydrogenase/reductase SDR				
**MSMEG_6203**	E	RV3684	**1.14**	**-0.64**	cysteine synthase/cystathionine beta-synthase				
**MSMEG_0503**	G		**1.14**	**0.24**	DeoR family transcriptional regulator				
**MSMEG_6903**	K	RV0047C	**1.14**	**0.20**	PadR family transcriptional regulator				
**uvrB**	L	Rv1633	**1.14**	**0.81**	excinuclease ABC subunit B				
**MSMEG_5375**	K		**1.13**	**0.05**	GntR family transcriptional regulator				
**MSMEG_2936**	S	RV2609C	**1.13**	**-0.31**	hydrolase, nudix family protein				
**MSMEG_0283**	R		**1.13**	**-1.04**	short chain dehydrogenase				
**MSMEG_6126**	R		**1.13**	**-0.22**	D-isomer specific 2-hydroxyacid dehydrogenase				
**MSMEG_6796**	R		**1.13**	**0.16**	hypothetical protein				
**ispG**	I		**1.13**	**-0.24**	4-hydroxy-3-methylbut-2-en-1-yl diphosphate synthase				
**MSMEG_3790**	J	Rv1644	**1.13**	**0.57**	TrmH family RNA methyltransferase				
**MSMEG_1906**	C		**1.13**	**-0.25**	toluate 1,2-dioxygenase electron transfer component				
**MSMEG_4307**	S	RV2230C	**1.13**	**-0.09**	hypothetical protein				
**MSMEG_2680**	E		**1.13**	**0.09**	amino acid transporter				
**MSMEG_4964**	K		**1.13**	**0.62**	TetR family transcriptional regulator				
**MSMEG_2355**	R	RV3027C	**1.12**	**0.21**	hypothetical protein				
**MSMEG_4339**	K		**1.12**	**-0.26**	hypothetical protein				
**MSMEG_0972**	O	Rv0527	**1.12**	**-0.42**	cytochrome C biogenesis protein transmembrane region				
**MSMEG_4583**	R	RV2426C	**1.12**	**-0.49**	ATPase AAA				
**MSMEG_1574**	E	Rv3432c	**1.12**	**0.11**	glutamate decarboxylase				
**MSMEG_5383**	R		**1.12**	**0.29**	dehydrogenase/reductase SDR family protein member 4				
**MSMEG_1497**	I	Rv0752c	**1.12**	**0.76**	acyl-CoA dehydrogenase				
**MSMEG_2739**	M	RV2721C	**1.12**	**-0.43**	hypothetical protein				
**MSMEG_4205**	K		**1.11**	**-0.90**	transcriptional regulator				
**MSMEG_0470**	I		**1.11**	**-0.75**	para-nitrobenzyl esterase				
**MSMEG_4632**	S	RV2449C	**1.11**	**0.46**	saccharopine dehydrogenase				
**MSMEG_6409**	I	RV3816C	**1.11**	**0.33**	acyltransferase				
**MSMEG_0234**	O	RV0198C	**1.11**	**-0.30**	metallopeptidase				
**MSMEG_1340**	I	RV0635	**1.11**	**0.47**	(3R)-hydroxyacyl-ACP dehydratase subunit HadA				
**MSMEG_3187**	I	RV1565C	**1.11**	**0.35**	acyltransferase domain-containing protein				

**MSMEG_2249**	C		1.11	-0.42	hypothetical protein				
**aroC**	E		1.11	-0.33	chorismate synthase				
**thiS**	H	Rv0416	1.11	-0.10	sulfur carrier protein ThiS				
**MSMEG_3817**	S	RV1632C	1.11	-0.42	hypothetical protein				
**MSMEG_6238**	T		1.10	-0.22	two-component system sensor kinase				
**cobN**	H	Rv2062c	1.10	0.02	cobaltochelatase subunit CobN				
**MSMEG_0320**	R	RV0230C	1.10	-0.22	phosphotriesterase				
**MSMEG_3026**	L	RV2554C	1.10	0.14	Holliday junction resolvase-like protein				
**MSMEG_3266**	G		1.10	0.31	maltose/maltodextrin-binding protein				
**MSMEG_0032**	D	Rv2154c	1.10	-0.21	cell cycle protein, FtsW/RodA/SpoVE family protein				
**MSMEG_0527**	R		1.10	-0.33	2-hydroxycyclohexnecarboxyl-CoA dehydrogenase				
**MSMEG_4366**	L		1.10	-0.18	serine/threonine protein kinase				
**MSMEG_5641**	M		1.10	0.28	glycoside hydrolase family protein				
**MSMEG_3244**	S		1.10	0.15	hypothetical protein				
**MSMEG_4250**	P	RV2184C	1.10	0.31	membrane transport ATPase				
**obgE**	R		1.09	-0.08	GTPase ObgE				
**MSMEG_0141**	S	RV0176	1.09	-0.10	mce associated transmembrane protein				
**MSMEG_3170**	S		1.09	-0.30	hypothetical protein				
**guaA**	F		1.09	0.32	GMP synthase				
**MSMEG_0200**	C		1.09	-0.59	hypothetical protein				
**pyrC**	F		1.09	-0.19	dihydroorotase				
**MSMEG_5259**	O	RV1084	1.09	0.26	hypothetical protein				
**MSMEG_5850**	S	RV0775	1.09	-0.69	TetR family transcriptional regulator				
**MSMEG_5862**	R	RV0765C	1.09	0.22	short chain dehydrogenase				
**MSMEG_6098**	S	RV3603C	1.09	-0.19	chalcone/stilbene synthase				
**cobG**	P	Rv2064	1.09	0.07	precorrin-3B synthase				
**MSMEG_1939**	L	RV3204	1.09	0.71	6-O-methylguanine DNA methyltransferase				
**trmB**	R		1.09	-0.05	tRNA (guanine-N(7)-)-methyltransferase				
**MSMEG_0932**	G	RV0485	1.09	-0.36	ROK family protein				
**MSMEG_2130**	I		1.09	-0.17	acyl-CoA dehydrogenase				
**MSMEG_5830**	S	Rv0798c	1.09	-0.47	hypothetical protein				
**MSMEG_0127**	C	Rv0162c	1.09	0.33	oxidoreductase, zinc-binding dehydrogenase				
**MSMEG_5639**	I	Rv0905	1.09	0.70	enoyl-CoA hydratase				
**MSMEG_3235**	T		1.09	0.64	amino acid ABC transporter				
**MSMEG_5451**	L	RV1000C	1.09	-0.38	alkylated DNA repair protein				
**MSMEG_6682**	K		1.09	-0.11	RNA polymerase sigma-70 factor				
**MSMEG_1943**	L	RV3201C	1.09	0.06	ATP-dependent DNA helicase				
**MSMEG_5885**	R	RV3485C	1.09	0.11	short chain dehydrogenase				
**MSMEG_2431**	Q	RV2915C	1.09	-0.81	amidohydrolase				
**MSMEG_0737**	R	RV1544	1.08	-0.27	dehydrogenase				
**folB**	H	Rv3607c	1.08	0.23	dihydroneopterin aldolase				
**MSMEG_3240**	K		1.08	-0.10	LuxR family transcriptional regulator				
**MSMEG_3058**	P		1.08	-0.08	lipoprotein, nlpa family protein				
**MSMEG_1698**	R		1.08	0.18	ammonia monooxygenase				
**MSMEG_5762**	R		1.08	0.57	zinc-binding oxidoreductase				
**MSMEG_3528**	S	RV0116C	1.08	0.29	ErfK/YbiS/YcfS/YnhG family protein				
**MSMEG_1114**	R		1.08	0.51	short chain dehydrogenase				
**MSMEG_2767**	R	RV2695	1.08	-0.13	hypothetical protein				
**MSMEG_1062**	Q	Rv0542c	1.08	0.36	O-succinylbenzoic acid--CoA ligase				

**MSMEG_6673**	J		**1.08**	**-0.49**	6-aminohexanoate-cyclic-dimer hydrolase				
**pdxH**	H	Rv2607	**1.07**	**-1.26**	pyridoxamine 5'-phosphate oxidase				
**MSMEG_5512**	H	RV0958	**1.07**	**-0.48**	magnesium chelatase				
**MSMEG_1069**	E		**1.07**	**-0.09**	amino acid permease				
**lspA**	U	Rv1539	**1.07**	**-0.01**	lipoprotein signal peptidase				
**MSMEG_1376**	G	Rv0729	**1.07**	**-0.48**	xylulose kinase				
**MSMEG_2576**	L		**1.07**	**-0.43**	deoxyribodipyrimidine photo-lyase				
**MSMEG_5203**	S		**1.06**	**0.40**	DoxX subfamily protein				
**MSMEG_6179**	I		**1.06**	**0.48**	acetyl-coenzyme A synthetase				
**MSMEG_0741**	C		**1.06**	**-0.63**	hypothetical protein				
**MSMEG_1433**	Q		**1.06**	**-0.38**	thioesterase				
**MSMEG_2738**	C	Rv1442	**1.06**	**-0.51**	biotin sulfoxide reductase				
**MSMEG_2472**	K		**1.06**	**0.29**	AsnC family transcriptional regulator				
**MSMEG_1139**	K		**1.06**	**-0.17**	transcriptional regulator				
**MSMEG_5009**	V	RV1272C	**1.06**	**0.25**	ABC transporter				
**MSMEG_2941**	G		**1.06**	**0.22**	NAD dependent epimerase/dehydratase				
**MSMEG_4969**	K		**1.06**	**-0.14**	two-component system response regulator				
**MSMEG_1491**	E		**1.06**	**-0.55**	histidinol-phosphate aminotransferase 2				
**MSMEG_4349**	O	RV2264C	**1.05**	**-0.01**	hypothetical protein				
**pth**	J	Rv1014c	**1.05**	**-0.07**	peptidyl-tRNA hydrolase				
**MSMEG_2492**	C		**1.05**	**-0.44**	D-lactate dehydrogenase				
**MSMEG_6802**	G		**1.05**	**0.02**	ABC transporter ATP-binding protein				
**MSMEG_5309**	K		**1.05**	**-0.14**	hypothetical protein				
**rplV**	J	Rv0706	**1.05**	**-0.35**	50S ribosomal protein L22				
**MSMEG_1463**	E		**1.05**	**-0.14**	gamma-glutamyltranspeptidase				
**pstA**	P	Rv0930	**1.05**	**0.65**	phosphate ABC transporter permease				
**MSMEG_5795**	H	RV0812	**1.05**	**-0.25**	4-amino-4-deoxychorismate lyase				
**MSMEG_6591**	E		**1.05**	**-0.37**	class V aminotransferase				
**MSMEG_0657**	R	RV3161C	**1.05**	**-0.43**	Rieske (2Fe-2S) domain-containing protein				
**MSMEG_5906**	I	Rv3504	**1.05**	**0.79**	acyl-CoA dehydrogenase				
**MSMEG_0862**	H	Rv0866	**1.05**	**0.43**	molybdopterin biosynthesis protein MoeA				
**MSMEG_0244**	K		**1.05**	**-0.02**	two component response transcriptional regulatory protein prra				
**MSMEG_1219**	E		**1.05**	**-0.74**	ABC transporter permease				
**MSMEG_3524**	Q		**1.05**	**0.03**	linalool 8-monooxygenase				
**MSMEG_3269**	G		**1.05**	**0.16**	sugar ABC transporter ATP-binding protein				
**MSMEG_3856**	K		**1.04**	**-0.01**	CadC family transcriptional regulator				
**MSMEG_4511**	Q	Rv2380c	**1.04**	**-0.27**	linear gramicidin synthetase subunit B				
**MSMEG_1425**	R	RV0695	**1.04**	**-0.08**	creatininase subfamily protein				
**MSMEG_0250**	R	Rv0206c	**1.04**	**-0.07**	membrane protein, MmpL family protein				
**MSMEG_4217**	D		**1.04**	**0.13**	DivIVA protein				
**secG**	U	Rv1440	**1.04**	**-0.11**	preprotein translocase subunit SecG				
**MSMEG_1245**	H		**1.04**	**0.05**	phosphoadenosine phosphosulfate reductase				
**MSMEG_0619**	N	Rv0286	**1.04**	**-0.04**	PPE family protein				
**MSMEG_2596**	R	RV2859C	**1.04**	**-0.26**	peptidase C26				
**MSMEG_1701**	F		**1.04**	**-0.02**	purine nucleoside phosphorylase				
**MSMEG_0090**	S		**1.04**	**0.33**	hypothetical protein				
**pgm**	G	Rv3068c	**1.04**	**0.83**	phosphoglucomutase				
**MSMEG_4502**	S		**1.04**	**0.06**	16S ribosomal RNA methyltransferase RsmE				
**MSMEG_2733**	S	RV2728C	**1.04**	**0.21**	hypothetical protein				

MSMEG_2503	P		1.04	-0.41	hypothetical protein
MSMEG_1764	E	Rv3290c	1.04	-0.87	L-lysine aminotransferase
MSMEG_1571	R		1.03	-0.20	integral membrane protein
MSMEG_1589	C		1.03	0.28	hypothetical protein
MSMEG_1149	S	RV2390C	1.03	-0.64	hypothetical protein
MSMEG_1702	R	Rv3306c	1.03	0.48	amidohydrolase
MSMEG_0357	G		1.03	-0.53	transmembrane transporter
MSMEG_1214	G		1.03	0.02	oxidoreductase
MSMEG_4496	R	RV2367C	1.03	0.92	metalloprotease
MSMEG_5282	K		1.03	-0.66	transcriptional regulator
MSMEG_2278	R		1.03	-0.10	membrane-bound oxidoreductase
MSMEG_2760	G	Rv2702	1.03	-0.20	polyphosphate glucokinase
MSMEG_2501	P		1.03	0.40	nitrate/sulfonate/bicarbonate ABC transporter inner membrane protein
MSMEG_3761	O		1.03	0.62	Clp protease subunit
MSMEG_2349	S	RV3031	1.02	0.46	glycosyl hydrolase family protein
MSMEG_3619	R	RV1856C	1.02	-0.33	short chain dehydrogenase
MSMEG_3670	P		1.02	0.06	transporter small multidrug resistance (SMR) family protein
MSMEG_6929	R	RV3910	1.02	-0.17	hypothetical protein
MSMEG_3825	R		1.02	-0.15	3-oxoacyl-ACP reductase
MSMEG_1903	C	RV1866	1.02	-0.01	caib/baif family protein
MSMEG_4375	R		1.02	0.13	3-oxoacyl-ACP reductase
MSMEG_5076	M	RV1217C	1.02	0.27	ABC transporter
MSMEG_6645	R	RV1130	1.02	0.07	2-methylcitrate dehydratase
MSMEG_3671	G		1.02	0.06	integral membrane protein
MSMEG_2070	I		1.01	0.41	acyl-CoA dehydrogenase
MSMEG_5199	I		1.01	0.34	acyl-CoA dehydrogenase
trmD	J	Rv2906c	1.01	-0.54	tRNA (guanine-N(1)-)-methyltransferase
MSMEG_1648	K		1.01	0.38	transcriptional regulator
MSMEG_2566	R		1.01	0.75	3-alpha-(or 20-beta)-hydroxysteroid dehydrogenase
MSMEG_4219	S		1.01	-0.06	hypothetical protein
MSMEG_3508	R	RV1191	1.01	0.81	alpha/beta hydrolase
MSMEG_0762	Q		1.00	0.73	cytochrome P450
MSMEG_1195	S		1.00	-0.23	hypothetical protein
MSMEG_5210	R		1.00	-0.24	hydrolase
MSMEG_0422	G	RV1998C	1.00	-0.19	transferase
GeneName	COG_Code	RV Number	Geomean_Fold	Geomean_Fold	Product
MSMEG_1910	R		-1.14	-0.97	muconate cycloisomerase
MSMEG_2112	E		-1.11	0.19	hypothetical protein
MSMEG_1782	R		-1.03	0.26	oxidoreductase, short chain dehydrogenase/reductase
MSMEG_3022	S		-1.03	0.62	transglycosylase associated protein
MSMEG_6935	M	RV3915	-1.14	-0.51	N-acetylmuramoyl-L-alanine amidase
MSMEG_0266	E	RV2531C	-1.15	-0.46	arginine decarboxylase
MSMEG_1775	Q		-1.50	-0.31	cytochrome P450 monooxygenase
MSMEG_0375	I		-2.35	-0.62	phospholipase D
MSMEG_0670	O		-1.21	-0.68	FAD dependent oxidoreductase
MSMEG_0536	R		-1.33	0.04	intracellular protease PfpI family protein

**MSMEG_5616**	R	RV0911	**-1.28**	**0.68**	glyoxalase/bleomycin resistance protein/dioxygenase				
**MSMEG_6768**	C		**-1.39**	**0.13**	halogenase				
**MSMEG_6769**	P		**-1.03**	**-0.03**	transporter monovalent cation:proton antiporter-2 (CPA2) family protein				
**MSMEG_3364**	E		**-1.44**	**0.02**	RhtB family protein transporter				
**MSMEG_4618**	Q		**-1.60**	**-0.21**	isochorismatase				
**MSMEG_3536**	G		**-1.38**	**-0.14**	sugar transporter				
**MSMEG_5605**	C		**-1.60**	**-0.12**	cytochrome bd ubiquinol oxidase subunit I				
**MSMEG_4318**	P		**-1.02**	**-0.10**	hypothetical protein				
**wrbA**	R		**-1.08**	**-0.39**	NAD(P)H:quinone oxidoreductase, type IV				
**MSMEG_4063**	R		**-1.08**	**-0.02**	amidohydrolase				
**MSMEG_2563**	Q		**-1.21**	**-0.23**	cytochrome P450				
**MSMEG_5922**	I	Rv3522	**-1.27**	**-0.25**	lipid-transfer protein				
**MSMEG_6734**	C		**-2.01**	**0.07**	dibenzothiophene desulfurization enzyme A				
**MSMEG_3726**	G		**-1.76**	**-0.59**	alcohol dehydrogenase				
**MSMEG_6885**	C		**-1.93**	**-0.82**	MmcI protein				
**MSMEG_4822**	C		**-1.25**	**0.02**	hypothetical protein				
**MSMEG_1392**	C		**-1.65**	**-0.64**	alcohol dehydrogenase				
**recC**	L	Rv0631c	**-1.14**	**0.08**	exodeoxyribonuclease V subunit gamma				
**anmK**	O		**-1.56**	**-0.20**	anhydro-N-acetylmuramic acid kinase				
**dnaA**	L	Rv0001	**-1.19**	**-0.70**	chromosomal replication initiation protein				
**cheR**	T		**-1.47**	**-0.52**	chemotaxis protein CheR				
**MSMEG_6264**	E		**-1.44**	**-0.21**	oxidoreductase				
**MSMEG_2098**	Q		**-1.15**	**-0.04**	fumarylacetoacetate hydrolase				
**MSMEG_1972**	C		**-1.12**	**0.02**	methane monooxygenase component C				
**MSMEG_2846**	P		**-1.10**	**-0.16**	ABC transporter permease				
**MSMEG_3362**	I		**-1.39**	**-0.24**	enoyl-CoA hydratase				
**MSMEG_1228**	P		**-1.03**	**0.38**	sulfatase				
**MSMEG_2539**	J	RV0560C	**-1.27**	**-0.26**	thiopurine S-methyltransferase (tpmt) superfamily protein				
**MSMEG_4814**	I		**-1.27**	**0.04**	hypothetical protein				
**MSMEG_3318**	C		**-1.29**	**-0.87**	oxidoreductase				
**MSMEG_1570**	R		**-1.02**	**0.02**	carboxylesterase				
**MSMEG_2208**	I		**-1.02**	**0.33**	acyl-CoA dehydrogenase				
**MSMEG_6710**	I		**-1.17**	**-0.49**	alpha/beta hydrolase				
**MSMEG_3994**	R		**-1.07**	**0.30**	short chain dehydrogenase				
**MSMEG_1319**	Q		**-1.09**	**-0.10**	Asp/Glu racemase				
**MSMEG_4420**	S		**-1.89**	**-0.50**	cupin				
**MSMEG_3042**	F	Rv1379	**-1.15**	**-0.37**	bifunctional pyrimidine regulatory protein PyrR/uracil phosphoribosyltransferase				
**MSMEG_3934**	G		**-1.99**	**-0.49**	phosphoenolpyruvate synthase				
**MSMEG_5124**	C	Rv1175c	**-1.41**	**-0.63**	2,4-dienoyl-CoA reductase				
**MSMEG_2146**	S		**-1.07**	**0.34**	gp15 protein				
**MSMEG_1303**	K		**-1.10**	**0.33**	transcriptional regulator				
**MSMEG_0447**	O		**-1.28**	**0.21**	hypothetical protein				
**MSMEG_4646**	C	RV2455C	**-1.15**	**-0.46**	pyruvate synthase				
**MSMEG_3095**	G		**-1.08**	**-0.32**	D-ribose-binding periplasmic protein				
**MSMEG_4387**	P		**-1.52**	**0.21**	ABC transporter permease				
**MSMEG_4066**	Q		**-1.12**	**0.19**	hypothetical protein				
**pcaG**	Q		**-1.04**	**0.10**	protocatechuate 3,4-dioxygenase subunit alpha				
**MSMEG_0635**	S	RV0309	**-1.68**	**-0.43**	hypothetical protein				
**MSMEG_1448**	P		**-1.36**	**0.18**	integral membrane transporter				

**pqqB**	R		**-1.55**	**0.22**	pyrroloquinoline quinone biosynthesis protein PqqB				
**ltaE**	E		**-1.04**	**0.14**	L-threonine aldolase				
**MSMEG_0351**	Q		**-1.41**	**-0.10**	virulence factor mce family protein				
**MSMEG_6367**	R	Rv3782	**-1.26**	**-0.22**	glycosyl transferase family protein				
**MSMEG_5198**	I		**-1.43**	**-0.68**	carnitinyl-CoA dehydratase				
**MSMEG_6763**	R	RV0077C	**-1.70**	**0.26**	oxidoreductase				
**MSMEG_2194**	K		**-1.04**	**-0.54**	MerR family transcriptional regulator				
**MSMEG_0517**	G	RV2039C	**-1.32**	**0.03**	sugar binding-protein dependent transporter system permease				
**MSMEG_0474**	M		**-1.48**	**0.18**	glucosamine--fructose-6-phosphate aminotransferase (isomerizing)				
**MSMEG_5130**	E	Rv1166	**-1.35**	**-0.46**	extracellular solute-binding protein				
**MSMEG_1797**	R		**-1.11**	**-0.43**	salicylate esterase				
**MSMEG_1963**	R		**-1.27**	**-0.63**	transcriptional regulatory protein				
**MSMEG_6686**	I		**-1.06**	**-0.15**	glutaryl-CoA dehydrogenase				
**MSMEG_0288**	E		**-1.39**	**-0.23**	FAD dependent oxidoreductase				
**MSMEG_5372**	T	Rv1028c	**-1.05**	**-0.16**	sensor protein KdpD				
**MSMEG_1086**	P		**-1.67**	**0.14**	ABC transporter permease				
**MSMEG_1297**	R		**-1.21**	**-0.23**	hydroxydechloroatrazine ethylaminohydrolase				
**MSMEG_0734**	R		**-1.02**	**-0.16**	Rieske (2Fe-2S) domain-containing protein				
**MSMEG_4393**	G		**-1.37**	**0.13**	carboxyvinyl-carboxyphosphonate phosphorylmutase				
**MSMEG_3929**	C		**-1.07**	**0.01**	[NiFe] hydrogenase subunit delta				
**MSMEG_1151**	K		**-1.34**	**-0.21**	DNA-binding protein				
**MSMEG_4110**	I		**-1.49**	**-0.26**	3-hydroxyacyl-CoA dehydrogenase				
**MSMEG_2254**	R		**-1.12**	**1.17**	oxalate decarboxylase OxdC				
**kdpB**	P	Rv1030	**-1.04**	**0.11**	potassium-transporting ATPase subunit B				
**MSMEG_1034**	R		**-1.18**	**0.00**	esterase				
**cysE**	E	Rv2335	**-1.03**	**0.06**	serine O-acetyltransferase				
**MSMEG_1486**	K		**-1.07**	**-0.28**	RNA polymerase sigma factor SigL				
**argB**	E	Rv1654	**-1.20**	**-0.37**	acetylglutamate kinase				
**MSMEG_4606**	D		**-1.75**	**-0.25**	hypothetical protein				
**MSMEG_2081**	I	Rv3139	**-1.21**	**-0.88**	acyl-CoA dehydrogenase				
**MSMEG_0902**	M	Rv0644c	**-1.27**	**-0.01**	cyclopropane-fatty-acyl-phospholipid synthase				
**MSMEG_4879**	C		**-1.49**	**-0.10**	hypothetical protein				
**MSMEG_3579**	M	RV2434C	**-1.36**	**-0.22**	transmembrane protein				
**MSMEG_3809**	C		**-1.43**	**-0.03**	hypothetical protein				
**MSMEG_6725**	R		**-1.03**	**0.20**	ABC transporter ATP-binding protein				
**MSMEG_4809**	R		**-1.65**	**0.02**	Rieske (2Fe-2S) domain-containing protein				
**MSMEG_4077**	I		**-1.12**	**0.07**	enoyl-CoA hydratase				
**MSMEG_6718**	G		**-1.03**	**-0.03**	transporter major facilitator family protein				
**MSMEG_2102**	P		**-1.19**	**-0.35**	nitrate transport ATP-binding protein NrtD				
**MSMEG_2229**	I		**-1.55**	**0.40**	enoyl-CoA hydratase				
**MSMEG_1796**	C		**-1.72**	**-0.96**	membrane protein				
**MSMEG_4853**	E		**-1.18**	**-0.08**	peptidase, M24 family protein				
**MSMEG_3017**	S	RV2567	**-1.27**	**0.06**	hypothetical protein				
**MSMEG_4731**	Q	Rv1521	**-1.32**	**-0.19**	acyl-CoA synthetase				
**MSMEG_3090**	G		**-1.10**	**-0.10**	ribose transporter permease RbsC				
**MSMEG_3970**	J	RV3175	**-1.13**	**-0.14**	glutamyl-tRNA(Gln) amidotransferase subunit A				
**MSMEG_2209**	K		**-1.63**	**-0.56**	GntR family transcriptional regulator				
**MSMEG_2845**	P		**-1.29**	**0.07**	ABC transporter permease				
**purH**	F	Rv0957	**-1.16**	**-0.75**	bifunctional phosphoribosylaminoimidazolecarboxamide formyltransferase/IMP cyclohydrolase				

**MSMEG_3378**	V		**-1.80**	**-0.58**	beta-lactamase					
**MSMEG_0125**	S		**-1.41**	**-0.20**	integral membrane protein					
**MSMEG_5579**	K		**-1.26**	**-0.09**	MarR family transcriptional regulator					
**MSMEG_1155**	I		**-1.27**	**0.08**	carnitinyl-CoA dehydratase					
**MSMEG_4521**	P		**-1.24**	**-0.17**	Tat (twin-arginine translocation) pathway signal sequence					
**MSMEG_0506**	G		**-1.37**	**0.00**	ABC transporter permease					
**MSMEG_0487**	P		**-1.00**	**0.10**	ABC transporter permease					
**MSMEG_5520**	C	RV0953C	**-1.15**	**0.54**	hypothetical protein					
**MSMEG_1964**	C	RV1774	**-1.08**	**0.41**	mitomycin radical oxidase					
**MSMEG_3657**	C	RV1817	**-1.35**	**-0.21**	hypothetical protein					
**MSMEG_6389**	M	Rv3795	**-1.37**	**-0.16**	arabinosyltransferase A					
**MSMEG_0534**	G		**-1.34**	**-0.12**	permease, major facilitator family protein					
**MSMEG_4422**	C		**-1.08**	**0.25**	oxidoreductase					
**MSMEG_2096**	R		**-2.15**	**-0.84**	integral membrane protein					
**MSMEG_4135**	R		**-1.16**	**0.25**	lysine decarboxylase superfamily protein					
**MSMEG_0293**	R		**-1.08**	**0.11**	Rieske (2Fe-2S) domain-containing protein					
**MSMEG_1137**	E		**-1.35**	**-0.37**	amino acid permease					
**MSMEG_2069**	R		**-1.00**	**0.34**	phosphotransferase enzyme family protein					
**MSMEG_6263**	E		**-1.08**	**0.20**	glutamate synthase					
**MSMEG_2108**	R		**-1.09**	**-0.70**	hypothetical protein					
**MSMEG_0149**	H	Rv0423c	**-1.04**	**-0.17**	thiamine biosynthesis protein ThiC					
**MSMEG_2810**	G		**-1.12**	**-0.11**	major facilitator superfamily protein					
**MSMEG_6712**	C		**-1.36**	**0.55**	maleylacetate reductase					
**MSMEG_5609**	Q	RV0913C	**-1.22**	**0.90**	carotenoid oxygenase					
**MSMEG_2005**	G		**-1.33**	**0.66**	sugar phosphate isomerase/epimerase					
**MSMEG_2807**	K		**-1.43**	**-0.12**	two-component system response regulator					
**MSMEG_3557**	E		**-1.09**	**0.56**	amino acid permease					
**MSMEG_5593**	H		**-1.24**	**0.12**	pyruvate dehydrogenase					
**MSMEG_2909**	R		**-1.20**	**0.18**	starvation-sensing protein RspA					
**MSMEG_5954**	M		**-1.30**	**-0.32**	cell surface polysaccharide biosynthesis					
**MSMEG_3346**	G		**-1.63**	**-0.04**	hydroxyacid aldolase					
**MSMEG_0174**	S		**-1.14**	**0.32**	inner membrane protein					
**MSMEG_2657**	R	RV2782C	**-1.19**	**-0.11**	peptidase, M16 family protein					
**MSMEG_5021**	R		**-1.33**	**0.06**	alcohol dehydrogenase					
**MSMEG_0170**	G		**-1.77**	**-0.84**	transmembrane transporter					
**MSMEG_5509**	R		**-1.07**	**0.10**	hypothetical protein					
**MSMEG_5364**	R		**-1.29**	**-0.13**	amidohydrolase					
**MSMEG_5420**	P		**-1.15**	**-0.05**	Tat-translocated enzyme					
**MSMEG_6872**	V		**-1.41**	**0.17**	beta-lactamase					
**MSMEG_4062**	J		**-1.19**	**0.08**	glutamyl-tRNA(Gln) amidotransferase subunit A					
**MSMEG_3991**	R		**-1.12**	**0.48**	cyclase					
**MSMEG_4049**	G		**-1.31**	**-0.02**	sugar transporter permease					
**rpsO**	J	Rv2785c	**-1.06**	**0.08**	30S ribosomal protein S15					
**MSMEG_2464**	C		**-1.65**	**0.20**	2Fe-2S-binding domain-containing protein					
**MSMEG_6745**	K		**-1.07**	**-0.01**	GntR family transcriptional regulator					
**MSMEG_2067**	H	RV2675C	**-1.30**	**-0.27**	methyltransferase type 12					
**MSMEG_4093**	C	RV2298	**-1.25**	**0.12**	oxidoreductase, aldo/keto reductase					
**MSMEG_0654**	C		**-1.03**	**0.16**	hypothetical protein					
**MSMEG_4092**	C		**-1.05**	**-0.48**	alkanal monooxygenase					

**MSMEG_1506**	S		-1.21	0.29	TetR family transcriptional regulator				
**MSMEG_6296**	R		-1.41	0.62	5-exo-alcohol dehydrogenase				
**MSMEG_0438**	P	RV0265C	-1.23	0.02	periplasmic binding protein				
**MSMEG_4793**	Q		-1.10	0.23	virulence factor Mce family protein				
**MSMEG_2981**	E		-1.62	-0.47	branched-chain amino acid ABC transporter permease				
**cadA**	P	Rv1469	-1.07	-0.06	cadmium-translocating P-type ATPase				
**MSMEG_3517**	E		-1.14	-0.11	HAD-superfamily hydrolase				
**MSMEG_4456**	S	RV2313C	-1.16	-0.12	hypothetical protein				
**MSMEG_0339**	C	RV3520C	-1.26	-0.07	FMN-dependent monooxygenase				
**MSMEG_6795**	I		-1.91	-0.48	enoyl-CoA hydratase/isomerase				
**MSMEG_2772**	E	RV2690C	-1.35	0.50	amino acid permease				
**argS**	J	Rv1292	-1.37	-0.13	arginyl-tRNA synthetase				
**MSMEG_5839**	E	Rv0781	-1.12	-0.08	protease 2				
**MSMEG_3411**	S		-1.03	0.37	MOSC domain-containing protein				
**MSMEG_3367**	R		-1.17	0.47	short-chain dehydrogenase/reductase SDR				
**tgt**	J		-1.07	-0.17	queuine tRNA-ribosyltransferase				
**MSMEG_0126**	R		-1.29	0.18	mandelate racemase/muconate lactonizing enzyme				
**MSMEG_4949**	J	Rv1300	-1.23	0.02	HemK family modification methylase				
**MSMEG_6237**	H		-1.22	0.08	hypothetical protein				
**MSMEG_6908**	K	RV0043C	-1.09	-0.47	HTH-type transcriptional regulator				
**MSMEG_4020**	I		-1.11	0.25	enoyl-CoA hydratase/isomerase				
**MSMEG_4610**	Q		-1.07	0.21	IgiC				
**glyS**	J		-1.34	-0.09	glycyl-tRNA synthetase				
**MSMEG_5979**	J		-1.10	-0.01	transferase				
**MSMEG_0205**	S		-1.67	-0.86	tetracenomycin polyketide synthesis hydroxylase TcmH				
**MSMEG_4008**	R		-1.30	0.17	oxidoreductase, 2OG-Fe(II) oxygenase				
**MSMEG_5671**	S		-1.12	0.07	prolyl-tRNA synthetase				
**MSMEG_1452**	S		-1.20	0.03	sulfatase-modifying factor 1				
**MSMEG_3556**	H		-1.40	-0.09	integral membrane transporter				
**MSMEG_0461**	S		-1.53	0.07	CinZ protein				
**MSMEG_3731**	E		-1.40	-0.24	dipeptidase 2				
**MSMEG_6317**	E		-1.18	-0.54	lipolytic protein G-D-S-L				
**MSMEG_6660**	F		-1.09	0.08	permease, cytosine/purines, uracil, thiamine, allantoin family protein				
**MSMEG_1231**	S	RV2272	-1.06	0.11	inner membrane protein YidH				
**MSMEG_6639**	K		-1.03	-0.06	GntR family transcriptional regulator				
**MSMEG_0545**	T	RV0386	-1.32	0.00	LuxR family transcriptional regulator				
**MSMEG_5818**	Q	Rv0169	-1.29	0.50	virulence factor Mce family protein				
**MSMEG_4056**	G		-1.06	-0.02	hypothetical protein				
**MSMEG_3812**	I		-1.05	-0.35	acyl-CoA thioesterase				
**MSMEG_0157**	H	Rv0118c	-1.25	-0.10	oxalyl-CoA decarboxylase				
**MSMEG_5812**	R		-1.23	-0.19	short chain dehydrogenase				
**MSMEG_4082**	C		-1.33	-0.19	monoxygenase				
**MSMEG_3563**	G	RV1877	-1.19	-0.32	drug transporter				
**MSMEG_5416**	M	Rv1022	-1.05	-0.10	LpqU protein				
**MSMEG_1154**	C		-1.25	0.59	formyl-coenzyme A transferase				
**MSMEG_3273**	G		-1.20	-0.08	glutamyl aminopeptidase, M42 family protein				
**MSMEG_2172**	P		-1.03	0.06	dicarboxylate-carrier protein				
**MSMEG_3960**	K		-1.08	-0.15	transcriptional regulator				
**MSMEG_0281**	E		-1.60	0.24	choline dehydrogenase				

**rpsF**	J	Rv0053	-1.00	-0.25	30S ribosomal protein S6				
**MSMEG_4844**	I		-1.09	-0.07	acyl-CoA dehydrogenase				
**MSMEG_4736**	S		-1.18	-0.52	hypothetical protein				
**MSMEG_5615**	S		-1.17	0.33	hypothetical protein				
**MSMEG_2165**	G		-1.39	0.30	transketolase				
**MSMEG_6459**	E	Rv3859c	-1.02	-0.07	ferredoxin-dependent glutamate synthase 1				
**MSMEG_2525**	E		-1.19	-0.47	amino acid permease				
**MSMEG_0535**	K		-1.39	0.33	GntR family transcriptional regulator				
**MSMEG_5948**	M		-1.23	-0.06	glycosyl transferase family protein				
**MSMEG_0546**	H		-1.30	-0.02	hypothetical protein				
**MSMEG_2544**	K		-1.42	-0.21	LysR family transcriptional regulator				
**MSMEG_4154**	L	RV3431C	-1.53	0.13	transposase, Mutator family protein				
**MSMEG_0376**	K		-1.20	0.20	AraC family transcriptional regulator				
**mhpA**	C		-1.31	0.15	3-(3-hydroxyphenyl)propionate hydroxylase				
**MSMEG_5984**	M		-1.06	-0.02	UDP-phosphate galactosephosphotransferase				
**MSMEG_4123**	I		-1.03	-0.11	3-hydroxyisobutyrate dehydrogenase				
**MSMEG_2007**	Q		-1.13	0.09	HpcE protein				
**MSMEG_0511**	M		-1.02	0.56	sugar isomerase				
**MSMEG_4075**	C		-1.28	-0.14	CoA-binding protein				
**hypB**	K		-1.00	-0.27	hydrogenase nickel incorporation protein HypB				
**MSMEG_0350**	Q	Rv1970	-1.02	0.22	virulence factor Mce family protein				
**MSMEG_3382**	K		-1.04	-0.01	ArsR family transcriptional regulator				
**MSMEG_0778**	K		-1.50	0.53	transcriptional regulator				
**MSMEG_6705**	K		-1.28	0.15	regulatory protein				
**MSMEG_4103**	C		-1.24	0.25	alkanesulfonate monooxygenase				
**MSMEG_2982**	E		-1.02	0.22	periplasmic binding protein				
**MSMEG_2538**	K	RV2887	-1.46	0.23	MarR family transcriptional regulator				
**MSMEG_2844**	R		-1.59	-0.06	ABC transporter ATP-binding protein				
**MSMEG_2918**	R		-1.18	0.20	short-chain dehydrogenase/reductase SDR				
**MSMEG_6880**	E		-1.01	0.20	hydrophobic amino acid ABC transporter				
**MSMEG_0117**	R	RV1190	-1.28	-0.56	hydrolase				
**MSMEG_2011**	K		-1.01	-0.32	LacI family transcriptional regulator				
**MSMEG_0341**	C		-1.63	-0.18	hypothetical protein				
**MSMEG_0475**	M		-1.33	0.25	nucleotide-sugar dehydrogenase				
**MSMEG_3373**	G		-1.42	-0.31	major facilitator superfamily protein				
**MSMEG_3544**	C		-1.19	0.36	hypothetical protein				
**MSMEG_5810**	C		-1.84	0.44	monooxygenase				
**MSMEG_0531**	I		-1.31	-0.05	acyl-CoA dehydrogenase				
**MSMEG_4396**	Q		-1.21	0.12	isochorismatase hydrolase				
**MSMEG_1133**	H	Rv0562	-1.33	-0.15	bifunctional short chain isoprenyl diphosphate synthase				
**MSMEG_4023**	C	RV0794C	-1.24	0.39	oxidoreductase				
**MSMEG_6242**	C		-1.81	0.49	alcohol dehydrogenase				
**MSMEG_1222**	L		-1.35	0.35	ISMsm6, transposase				
**MSMEG_0881**	S		-1.10	-0.06	hypothetical protein				
**rpsT**	J	Rv2412	-1.06	-0.13	30S ribosomal protein S20				
**sucD**	C	Rv0952	-1.03	0.22	succinyl-CoA synthetase subunit alpha				
**MSMEG_2297**	O		-1.22	-0.76	glutaredoxin				
**MSMEG_3157**	S	RV1491C	-1.05	-0.15	hypothetical protein				
**hypF**	O		-1.24	0.05	[NiFe] hydrogenase maturation protein HypF				

**MSMEG_6358**	S		**-1.15**	**0.31**	mesocentin				
**MSMEG_2908**	G		**-1.04**	**-0.14**	2-keto-3-deoxygluconate kinase				
**MSMEG_6340**	R		**-1.12**	**0.26**	short-chain dehydrogenase/reductase SDR				
**MSMEG_2924**	M		**-1.21**	**0.18**	permease binding-protein component				
**MSMEG_2926**	E		**-1.10**	**0.22**	glycine betaine/carnitine/choline transport ATP-binding protein opuCA				
**MSMEG_5443**	C		**-1.10**	**-0.04**	dehydrogenase				
**MSMEG_0781**	E		**-1.46**	**-0.13**	amino acid permease				
**MSMEG_0326**	Q		**-1.50**	**-0.18**	AMP-dependent synthetase/ligase				
**MSMEG_6939**	D	Rv3918c	**-1.29**	**-0.11**	Soj family protein				
**MSMEG_1458**	H		**-1.32**	**0.29**	tena/thi-4 family protein				
**MSMEG_4855**	R		**-1.40**	**0.16**	amidohydrolase				
**hisG**	E	Rv2121c	**-1.05**	**0.26**	ATP phosphoribosyltransferase				
**MSMEG_3715**	Q		**-1.56**	**-0.17**	linear gramicidin synthetase subunit C				
**MSMEG_5394**	P	Rv1031	**-1.01**	**-0.01**	potassium-transporting ATPase subunit C				
**MSMEG_0147**	I		**-1.43**	**-0.42**	C-5 sterol desaturase				
**MSMEG_4074**	R		**-1.13**	**-0.53**	peroxisomal trans-2-enoyl-CoA reductase				
**MSMEG_3987**	C		**-1.30**	**-0.25**	FAD dependent oxidoreductase				
**MSMEG_4819**	C		**-1.13**	**-0.37**	hypothetical protein				
**MSMEG_4541**	R		**-1.49**	**0.31**	ABC transporter ATP-binding protein				
**MSMEG_2166**	G	Rv3379c	**-1.15**	**-0.39**	transketolase				
**MSMEG_3358**	S		**-1.16**	**-0.40**	YaeQ protein				
**MSMEG_6668**	P		**-1.16**	**0.29**	ABC transporter periplasmic protein				
**MSMEG_4119**	I		**-1.20**	**0.76**	3-hydroxybutyryl-CoA dehydratase				
**MSMEG_0151**	C	Rv0156	**-1.40**	**-0.39**	PntAB protein				
**MSMEG_6851**	R	RV1910C	**-1.44**	**-0.09**	phosphatidylethanolamine-binding protein				
**MSMEG_4357**	R		**-1.32**	**-0.15**	ABC transporter ATP-binding protein				
**MSMEG_0514**	G		**-1.25**	**-0.02**	alpha-galactosidase				
**MSMEG_0270**	R		**-1.06**	**0.24**	aminoglycoside phosphotransferase				
**hydA**	F		**-1.33**	**0.00**	phenylhydantoinase				
**MSMEG_6695**	Q		**-1.11**	**0.73**	cytochrome P450				
**MSMEG_4551**	C		**-1.01**	**-0.13**	monoxygenase				
**MSMEG_2236**	I		**-1.29**	**0.22**	thiolase				
**MSMEG_3216**	O	Rv1608c	**-1.08**	**0.33**	peroxiredoxin Q				
**MSMEG_6911**	E		**-1.30**	**0.10**	ABC transporter ATP-binding protein				
**MSMEG_0338**	I		**-1.58**	**0.07**	acyl-CoA dehydrogenase				
**MSMEG_3006**	C		**-1.18**	**0.08**	Fe-dependent alcohol dehydrogenase				
**MSMEG_0185**	R		**-1.10**	**-0.39**	MmpL6 protein				
**MSMEG_1138**	C		**-1.01**	**-0.18**	alcohol dehydrogenase				
**MSMEG_5595**	K		**-1.00**	**0.08**	MarR family transcriptional regulator				
**araB**	C		**-1.66**	**-0.12**	ribulokinase				
**MSMEG_3664**	P	RV3236C	**-1.01**	**-0.39**	transporter monovalent cation:proton antiporter-2 (CPA2) family protein				
**MSMEG_4118**	I		**-1.48**	**0.27**	acyl-CoA dehydrogenase				
**MSMEG_2554**	R		**-1.04**	**-0.02**	phosphotransferase enzyme family protein				
**glnT**	E		**-1.12**	**-0.17**	glutamine synthetase				
**MSMEG_1177**	F		**-1.45**	**0.03**	cytosine/purines uracil thiamine allantoin permease				
**kdpA**	P	Rv1029	**-1.05**	**-0.07**	potassium-transporting ATPase A				
**MSMEG_4850**	R		**-1.12**	**-0.37**	short-chain dehydrogenase/reductase SDR				
**MSMEG_3730**	K		**-1.21**	**-0.03**	Fis family transcriptional regulator				
**MSMEG_6708**	R	Rv0134	**-1.00**	**-0.33**	epoxide hydrolase				

**MSMEG_1152**	G		-1.44	0.41	citrate-proton symporter					
**MSMEG_3982**	I		-1.64	-0.01	acyl-CoA dehydrogenase					
**MSMEG_6838**	R		-1.15	0.17	esterase					
**MSMEG_4039**	C		-1.35	0.11	aryl-alcohol dehydrogenase					
**MSMEG_2311**	R		-1.33	-0.06	hypothetical protein					
**MSMEG_2681**	R	Rv0840c	-1.14	0.01	proline imino-peptidase					
**purC**	F	Rv0780	-1.06	0.12	phosphoribosylaminoimidazole-succinocarboxamide synthase					
**MSMEG_0481**	C		-1.52	0.58	FAD dependent oxidoreductase					
**MSMEG_1123**	R		-1.09	0.10	cobalamin synthesis protein					
**MSMEG_4801**	R		-1.05	0.02	carveol dehydrogenase					
**MSMEG_6525**	A		-1.56	-0.35	hypothetical protein					
**MSMEG_1330**	K		-1.13	-0.06	MarR family transcriptional regulator					
**MSMEG_0212**	S		-1.02	0.14	lyase					
**MSMEG_1117**	K		-1.15	0.37	transcriptional regulator					
**MSMEG_5024**	L		-1.62	-0.13	T/U mismatch-specific DNA glycosylase					
**MSMEG_5581**	S		-1.04	0.12	hypothetical protein					
**MSMEG_3400**	J	Rv3375	-1.31	0.12	glutamyl-tRNA(Gln) amidotransferase subunit A					
**MSMEG_0508**	G	RV2038C	-1.01	-0.33	glycerol-phosphate porter					
**MSMEG_4112**	Q		-1.58	0.43	cyclohexanecarboxylate-CoA ligase					
**MSMEG_6790**	G		-1.02	-0.06	AP endonuclease, family protein 2					
**MSMEG_3578**	T	RV2435C	-1.07	-0.36	cyclase					
**MSMEG_1451**	P	Rv0711	-1.23	-0.70	arylsulfatase					
**MSMEG_2549**	G	RV1672C	-1.25	-0.47	major facilitator superfamily protein					
**MSMEG_4740**	C		-1.25	0.25	glycosyltransferase 28					
**MSMEG_0524**	R		-1.00	0.26	short chain dehydrogenase					
**MSMEG_6835**	I		-1.44	-0.02	Fatty acid desaturase					
**MSMEG_0476**	M		-1.10	0.42	chitin synthase					
**narH**	C	Rv1162	-1.35	-0.06	nitrate reductase subunit beta					
**MSMEG_0349**	Q	Rv1969	-1.32	0.57	virulence factor mce family protein					
**MSMEG_2217**	Q		-1.55	0.09	AMP-dependent synthetase/ligase					
**MSMEG_2912**	G		-1.34	-0.13	inner membrane metabolite transporter YdfJ					
**MSMEG_1795**	R		-1.35	0.16	2-deoxy-scyllo-inosamine dehydrogenase					
**MSMEG_2812**	I		-1.23	-0.23	C-5 sterol desaturase					
**MSMEG_2677**	R		-1.52	0.01	large membrane protein					
**rbsR**	K		-1.40	-0.19	ribose operon repressor					
**MSMEG_1390**	I	Rv0675	-1.05	0.33	enoyl-CoA hydratase					
**MSMEG_1798**	G		-1.02	-0.24	major facilitator superfamily protein					
**MSMEG_0486**	P		-1.30	-0.11	ABC transporter periplasmic protein					
**MSMEG_5626**	C		-1.13	0.04	luciferase					
**pstS**	P		-1.09	0.07	phosphate ABC transporter substrate-binding protein					
**MSMEG_2537**	G		-1.14	0.31	transporter protein					
**MSMEG_1840**	C	Rv3251c	-1.02	-0.18	rubredoxin					
**MSMEG_3436**	S	RV0366C	-1.02	-0.25	hypothetical protein					
**MSMEG_3434**	O		-1.04	-0.09	peptidyl-prolyl cis-trans isomerase domain-containing protein					
**MSMEG_4472**	G		-1.09	-0.02	multidrug-efflux transporter protein					
**MSMEG_2996**	C		-1.24	-0.16	hypothetical protein					
**MSMEG_2856**	Q		-1.32	0.18	virulence factor Mce family protein					
**MSMEG_0655**	R		-1.09	0.29	glucose 1-dehydrogenase					
**MSMEG_1220**	E		-1.47	0.00	ABC transporter permease					

**MSMEG_1082**	K		**-1.41**	**-0.06**	response regulator				
**MSMEG_3349**	Q		**-1.41**	**0.30**	trap dicarboxylate transporter dctq subunit				
**MSMEG_6874**	C		**-1.35**	**-0.09**	aldehyde dehydrogenase				
**MSMEG_1607**	R		**-1.15**	**-0.11**	tautomerase				
**MSMEG_5728**	G		**-1.00**	**0.22**	polysaccharide deacetylase				
**MSMEG_1780**	P		**-1.37**	**0.21**	hypothetical protein				
**MSMEG_0530**	R		**-1.46**	**-0.14**	short chain dehydrogenase				
**MSMEG_0343**	K		**-1.07**	**0.18**	TetR family transcriptional regulator				
**MSMEG_0543**	C		**-1.18**	**-0.08**	hypothetical protein				
**MSMEG_2212**	R	RV2263	**-1.15**	**0.65**	short chain dehydrogenase				
**MSMEG_0158**	C		**-1.14**	**-0.95**	formyl-coenzyme A transferase				
**MSMEG_1140**	C		**-1.35**	**0.60**	NAD(P)H-dependent glycerol-3-phosphate dehydrogenase				
**MSMEG_0515**	G		**-1.79**	**-0.21**	sugar transporter sugar binding lipoprotein				
**MSMEG_1513**	C		**-1.07**	**0.14**	hypothetical protein				
**MSMEG_6309**	E		**-1.28**	**-0.05**	ABC transporter ATP-binding protein				
**MSMEG_3040**	V	RV1367C	**-1.30**	**-0.42**	beta-lactamase				
**MSMEG_3805**	R		**-1.16**	**-0.15**	oxidoreductase, short chain dehydrogenase/reductase				
**pcaH**	Q		**-1.20**	**0.05**	protocatechuate 3,4-dioxygenase subunit beta				
**MSMEG_3951**	I		**-1.45**	**-0.55**	hypothetical protein				
**pqqE**	R		**-1.13**	**0.03**	pyrroloquinoline quinone biosynthesis protein PqqE				
**MSMEG_0824**	R	RV0421C	**-1.15**	**0.11**	hypothetical protein				
**MSMEG_4389**	C		**-1.18**	**0.29**	monooxygenase, NtaA/SnaA/SoxA family protein				
**MSMEG_0442**	K		**-1.08**	**-0.24**	tetracycline repressor domain-containing protein				
**MSMEG_1751**	C	RV2298	**-1.20**	**0.11**	norsolorinic acid reductase				
**MSMEG_6765**	V		**-1.15**	**-0.07**	ABC transporter				
**MSMEG_2894**	P		**-1.38**	**0.32**	steroid monooxygenase				
**MSMEG_4765**	K		**-1.30**	**0.07**	MerR family transcriptional regulator				
**MSMEG_5953**	E		**-1.06**	**-0.28**	hypothetical protein				
**MSMEG_0576**	R		**-1.50**	**0.02**	MmpL4 protein				
**MSMEG_4785**	Q		**-1.25**	**-0.35**	mce-family protein mce1f				
**MSMEG_1979**	S		**-1.37**	**0.40**	antibiotic biosynthesis monooxygenase				
**MSMEG_1301**	K		**-1.35**	**-0.36**	hypothetical protein				
**MSMEG_6774**	I		**-1.34**	**-0.07**	enoyl-CoA hydratase				
**MSMEG_5494**	I	Rv0972c	**-1.23**	**0.02**	acyl-CoA dehydrogenase				
**MSMEG_4087**	G		**-1.26**	**-0.19**	major facilitator superfamily protein				
**MSMEG_0333**	I		**-1.11**	**-0.24**	carboxyl transferase domain-containing protein				
**MSMEG_0284**	R		**-1.30**	**0.14**	ribosyldihydronicotinamide dehydrogenase				
**MSMEG_0484**	C		**-1.28**	**-0.12**	formamidase				
**MSMEG_0772**	S		**-1.32**	**0.13**	phytase				
**MSMEG_5573**	G		**-1.14**	**-0.16**	sugar ABC transporter permease				
**rplM**	J	Rv3443c	**-1.03**	**-0.38**	50S ribosomal protein L13				
**MSMEG_3449**	H		**-1.16**	**0.11**	DNA-binding protein				
**soxR**	K		**-1.32**	**0.42**	redox-sensitive transcriptional activator SoxR				
**MSMEG_1085**	P		**-1.13**	**-0.10**	dipeptide transporter permease DppB				
**MSMEG_4448**	T		**-1.01**	**-0.04**	transcriptional modulator of MazE				
**MSMEG_3248**	E		**-1.10**	**0.04**	branched-chain amino acid ABC transporter permease				
**MSMEG_2042**	R	RV3168	**-1.29**	**0.60**	phosphotransferase enzyme family protein				
**MSMEG_3583**	C		**-1.11**	**0.23**	monooxygenase				
**MSMEG_4048**	R		**-1.43**	**-0.19**	cyclase				

**MSMEG_2239**	K		-1.45	-0.36	TetR family transcriptional regulator					
**MSMEG_6469**	S	RV0140	-1.27	-0.17	hypothetical protein					
**MSMEG_4450**	E		-1.11	-0.02	alpha/beta hydrolase					
**MSMEG_5109**	R		-1.06	-0.89	hypothetical protein					
**MSMEG_6669**	R		-1.07	-0.01	ABC transporter permease					
**MSMEG_5565**	S		-1.35	-0.25	hypothetical protein					
**MSMEG_1306**	C		-1.53	0.36	aldehyde dehydrogenase					
**MSMEG_2171**	C		-1.44	0.53	L-carnitine dehydratase/bile acid-inducible protein F					
**MSMEG_5802**	R		-1.21	0.64	oxidoreductase, short chain dehydrogenase/reductase					
**MSMEG_5194**	G		-1.01	0.16	integral membrane protein					
**MSMEG_2978**	E		-1.33	0.16	ABC transporter ATP-binding protein					
**MSMEG_4335**	R		-1.06	0.47	diacylglycerol kinase					
**MSMEG_5523**	E		-1.05	-0.71	peptidase					
**MSMEG_6564**	K		-1.45	-0.54	TetR family transcriptional regulator					
**MSMEG_0148**	K		-1.41	-0.02	TetR family transcriptional regulator					
**MSMEG_6941**	R	RV3920C	-1.23	-0.20	R3H domain-containing protein					
**MSMEG_0128**	R	RV0163	-1.41	-0.17	thioesterase					
**MSMEG_4397**	K		-1.11	0.16	MerR family transcriptional regulator					
**hybA**	C		-1.39	-0.14	hydrogenase-2, small subunit					
**narI**	C	Rv1164	-1.78	-0.27	respiratory nitrate reductase subunit gamma					
**MSMEG_1977**	R		-1.32	-0.28	alcohol dehydrogenase					
**MSMEG_3905**	S	RV2117	-1.01	0.02	hypothetical protein					
**MSMEG_4140**	E		-1.38	0.39	GntR family transcriptional regulator					
**MSMEG_1281**	S		-1.07	0.13	hypothetical protein					
**MSMEG_2252**	C		-1.31	0.11	flavin-type hydroxylase					
**MSMEG_2879**	I		-1.31	-0.14	acyl-CoA dehydrogenase					
**MSMEG_2162**	C		-1.72	0.81	MmcJ protein					
**MSMEG_3301**	C		-1.40	-0.22	hypothetical protein					
**MSMEG_4798**	C		-1.01	-0.19	L-carnitine dehydratase/bile acid-inducible protein F					
**MSMEG_0485**	J		-1.14	0.60	amidase					
**MSMEG_5632**	R		-1.23	0.28	oxidoreductase, short chain dehydrogenase/reductase					
**MSMEG_4102**	I		-1.58	0.32	hypothetical protein					
**MSMEG_1173**	K	RV2642	-1.25	-0.02	ArsR family transcriptional regulator					
**MSMEG_0526**	E		-1.51	0.17	oxidoreductase, GMC family protein					
**oppD**	P	RV1281C	-1.30	0.15	ABC transporter ATP-binding protein					
**MSMEG_0817**	K		-1.01	0.07	LysR family transcriptional regulator					
**MSMEG_2241**	Q		-1.03	0.24	acyl-CoA synthetase					
**MSMEG_4155**	L		-1.20	0.21	transposase A					
**MSMEG_1125**	K	RV2621C	-1.14	0.01	ArsR family transcriptional regulator					
**MSMEG_1178**	K		-1.44	-0.46	transcriptional regulator					
**MSMEG_1509**	K		-1.17	-0.77	TetR family transcriptional regulator					
**MSMEG_2456**	C		-1.12	-0.17	5,10-methylenetetrahydromethanopterin reductase					
**MSMEG_6700**	K		-1.27	0.17	regulatory protein					
**MSMEG_1211**	I		-1.40	-0.21	Fatty acid desaturase					
**MSMEG_0839**	R	RV0434	-1.14	-0.22	ATP-dependent protease La					
**MSMEG_0912**	I	Rv0468	-1.15	0.34	3-hydroxybutyryl-CoA dehydrogenase					
**MSMEG_0428**	R	Rv0253	-1.40	0.11	nitrite reductase [NAD(P)H] small subunit					
**MSMEG_6780**	R		-1.47	-0.19	phenazine biosynthesis protein PhzF					
**MSMEG_6776**	K		-1.02	-0.13	LysR family transcriptional regulator					

**mtnN**	F		**-1.32**	**0.01**	MTA/SAH nucleosidase				
**MSMEG_5807**	P		**-1.11**	**-0.09**	D-methionine transport ATP-binding protein MetN				
**MSMEG_3369**	G		**-1.12**	**-0.31**	transporter				
**MSMEG_5479**	S	RV0991C	**-1.08**	**-0.58**	type I antifreeze protein				
**MSMEG_2039**	K		**-1.02**	**0.03**	transcriptional regulator				
**MSMEG_5112**	A		**-1.08**	**0.08**	hypothetical protein				
**MSMEG_6037**	R		**-1.21**	**-0.08**	2-hydroxy-6-ketonona-2,4-dienedioic acid hydrolase				
**MSMEG_5968**	R		**-1.22**	**0.00**	polysaccharide biosynthesis protein				
**dcd**	F	Rv0321	**-1.35**	**0.07**	deoxycytidine triphosphate deaminase				
**MSMEG_0532**	K		**-1.06**	**-0.62**	TetR family transcriptional regulator				
**MSMEG_5395**	T	Rv1028c	**-1.31**	**-0.16**	sensor protein KdpD				
**MSMEG_2666**	R	RV2771C	**-1.99**	**0.03**	multimeric flavodoxin WrbA				
**MSMEG_4823**	Q		**-1.11**	**0.29**	cytochrome p450				
**MSMEG_1158**	C		**-1.39**	**-0.07**	5-oxovalerate dehydrogenase				
**MSMEG_6510**	V		**-1.10**	**0.28**	multidrug ABC transporter				
**MSMEG_4002**	R		**-1.21**	**-0.17**	oxidoreductase, zinc-binding dehydrogenase				
**MSMEG_4858**	K		**-1.08**	**-0.16**	TetR family transcriptional regulator				
**MSMEG_3513**	R	Rv2416c	**-1.36**	**-0.08**	hypothetical protein				
**MSMEG_3402**	F		**-1.29**	**0.37**	cytosine permease				
**MSMEG_1975**	R		**-1.22**	**0.04**	amidohydrolase				
**MSMEG_2952**	G		**-1.01**	**-0.32**	transporter				
**MSMEG_2193**	K		**-1.35**	**0.07**	TetR family transcriptional regulator				
**MSMEG_3398**	G		**-1.16**	**-0.01**	integral membrane transporter				
**MSMEG_2600**	K		**-1.66**	**-0.10**	regulatory protein				
**MSMEG_0196**	C		**-1.30**	**0.32**	dehydrogenase				
**MSMEG_1074**	G		**-1.08**	**-0.13**	polysaccharide deacetylase				
**MSMEG_3348**	G		**-1.05**	**0.62**	extracellular solute-binding protein				
**MSMEG_5140**	C	Rv1161	**-1.06**	**-0.05**	nitrate reductase subunit alpha				
**MSMEG_1298**	R		**-1.17**	**-0.06**	guanine deaminase				
**MSMEG_4004**	R		**-1.29**	**0.01**	3-oxoacyl-ACP reductase				
**MSMEG_5778**	R		**-1.19**	**-0.13**	alcohol dehydrogenase				
**MSMEG_5931**	R	RV3530C	**-1.05**	**-0.07**	short chain dehydrogenase				
**MSMEG_6300**	K		**-1.36**	**0.15**	GntR family transcriptional regulator				
**MSMEG_5317**	A	RV0314C	**-1.22**	**-0.55**	hypothetical protein				
**MSMEG_5184**	C	Rv1143	**-1.34**	**0.09**	alpha-methylacyl-CoA racemase				
**MSMEG_4886**	G		**-1.72**	**-0.52**	major facilitator family protein transporter				
**MSMEG_0469**	K		**-1.78**	**-0.33**	PadR family transcriptional regulator				
**MSMEG_4439**	J		**-1.38**	**-0.70**	acetyltransferase				
**MSMEG_3823**	R		**-1.48**	**-0.14**	3-oxoacyl-ACP reductase				
**MSMEG_4795**	Q		**-1.10**	**-0.23**	ABC transporter				
**MSMEG_6643**	R	RV1129C	**-1.13**	**-0.36**	DNA-binding protein				
**MSMEG_3927**	C		**-1.23**	**-0.07**	peptidase M52, hydrogen uptake protein				
**MSMEG_1351**	M	Rv3392c	**-1.36**	**-0.18**	cyclopropane-fatty-acyl-phospholipid synthase				
**MSMEG_0217**	C		**-1.14**	**0.35**	alcohol dehydrogenase				
**MSMEG_1100**	S		**-1.36**	**0.31**	DGPFAETKE domain-containing protein				
**MSMEG_2286**	R		**-1.03**	**-0.08**	aminoglycoside phosphotransferase				
**MSMEG_0195**	P		**-1.29**	**-0.23**	steroid monooxygenase				
**MSMEG_5366**	G		**-1.09**	**1.39**	hypothetical protein				
**MSMEG_4014**	E		**-1.07**	**0.05**	N-carbamoyl-L-amino acid amidohydrolase				

**MSMEG_1235**	P		**-1.02**	**-0.17**	sulfate permease				
**MSMEG_4005**	G		**-1.20**	**0.00**	calcium-binding protein				
**MSMEG_5475**	K		**-1.09**	**-0.18**	acetate operon repressor				
**MSMEG_1254**	R		**-1.10**	**-0.83**	DEAD/DEAH box helicase				
**MSMEG_5291**	Q	Rv1058	**-1.15**	**-0.14**	long-chain-fatty-acid--CoA ligase				
**MSMEG_1745**	K		**-1.08**	**-0.41**	transcriptional regulator				
**MSMEG_1823**	I	RV3268	**-1.10**	**0.26**	hypothetical protein				
**MSMEG_4001**	G		**-1.07**	**-0.20**	ribose transporter permease RbsC				
**MSMEG_5950**	R		**-1.40**	**0.14**	colanic acid biosynthesis acetyltransferase WcaF				
**MSMEG_3965**	R		**-1.26**	**-0.49**	hypothetical protein				
**MSMEG_0777**	C	Rv0407	**-1.09**	**-0.39**	F420-dependent glucose-6-phosphate dehydrogenase				
**MSMEG_5458**	T	RV0998	**-1.03**	**-0.45**	cyclic nucleotide-binding protein				
**MSMEG_6002**	I	RV3551	**-1.23**	**0.13**	coenzyme A transferase subunit A				
**MSMEG_0390**	I		**-1.15**	**-0.35**	acyltransferase				
**MSMEG_6716**	G		**-1.42**	**-0.43**	AP endonuclease, family protein 2				
**ltrA**	S		**-1.36**	**-0.34**	low temperature requirement protein LtrA				
**MSMEG_4070**	K		**-1.21**	**0.11**	TetR family transcriptional regulator				
**MSMEG_4006**	Q		**-1.24**	**-0.32**	CdaR family transcriptional regulator				
**ackA**	C	Rv0409	**-1.26**	**-0.49**	acetate kinase				
**MSMEG_5612**	J		**-1.49**	**0.21**	amino-acid acetyltransferase				
**MSMEG_0513**	S		**-1.52**	**0.11**	integral membrane protein				
**MSMEG_0175**	E		**-1.09**	**-0.75**	FAD dependent oxidoreductase				
